# Mechanochemical Phosphorylation of Acetylides Using Condensed Phosphates: A Sustainable Route to Alkynyl Phosphonates

**DOI:** 10.1021/acscentsci.3c00725

**Published:** 2023-07-21

**Authors:** Tiansi Xin, Christopher C. Cummins

**Affiliations:** Department of Chemistry, Massachusetts Institute of Technology, Cambridge, Massachusetts 02139, United States

## Abstract

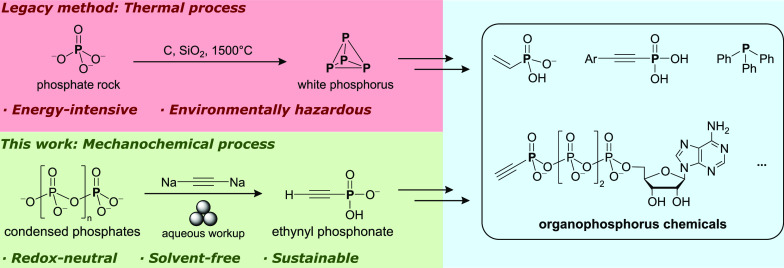

In pursuit of a more sustainable route to phosphorus–carbon (P–C) bond-containing chemicals, we herein report that phosphonates can be prepared by mechanochemical phosphorylation of acetylides using polyphosphates in a single step, redox-neutral process, bypassing white phosphorus (P_4_) and other high-energy, environmentally hazardous intermediates. Using sodium triphosphate (Na_5_P_3_O_10_) and acetylides, alkynyl phosphonates **1** can be isolated in yields of up to 32%, while reaction of sodium pyrophosphate (Na_4_P_2_O_7_) and sodium carbide (Na_2_C_2_) engendered, in an optimized yield of 63%, ethynyl phosphonate **2**, an easily isolable compound that can be readily converted to useful organophosphorus chemicals. Highly condensed phosphates like Graham’s salt and bioproduced polyphosphate were also found to be compatible after reducing the chain length by grinding with orthophosphate. These results demonstrate the possibility of accessing organophosphorus chemicals directly from condensed phosphates and may offer an opportunity to move toward a “greener” phosphorus industry.

## Abstract 2

Solvent-free mechanochemical phosphorylation of acetylides using condensed phosphates, a more sustainable route to alkynyl phosphonates and organophosphorus chemicals.

## Introduction

**Figure 1:**
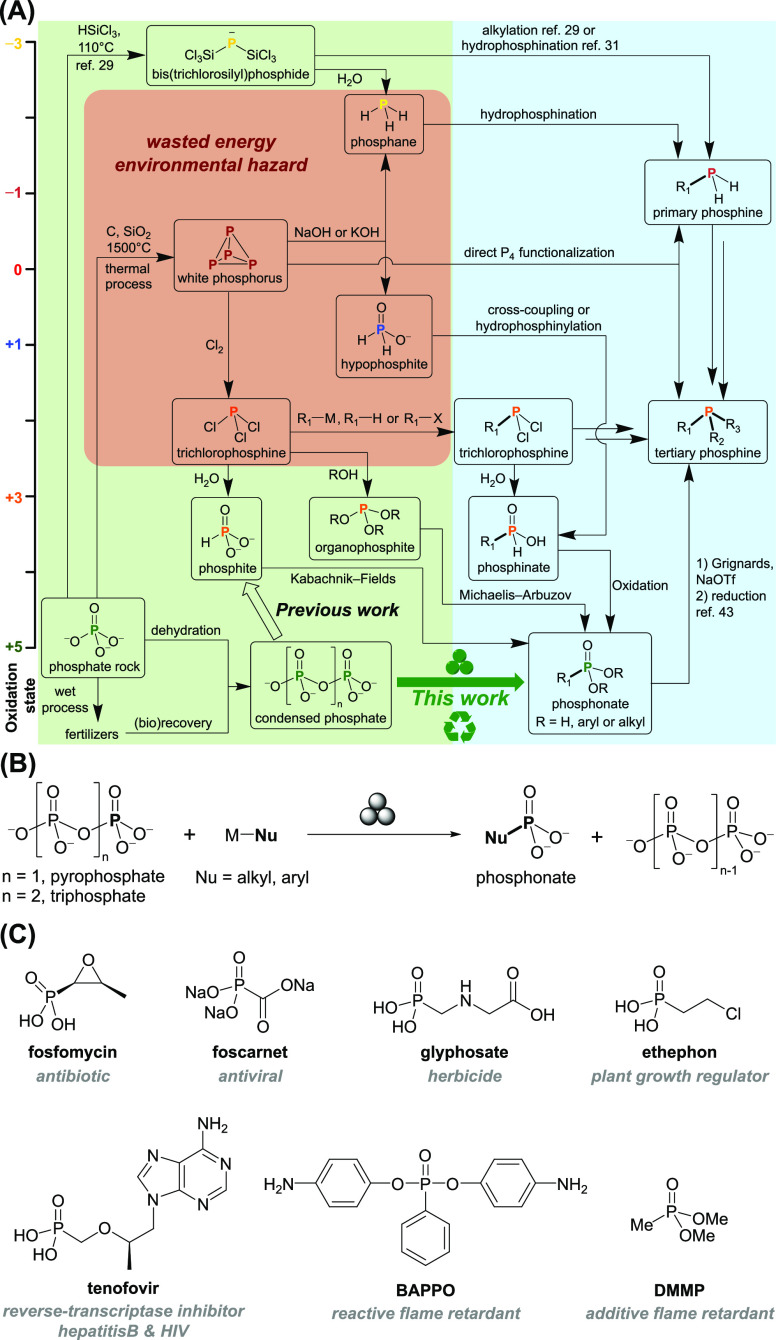
(A) Overview of key P–C bond formation steps in P-chemical manufacture. (B) The envisioned mechanochemical phosphorylation of carbon nucleophiles. (C) Selected examples of useful phosphonate-based organophosphorus chemicals.

Phosphorus–carbon (P–C) bonds are widely found in organophosphorus compounds that have emerged as useful pharmaceuticals, flame retardants, agrochemicals, ligands, and materials.^1−8^ At present, P–C bond-containing chemicals are derived almost exclusively from white phosphorus (P_4_), the tetrahedral molecule originally discovered by German alchemist Hennig Brand in 1669^9^ that has now become one of the most important feedstock materials in the modern phosphorus industry.^1,8,10^ Typically, mined phosphate rock goes through an energy intensive legacy process known as the “thermal process”, in which phosphate rock is treated with coke and sand at over 1500 °C to produce P_4_ (Figure 1A).^10^ The produced P_4_ is then oxidized to trichlorophosphine (PCl_3_) with chlorine gas (Cl_2_) and used in P–C bond forming reactions.^1,11^ Alternatively, P–C bonds can be accessed from hypophosphite (H_2_PO_2_^–^) and phosphane (phosphine gas, PH_3_) produced from P_4_ by cross-coupling or hydrophosphinylation^12,13^ and by hydrophosphination,^1,6,14,15^ respectively. Recent breakthroughs in P_4_ chemistry also allowed for direct functionalization of P_4_ into P–C bond-containing products.^16^

Although P_4_ remains at the center of P-chemical production, there are several drawbacks associated with its manufacture and utilization. The thermal process requires a massive amount of energy input for continuous electric arc furnace operation, limiting its facilities to regions with cheap sources of power.^10^ Furthermore, many substances involved in subsequent transformations, including Cl_2_, PCl_3_, PH_3_, and P_4_ itself, are environmentally hazardous and thus must be carefully regulated.^17,18^ In line with the principles of Green Chemistry^19^ and the United Nations’ Sustainable Development Goals,^20−22^ the issue of global phosphorus sustainability has received increasing attention over the past decade with the sustainable production of P-chemicals being one of its major targets.^23−26^ New strategies therefore need to be developed to address these energy and environmental issues.^27^

Another major portion of the mined phosphate rock, on the other hand, is treated with sulfuric acid to produce phosphoric acid and eventually fertilizers, in what is known as the “wet process”.^7^ Condensed phosphates (polyphosphates) can be prepared readily from phosphoric acid and its salts by dehydration.^7^ Apart from phosphate rock, phosphate removal protocols are being implemented worldwide to ameliorate eutrophication, and phosphates recovered from waste streams also constitute a new input stream of this nonrenewable resource.^23−26,28^ These phosphates can thus be considered as green starting materials.

Previously, we have shown that P–C bonds can be accessed from bis(trichlorosilyl)phosphide (Figure 1A, top), an intermediate directly prepared from phosphates, bypassing the hazardous P_4_.^29−31^ More recently, we demonstrated that phosphite can be produced from condensed phosphates and metal hydrides without traversing lower oxidation states than +3.^32,33^ This “hydride phosphorylation” breakthrough was made possible by mechanochemistry, an increasingly popular technique that is often recognized as green and sustainable.^34−41^ Moreover, polyphosphates recovered from microorganisms were also shown to be promising substrates.^32^

Building upon these principles, we sought to expand this redox-neutral mechanochemical phosphorylation to carbon nucleophiles and achieve direct P–C bond formation from polyphosphates (Figure 1B). The product phosphonate is found in many drugs, agrochemicals, and flame retardants (Figure 1C) and is currently manufactured starting from P_4_.^5,8,42^ Recent advances also enabled direct preparation of tertiary phosphines from organophosphonates.^43^

## Results and Discussion

**Figure 2:**
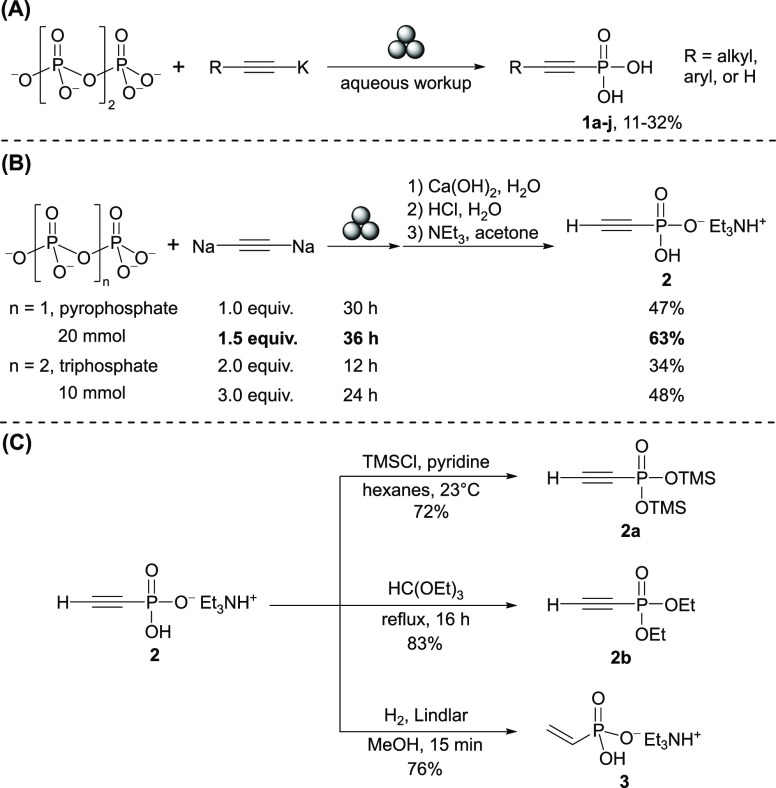
(A) Synthesis of alkynyl phosphonates **1a**–**j**. (B) Synthesis of ethynyl phosphonate **2**, yield based on reducible phosphate (1 per Na_4_P_2_O_7_, 2 per Na_5_P_3_O_10_). (C) Synthesis of **2a**, **2b**, and **3** from **2**.

With the knowledge gained from the hydride phosphorylation reaction, we started out by exploring the mechanochemical reaction between sodium triphosphate (Na_5_P_3_O_10_) and common organometallic reagents (Supporting Information). Mechanochemical reactions were conducted in a Restch PM100 planetary ball mill using stainless steel jars and ball bearings at a rotational frequency of 450 rpm. We found that, among the tested organometallic reagents, phenylacetylide gave better results than the rest, with the best phosphonate yield of 33% achieved when using potassium phenylacetylide. Extending the substrates to common alkyl and aryl acetylides also afforded the corresponding alkynyl phosphonates **1a**–**j** but in rather poor isolated yields possibly due to side reactions of acetylides under mechanochemical conditions (Figure 2A; see the Supporting Information for more details).

In order to extend the synthetic applications of this “acetylide phosphorylation” reaction, we then targeted ethynyl phosphonate (HCCPO_3_^2–^), a phosphonate with a terminal alkyne that allows for further functionalizations. Our first experiment involving treatment of Na_5_P_3_O_10_ with stoichiometric sodium acetylide (NaCCH) did not lead to the desired product, possibly due to the decomposition of NaCCH caused by deprotonation. To our delight, switching to sodium carbide (Na_2_C_2_) resulted in clean formation of HCCPO_3_^2–^ with an isolated yield of 34% after an aqueous workup, which could be improved to 48% when a higher carbide loading (Na_2_C_2_:reducible P = 1.5:1; for the definition of reducible P, see ref (32)) was used (Figure 2B). Sodium pyrophosphate (Na_4_P_2_O_7_) was found to give higher yields after elongated grinding times, with the best of 63% isolated yield achieved with the same carbide loading. Analytically pure ethynyl phosphonate can be isolated as the triethylammonium salt **2** simply by precipitation and recrystallization, while previous reports of its synthesis noted the formation of substantial side products and necessitated HPLC separation.^44,45^ The obtained **2** can be readily silylated or ethylated to afford the corresponding ester **2a** or **2b**, which can be used in further transformations (Figure 2C). More importantly, **2** can be partially hydrogenated to vinyl phosphonate **3** (Figure 2C), a useful monomer in the polymer industry for production of electrolyte membranes and other materials.^46^

Having isolated pure **2** in decent yields, we envision that **2** (and its derivatives **2a** and **2b**) can now serve as a new, sustainable starting material for P–C bond containing chemicals. Treatment of **2a** with aryl iodides under typical Sonogashira coupling conditions^47,48^ led to more alkynyl phosphonates (Table 1), including ones bearing functional groups, such as carboxylate ester, nitro, aldehyde and borate, that are typically not compatible with acetylides in ball milling. Use of diiodides and triiodides also afforded the corresponding bis- and tris-phosphonates, which may find applications as useful secondary building units in the construction of metal–organic framework (MOF) materials.^49−53^

**Table 1:**
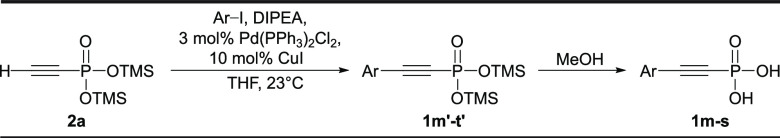

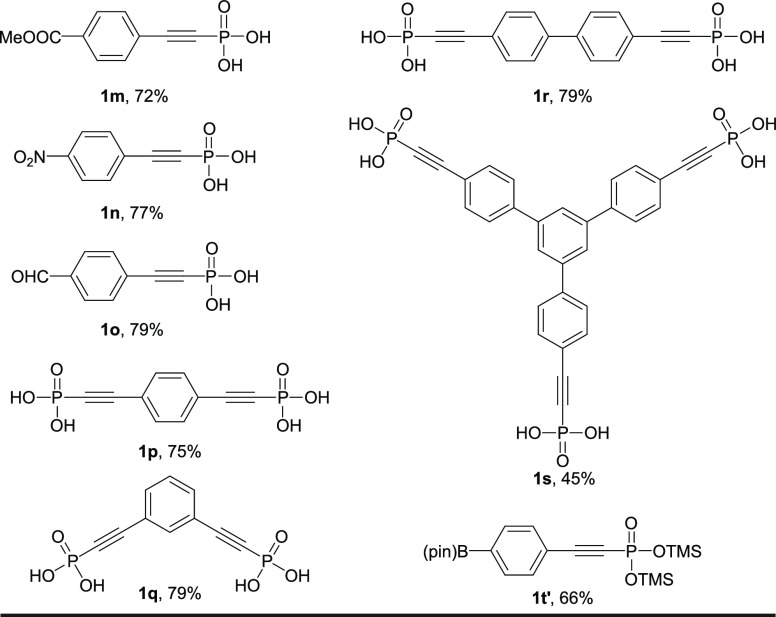
Synthesis of Alkynylphosphonic Acids from **2a**^a^

a Reactions carried out on a 0.3 mmol scale. Yield over two steps except for **1t′**.

**Figure 3:**
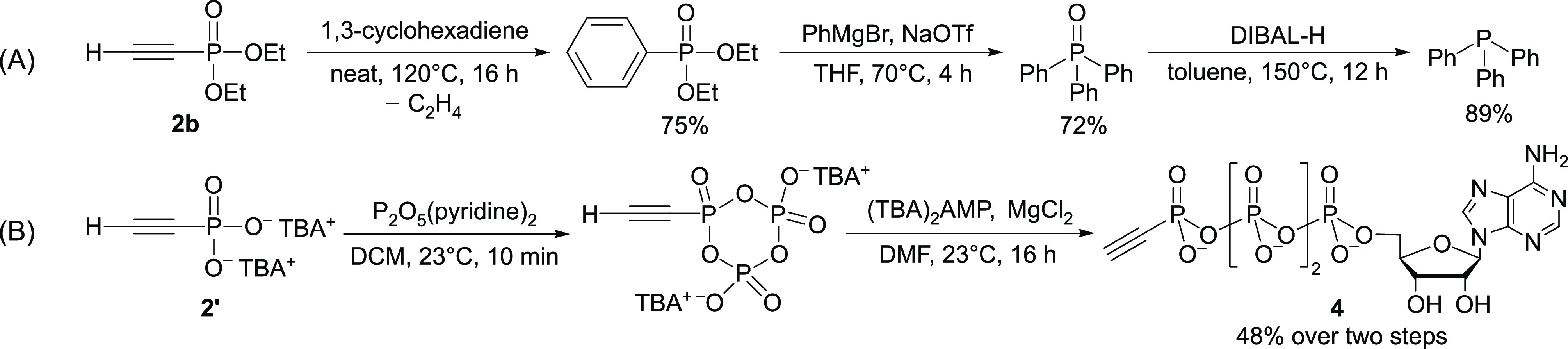
Synthesis of triphenylphosphine from **2b** (A) and synthesis of **4** from **2′** (B).

In addition, phenyl rings can be constructed from **2b** and cyclohexa-1,3-diene by Diels–Alder reaction with ethylene elimination (Figure 3A).^54^ The obtained phenyl phosphonate can be directly converted to triphenyl phosphine (PPh_3_) by treatment with the phenyl Grignard reagent and NaOTf followed by reduction.^43^ This is, to the best of our knowledge, the first example of PPh_3_ synthesis without the involvement of P_4_. There are many established procedures that convert **2b** into other useful organophosphorus compounds as well.^55−68^ Moreover, ethynyl phosphonate offers opportunities for terminal phosphate modification of bioactive molecules. Nucleoside tetraphosphate **4** featuring a “clickable” moiety, for example, can be readily synthesized from the TBA salt **2′** using a diphosphorylation protocol recently developed by our group (Figure 3B).^69^ A number of such modified nucleotide analogues have found applications as probes to investigate biological processes and as tools for biotechnology and drug discovery.^45,70−74^ Similar strategies were also developed for labeling amino acids and peptides using ethynyl phosphonate.^75−78^

**Figure 4:**
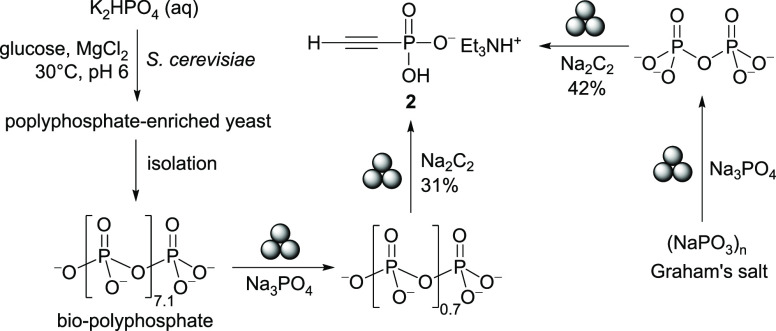
Synthesis of ethynyl phosphonate **2** from bio-polyP and Graham’s salt; yield is based on reducible phosphate.

Lastly, we turned our attention to bioproduced condensed phosphates. Microorganisms are known to take up phosphate from their surroundings and store it as intracellular polyphosphate granules,^79,80^ forming the basis of the enhanced biological phosphorus removal (EBPR) process.^81,82^ A recent protocol of polyphosphate accumulation using *Saccharomyces cerevisiae* (baker’s yeast) allowed us to expand our mechanochemical phosphorylation to bioproduced polyphosphate (bio-polyP) of similar properties to what is recovered from waste streams by EBPR.^32,83−86^ As shown in Table S2, highly condensed phosphates like Graham’s salt afford the desired alkynyl phosphonates in poor yields. We therefore sought to break down these highly condensed phosphates into pyrophosphate, which has been shown to be a superior phosphorylation reagent. Treating Graham’s salt with stoichiometric sodium phosphate Na_3_PO_4_ under typical ball-milling conditions led to the clean formation of pyrophosphate, and subsequent reaction with Na_2_C_2_ afforded **2** in 42% yield (Figure 4, right). Similarly, a bio-polyP with an average chain length of 8.1 could be broken down to a phosphate mixture with an average chain length of 1.7. Ethynyl phosphonate **2** could be prepared from this mixture in 31% yield (Figure 4, left). These initial results demonstrate that polyphosphates recovered from microorganisms could be promising starting materials for sustainable production of P-chemicals, presenting an opportunity for a “closed-loop” phosphorus industry.^25^

## Conclusions

We have achieved direct P–C bond formation from condensed phosphates via mechanochemical phosphorylation of common acetylides. This new method bypasses white phosphorus as a hazardous intermediate while replacing the carbon-intensive thermal process with a green, sustainable mechanochemical process. Pyrophosphate Na_4_P_2_O_7_ was found to be the optimal phosphorylation reagent for Na_2_C_2_ to afford ethynyl phosphonate **2**, an easily isolable compound that is converted readily to useful organophosphorus chemicals. Bioproduced polyphosphate was also found to be a suitable phosphate source after breaking it down to pyrophosphate by grinding with orthophosphate. With mechanochemistry becoming more common in industrial chemistry,^87^ this study presents a new entry point into organophosphorus chemical production as an alternative to white phosphorus.

## Supporting Information Available

General information, experimental procedures and results, characterization data, copies of ^1^H, ^13^C and ^31^P NMR spectra, and references (PDF)
