# Supplementary material for: Mechanochemical Phosphorylation of Acetylides Using Condensed Phosphates: A Sustainable Route to Alkynyl Phosphonates
Source: ACS Cent Sci. 2023 Jul 21;9(8):1575–80. doi: 10.1021/acscentsci.3c00725 (PMC10451036; doi:10.1021/acscentsci.3c00725)
Supplement: Supplementary file 1 — oc3c00725_si_001.pdf [file oc3c00725_si_001.pdf]

Supporting Information: Mechanochemical Phosphorylation of Acetylides Using Condensed Phosphates: a Sustainable Route to Alkynyl Phosphonates

Tiansi Xin and Christopher C. Cummins*

Department of Chemistry, Massachusetts Institute of Technology, Cambridge, MA 02139, USA

E-mail: ccummins@mit.edu

Contents

S1 General considerations **S6**

S2 Reaction screening **S8**

S2.1 Nucleophile screening S8

S2.2 Phosphate screening S8

S2.3 Ethynyl phosphonate condition screening S12

S2.4 Typical spectra S14

S3 Synthesis of alkynylphosphonic acids from acetylides **S26**

S3.1 Preparation of acetylides S26

S3.2 General procedure S26

S3.3 Synthesis of alkynylphosphonic acids **1a–j** S28

S3.3.1 (Prop-1-yn-1-yl)phosphonic acid **1a** S29

S3.3.2 (Hex-1-yn-1-yl)phosphonic acid **1b** S29

S3.3.3 (3,3-Dimethylbut-1-yn-1-yl)phosphonic acid **1c** S30

S3.3.4 (Cyclopropylethynyl)phosphonic acid **1d** S30

S3.3.5 (Cyclohex-1-en-1-ylethynyl)phosphonic acid **1e** S31

S3.3.6 Octa-1,7-diyne-1,8-diylbis(phosphonic acid) **1f** S31

S3.3.7 Ethyne-1,2-diylbis(phosphonic acid) **1g** S32

S3.3.8 (Phenylethynyl)phosphonic acid **1h** S32

S3.3.9 ((4-Fluorophenyl)ethynyl)phosphonic acid **1i** S33

S3.3.10 ((4-Bromophenyl)ethynyl)phosphonic acid **1j** S33

S3.3.11 Attempted synthesis of (3,3-diethoxyprop-1-yn-1-yl)phosphonic acid **1k** S34

S3.3.12 Attempted synthesis of (3-((tetrahydro-2*H*-pyran-2-yl)oxy)prop-1-yn-1-yl)phosphonic acid **1l** S34

S4 Synthesis of ethynyl phosphonate S69

S4.1 Triethylammonium hydrogen ethynyl phosphonate **2** S69

S4.2 Bis(triethylammonium) ethynyl phosphonate **2″** S71

S4.3 Bis(tetra-*n*-butylammonium) ethynyl phosphonate **2′** S72

S5 Functionalization of ethynyl phosphonate S81

S5.1 Synthesis of bis(trimethylsilyl) ethynyl phosphonate **2a** S81

S5.2 Synthesis of diethyl ethynyl phosphonate **2b** S81

S5.3 Synthesis of vinyl phosphonate **3** from **2** S82

S5.4 Synthesis of triphenylphosphine from **2b** S83

S5.4.1 Synthesis of diethyl phenyl phosphonate S83

S5.4.2 Synthesis of triphenylphosphine oxide S83

S5.4.3 Synthesis of triphenylphosphine S84

S5.5 Synthesis of **4** from **2b** S84

S6 Synthesis of alkynylphosphonic acids from 2a S95

S6.1 General procedure S95

S6.2 Synthesis of bis(trimethylsilyl) alkynyl phosphonates **1m–s′** S95

S6.2.1 Bis(trimethylsilyl) ((4-(methoxycarbonyl)phenyl)ethynyl)phosphonate **1m′** S95

S6.2.2 Bis(trimethylsilyl) ((4-nitrophenyl)ethynyl)phosphonate **1n′** . . . S96

S6.2.3 Bis(trimethylsily) ((4-formylphenyl)ethynyl)phosphonate **1o′** . . . S96

S6.2.4 Tetrakis(trimethylsilyl) (1,4-phenylenebis(ethyne-2,1-diyl))bis(phosphonate) **1p′** S97
S6.2.5 Tetrakis(trimethylsilyl) (1,3-phenylenebis(ethyne-2,1-diyl))bis(phosphonate) **1q′** S97
S6.2.6 Tetrakis(trimethylsilyl) ([1,1’-biphenyl]-4,4’-diylbis(ethyne-2,1-diyl))-bis(phosphonate) **1r′** S98
S6.2.7 Hexakis(trimethylsilyl) [benzene-1,3,5-triyltris(4,1-phenyleneethyne-2,1-diyl)]tris(phosphonate) **1s′** S98
S6.2.8 Bis(trimethylsilyl) ((4-(tetramethyldioxaborolanyl)phenyl)ethynyl)phosphonate **1t′** S99
S6.3 Syntheis of alkynylphosphonic acids **1m–s** S100
S6.3.1 ((4-(methoxycarbonyl)phenyl)ethynyl)phosphonic acid **1m** S100
S6.3.2 ((4-nitrophenyl)ethynyl)phosphonic acid **1n** S100
S6.3.3 ((4-formylphenyl)ethynyl)phosphonic acid **1o** S101
S6.3.4 1,4-phenylenebis(ethyne-2,1-diyl))bis(phosphonic acid) **1p** S101
S6.3.5 1,3-phenylenebis(ethyne-2,1-diyl))bis(phosphonic acid) **1q** S101
S6.3.6 ([1,1’-biphenyl]-4,4’-diylbis(ethyne-2,1-diyl))bis(phosphonic acid) **1r** S102
S6.3.7 ([benzene-1,3,5-triyltris(4,1-phenyleneethyne-2,1-diyl)]tris(phosphonic acid) **1s** S102

S7 Synthesis of 2 from Graham’s salt and bio-polyP S149
S7.1 Breaking down Graham’s salt S149
S7.2 Synthesis of **2** from Graham’s salt S149
S7.3 Breaking down bio-polyP S150

S7.4 Synthesis of **2** from bio-polyP S150

S8 References S155

S1 General considerations

Air-sensitive manipulations were performed in a Vacuum Atmospheres model MO-40M glovebox under an inert atmosphere of purified N_2 or using standard Schlenk techniques. Mechanochemical reactions were performed in a 125 mL stainless steel milling jar (Retsch, part number: 01.462.0148) equipped with a safety closure device (Retsch, part number: 22.867.0007) and thirty 10 mm stainless steel balls (Retsch, part number: 05.368.0063) on a Retsch PM 100 planetary ball mill. No unexpected or unusually high safety hazards were encountered.

Dry solvents used were obtained anhydrous and oxygen-free by bubble degassing (argon) and purified by passing through columns of alumina and Q5.[S1] Once collected, solvents were stored over activated 4 Å molecular sieves (20 wt%) inside the glovebox.[S2] All glassware for air-sensitive manipulations was oven-dried for at least 6 h prior to use, at temperatures greater than 150 °C. Deuterated solvents were purchased from Cambridge Isotope Labs and used as received. Column chromatography purifications were performed on silica gel 60 Å (VWR North America). Ion-exchange columns were performed on Amberchrom (formerly DOWEX) 50WX8 (H^+) resin (Sigma-Millipore), which was regenerated with excess 1 M HCl after each use.

Sodium tripolyphosphate ($Na_5P_3O_{10}$, ≥98%, Sigma-Millipore), sodium hexametaphosphate (Graham's salt, CAS 10124-56-8, technical grade, Alfa Aesar), sodium phosphate (Na_3PO_4, 96%, Sigma-Millipore) and sodium fluorophosphate (Na_2PO_3F, 95%, Sigma-Millipore) were dried under dynamic vacuum at 200 °C for 24 h and stored in the glovebox. Anhydrous sodium pyrophosphate ($Na_4P_2O_7$) was prepared by heating sodium pyrophosphate tetrabasic decahydrate ($Na_4P_2O_7 \cdot 10H_2O$, ≥99.0%, Sigma-Millipore) at 150 °C in an oven to constant mass and stored in the glovebox. Sodium acetylide was isolated from

its suspension (18 wt% in mineral oil, Sigma-Millipore) by washing with dry hexanes followed by filtration in the glovebox and drying under dynamic vacuum. Sodium carbide (Na_2C_2) was synthesized by heating sodium acetylide at >150 °C under dynamic vacuum for more than 12 h according to a literature procedure and used without further purification.[S3] Unless otherwise noted, all other chemicals were purchased commercially and used as received.

NMR spectra were obtained on 400 MHz Bruker Avance or 500 MHz Bruker Neo spectrometers. ^1H and ^{13}C NMR spectra were referenced to residual solvent peaks: D_2O (^1H = 4.79 ppm), $CDCl_3$ (^1H = 7.26 ppm, ^{13}C = 77.16 ppm), CD_3CN (^1H = 1.94 ppm, ^{13}C = 1.32, 118.26 ppm). ^{19}F NMR spectra were referenced externally to $CFCl_3$ (0 ppm). ^{31}P NMR spectra were referenced externally to 85% H_3PO_4 (0 ppm). Parameters for ^{31}P NMR spectroscopic data collection in yield determination were as follows: 30° pulse, acquisition time (3.0 s), scan delay (40 s, vide infra), 16 scans, and 25 °C. Combustion analysis (for C, H, N) was performed at Midwest Micro Laboratories (Indianapolis, IN, USA). Electrospray ionization mass spectrometry (ESI-MS) was performed using a Micromass Q-TOF ESI spectrometer. High resolution mass spectral (HRMS) data were collected using a Jeol AccuTOF 4G LC-Plus mass spectrometer equipped with an Ion-Sense DART source. Data were calibrated to a sample of PEG-600 and were collected in positive-ion mode.

S2 Reaction screening

S2.1 Nucleophile screening

In the glovebox, a 125 mL stainless steel milling jar was charged with $Na_5P_3O_{10}$ (1.84 g, 5.0 mmol, 3.0 equiv), the organometallic reagent (1.7 mmol, 1.0 equiv, based on carbon anion) and 30 grinding balls. The milling jar was sealed with the safety closure device, removed from the glovebox, and mounted onto the planetary ball mill. Milling was first conducted at a rotational speed of 200 rpm for 10 min to mix up the starting materials, and then conducted at 450 rpm for 2 h. After cooling to ambient temperature, the jar was brought back into the glovebox and opened. An aliquot (ca. 400 mg) was removed from the jar and transferred into a 20 mL scintillation vial. The vial was brought out of the glovebox and quenched with ice water (ca. 8 mL). $NaHCO_3$ (ca. 300 mg) was added to neutralize the solution. All solid material was dissolved with the help of sonication. A known amount of triethyl phosphate ($PO(OEt)_3$, ca. 100 mg) was added to the solution as an internal standard and dissolved. An aliquot of the resulting solution was transferred to an NMR tube. D_2O (ca. 0.1 mL) was added and the solution was analyzed by ^{31}P NMR spectroscopy. The ratio of the phosphonate product to $PO(OEt)_3$ was estimated from the relative integration of the observed signals and the yields were calculated. Results are summarized in Table S1. Typical spectra are shown in Section S2.4.

S2.2 Phosphate screening

In the glovebox, a 125 mL stainless steel milling jar was charged with phosphate, potassium phenylacetylide and 30 grinding balls. The milling jar was sealed with the safety closure device, removed from the glovebox, and mounted onto the planetary ball mill.

Table S1: Organometallic compounds screened for mechanochemical phosphorylation

entry	nucleophile	time (h)	phosphonate yield (%)	entry	nucleophile	time (h)	phosphonate yield (%)
**1**	$LiCH_3$	2	7	**8**	$Mg(C_6H_5)_2$	2	10
**2**	$NaCH_3$	2	9	**9**	$Na(CH_2)CH_3$	2	3
**3**	$NaCH_3$	4	8	**10**	$KCH_2C_6H_5$	2	0
**4**	KCH_3	2	9	**11**	$LiC{\equiv}CC_6H_5$	2	0
**5**	$Mg(CH_3)_2$	2	11	**12**	$NaC{\equiv}CC_6H_5$	2	25(22[a])
**6**	$Mg(CH_3)_2$	4	10	**13**	$KC{\equiv}CC_6H_5$	2	33(31[a])
**7**	NaC_6H_5	2	11	**14**	$KC{\equiv}CC_6H_5$	4	22

[a] Isolated yield.

Milling was first conducted at a rotational speed of 200 rpm for 10 min to mix up the starting materials, and then conducted at 450 rpm for the specified time. After cooling to ambient temperature, the jar was brought back into the glovebox and opened. An aliquot (ca. 400 mg) was removed from the jar and transferred into a 20 mL scintillation vial. The vial was brought out of the glovebox and quenched with ice water (ca. 8 mL). $NaHCO_3$ (ca. 300 mg) was added to neutralize the solution. All solid material was dissolved with the help of sonication. A known amount of triethyl phosphate ($PO(OEt)_3$, ca. 100 mg) was added to the solution as an internal standard and dissolved. An aliquot of the resulting solution was transferred to an NMR tube. D_2O (ca. 0.1 mL) was added and the solution was analyzed by ^{31}P NMR spectroscopy. The ratio of the phosphonate product to $PO(OEt)_3$ was estimated from the relative integration of the observed signals and the yields were calculated. Results are summarized in Table S2. Typical spectra are shown in Section S2.4. The results suggested that $Na_5P_3O_{10}$ is a better phosphate

source. Neither elongating milling time nor increasing the phosphate:acetylide ratio led to an increased phosphonate yield (entry 3–8), suggesting that the acetylide was mostly consumed at the end of the ball milling process. Extraction of a quenched reaction mixture of entry 4 with DCM afforded a black solid after removing DCM under reduced pressure, which was analyzed by ^{1}H and ^{31}P NMR. Apart from some phenylacetylene, there were broad signals in the aromatic region in the ^{1}H NMR spectrum as well as some unidentified peaks (Figure S12), while no signals were found in the ^{31}P NMR spectrum. These results may suggest that oligomerization and polymerization could be the major side reactions of phenylacetylide in ball milling, which accounts for the low phosphonate yields.

Table S2: Phosphates screened for mechanochemical phosphorylation

entry	phosphate	phosphate amount (mmol)	acetylide amount (mmol)	time (h)	phosphonate yield (%)
**1**	$Na_4P_2O_7$	5.0	1.0	2	3
**2**	$Na_4P_2O_7$	5.0	1.7	2	5
**3**	$Na_5P_3O_{10}$	5.0	0.5	2	21
**4**	$Na_5P_3O_{10}$	5.0	0.5	4	16
**5**	$Na_5P_3O_{10}$	5.0	1.0	2	31
**6**	$Na_5P_3O_{10}$	5.0	1.0	4	20
**7**	$Na_5P_3O_{10}$	5.0	1.7	2	33(31[a])
**8**	$Na_5P_3O_{10}$	5.0	1.7	4	22
**9**	Na_2PO_3F	5.0	1.0	2	20
**10**	Na_2PO_3F	5.0	1.7	2	27
**11**	$Na_3P_3O_9$	5.0	1.0	2	1
**12**	$Na_3P_3O_9$	5.0	1.7	2	1
**13**	Graham's salt	5.0[b]	1.0	2	7
**14**	Graham's salt	5.0[b]	1.7	2	9

[a] Isolated yield. [b] Based on $NaPO_3$.

S2.3 Ethynyl phosphonate condition screening

In the glovebox, a 125 mL stainless steel milling jar was charged with phosphate, Na_2C_2 and 30 grinding balls. The milling jar was sealed with the safety closure device, removed from the glovebox, and mounted onto the planetary ball mill. Milling was first conducted at a rotational speed of 200 rpm for 10 min to mix up the starting materials, and then conducted at 450 rpm for the specified time. After cooling to ambient temperature, **2** was isolated as described in S4.1 and yield was calculated. Results are summarized in Table S3.

Table S3: Conditions screened for ethynyl phosphonate **2** synthesis

entry	phosphate	phosphate amount (mmol)	Na_2C_2 amount (mmol)	time (h)	phosphonate yield (%)
**1**	$Na_4P_2O_7$	20	20	30	47
**2**	$Na_4P_2O_7$	20	30	36	63
**3**	$Na_5P_3O_{10}$	10	20	12	34
**4**	$Na_5P_3O_{10}$	10	30	24	48
**5**	Na_2PO_3F	20	30	8	32
**6**	$Na_3P_3O_9$	10	30	8	18
**7**	Graham's salt	20[a]	30	8	15
**8**	$Na_4P_2O_7$	20	30[b]	8	0
**9**	$Na_4P_2O_7$	20	30[c]	24	32
**10**	$Na_4P_2O_7$	20	20	30[d]	38
**11**	$Na_4P_2O_7$	20	20	40[e]	45
**12**	$Na_4P_2O_7$	20	20	72[f]	18

[a] Based on $NaPO_3$. [b] NaCCH was used instead of Na_2C_2. [c] CaC_2 was used instead of Na_2C_2. [d] Reaction conducted at 600 rpm. [e] Reaction conducted at 400 rpm. [f] Reaction conducted at 300 rpm.

S2.4 Typical spectra

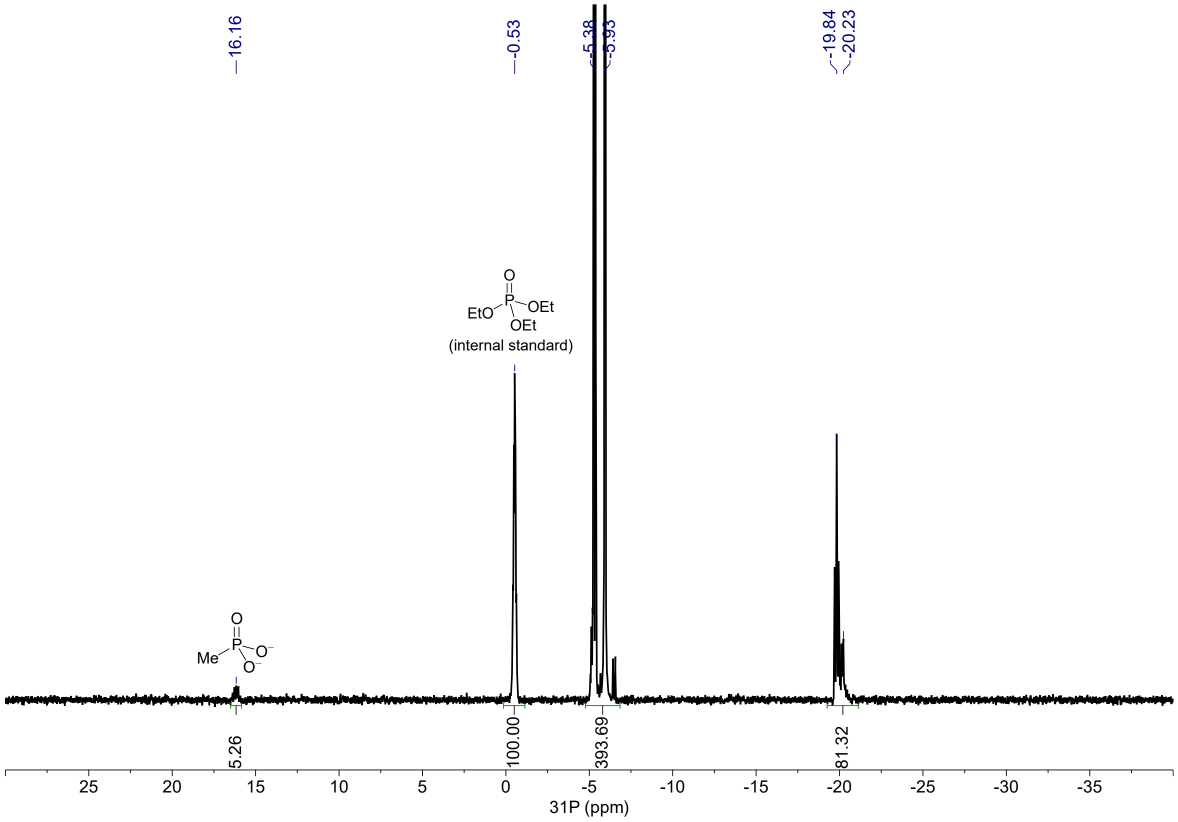

Figure S1: ^{31}P NMR spectrum of the mixture resulting from reaction of $Na_5P_3O_{10}$ and $NaCH_3$ after 2 h (Table S1 entry 2, 504 mg of crude solid with 137 mg of $PO(OEt)_3$ added).

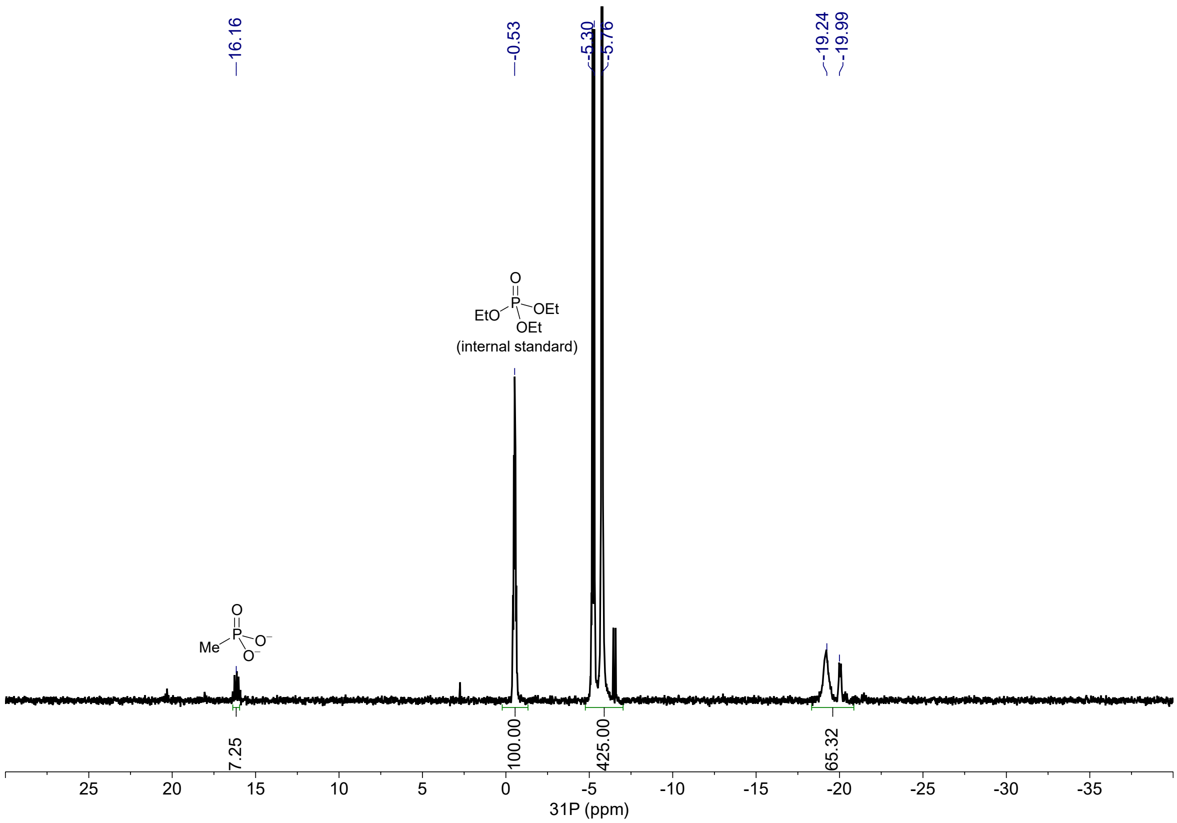

Figure S2: ^{31}P NMR spectrum of the mixture resulting from reaction of $Na_5P_3O_{10}$ and $Mg(CH_3)_2$ after 2 h (Table S1 entry 5, 530 mg of crude solid with 135 mg of $PO(OEt)_3$ added).

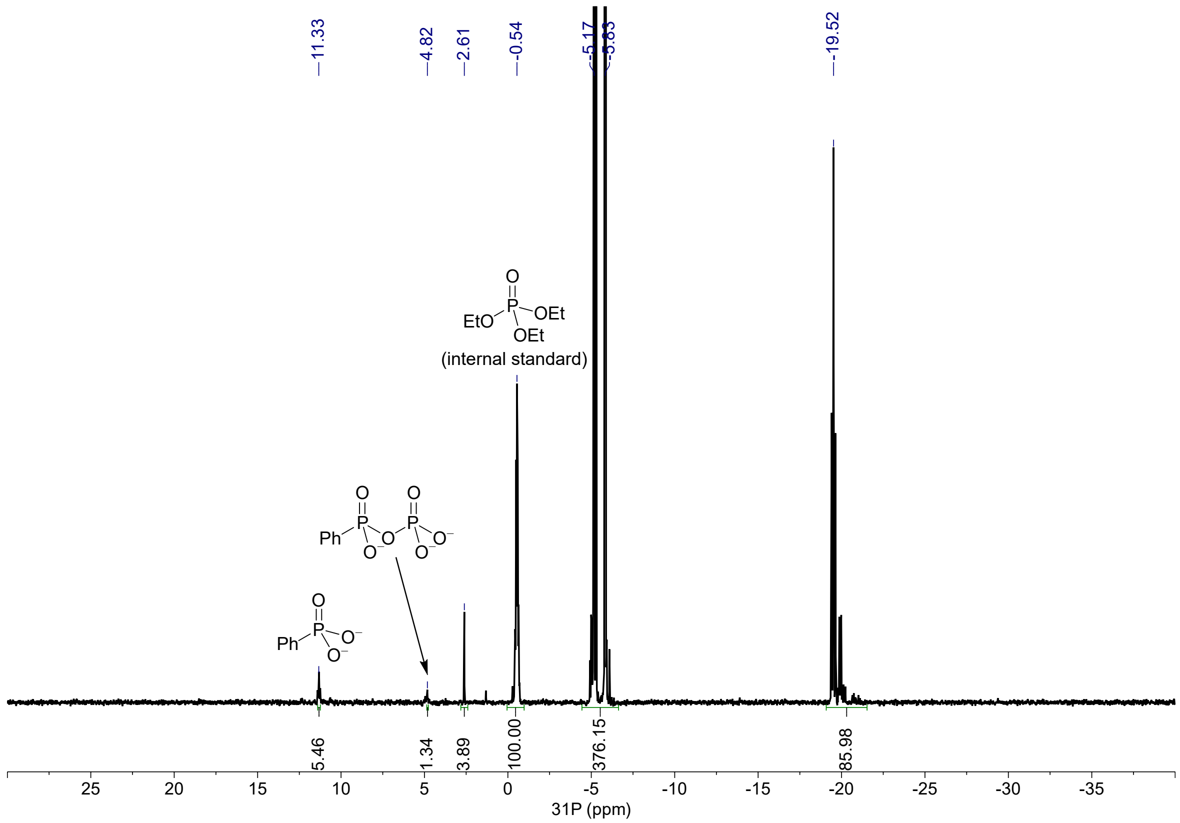

Figure S3: ^{31}P NMR spectrum of the mixture resulting from reaction of $Na_5P_3O_{10}$ and NaC_6H_5 after 2 h (Table S1 entry 7, 362 mg of crude solid with 96 mg of $PO(OEt)_3$ added).

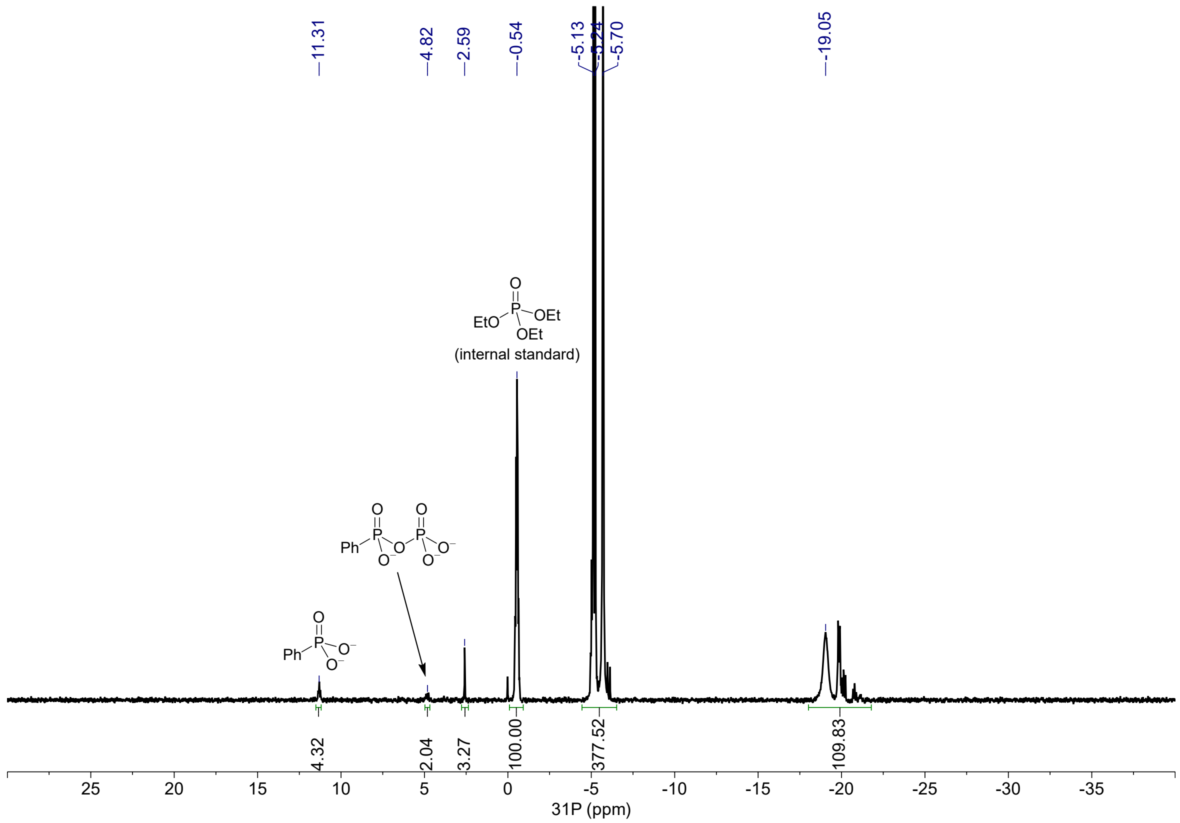

Figure S4: ^{31}P NMR spectrum of the mixture resulting from reaction of $Na_5P_3O_{10}$ and $Mg(C_6H_5)_2$ after 2 h (Table S1 entry 8, 448 mg of crude solid with 110 mg of $PO(OEt)_3$ added).

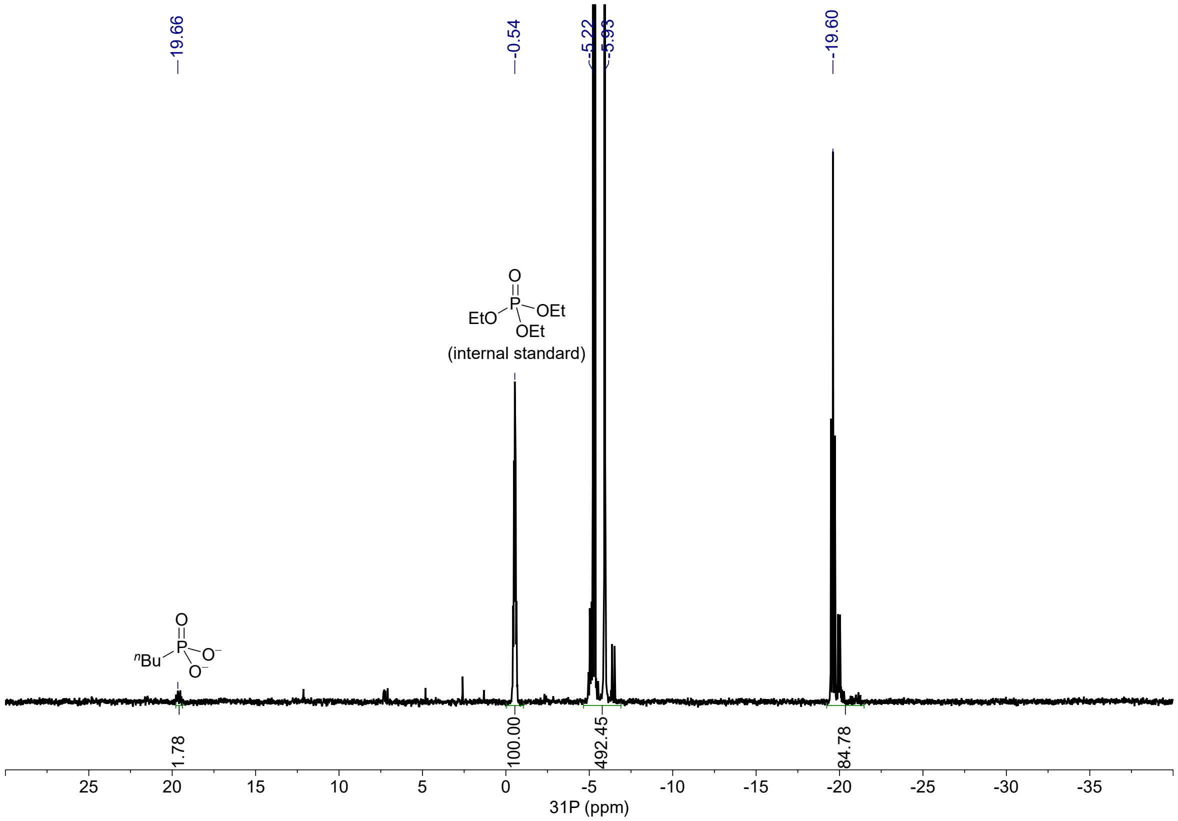

Figure S5: ^{31}P NMR spectrum of the mixture resulting from reaction of $Na_5P_3O_{10}$ and $Na(CH_2)_3CH_3$ after 2 h (Table S1 entry 9, 440 mg of crude solid with 101 mg of $PO(OEt)_3$ added).

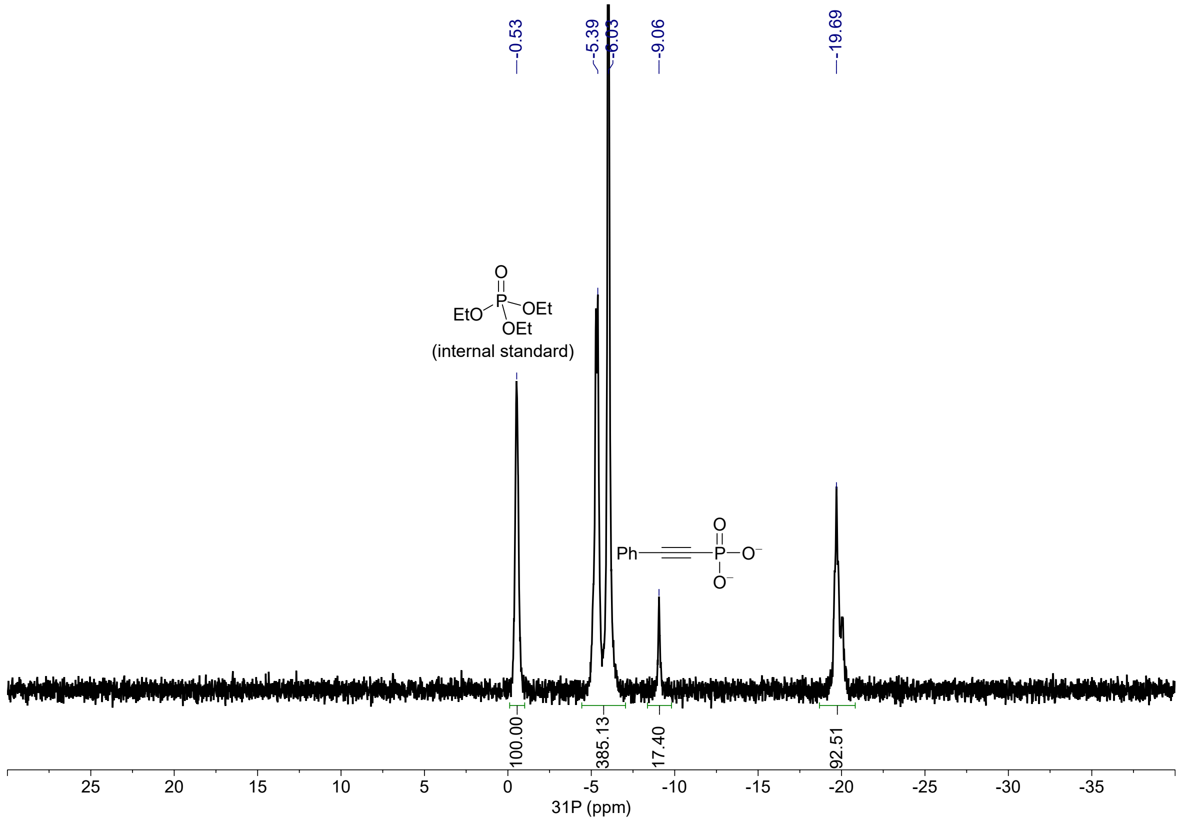

Figure S6: ^{31}P NMR spectrum of the mixture resulting from reaction of $Na_5P_3O_{10}$ and $KC{\equiv}CC_6H_5$ after 2 h (Table S1 entry 13, 381 mg of crude solid with 105 mg of $PO(OEt)_3$ added).

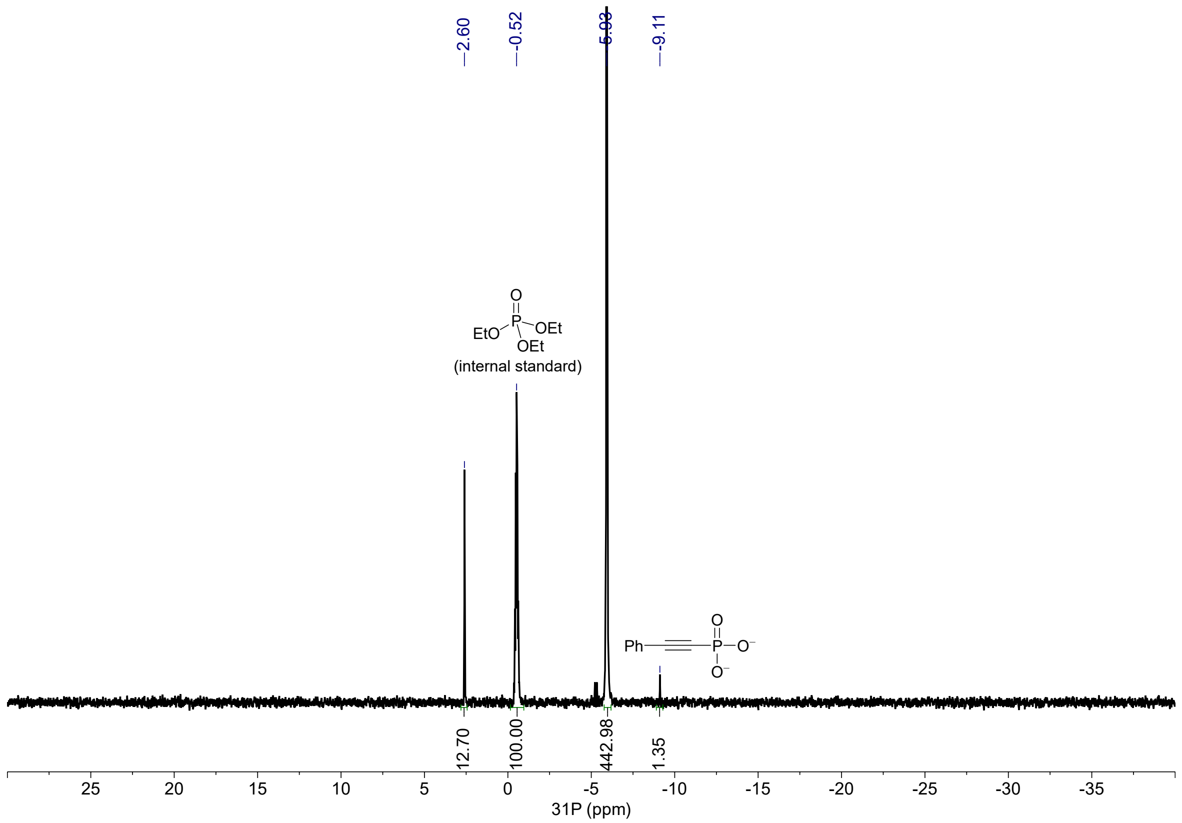

Figure S7: ^{31}P NMR spectrum of the mixture resulting from reaction of $Na_4P_2O_7$ and $KC{\equiv}CC_6H_5$ after 2 h (Table S2 entry 1, 399 mg of crude solid with 107 mg of $PO(OEt)_3$ added).

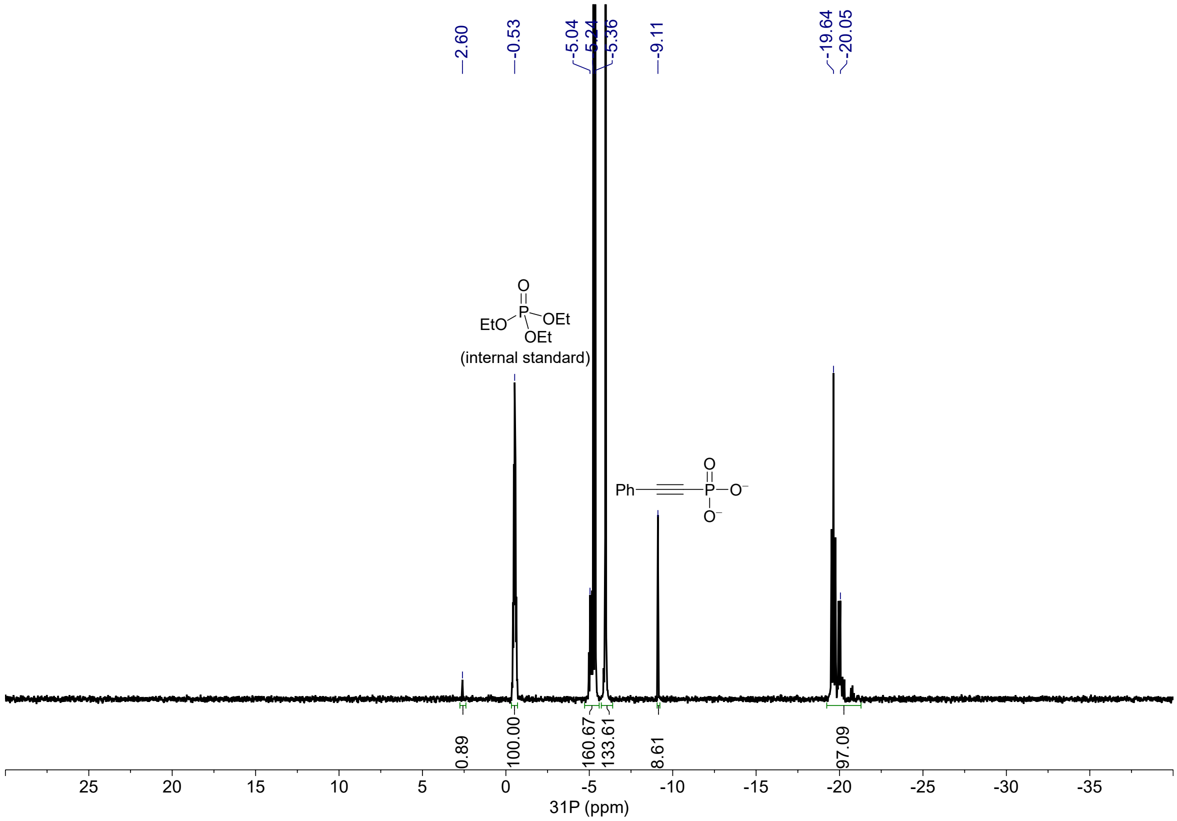

Figure S8: ^{31}P NMR spectrum of the mixture resulting from reaction of $Na_5P_3O_{10}$ and $KC{\equiv}CC_6H_5$ after 4 h (Table S2 entry 6, 436 mg of crude solid with 91 mg of $PO(OEt)_3$ added).

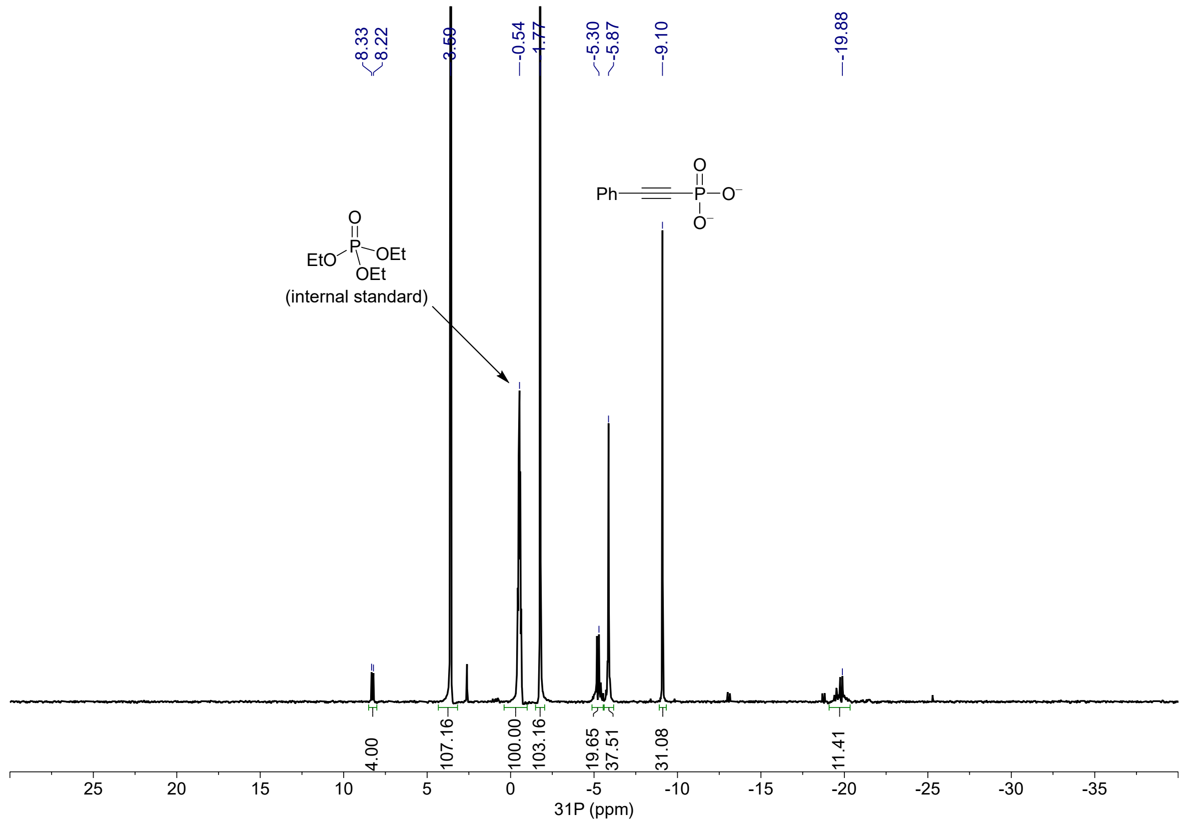

Figure S9: ^{31}P NMR spectrum of the mixture resulting from reaction of Na_2PO_3F and $KC{\equiv}CC_6H_5$ after 2 h (Table S2 entry 10, 470 mg of crude solid with 135 mg of $PO(OEt)_3$ added).

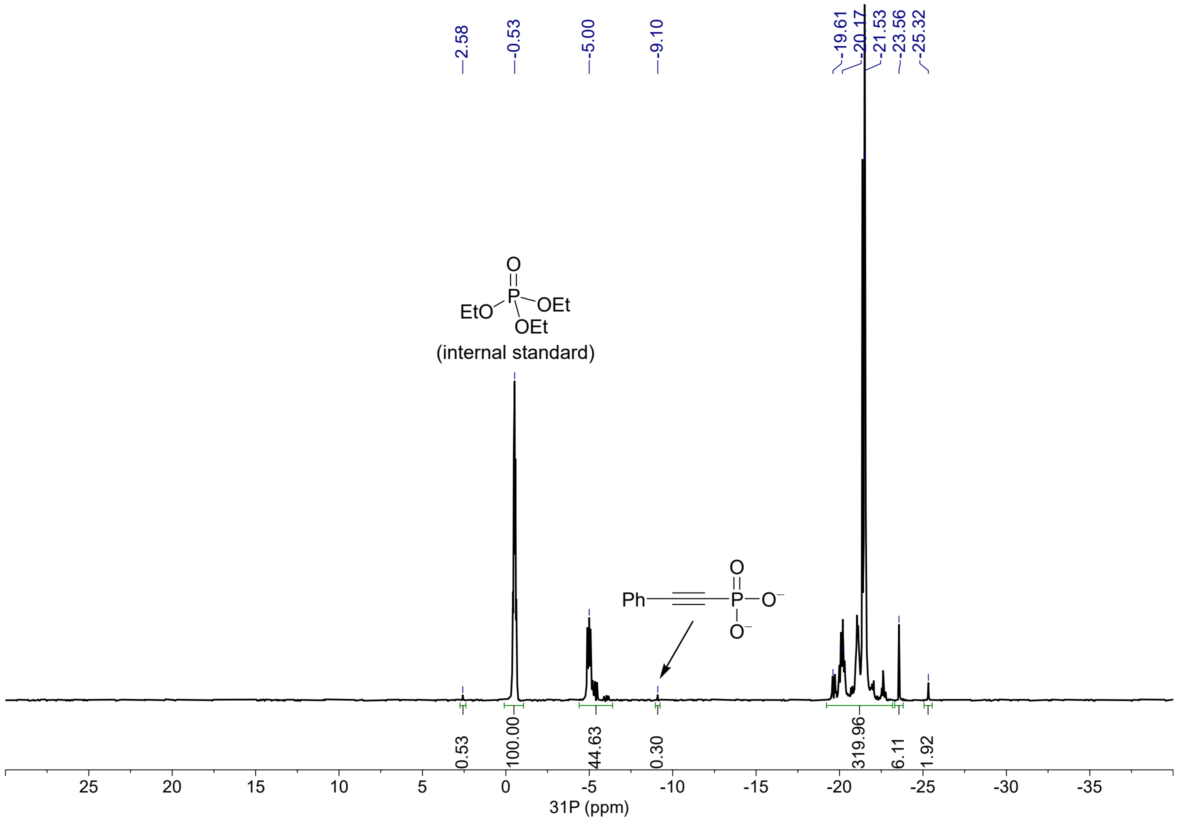

Figure S10: ^{31}P NMR spectrum of the mixture resulting from reaction of $Na_3P_3O_9$ and $KC{\equiv}CC_6H_5$ after 2 h (Table S2 entry 12, 429 mg of crude solid with 168 mg of $PO(OEt)_3$ added).

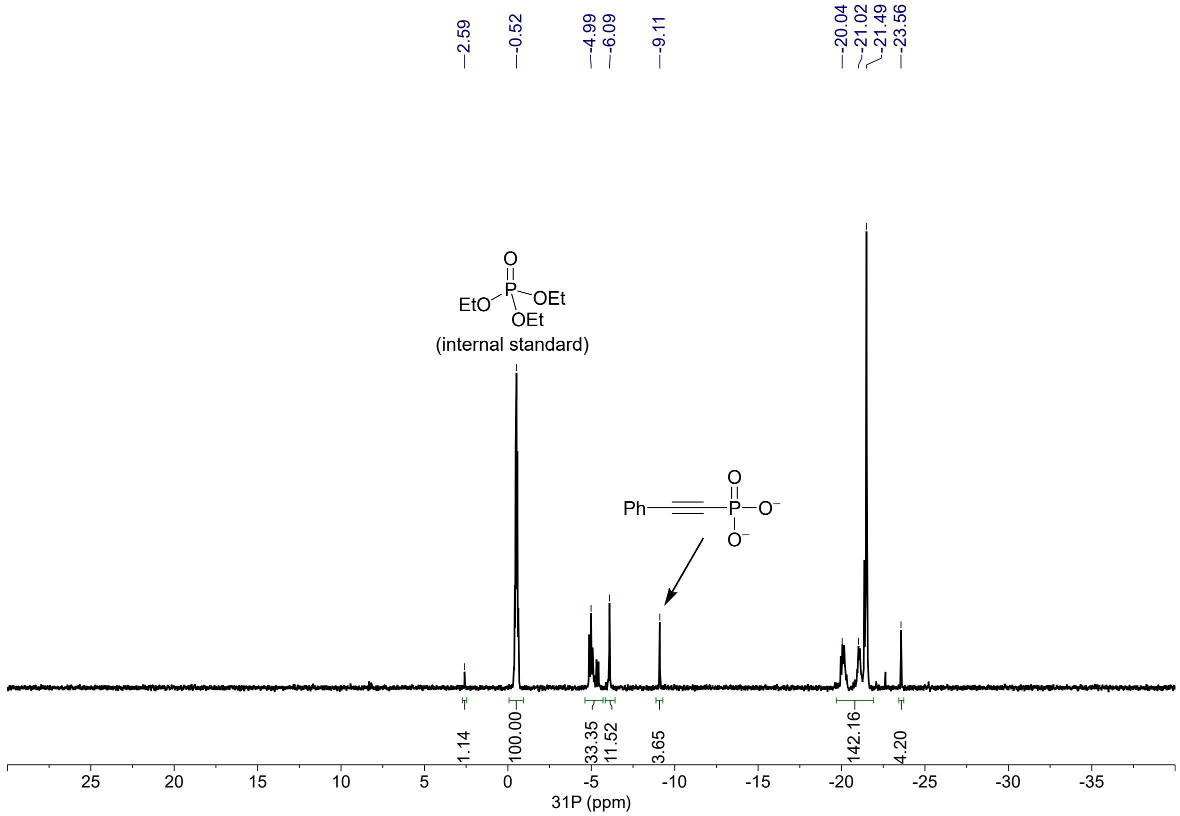

Figure S11: ^{31}P NMR spectrum of the mixture resulting from reaction of Graham's salt and KC≡CC_6H_5 after 2 h (Table S2 entry 13, 310 mg of crude solid with 172 mg of $PO(OEt)_3$ added).

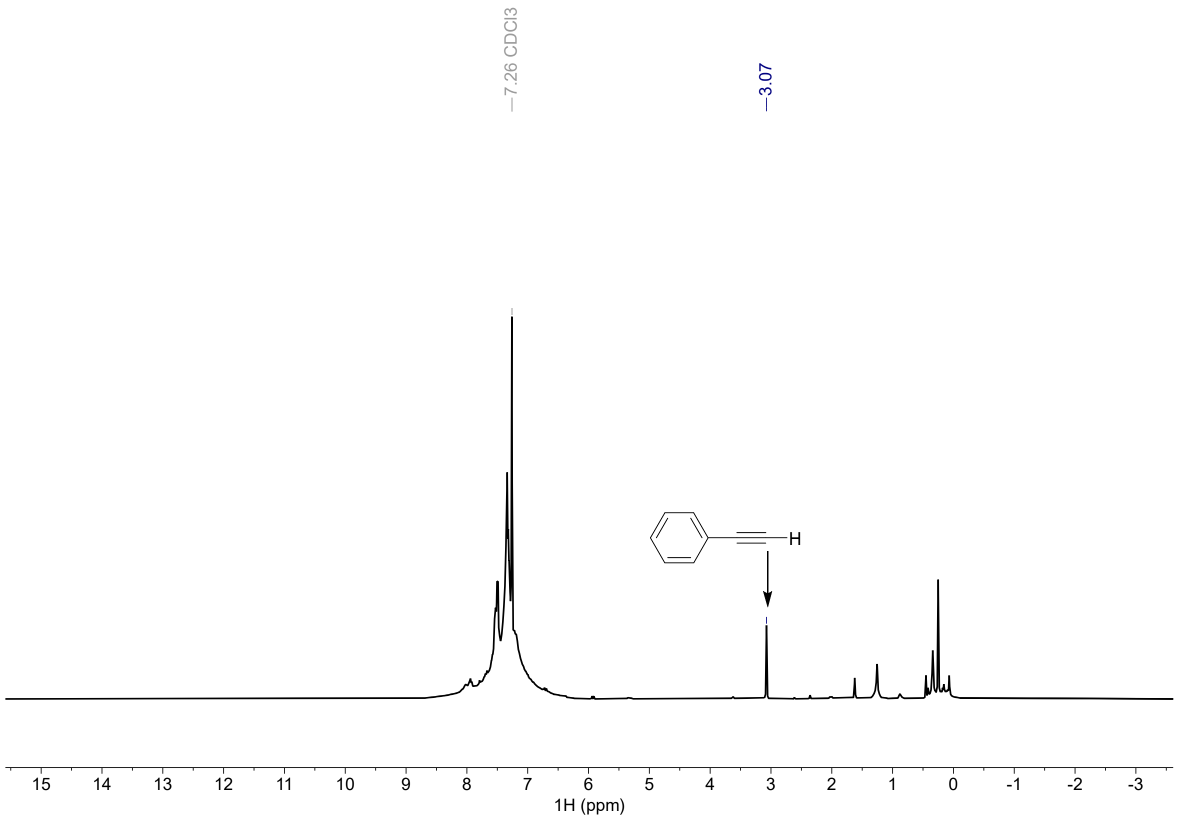

Figure S12: ^{1}H NMR spectrum of the solid isolated from the DCM extract of a quenched reaction mixture of $Na_5P_3O_{10}$ and $KC{\equiv}CC_6H_5$ after 4 h (Table S2 entry 4).

S3 Synthesis of alkynylphosphonic acids from acetylides

S3.1 Preparation of acetylides

In the glovebox, the alkyne (22 mmol, 1.1 equiv) was measured and dissolved in toluene (40 mL) in a 200 mL round bottom flask. The flask was cooled in a bath of liquid nitrogen. NaHMDS (0.6 M in toluene, 33 mL, 20 mmol, 1.0 equiv) or KHMDS (0.7 M in toluene, 28 mL, 20 mmol, 1.0 equiv) was added to the flask with vigorous stirring, resulting in a slurry. Stirring was continued for 1.5 h while the flask was allowed to warm to 25 °C. The precipitate was collected on a 60 mL medium frit by filtration. The collected solid was washed with toluene (2×15 mL) then hexanes (2×15 mL), and dried under reduced pressure to yield the desired acetylide as a fine powder. The acetylides thus obtained were stored at –35 °C in the glovebox and used without further purification.

S3.2 General procedure

Note: The results of mechanochemical reactions are heavily affected by actual milling conditions, including but not limited to grinding time, grinding speed or frequency, material and shape of jars and balls, and reaction scale. The reaction time and speed used herein may not be optimal for milling conducted under other conditions. Reoptimization of reaction conditions may be necessary and is recommended for reactions conducted using a different setup.

In the glovebox, a 125 mL stainless steel milling jar was charged with $Na_5P_3O_{10}$ (5.52 g, 15 mmol, 3.0 equiv), the acetylide (5 mmol, 1.0 equiv), and 30 grinding balls. The milling jar was sealed with the safety closure device, removed from the glovebox, and mounted onto the planetary ball mill. Milling was first conducted at a rotational speed of 200 rpm

for 10 min to mix up the starting materials, and then conducted at 450 rpm for the desired amount of time. After cooling to ambient temperature, the jar was opened in air and the milled material was dissolved in deionized ice water (200 mL). *Note: Residual acetylides react with water to generate heat and may release flammable or hazardous gas. Hence, this addition of ice water should be performed in a well-ventilated area and be slow at the beginning.* Care was taken to dissolve all solid material in the jar and on the lid. The obtained solution was transferred to a 500 mL flask equipped with a stir bar.

$Ca(OH)_2$ (5.0 g, 70 mmol) was added in one portion with vigorous stirring, resulting in precipitation. Stirring was continued for at least 2 h. An aliquot of the supernatant was analyzed by ^{31}P NMR spectroscopy, showing no remaining orthophosphate signal (a singlet at ca. 5 ppm). If orthophosphate or pyrophosphate (a singlet at ca. –7 ppm) signals are still present, another portion of $Ca(OH)_2$ (0.25 g) should be added to the suspension followed by stirring for another 1 h. This process should be repeated until no orthophosphate signal is present in ^{31}P NMR spectra. The suspension was filtered through a plug of Celite (centrifuging this suspension before filtration may aid this process, as this filtration often proves to be difficult). The collected precipitate was transferred back to the flask and suspended in another 80 mL of deionized water. The suspension was stirred for 30 min and the supernatant was checked by ^{31}P NMR spectroscopy again for orthophosphate. The suspension was filtered through a plug of Celite and the combined filtrate was concentrated on a rotovap at 50 °C to ca. 25 mL. An ion-exchange column containing 100 mL of Amberchrom (formerly DOWEX) 50WX8 (H^+) resin that had been washed with deionzied water to neutral pH was prepared. The obtained solution was loaded onto the column and eluted with deionzied water. Only pH$\leq$4 (measured by pH strips) eluents were collected. The collected solution was stirred over activated carbon (ca. 0.5 g) for 1 h, then the activated carbon was filtered off. The resulting solution was

lyophilized to yield the desired phosphonic acid as a white to off-white powder.

S3.3 Synthesis of alkynylphosphonic acids 1a–j

Table S4: Synthesis of alkynylphosphonic acids **1a**–**j** from acetylides[a]

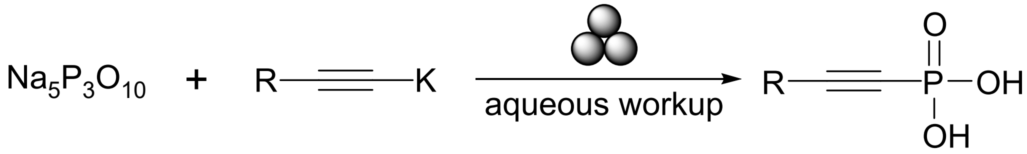

Product	R	reaction time (h)	isolated yield (%)
**1a**	methyl	4	31
**1b**	^{n}butyl	4	32
**1c**	^{t}butyl	12	24
**1d**	cyclopropyl	4	28
**1e**	1-cyclohexenyl	8	27
**1f**	$-(CH_2)_4-$	12	14
**1g**	Na[b]	4	11
**1h**	phenyl	4	31
**1i**	4-fluorophenyl	4	18
**1j**	4-bromophenyl	4	18
**1k**	$(EtO)_2CH-$	4	13(20)[c]
**1l**	THP–O–CH_2–[d]	4	8(9)[c]

[a] Conditions: $Na_5P_3O_{10}$ (15 mmol), KC≡CR (5 mmol), 450 rpm, yields were calculated with KC≡CR as the limiting reagent. [b] Na_2C_2 was used. [c] NMR yield, yield of the hydration product in parenthesis. [d] THP: tetrahydropyranyl.

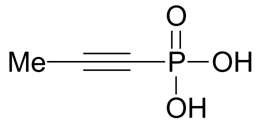

S3.3.1 (Prop-1-yn-1-yl)phosphonic acid 1a

Following the general procedure, ball milling $Na_5P_3O_{10}$ (5.52 g, 15 mmol, 3.0 equiv) and prop-1-yn-1-yl potassium (391 mg, 5.0 mmol, 1.0 equiv) for 4 h yielded the title compound **1a** as a white solid (186 mg, 1.55 mmol, 31%).

^{1}H NMR (500 MHz, D_2O, Figure S13) δ 1.86 (d, J = 3.7 Hz, 3H) ppm. ^{13}C NMR (126 MHz, D_2O, Figure S14) δ 92.06 (d, J = 46.8 Hz), 77.49 (d, J = 255.5 Hz), 3.07 (d, J = 4.0 Hz) ppm. ^{31}P NMR (203 MHz, D_2O, Figure S15) δ –9.56 (br) ppm. ESI-MS(–) (m/z): $[M – H]^-$ calcd for $C_3H_5O_3P$, 118.9904; found, 118.9911.

S3.3.2 (Hex-1-yn-1-yl)phosphonic acid 1b

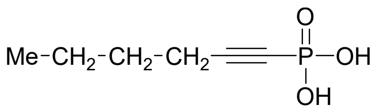

Following the general procedure, ball milling $Na_5P_3O_{10}$ (5.52 g, 15 mmol, 3.0 equiv) and hex-1-yn-1-yl potassium (600 mg, 5.0 mmol, 1.0 equiv) for 4 h yielded the title compound **1b** as an off-white solid (258 mg, 1.59 mmol, 32%).

^{1}H NMR (500 MHz, D_2O, Figure S16) δ 2.20 (td, J = 7.1, 3.6 Hz, 2H), 1.51 – 1.43 (m, 2H), 1.41 – 1.33 (m, 2H), 0.86 (t, J = 7.3 Hz, 1H) ppm. ^{13}C NMR (126 MHz, D_2O, Figure S17) δ 94.14 (d, J = 42.3 Hz), 80.55 (d, J = 239.4 Hz), 29.82 (d, J = 2.0 Hz), 21.41, 17.98 (d, J = 3.7 Hz), 12.85 ppm. ^{31}P NMR (203 MHz, D_2O, Figure S18) δ –8.86 (t, J = 3.7 Hz) ppm. ESI-MS(–) (m/z): $[M – H]^-$ calcd for $C_6H_{11}O_3P$, 161.0373; found, 161.0385.

S3.3.3 (3,3-Dimethylbut-1-yn-1-yl)phosphonic acid 1c

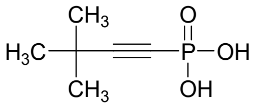

Following the general procedure, ball milling $Na_5P_3O_{10}$ (5.52 g, 15 mmol, 3.0 equiv) and 3,3-dimethylbut-1-yn-1-yl potassium (600 mg, 5.0 mmol, 1.0 equiv) for 12 h yielded the title compound **1c** as a white solid (193 mg, 1.19 mmol, 24%).

^{1}H NMR (500 MHz, D_2O, Figure S19) δ 1.16 (s) ppm. ^{13}C NMR (126 MHz, D_2O, Figure S20) δ 102.04 (d, J = 40.5 Hz), 78.75 (d, J = 237.6 Hz), 29.82, 26.94 (d, J = 3.3 Hz) ppm. ^{31}P NMR (203 MHz, D_2O, Figure S21) δ –8.68 (s) ppm. ESI-MS(–) (m/z): $[M - H]^-$ calcd for $C_6H_{11}O_3P$, 161.0373; found, 161.0395.

S3.3.4 (Cyclopropylethynyl)phosphonic acid 1d

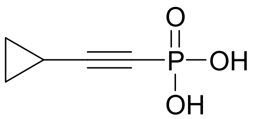

Following the general procedure, ball milling $Na_5P_3O_{10}$ (5.52 g, 15 mmol, 3.0 equiv) and cyclopropylethynyl potassium (520 mg, 5.0 mmol, 1.0 equiv) for 4 h yielded the title compound **1d** as an off-white solid (204 mg, 1.40 mmol, 28%).

^{1}H NMR (500 MHz, D_2O, Figure S22) δ 1.21 (ttd, J = 8.0, 4.8, 2.6 Hz, 1H), 0.71 (dt, J = 8.2, 3.2 Hz, 2H), 0.64 (dt, J = 5.5, 3.2 Hz, 2H) ppm. ^{13}C NMR (126 MHz, D_2O, Figure S23) δ 96.75 (d, J = 43.3 Hz), 75.64 (d, J = 240.0 Hz), 7.74 (d, J = 1.1 Hz), -1.12 (d, J = 4.6 Hz) ppm. ^{31}P NMR (203 MHz, D_2O, Figure S24) δ –8.93 (d, J = 3.1 Hz) ppm. ESI-MS(–) (m/z): $[M - H]^-$ calcd for $C_5H_7O_3P$, 145.0060; found, 145.0069.

S3.3.5 (Cyclohex-1-en-1-ylethynyl)phosphonic acid 1e

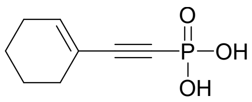

Following the general procedure, ball milling $Na_5P_3O_{10}$ (5.52 g, 15 mmol, 3.0 equiv) and cyclohex-1-en-1-ylethynyl potassium (720 mg, 5.0 mmol, 1.0 equiv) for 8 h yielded the title compound **1e** as an off-white solid (252 mg, 1.35 mmol, 27%).

^{1}H NMR (500 MHz, D_2O, Figure S25) δ 6.21 (t, J = 4.2 Hz, 1H), 2.10 – 2.01 (m, 4H), 1.57 (tt, J = 6.3, 5.8 Hz, 2H), 1.51 (tt, J = 5.8, 5.2 Hz, 2H) ppm. ^{13}C NMR (126 MHz, D_2O, Figure S26) δ 137.36 (d, J = 2.8 Hz), 119.90 (d, J = 4.7 Hz), 93.10 (d, J = 41.4 Hz), 86.83 (d, J = 235.4 Hz), 28.30, 25.19, 21.68, 20.85 ppm. ^{31}P NMR (203 MHz, D_2O, Figure S27) δ –8.68 (s) ppm. ESI-MS(–) (m/z): $[M - H]^-$ calcd for $C_8H_{11}O_3P$, 185.0373; found, 185.0350.

S3.3.6 Octa-1,7-diyne-1,8-diylbis(phosphonic acid) 1f

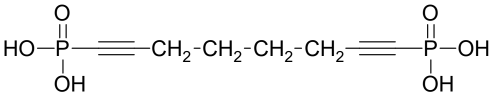

Following the general procedure, ball milling $Na_5P_3O_{10}$ (5.52 g, 15 mmol, 3.0 equiv) and octa-1,7-diyne-1,8-diylbispotassium (455 mg, 2.5 mmol, 1.0 equiv) for 12 h yielded the title compound **1f** as a white solid (96 mg, 0.36 mmol, 14%).

^{1}H NMR (500 MHz, D_2O, Figure S28) δ 2.21 (dp, J = 7.0, 3.4 Hz, 4H), 1.62 – 1.53 (m, 4H) ppm. ^{13}C NMR (126 MHz, D_2O, Figure S29) δ 93.58 (d, J = 42.4 Hz), 80.78 (d, J = 238.8 Hz), 26.85 (d, J = 1.9 Hz), 17.81 (d, J = 3.7 Hz) ppm. ^{31}P NMR (203 MHz, D_2O, Figure S30) δ –8.90 (t, J = 3.7 Hz) ppm. ESI-MS(–) (m/z): $[M - H]^-$ calcd for $C_8H_{12}O_6P_2$, 265.0036; found, 265.0037.

S3.3.7 Ethyne-1,2-diylbis(phosphonic acid) 1g

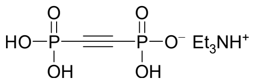

Following the general procedure, ball milling $Na_5P_3O_{10}$ (5.52 g, 15 mmol, 3.0 equiv) and Na_2C_2 (175 mg, 2.5 mmol, 1.0 equiv) for 4 h yielded crude **1g** as an off-white solid, which contains ca. 7% of the monophosphonic acid **2**. Recrystallization of the crude product in a mixture of EtOH (20 mL), Et_2O (20 mL) and NEt_3 (0.5 mL) yielded analytically pure **1g** as the triethylammonium salt (79 mg, 0.28 mmol, 11%).

^{1}H NMR (500 MHz, D_2O, Figure S31) δ 3.18 (q, J = 7.3 Hz, 2H), 1.26 (t, J = 7.3 Hz, 3H) ppm. ^{13}C NMR (126 MHz, D_2O, Figure S32) δ 89.54 (dd, J = 244.4, 37.3 Hz), 46.63, 8.19 ppm. ^{31}P NMR (203 MHz, D_2O, Figure S33) δ –12.46 (s) ppm. ESI-MS(–) (m/z): $[M – H]^-$ calcd for $C_2H_4O_6P_2$, 184.9410; found, 184.9414.

S3.3.8 (Phenylethynyl)phosphonic acid 1h

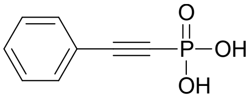

Following the general procedure, ball milling $Na_5P_3O_{10}$ (5.52 g, 15 mmol, 3.0 equiv) and phenylethynyl potassium (700 mg, 5.0 mmol, 1.0 equiv) for 4 h yielded the title compound **1h** as an off-white solid (283 mg, 1.55 mmol, 31%).

^{1}H NMR (500 MHz, D_2O, Figure S34) δ 7.57 – 7.50 (m, 2H), 7.44 – 7.34 (m, 3H) ppm. ^{13}C NMR (126 MHz, D_2O, Figure S35) δ 131.85 (d, J = 2.0 Hz), 128.99, 128.51, 122.01 (d, J = 4.5 Hz), 90.68 (d, J = 41.5 Hz), 89.72 (d, J = 232.9 Hz) ppm. ^{31}P NMR (203 MHz, D_2O, Figure S36) δ –9.00 (s) ppm. ESI-MS(–) (m/z): $[M – H]^-$ calcd for $C_8H_7O_3P$, 181.0060; found, 181.0066.

S3.3.9 ((4-Fluorophenyl)ethynyl)phosphonic acid 1i

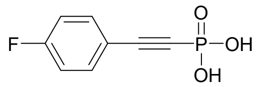

Following the general procedure, ball milling $Na_5P_3O_{10}$ (5.52 g, 15 mmol, 3.0 equiv) and (4-fluorophenyl)ethynyl potassium (790 mg, 5.0 mmol, 1.0 equiv) for 4 h yielded the title compound **1i** as a white solid (182 mg, 0.91 mmol, 18%).

^{1}H NMR (500 MHz, D_2O, Figure S37) δ 7.58 – 7.52 (m, 2H), 7.10 (t, J = 8.9 Hz, 2H) ppm. ^{13}C NMR (126 MHz, D_2O, Figure S38) δ 162.56 (d, J = 247.5 Hz), 133.98 (dd, J = 8.7, 2.2 Hz), 118.20 (t, J = 3.9 Hz), 115.56 (d, J = 22.3 Hz), 89.80 (d, J = 41.8 Hz), 89.28 (d, J = 232.9 Hz) ppm. ^{19}F NMR (471 MHz, D_2O, Figure S38) δ –110.95 (tt, J = 9.7, 5.5 Hz) ppm.^{31}P NMR (203 MHz, D_2O, Figure S40) δ –9.04 (s) ppm. ESI-MS(–) (m/z): $[M - H]^-$ calcd for $C_8H_6FO_3P$, 198.9966; found, 198.9978.

S3.3.10 ((4-Bromophenyl)ethynyl)phosphonic acid 1j

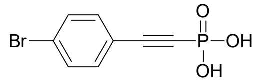

Following the general procedure, ball milling $Na_5P_3O_{10}$ (5.52 g, 15 mmol, 3.0 equiv) and (4-bromophenyl)ethynyl potassium (1.096 g, 5.0 mmol, 1.0 equiv) for 4 h yielded the title compound **1j** as an off-white solid (235 mg, 0.90 mmol, 18%).

^{1}H NMR (500 MHz, D_2O, Figure S41) δ 7.54 (dd, J = 8.4, 1.6 Hz, 2H), 7.45 – 7.39 (m, 2H) ppm. ^{13}C NMR (126 MHz, D_2O, Figure S42) δ 133.42 (d, J = 2.1 Hz), 131.54, 122.54, 121.14 (d, J = 4.5 Hz), 90.76 (d, J = 232.9 Hz) 89.67 (d, J = 41.6 Hz) ppm. ^{31}P NMR (203 MHz, D_2O, Figure S43) δ –9.12 (s) ppm. ESI-MS(–) (m/z): $[M - H]^-$ calcd for $C_8H_6BrO_3P$, 258.9165; found, 258.9195.

S3.3.11 Attempted synthesis of (3,3-diethoxyprop-1-yn-1-yl)phosphonic acid 1k

The ball milling reaction of $Na_5P_3O_{10}$ (5.52 g, 15 mmol, 3.0 equiv) and 3,3-diethoxyprop-1-yn-1-yl potassium (830 mg, 5.0 mmol, 1.0 equiv) was set up according to the general procedure. After cooling to ambient temperature, the jar was brought back into the glovebox and opened. An aliquot (ca. 400 mg, measured accurately) was removed from the jar and transferred into a 20 mL scintillation vial. The vial was brought out of the glovebox and quenched with ice water (ca. 8 mL). $NaHCO_3$ (ca. 300 mg) was added to neutralize the solution. All solid material was dissolved with the help of sonication. A known amount of triethyl phosphate ($PO(OEt)_3$, ca. 100 mg) was added to the solution as an internal standard and dissolved. An aliquot of the resulting solution was transferred to an NMR tube and to which was added D_2O (ca. 0.1 mL). Analysis was performed by ^{31}P NMR spectroscopy, showing 13% of the title compound **1k** and 20% of the hydration product (Figure S44).

S3.3.12 Attempted synthesis of (3-((tetrahydro-2*H*-pyran-2-yl)oxy)prop-1-yn-1-yl)phosphonic acid 1l

The ball milling reaction of $Na_5P_3O_{10}$ (5.52 g, 15 mmol, 3.0 equiv) and 3-((tetrahydro-2*H*-pyran-2-yl)oxy)prop-1-yn-1-yl potassium (891 mg, 5.0 mmol, 1.0 equiv) was set up according to the general procedure. After cooling to ambient temperature, the jar was brought back into the glovebox and opened. An aliquot (ca. 400 mg) was removed from the jar and transferred into a 20 mL scintillation vial. The vial was brought out of the glovebox and quenched with ice water (ca. 8 mL). $NaHCO_3$ (ca. 300 mg) was added to neutralize the solution. All solid material was dissolved with the help of sonication. A

known amount of triethyl phosphate ($PO(OEt)_3$, ca. 100 mg) was added to the solution as an internal standard and dissolved. An aliquot of the resulting solution was transferred to an NMR tube and to which was added D_2O (ca. 0.1 mL). Analysis was performed by ^{31}P NMR spectroscopy, showing 8% of the title compound **1l** and 9% of the hydration product (Figure S45).

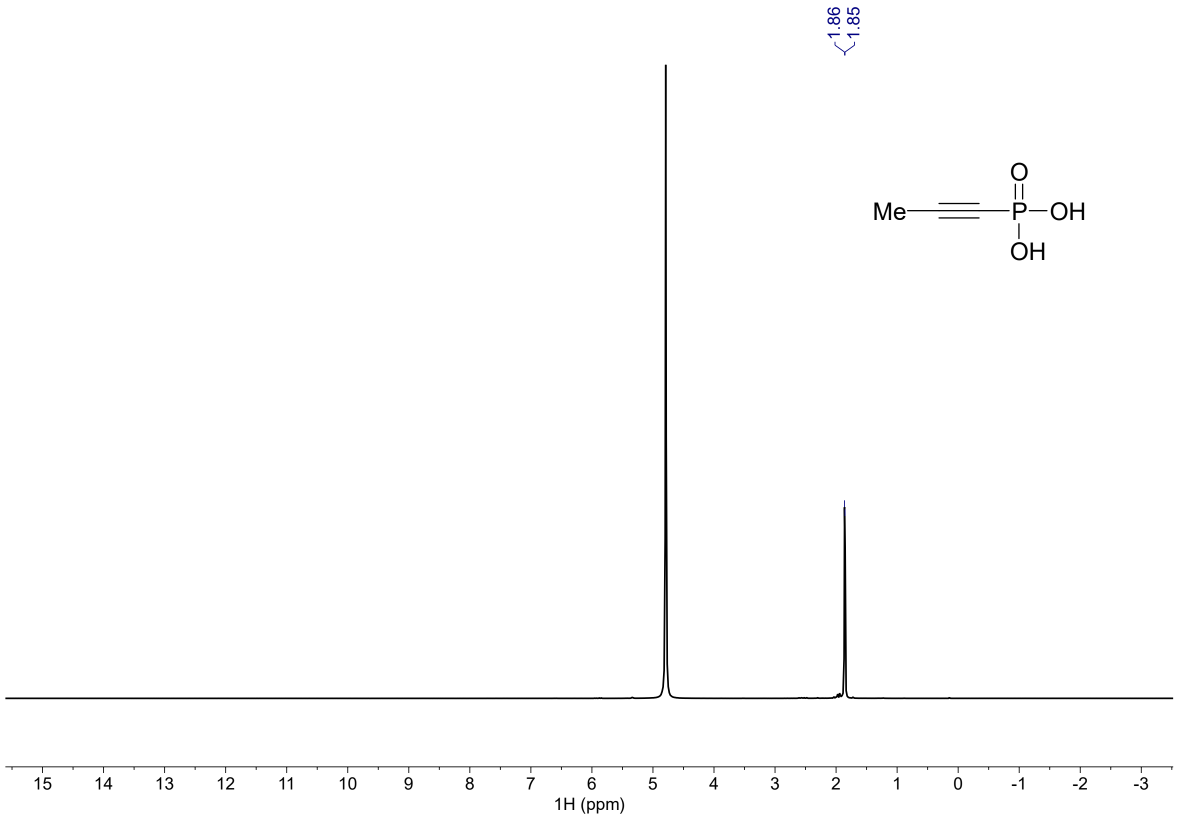

Figure S13: ^{1}H NMR spectrum of **1a** in D_2O at 25 °C, recorded at 500 MHz.

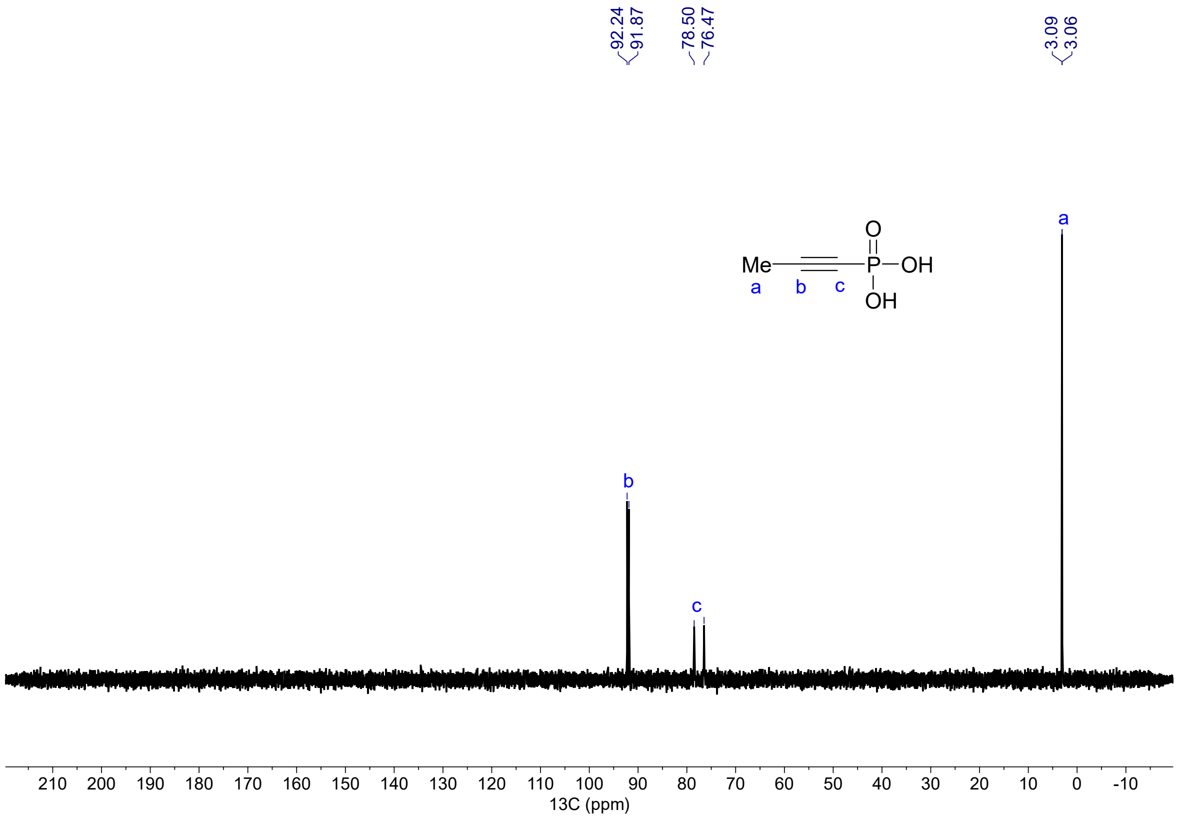

Figure S14: ^{13}C NMR spectrum of **1a** in D_2O at 25 °C, recorded at 126 MHz.

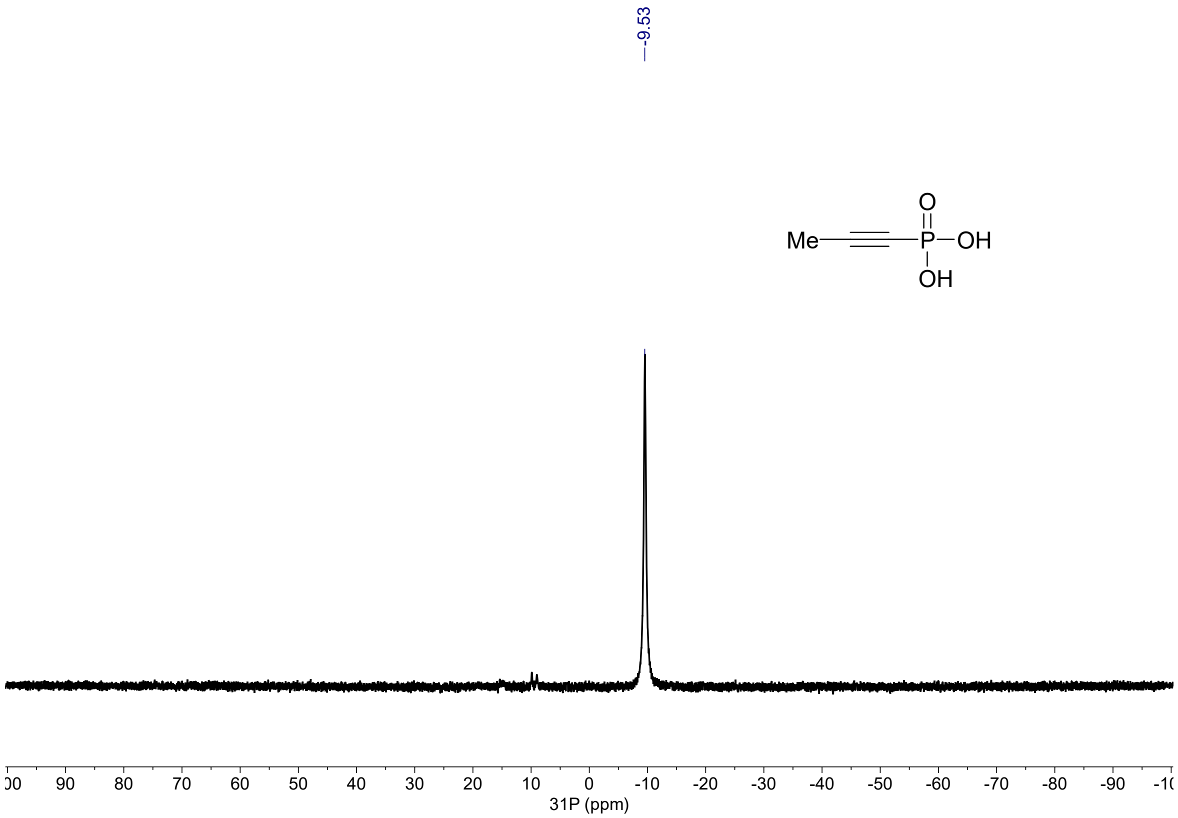

Figure S15: ^{31}P NMR spectrum of **1a** in D_2O at 25 °C, recorded at 203 MHz.

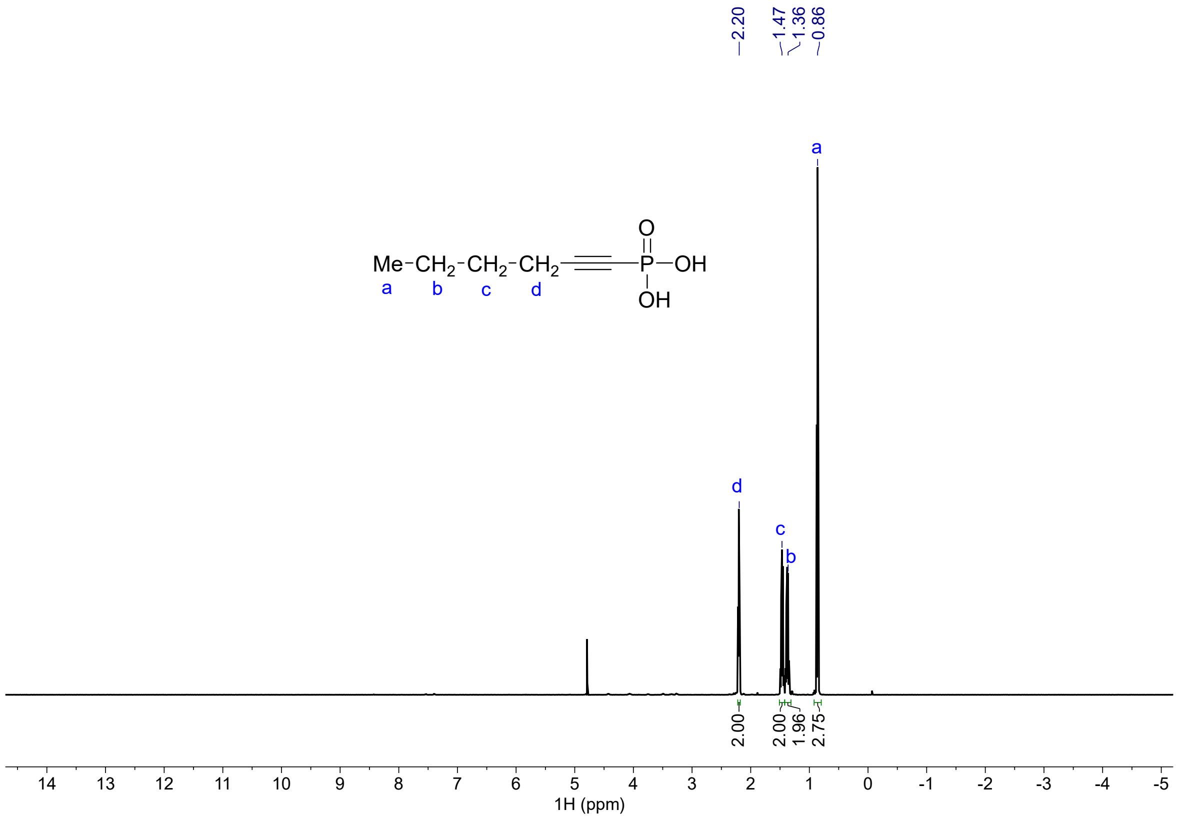

Figure S16: ^{1}H NMR spectrum of **1b** in D_2O at 25 °C, recorded at 500 MHz.

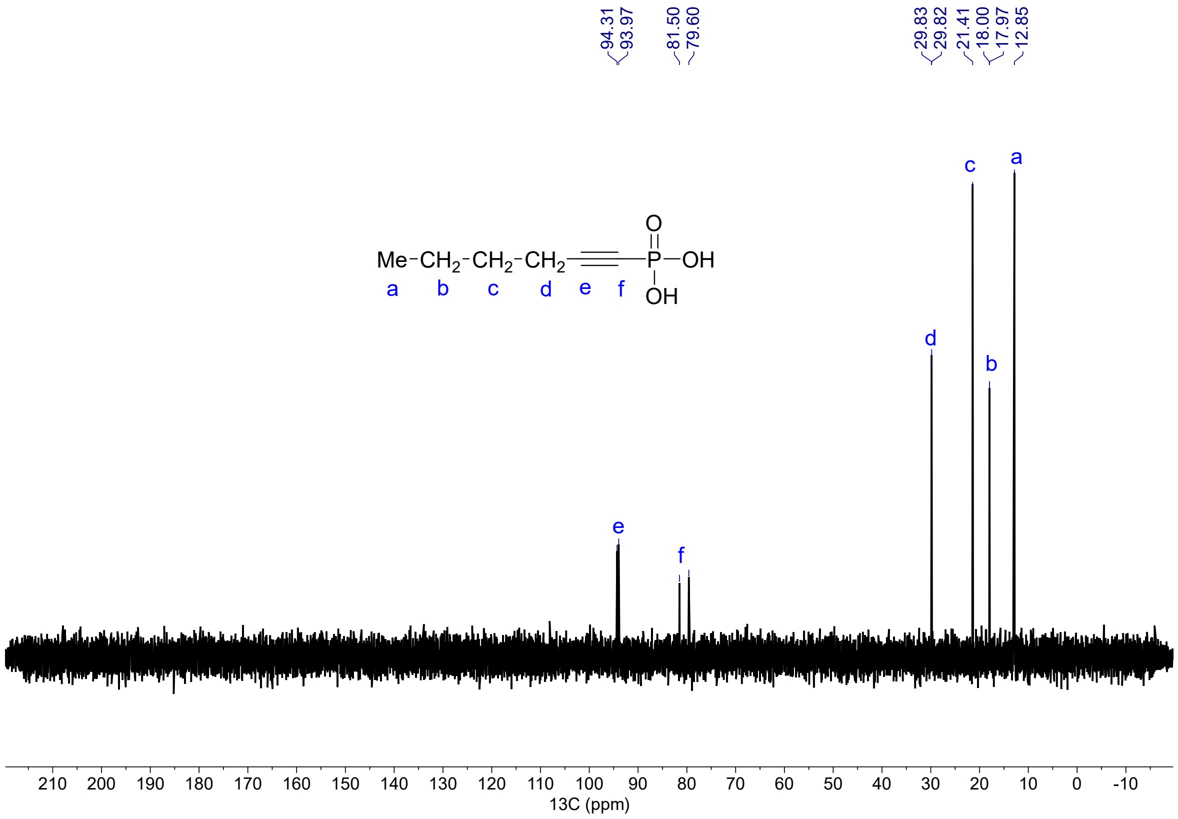

Figure S17: ${}^{13}C$ NMR spectrum of **1b** in D_2O at 25 °C, recorded at 126 MHz.

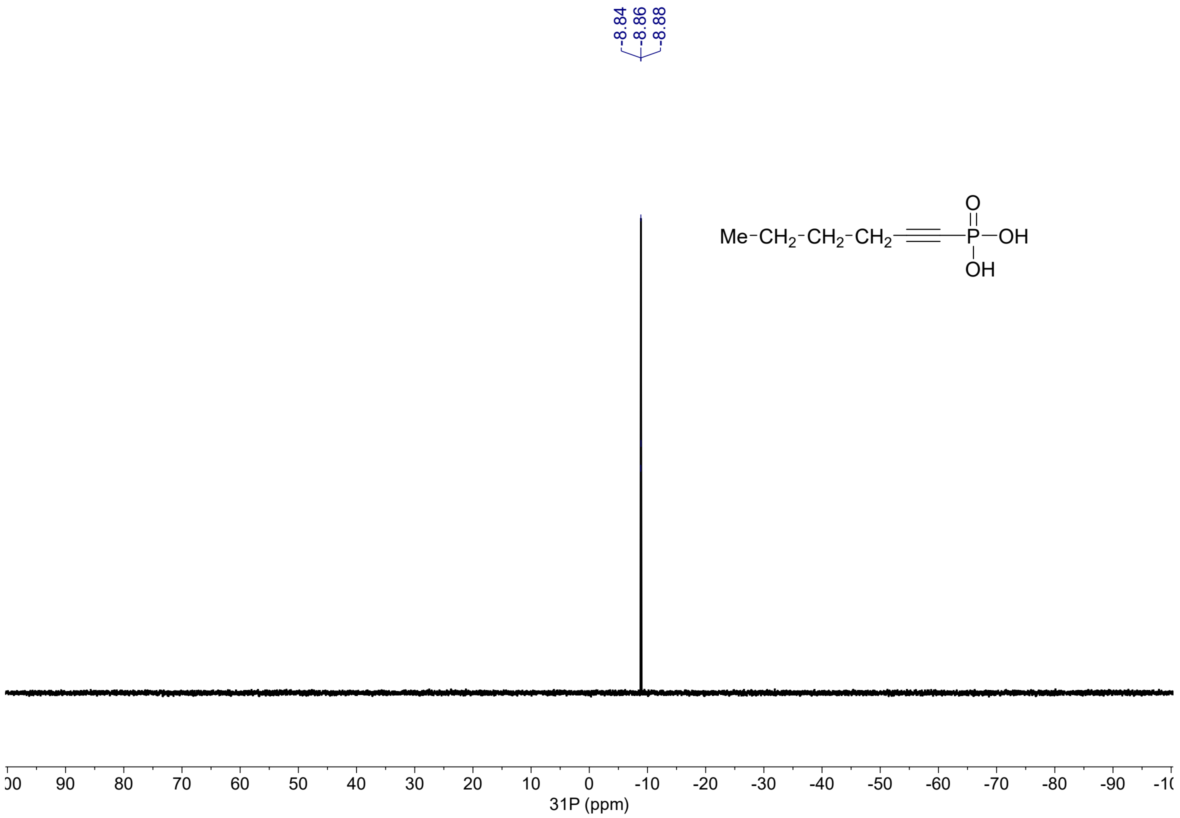

Figure S18: ^{31}P NMR spectrum of **1b** in D_2O at 25 °C, recorded at 203 MHz.

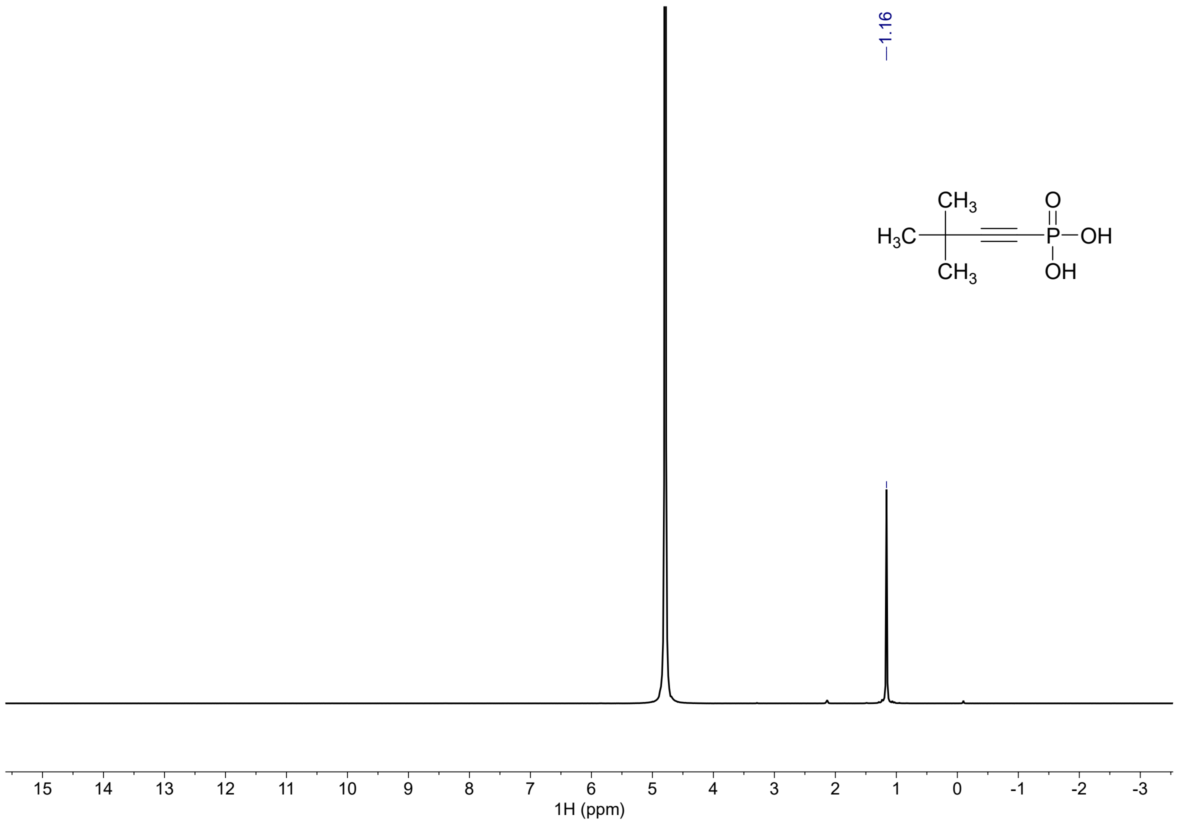

Figure S19: ^{1}H NMR spectrum of **1c** in D_2O at 25 °C, recorded at 500 MHz.

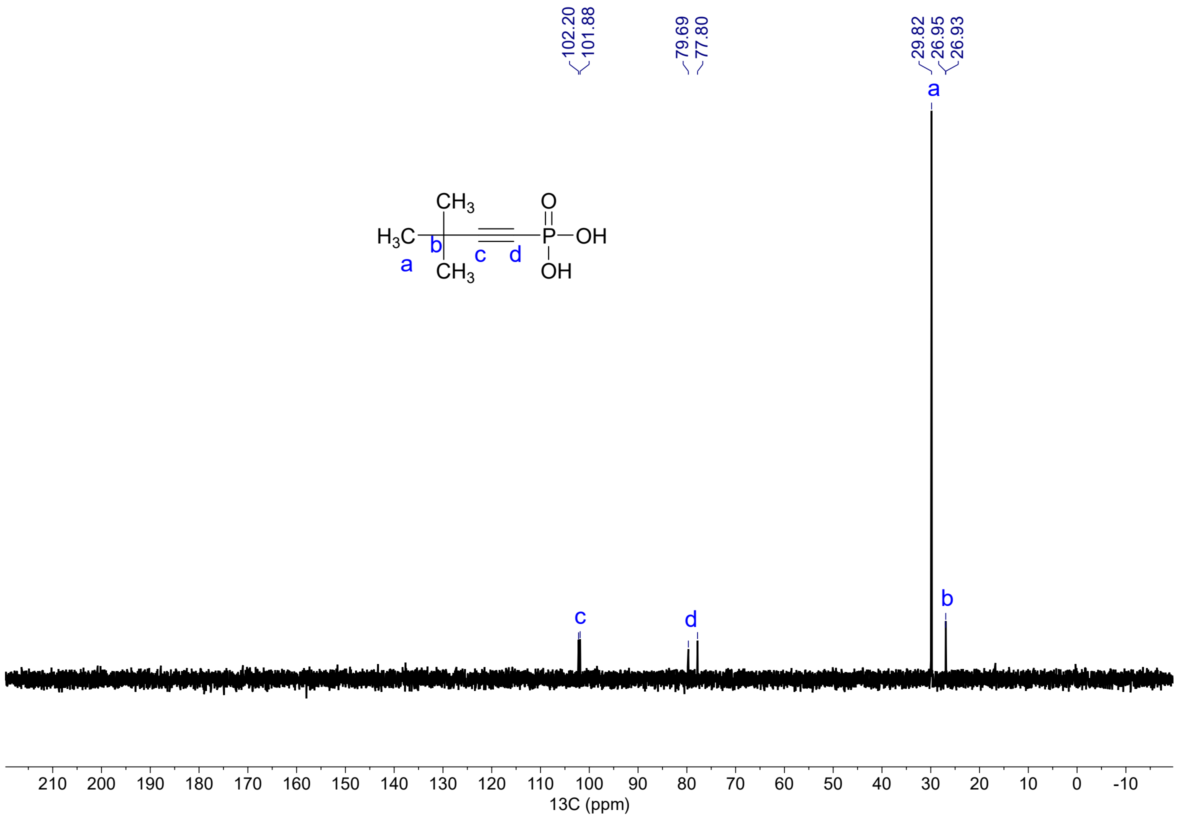

Figure S20: ^{13}C NMR spectrum of **1c** in D_2O at 25 °C, recorded at 126 MHz.

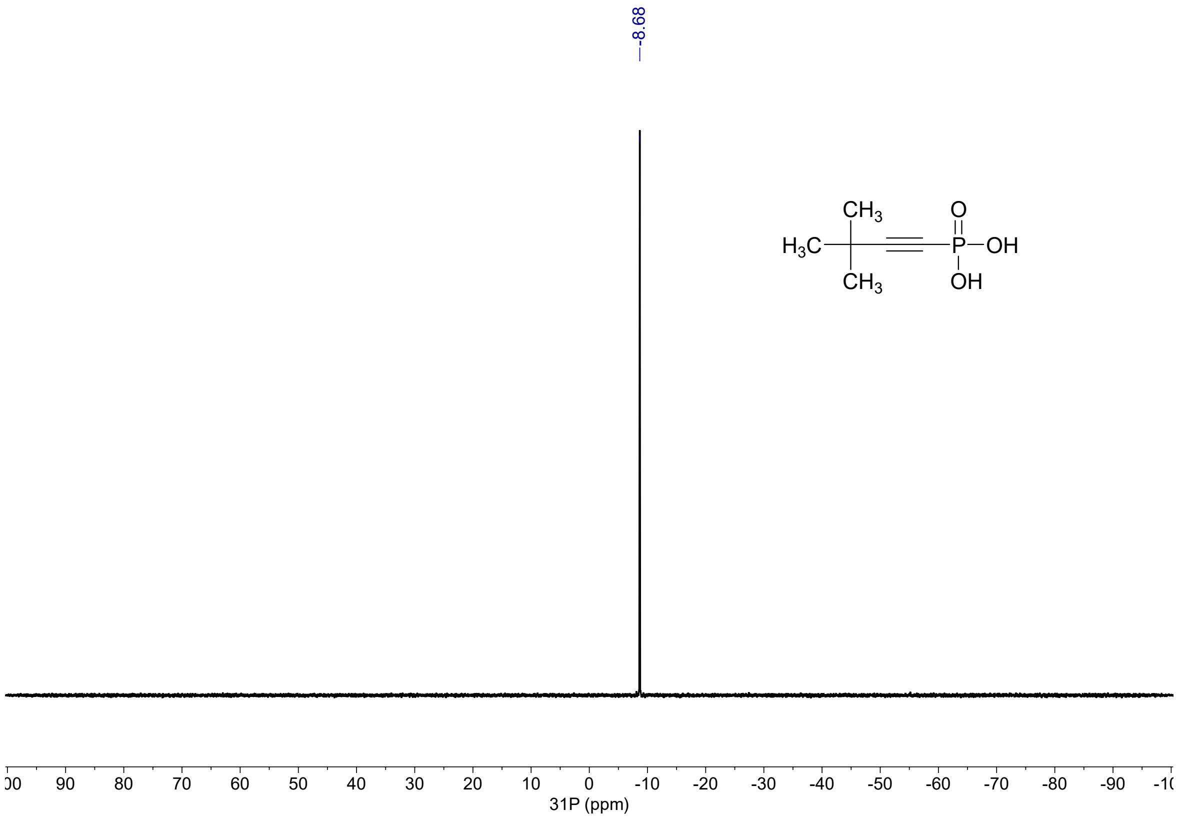

Figure S21: ^{31}P NMR spectrum of **1c** in D_2O at 25 °C, recorded at 203 MHz.

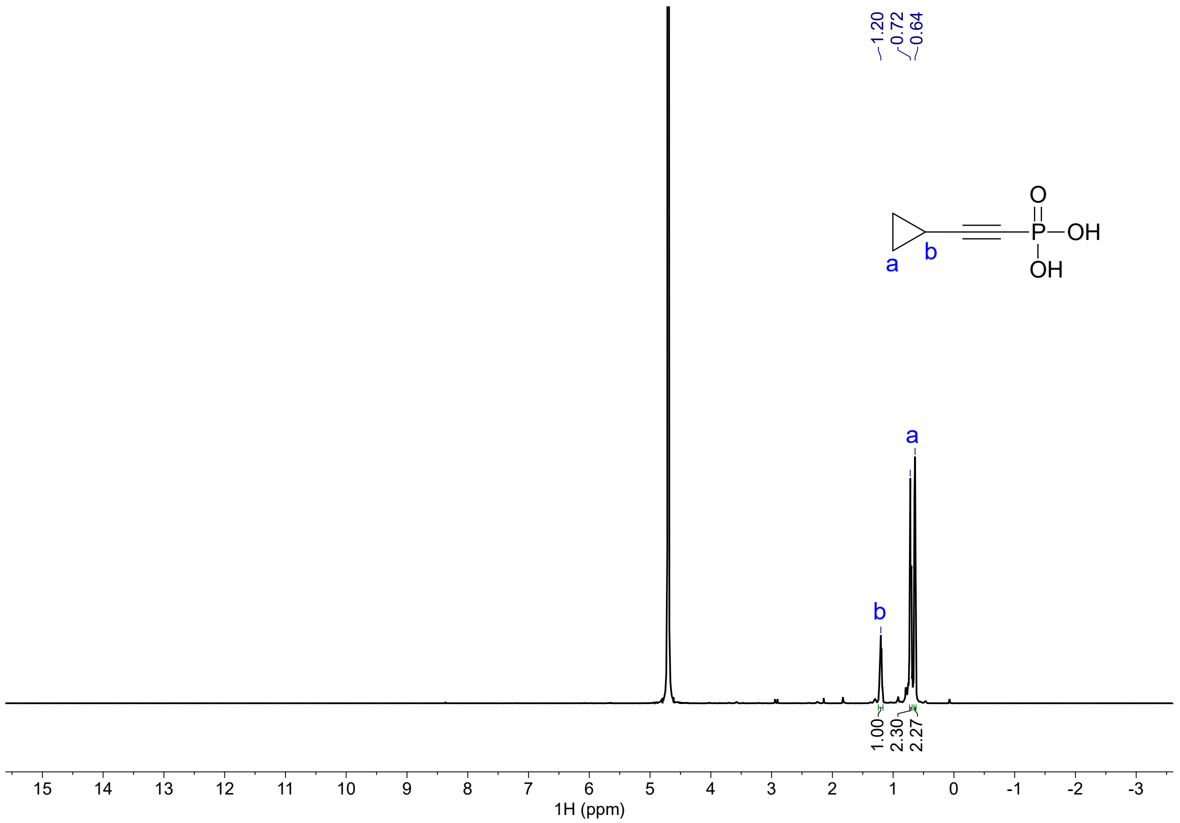

Figure S22: ^{1}H NMR spectrum of **1d** in D_2O at 25 °C, recorded at 500 MHz.

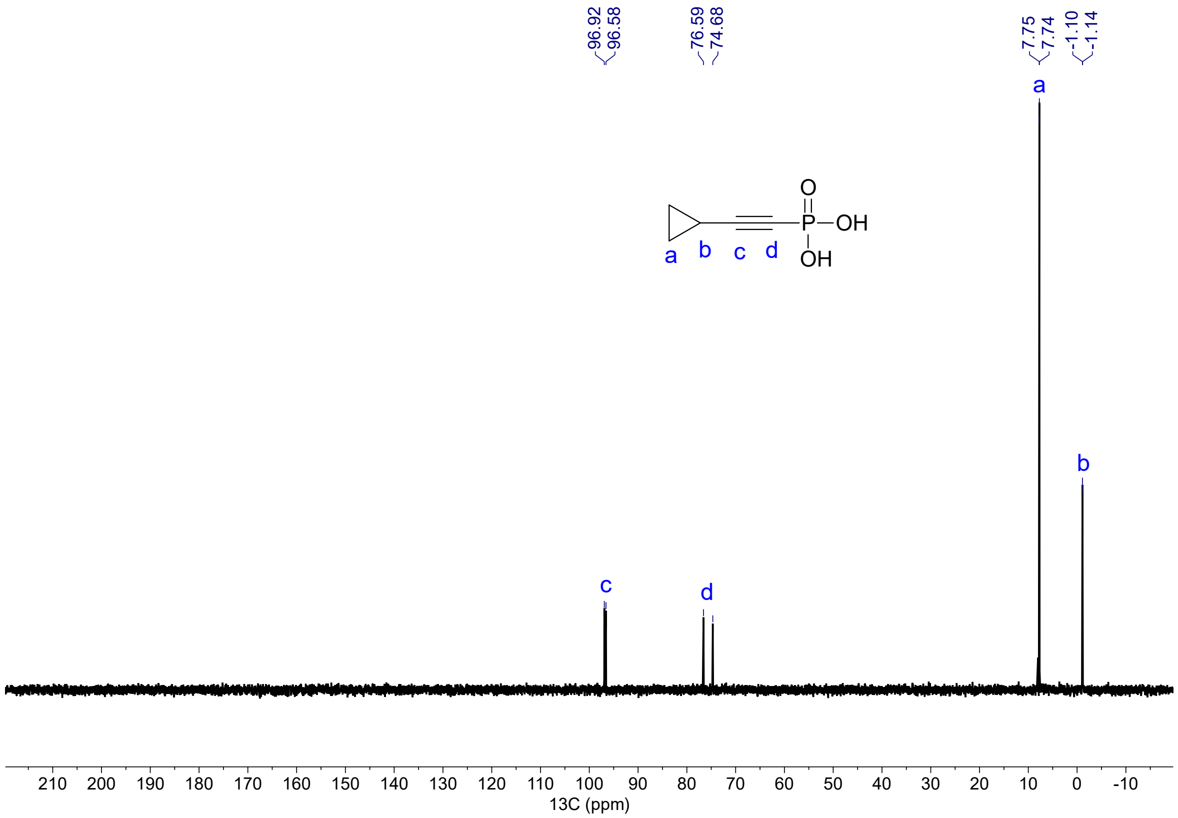

Figure S23: ^{13}C NMR spectrum of **1d** in D_2O at 25 °C, recorded at 126 MHz.

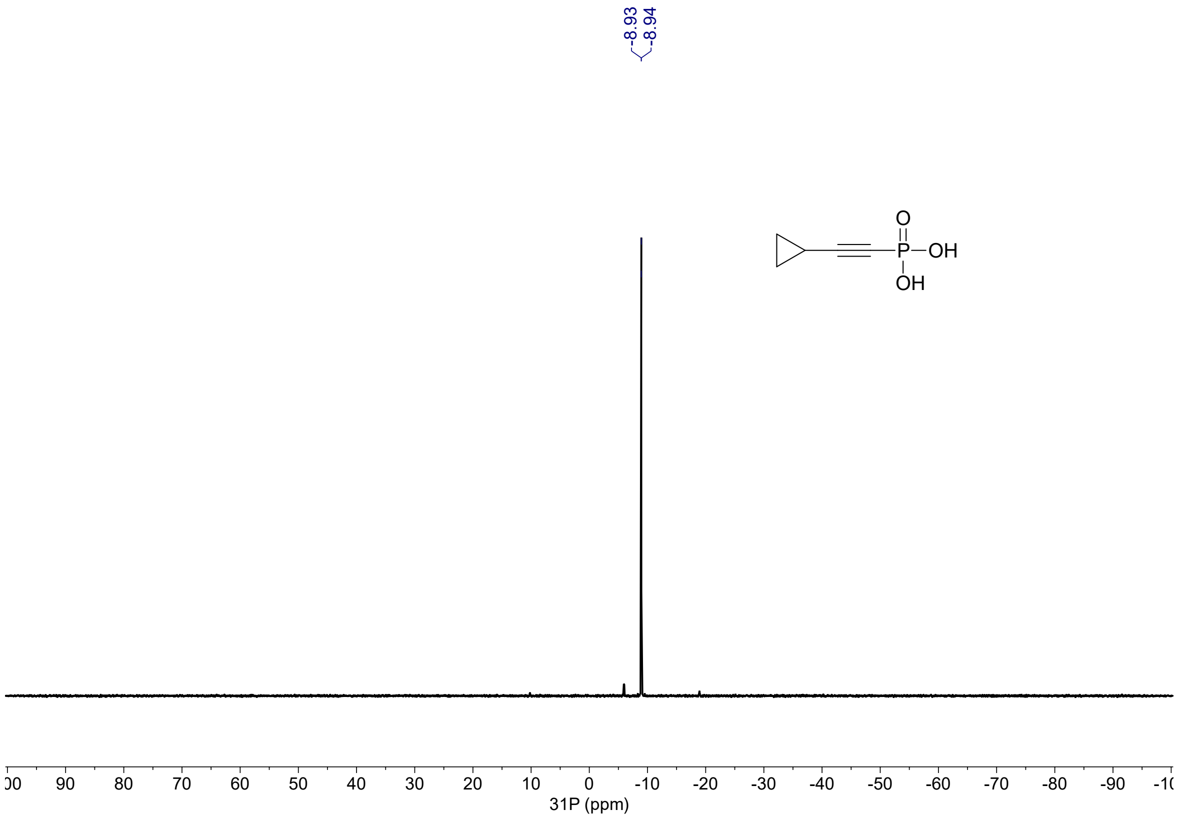

Figure S24: ^{31}P NMR spectrum of **1d** in D_2O at 25 °C, recorded at 203 MHz.

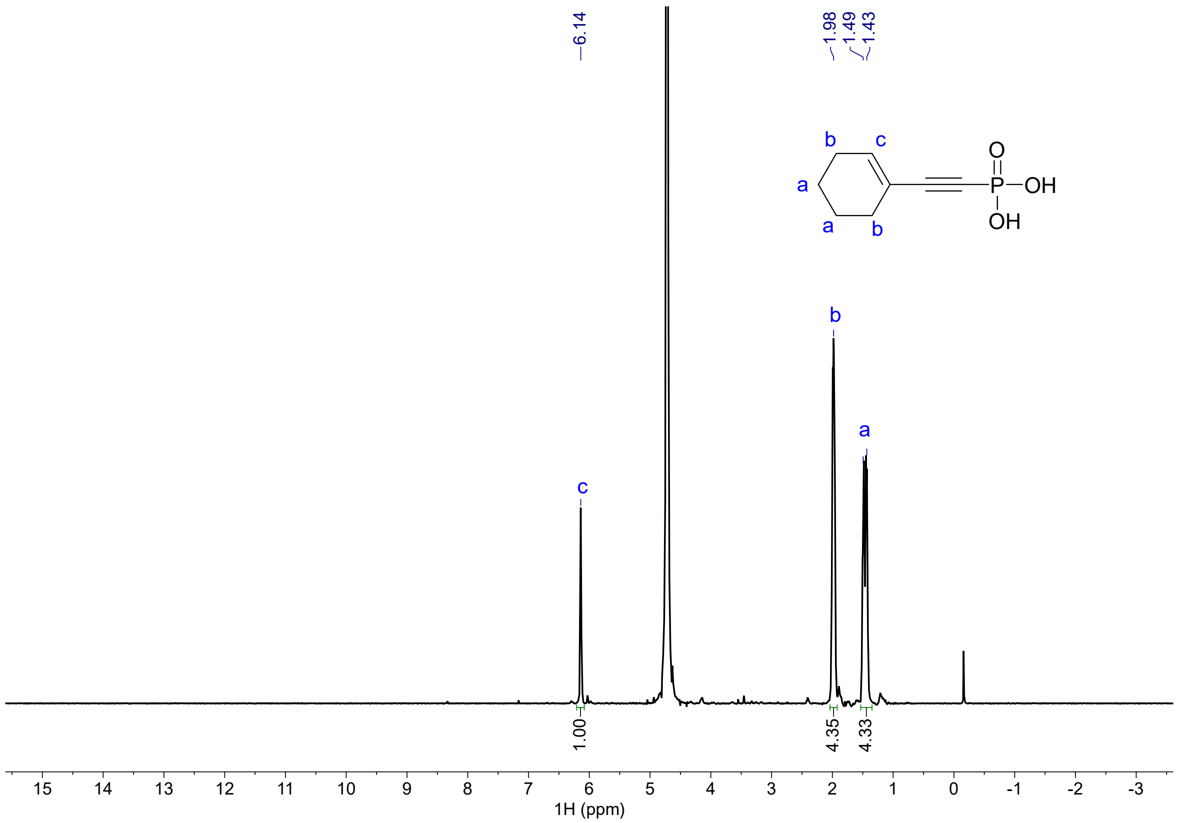

Figure S25: ^{1}H NMR spectrum of **1e** in D_2O at 25 °C, recorded at 500 MHz.

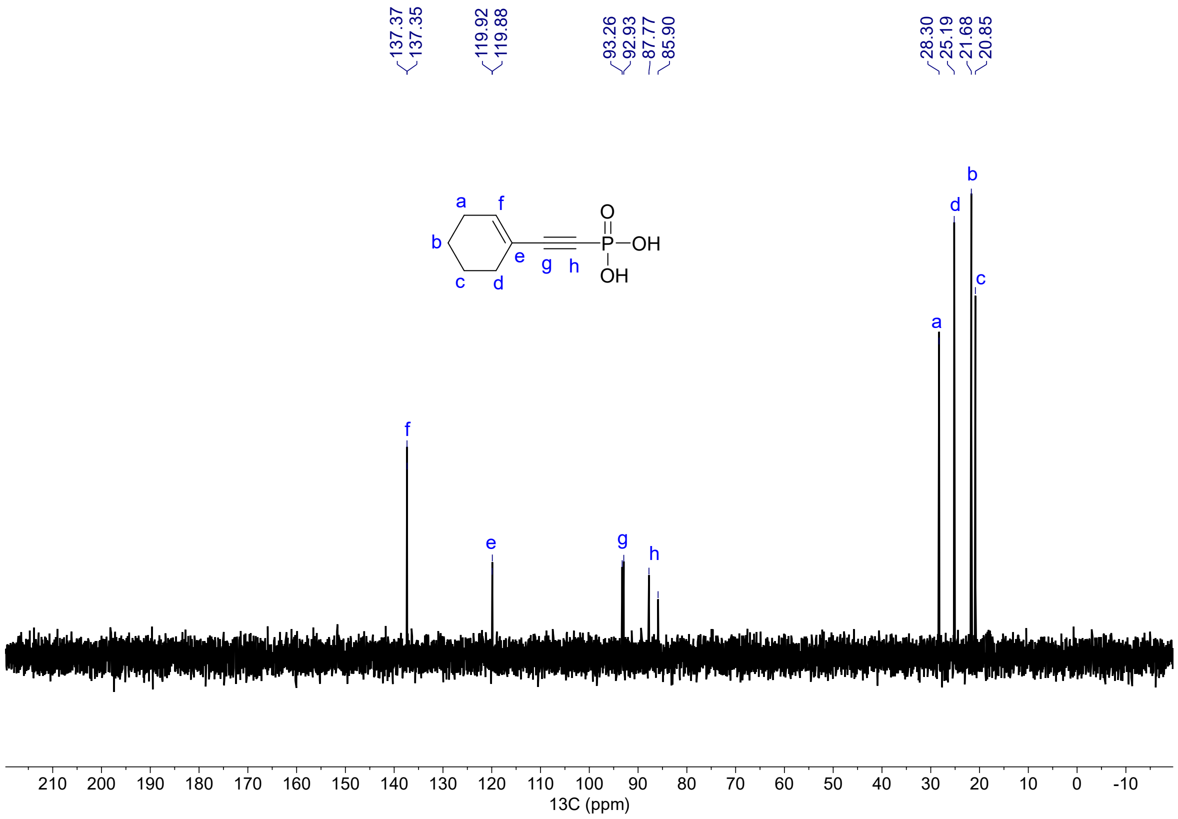

Figure S26: ^{13}C NMR spectrum of **1e** in D_2O at 25 °C, recorded at 126 MHz.

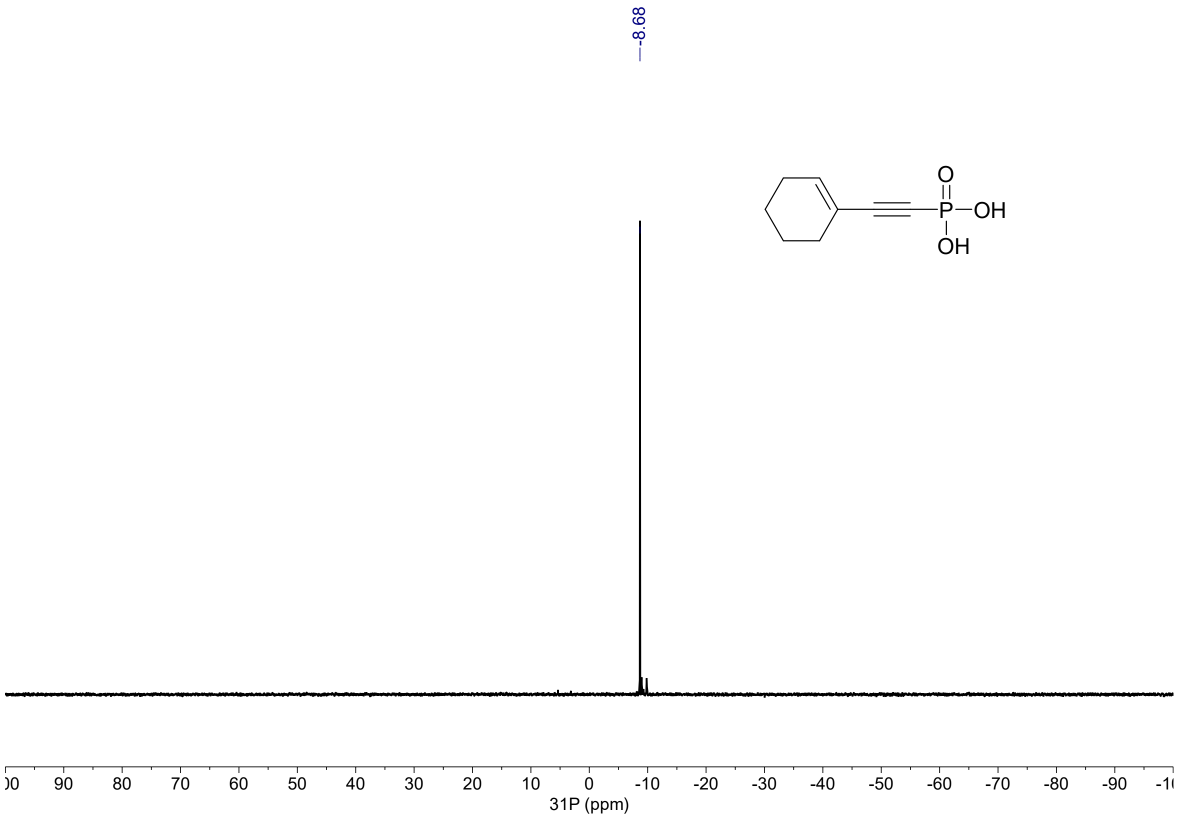

Figure S27: ^{31}P NMR spectrum of **1e** in D_2O at 25 °C, recorded at 203 MHz.

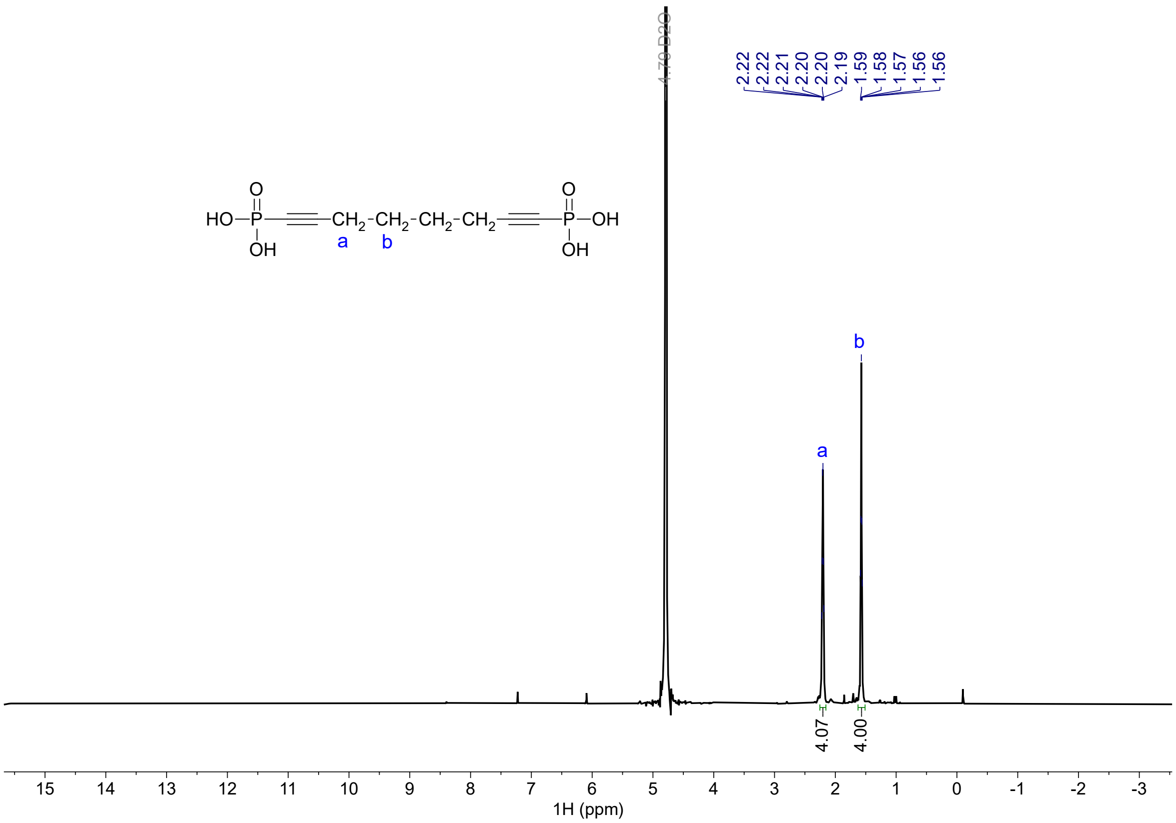

Figure S28: ^{1}H NMR spectrum of **1f** in D_2O at 25 °C, recorded at 500 MHz.

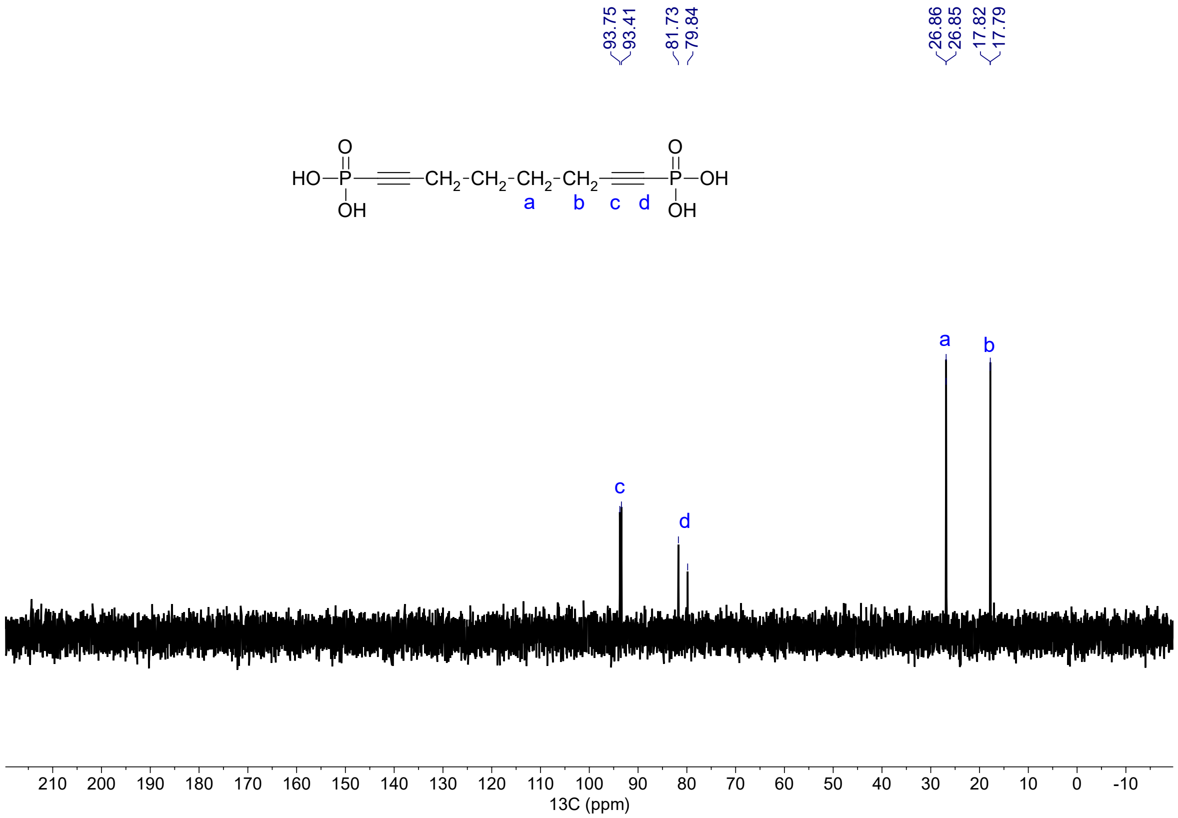

Figure S29: ^{13}C NMR spectrum of **1f** in D_2O at 25 °C, recorded at 126 MHz.

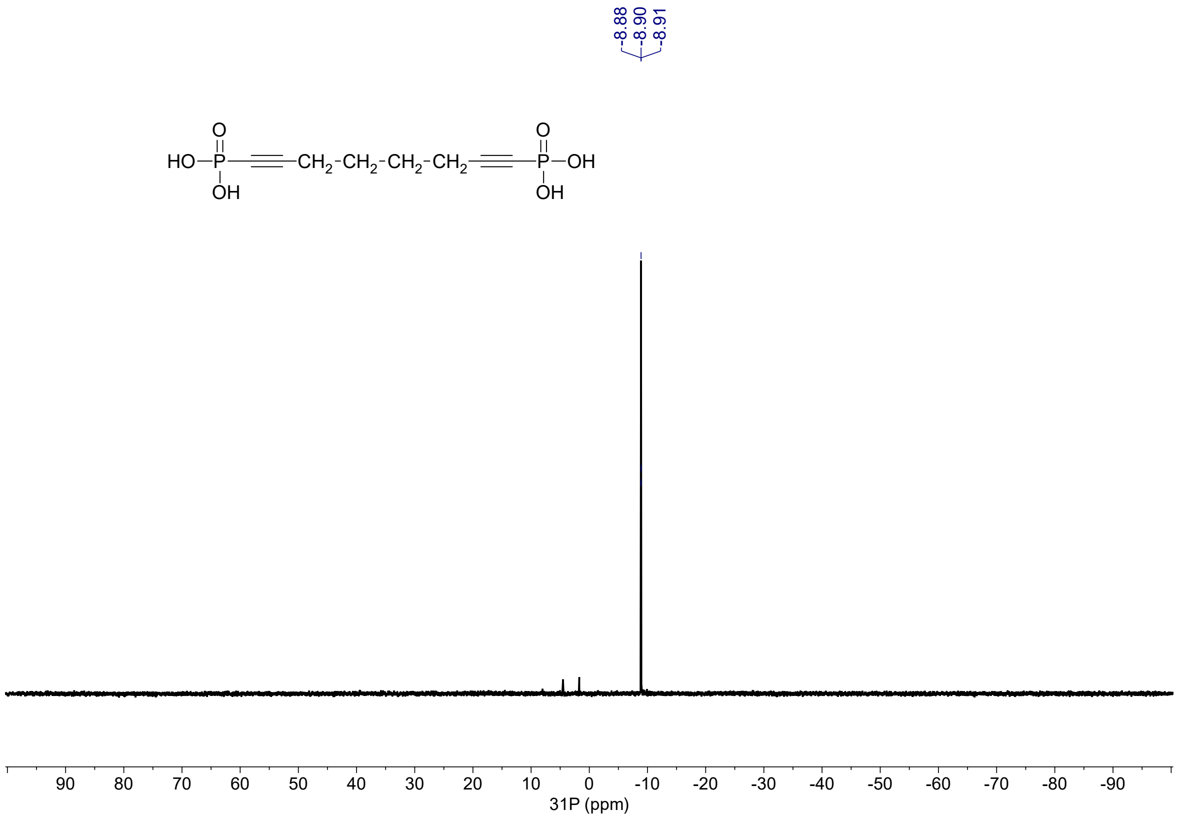

Figure S30: ^{31}P NMR spectrum of **1f** in D_2O at 25 °C, recorded at 203 MHz.

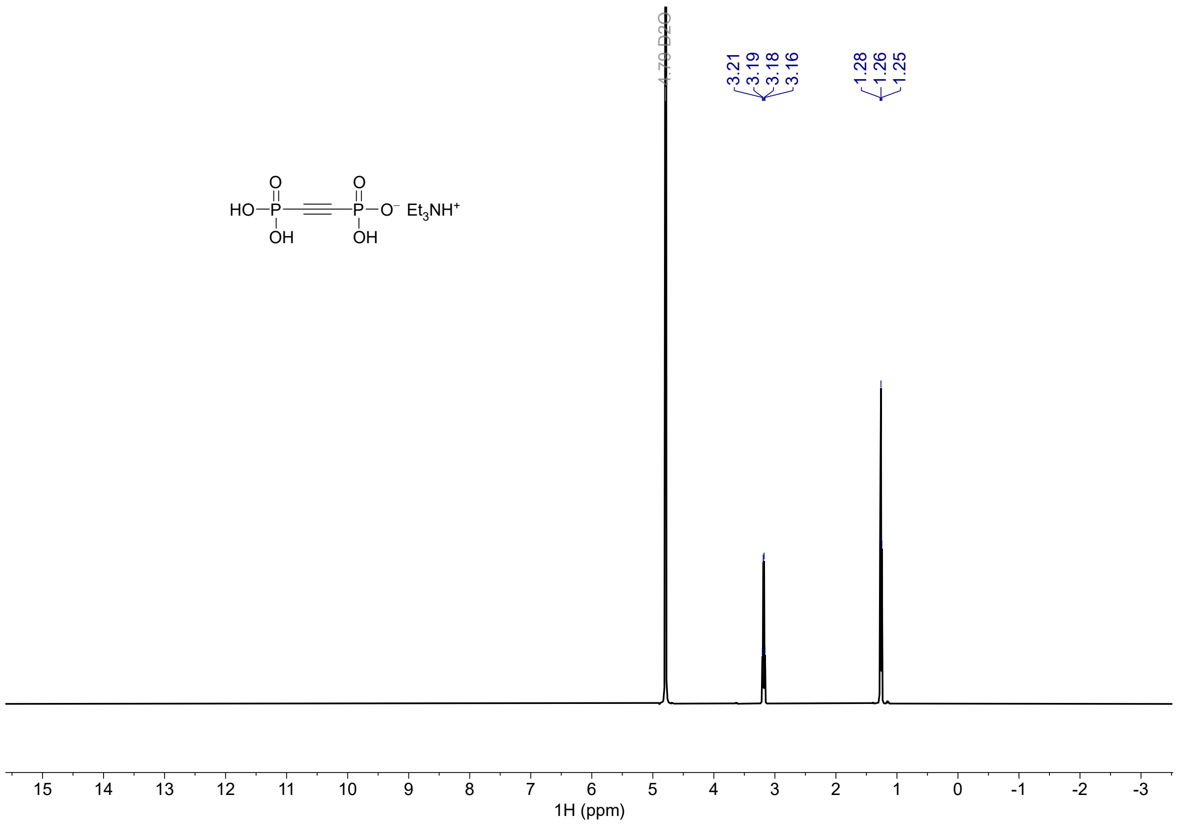

Figure S31: ^{1}H NMR spectrum of **1g** (triethylammonium salt) in D_2O at 25 °C, recorded at 500 MHz.

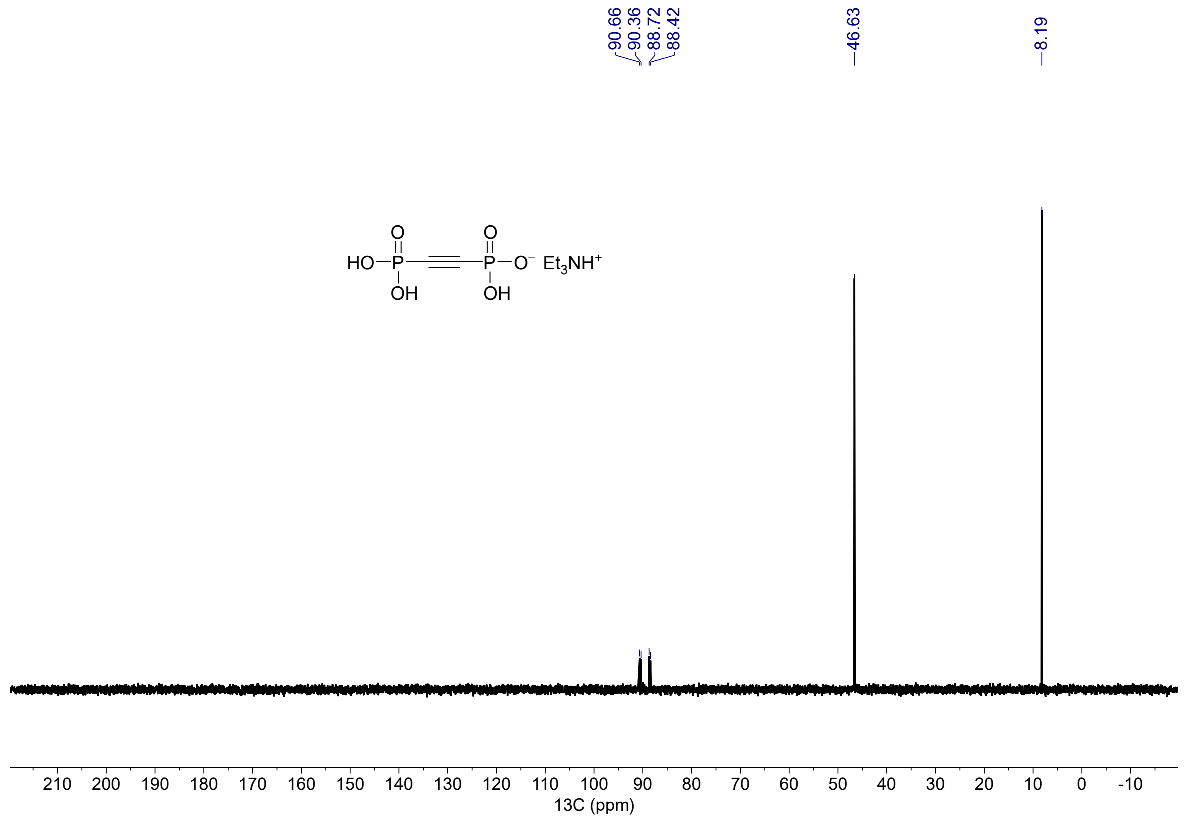

Figure S32: ^{13}C NMR spectrum of **1g** (triethylammonium salt) in D_2O at 25 °C, recorded at 126 MHz.

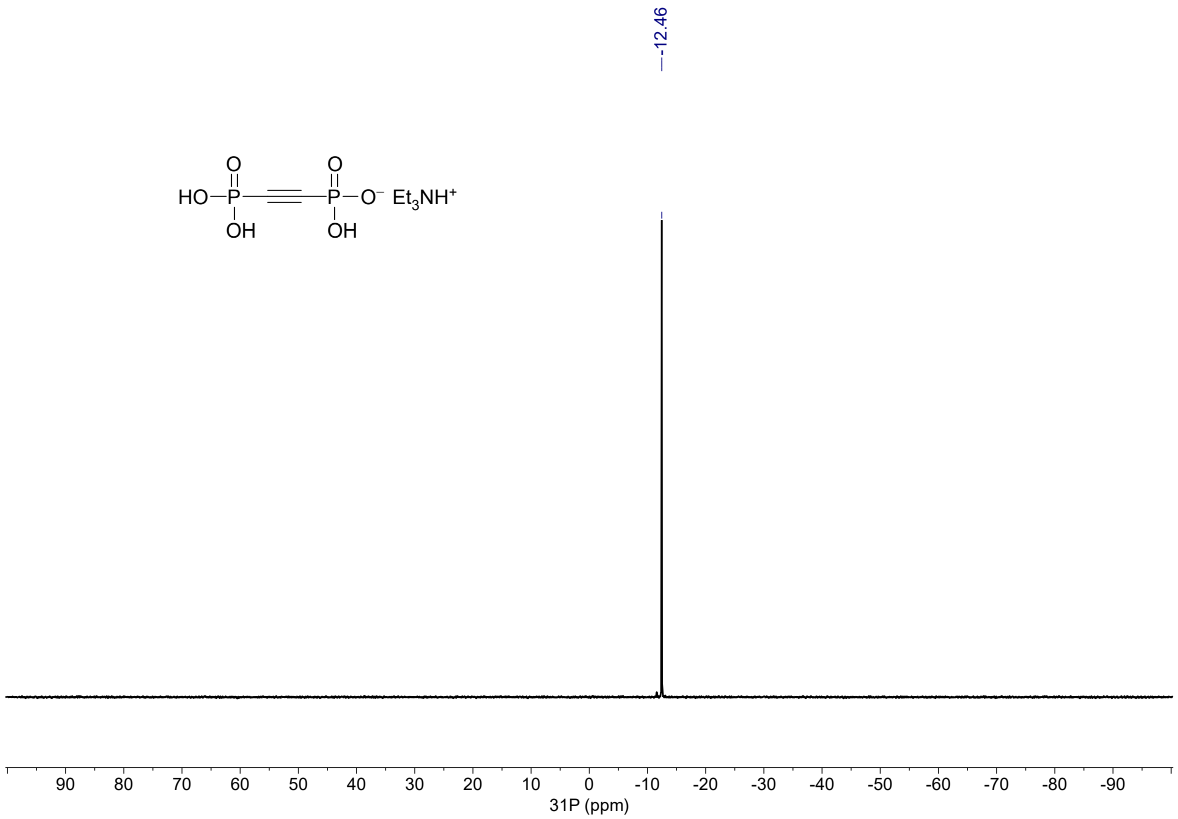

Figure S33: ^{31}P NMR spectrum of **1g** (triethylammonium salt) in D_2O at 25 °C, recorded at 203 MHz.

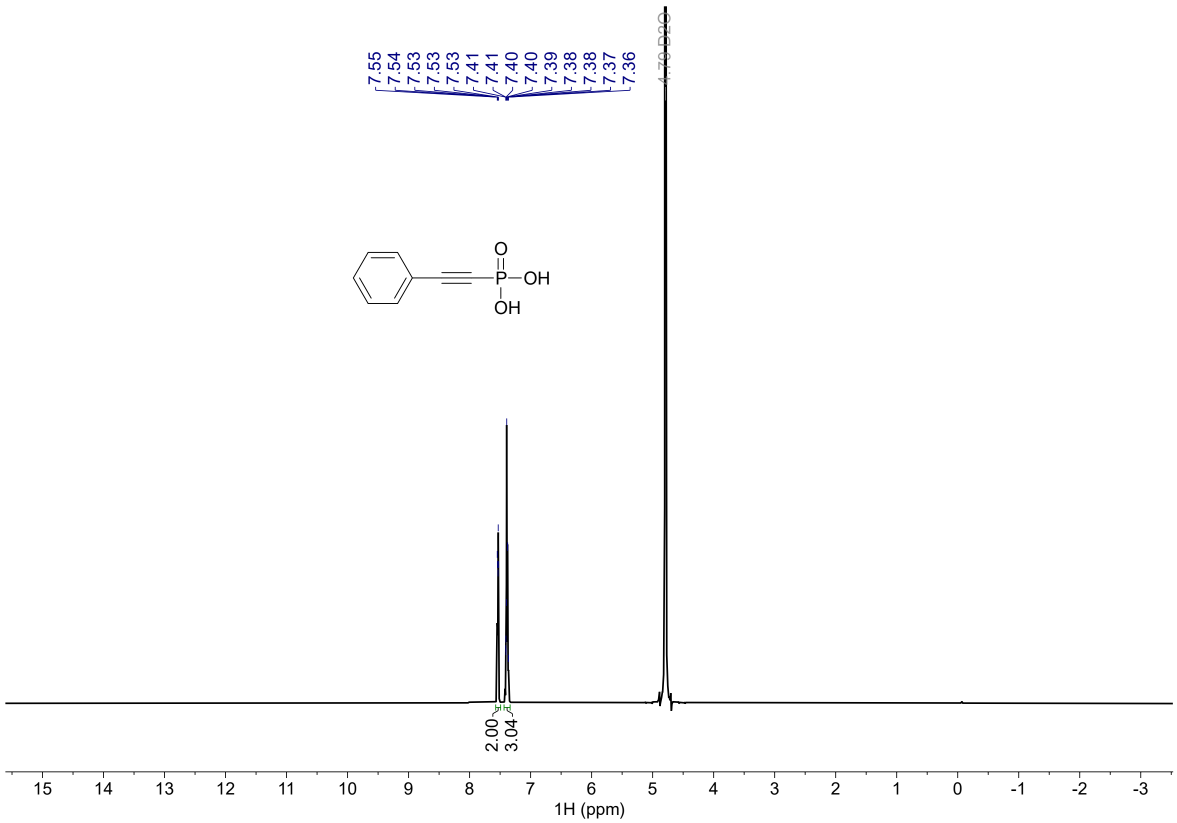

Figure S34: ^{1}H NMR spectrum of **1h** in D_2O at 25 °C, recorded at 500 MHz.

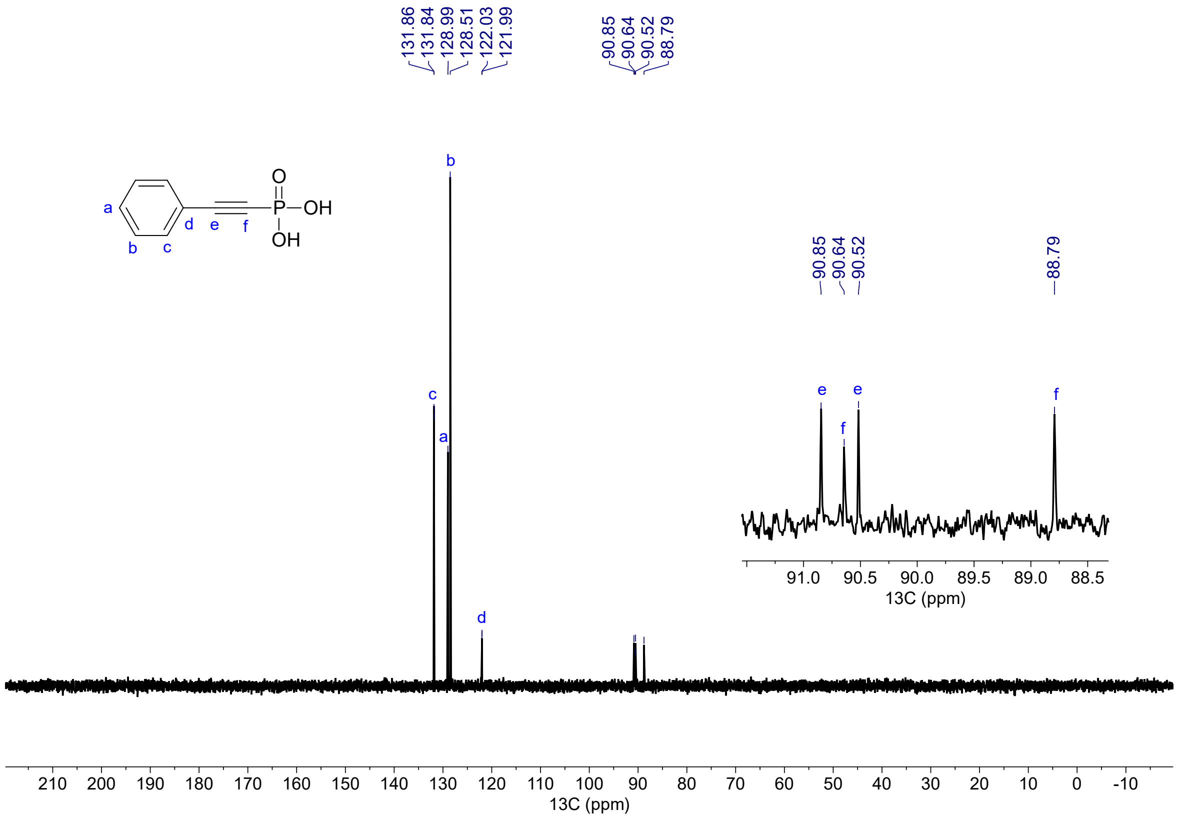

Figure S35: ^{13}C NMR spectrum of **1h** in D_2O at 25 °C, recorded at 126 MHz.

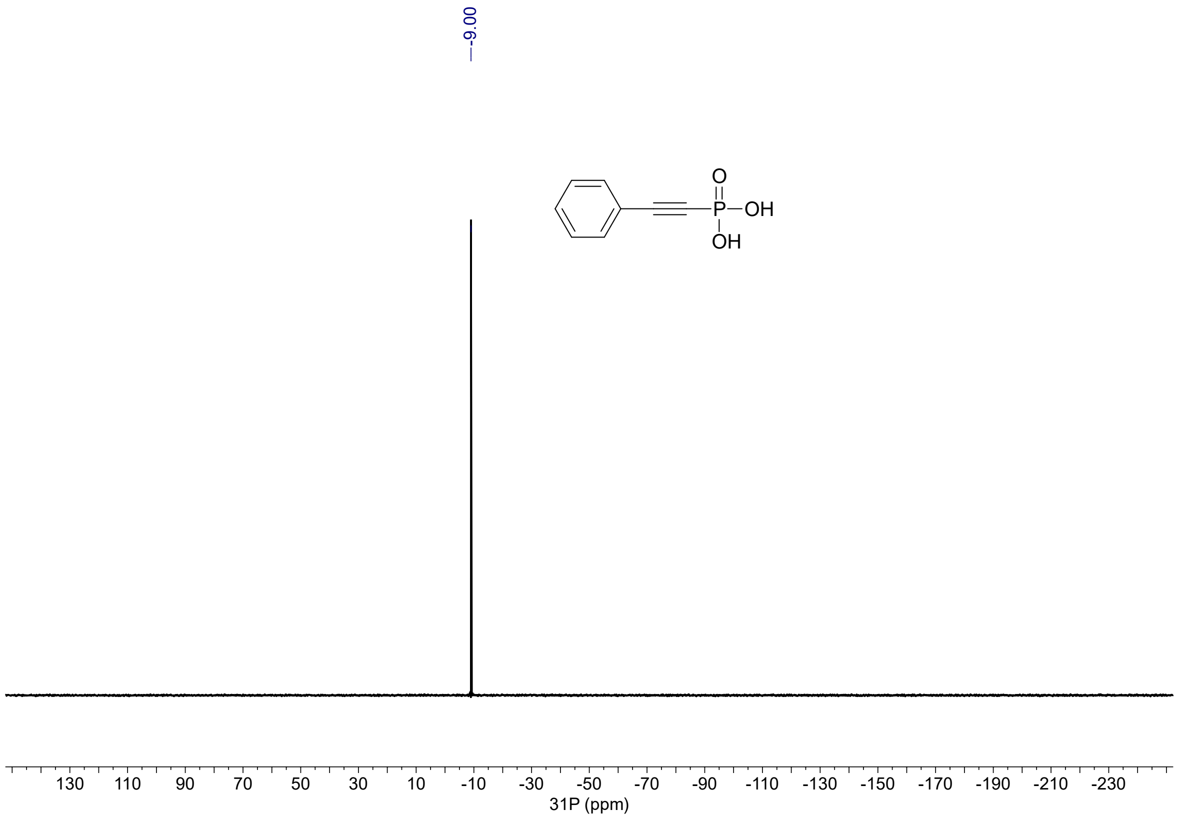

Figure S36: ^{31}P NMR spectrum of **1h** in D_2O at 25 °C, recorded at 203 MHz.

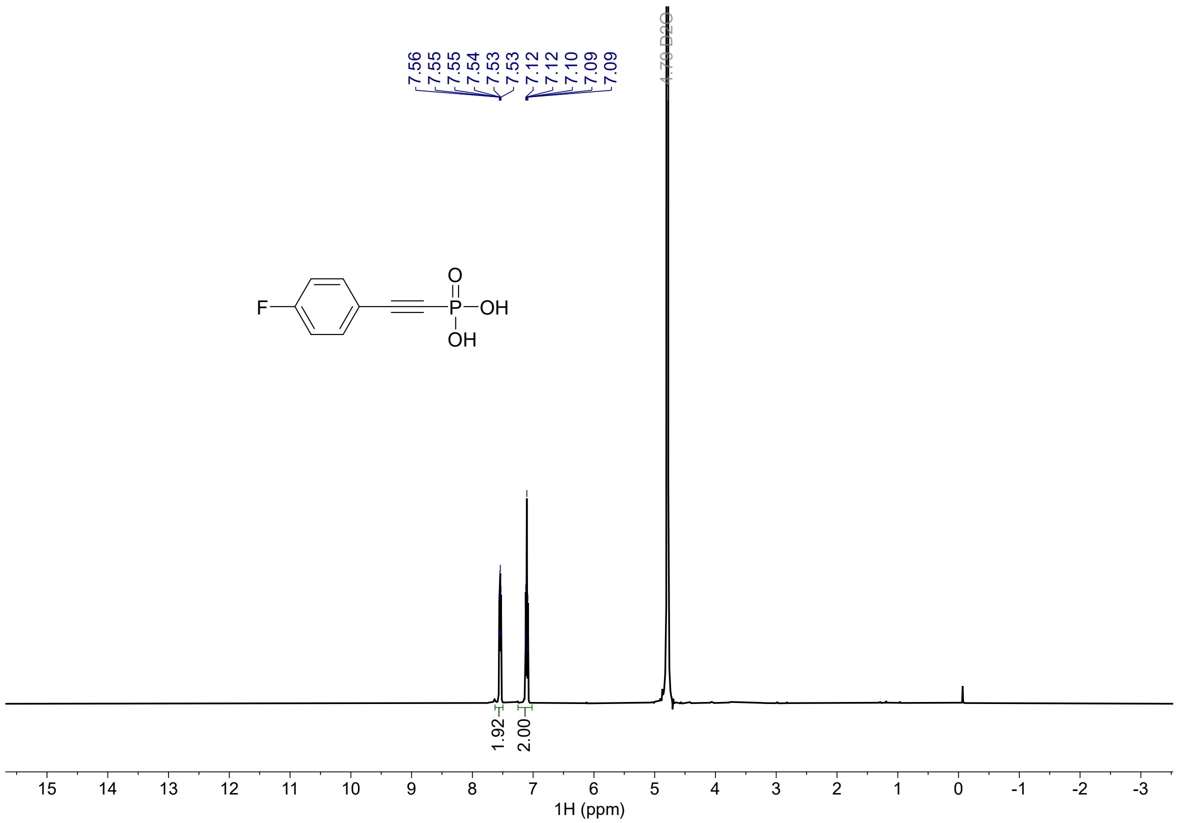

Figure S37: ^{1}H NMR spectrum of **1i** in D_2O at 25 °C, recorded at 500 MHz.

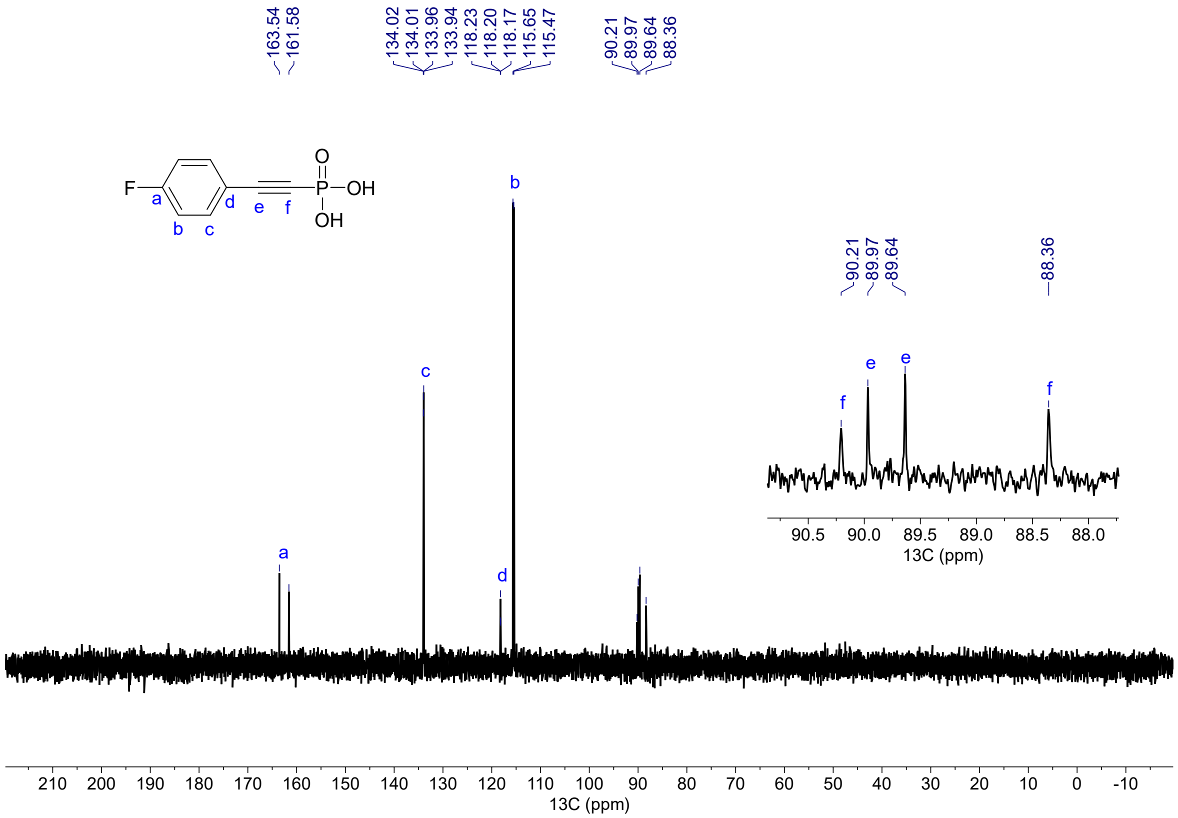

Figure S38: ^{13}C NMR spectrum of **1i** in D_2O at 25 °C, recorded at 126 MHz.

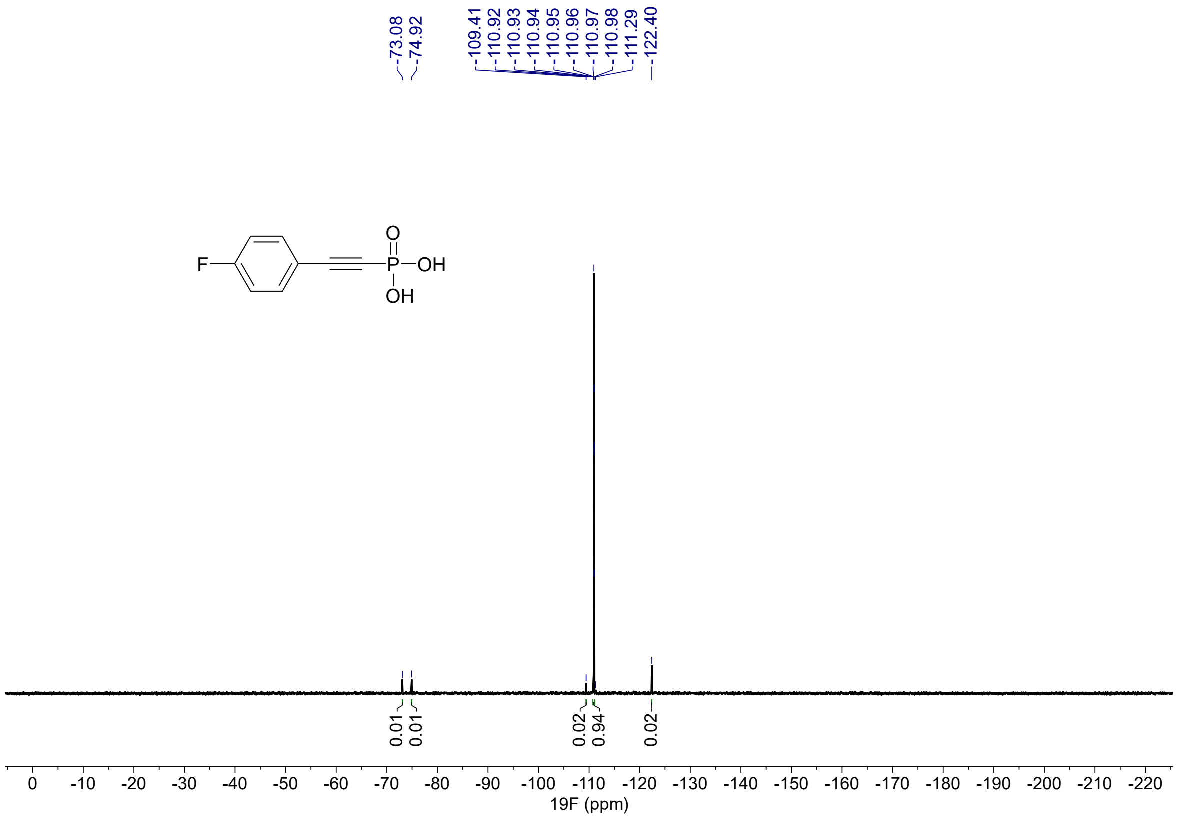

Figure S39: ^{19}F NMR spectrum of **1i** in D_2O at 25 °C, recorded at 471 MHz.

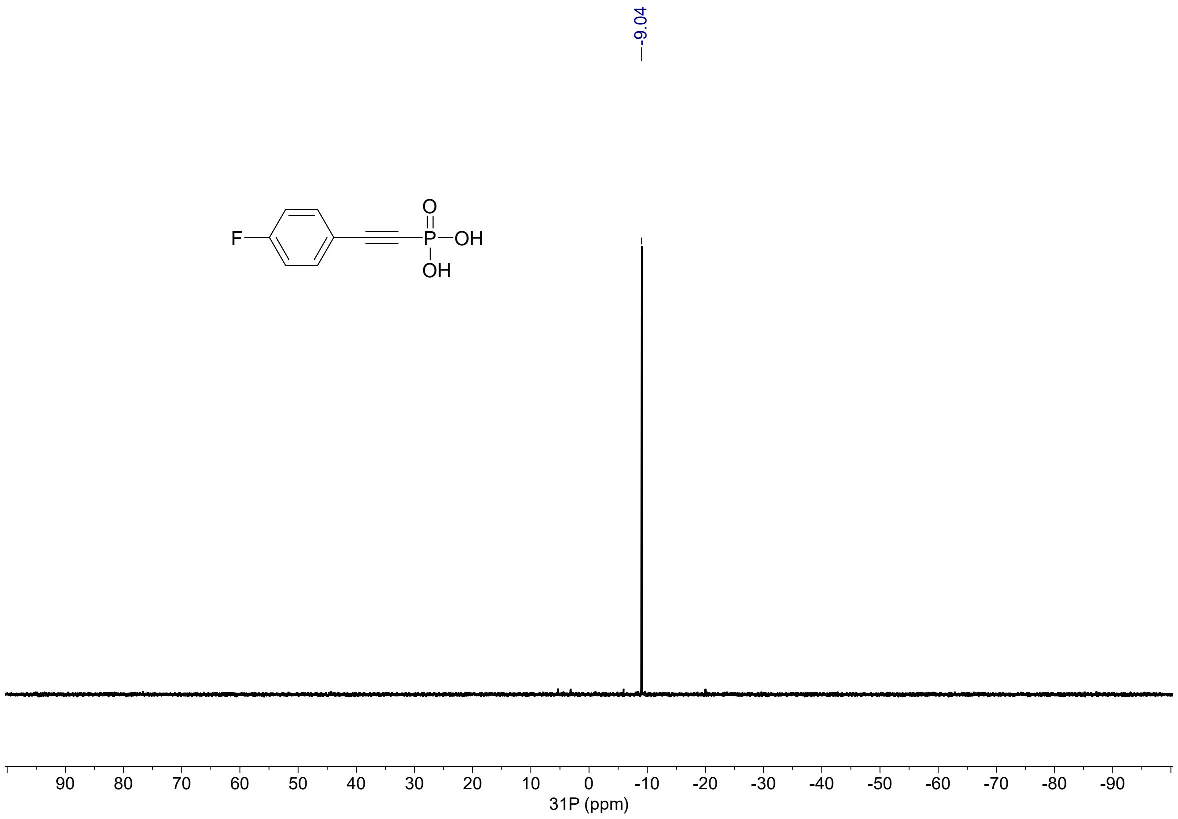

Figure S40: ^{31}P NMR spectrum of **1i** in D_2O at 25 °C, recorded at 203 MHz.

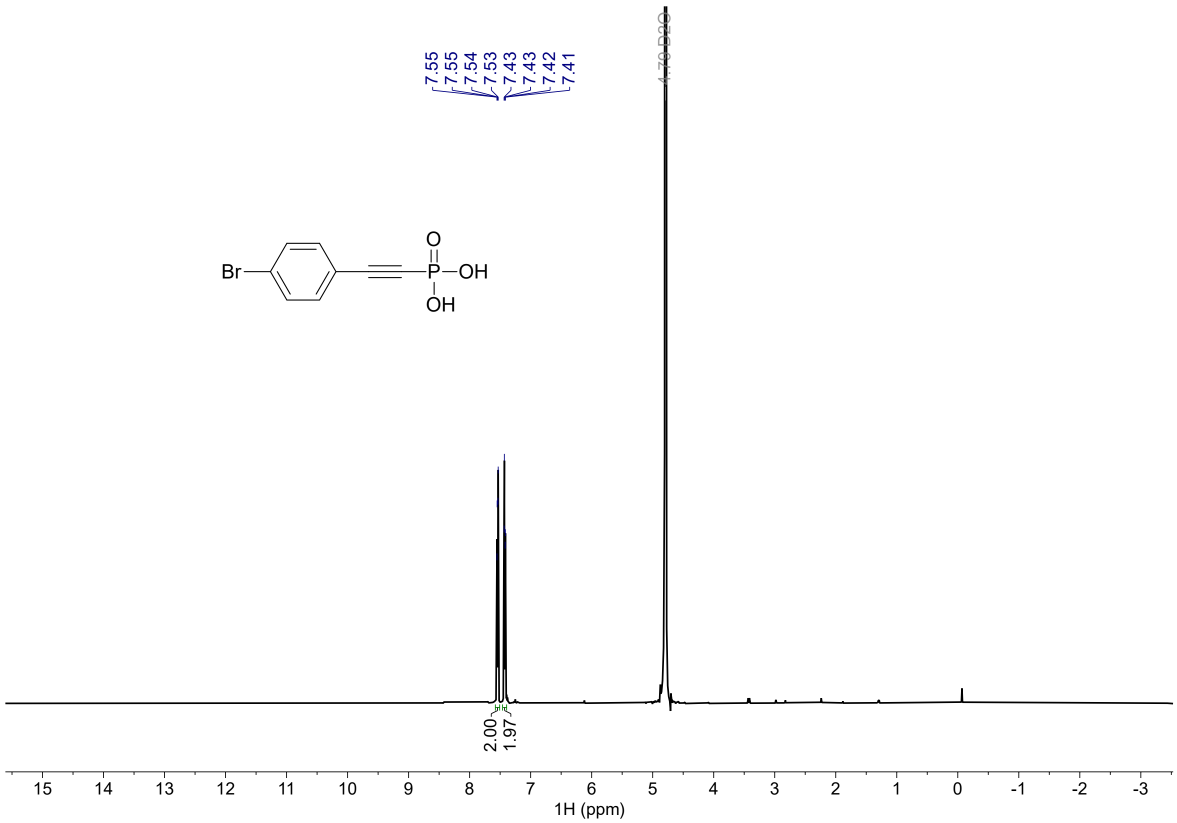

Figure S41: ^{1}H NMR spectrum of **1j** in D_2O at 25 °C, recorded at 500 MHz.

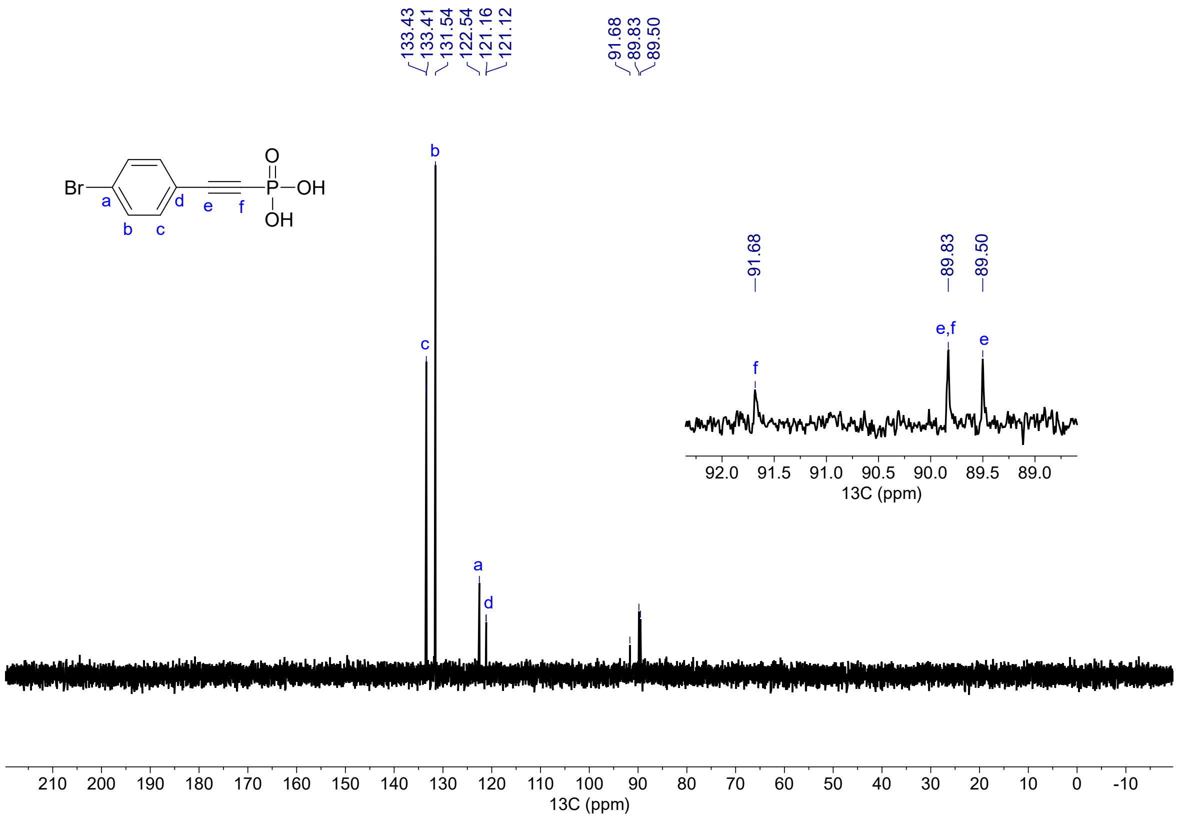

Figure S42: ^{13}C NMR spectrum of **1j** in D_2O at 25 °C, recorded at 126 MHz.

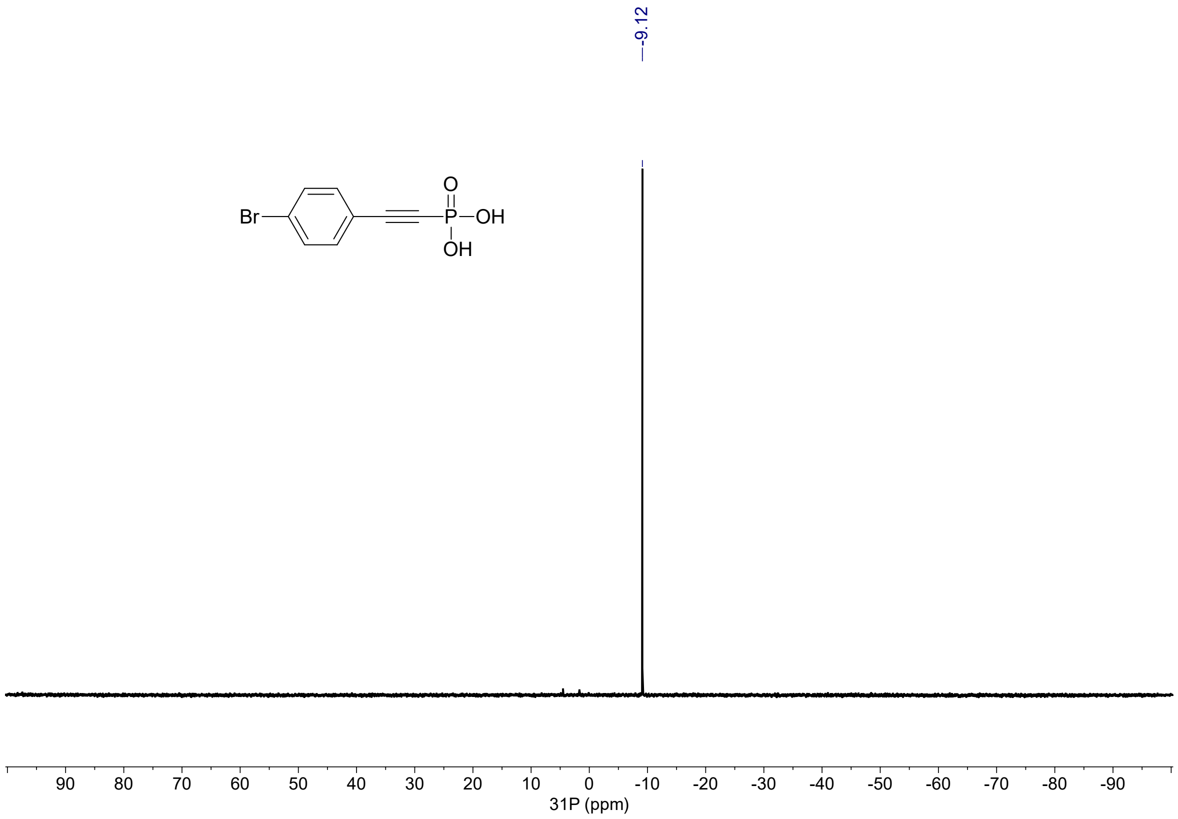

Figure S43: ^{31}P NMR spectrum of **1j** in D_2O at 25 °C, recorded at 203 MHz.

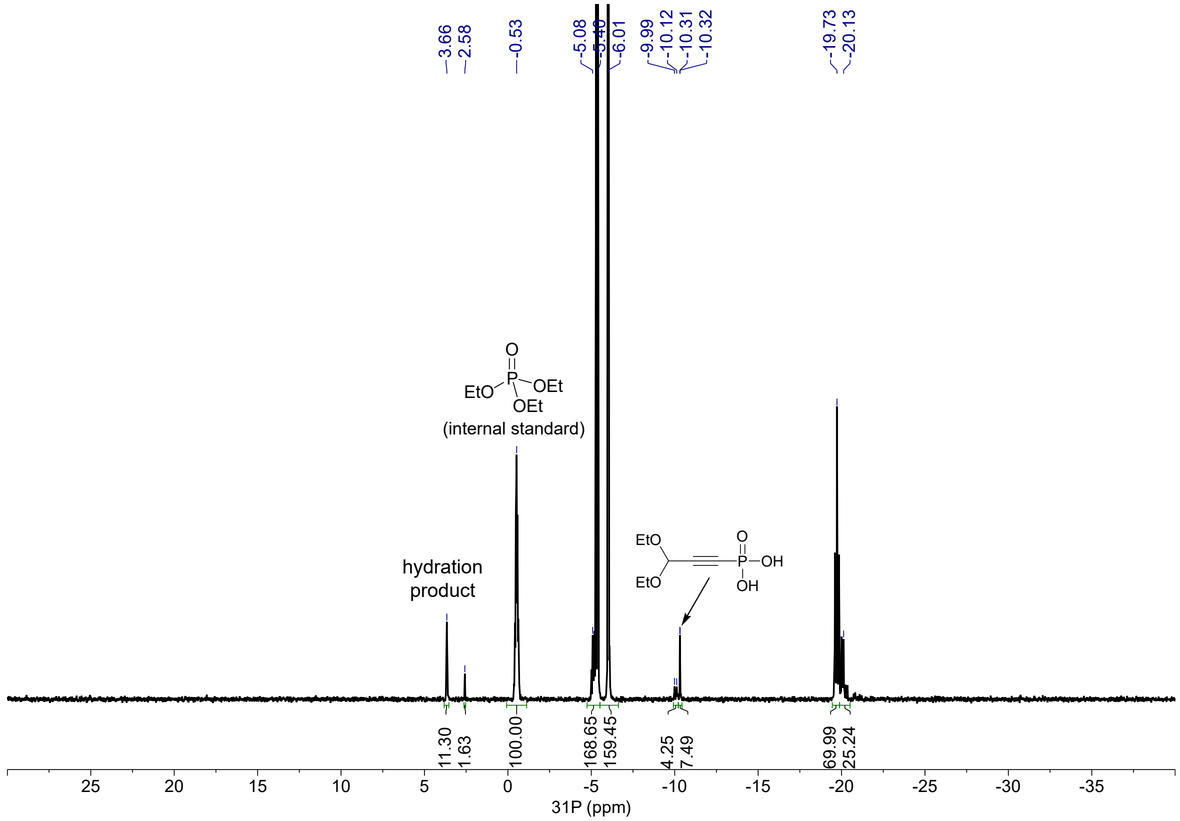

Figure S44: ^{31}P NMR spectrum of the mixture resulting from reaction of $Na_5P_3O_{10}$ and KC≡CCH$(OEt)_2$ (401 mg of crude solid with 104 mg of $PO(OEt)_3$ added).

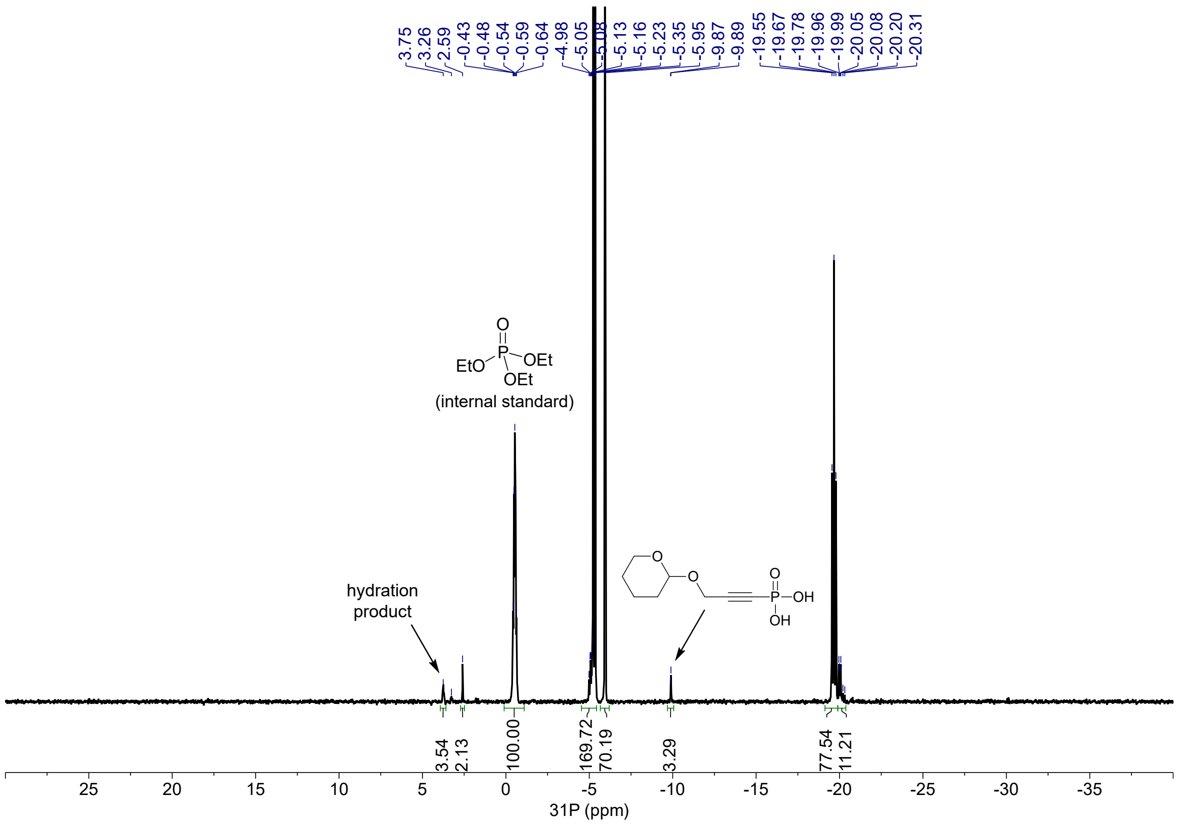

Figure S45: ^{31}P NMR spectrum of the mixture resulting from reaction of $Na_5P_3O_{10}$ and KC≡CCH_2THP (389 mg of crude solid with 137 mg of $PO(OEt)_3$ added).

S4 Synthesis of ethynyl phosphonate

S4.1 Triethylammonium hydrogen ethynyl phosphonate 2

Note: The results of mechanochemical reactions are heavily affected by actual milling conditions, including but not limited to grinding time, grinding speed or frequency, material and shape of jars and balls, and reaction scale. The reaction time and speed used herein may not be optimal for milling conducted under other conditions. Reoptimization of reaction conditions may be necessary and is recommended for reactions conducted using a different setup.

In the glovebox, a 125 mL stainless steel milling jar was charged with $Na_4P_2O_7$ (5.32 g, 20 mmol, 1.0 equiv), Na_2C_2 (2.10 g, 30 mmol, 1.5 equiv), and 30 grinding balls. The milling jar was sealed with the safety closure device, removed from the glovebox, and mounted onto the planetary ball mill. Milling was first conducted at a rotational speed of 200 rpm for 10 min to mix up the starting materials, and then conducted at 450 rpm for 36 h (with 30 min of break time and direction reversal after every 30 min of grinding for a total of 72 h). After cooling in an ice bath, the jar was opened in air and the milled material was dissolved in deionized ice water (200 mL). *Note: Residual acetylides react with water to generate heat and may release flammable or hazardous gas. Hence, this addition of ice water should be performed in a well-ventilated area and be slow at the beginning.* Care was taken to dissolve all solid material in the jar and on the lid. The obtained solution was transferred to a 1 L flask equipped with a stir bar.

$Ca(OH)_2$ (3.0 g, 40 mmol, 2.0 equiv) was added in one portion with vigorous stirring, resulting in precipitation. Stirring was continued for at least 2 h. An aliquot of the supernatant was analyzed by ^{31}P NMR spectroscopy, showing no remaining orthophos-

phate signal (a singlet at ca. 5 ppm). If orthophosphate or pyrophosphate (a singlet at ca. –7 ppm) signals are still present, another 0.25 g of $Ca(OH)_2$ should be added to the suspension followed by stirring for another 1 h. This process should be repeated until no orthophosphate signal is present in ^{31}P NMR spectra. The suspension was filtered through a plug of Celite (centrifuging the suspension before filtration may aid this process, as this filtration often proves to be difficult). The collected precipitate was transferred back to the flask and suspended in another 80 mL of deionized water. The suspension was stirred for 30 min and the supernatant was checked by ^{31}P NMR spectroscopy again for orthophosphate. The suspension was filtered through a plug of Celite. To the combined filtrate was added conc. HCl (ca. 10 mL) to reach pH≤1 (measured by pH strips). Volatile materials were removed from the resulting solution on a rotovap at 50 °C. The resulting solid was further dried under dynamic vacuum for 15 min.

The residue was suspended in acetone (100 mL) and HCl (2.0 M in Et_2O, 3 mL) in a 250 mL flask, and the suspension was stirred vigorously for 30 min before being filtered through a plug of Celite. The solid and Celite were washed with a mixture of acetone (50 mL) and HCl (2.0 M in Et_2O, 1.5 mL). Volatile materials were removed from the combined filtrate on a rotovap at 30 °C, and the oily residue was further dried under dynamic vacuum for 15 min. The residue was redissolved in acetone (80 mL), and NEt_3 (5.5 mL, 40 mmol, 2.0 equiv) was added to the solution dropwise with vigorous stirring. Vigorous stirring was continued overnight to break down chunks of crude product formed at the bottom of the flask (the process can be accelerated by sonication). The precipitate was collected on a medium frit, washed with acetone (2×15 mL) and dried under dynamic vacuum to yield the title compound **2** as an off-white powder (1.70 g, 8.2 mmol, 41%). The filtrate was concentrated to ca. 30 mL and placed at –25 °C overnight. The formed precipitate was collected on a medium frit, washed with acetone (2×10 mL) and dried

under dynamic vacuum to yield **2** as an off-white powder (0.91 g, 4.4 mmol, 22%). The two crops of product were combined for a total yield of 63%.

Anal. Calcd for $C_8H_{18}NO_3P$: C, 46.37; H, 8.76; N, 6.76. Found: C, 45.25; H, 8.35; N, 6.48 (Note: the results of elemental analysis were slightly off due to partial loss of NEt_3 during drying). ^{1}H NMR (500 MHz, CD_3CN, Figure S46) δ 11.89 (br, 2H), 3.33 – 2.88 (m, 6H), 2.73 (d, J = 11.3 Hz, 1H), 1.27 (t, J = 6.3 Hz, 9H) ppm. ^{13}C NMR (126 MHz, CD_3CN, Figure S47) δ 85.53 (d, J = 247.6 Hz), 81.09 (d, J = 43.9 Hz), 46.49, 8.97 ppm. ^{31}P NMR (203 MHz, CD_3CN, Figure S48) δ –12.63 (d, J = 11.2 Hz) ppm. ESI-MS(–) (m/z): $[M - H]^-$ calcd for $C_2H_3O_3P$, 104.9747; found, 104.9750.

S4.2 Bis(triethylammonium) ethynyl phosphonate 2″

Triethylammonium hydrogen ethynyl phosphonate **2** (1.75 g, 8.5 mmol, 1.0 equiv) was dissolved in absolute ethanol (30 mL) and NEt_3 (2.3 mL, 17 mmol, 2.0 equiv) was added. Et_2O (60 mL) was added to the solution in portions, resulting in a cloudy solution. The solution was filtered, and Et_2O (30 mL) was carefully layered on top of the filtrate. The mixture was placed at –25 °C overnight. The formed crystals were collected on a medium frit and dried under reduced pressure to yield the title compound **2″** as a colorless crystalline solid (1.35 g, 4.9 mmol, 52%).

^{1}H NMR (500 MHz, CD_3CN, Figure S49) δ 11.89 (br, 2H), 3.01 (q, J = 7.3 Hz, 12H), 2.69 (d, J = 11.3 Hz, 1H), 1.25 (t, J = 7.3 Hz, 18H) ppm. ^{13}C NMR (126 MHz, CD_3CN, Figure S50) δ 85.58 (d, J = 247.4 Hz), 81.04 (d, J = 44.0 Hz), 46.49, 8.95 ppm. ^{31}P NMR (203 MHz, CD_3CN, Figure S51) δ –12.63 (d, J = 11.1 Hz) ppm.

S4.3 Bis(tetra-*n*-butylammonium) ethynyl phosphonate **2′**

Bis(triethylammonium) ethynyl phosphonate **2″** (0.308 g, 1.0 mmol) was dissolved in deionized water (10 mL). The solution was passed through a column (ca. 10 mL) of Amberchrom (formerly DOWEX) 50WX8 (TBA^+) resin. Another 40 mL of water was used to elute out the product. The obtained solution was lyophilized to afford a pale yellow liquid, which was transferred into a glovebox. The liquid was dissolved in dry DCM (10 mL), and insoluble material was filtered off. Volatile material was removed under reduced pressure to yield the title compound **2′** as a colorless liquid (0.432 g, 0.73 mmol, 73%). The compound is prone to oligomerization, and is recommended to be stored at low temperature under a dry atmosphere.

^{1}H NMR (500 MHz, CD_3CN, Figure S52) δ 3.30 – 2.95 (m, 16H), 2.44 (d, J = 10.5 Hz, 1H), 1.61 (dq, J = 11.9, 8.2, 7.7 Hz, 16H), 1.36 (h, J = 7.4 Hz, 16H), 0.97 (t, J = 7.3 Hz, 24H) ppm. ^{31}P NMR (203 MHz, CD_3CN, Figure S53) δ –14.02 (d, J = 10.6 Hz) ppm.

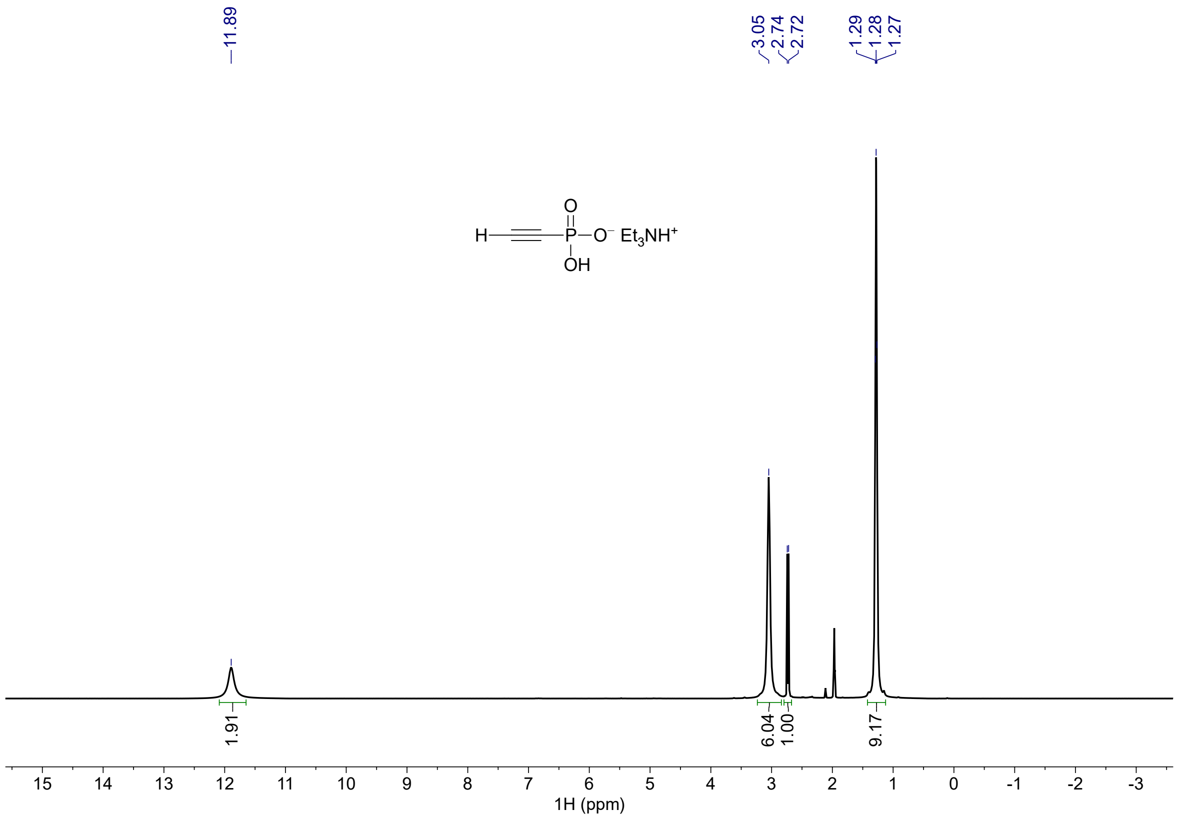

Figure S46: ^{1}H NMR spectrum of **2** in CD_3CN at 25 °C, recorded at 500 MHz.

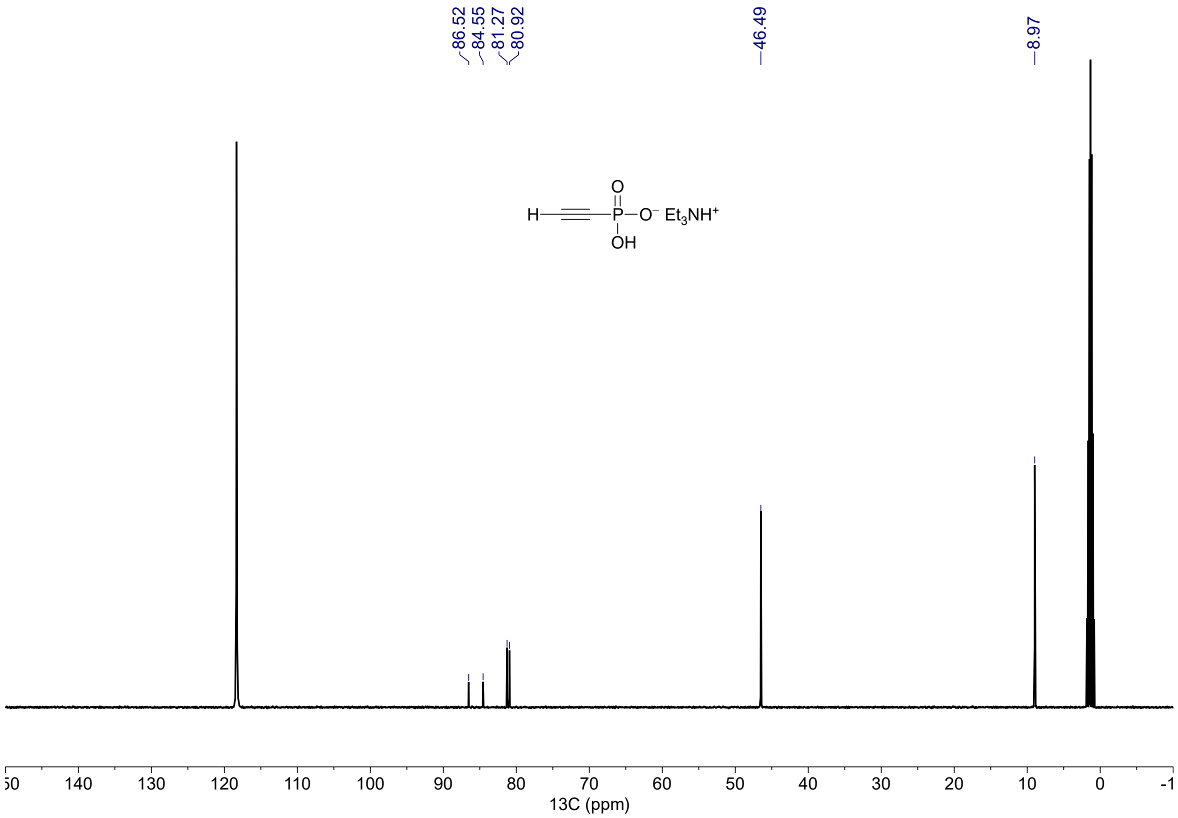

Figure S47: ^{13}C NMR spectrum of **2** in CD_3CN at 25 °C, recorded at 126 MHz.

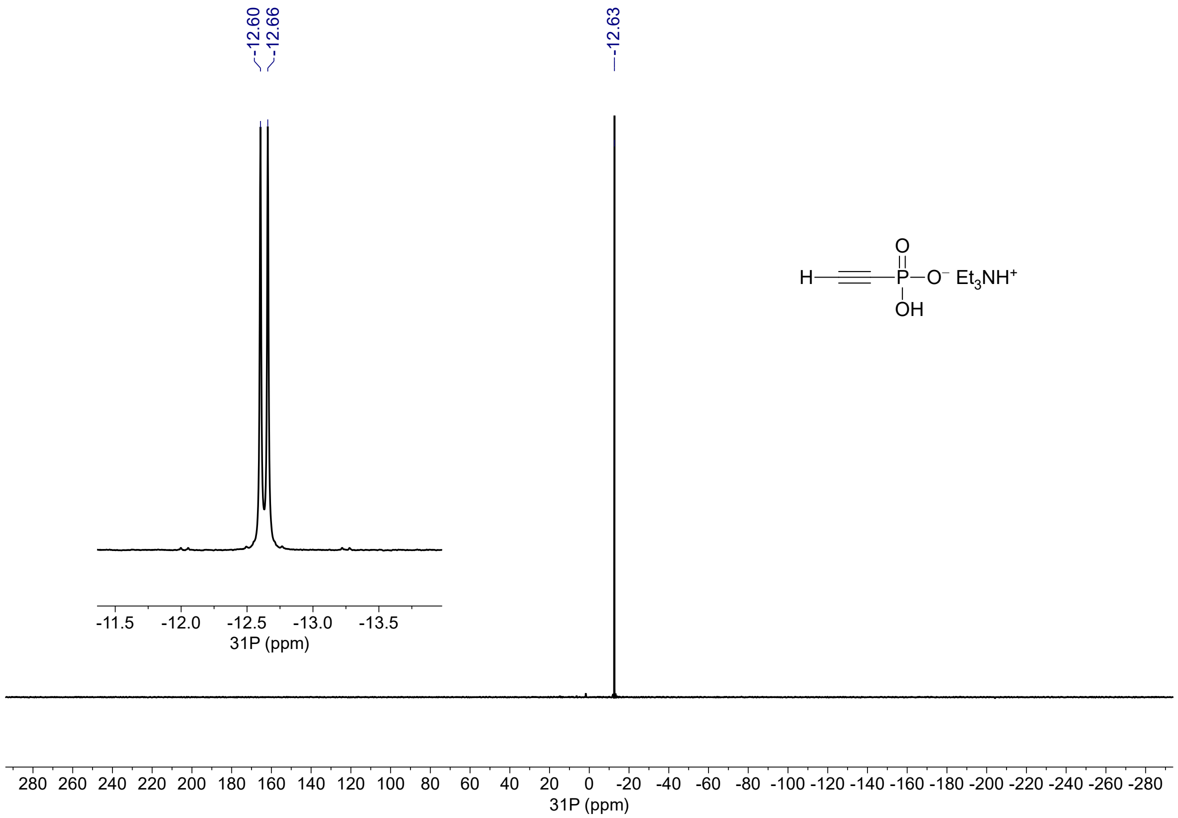

Figure S48: $^{31}P\{^{1}H\}$ NMR spectrum of **2** in CD_3CN at 25 °C, recorded at 203 MHz. Inset: ^{31}P NMR spectrum of **2** in CD_3CN at 25 °C, recorded at 203 MHz.

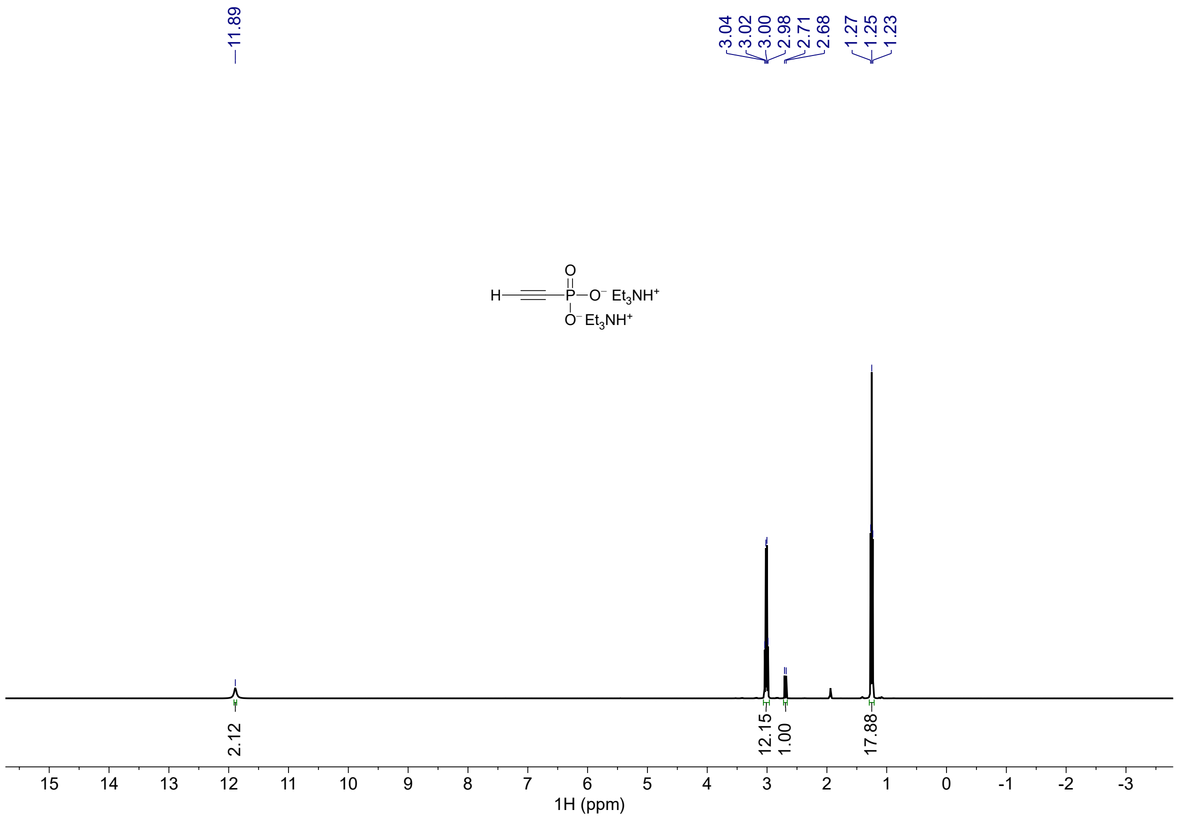

Figure S49: ^{1}H NMR spectrum of **2″** in CD_3CN at 25 °C, recorded at 500 MHz.

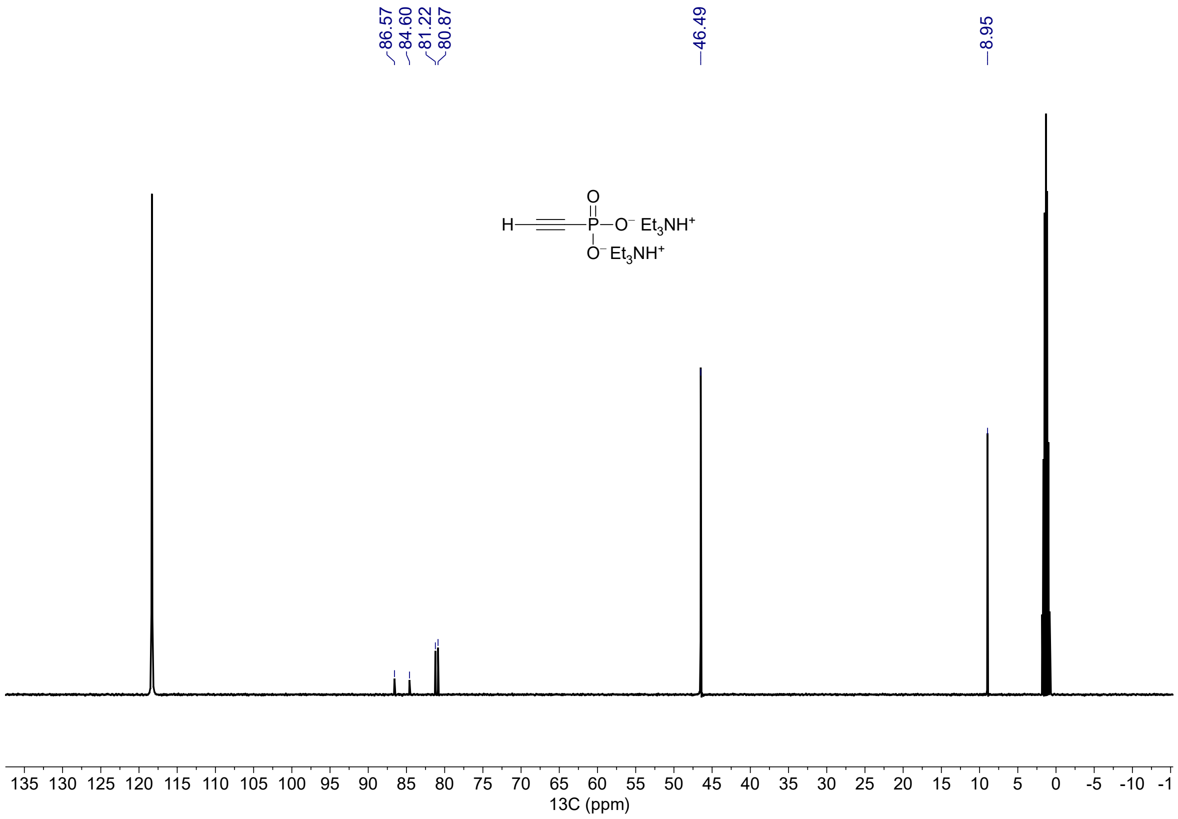

Figure S50: ^{13}C NMR spectrum of **2″** in CD_3CN at 25 °C, recorded at 126 MHz.

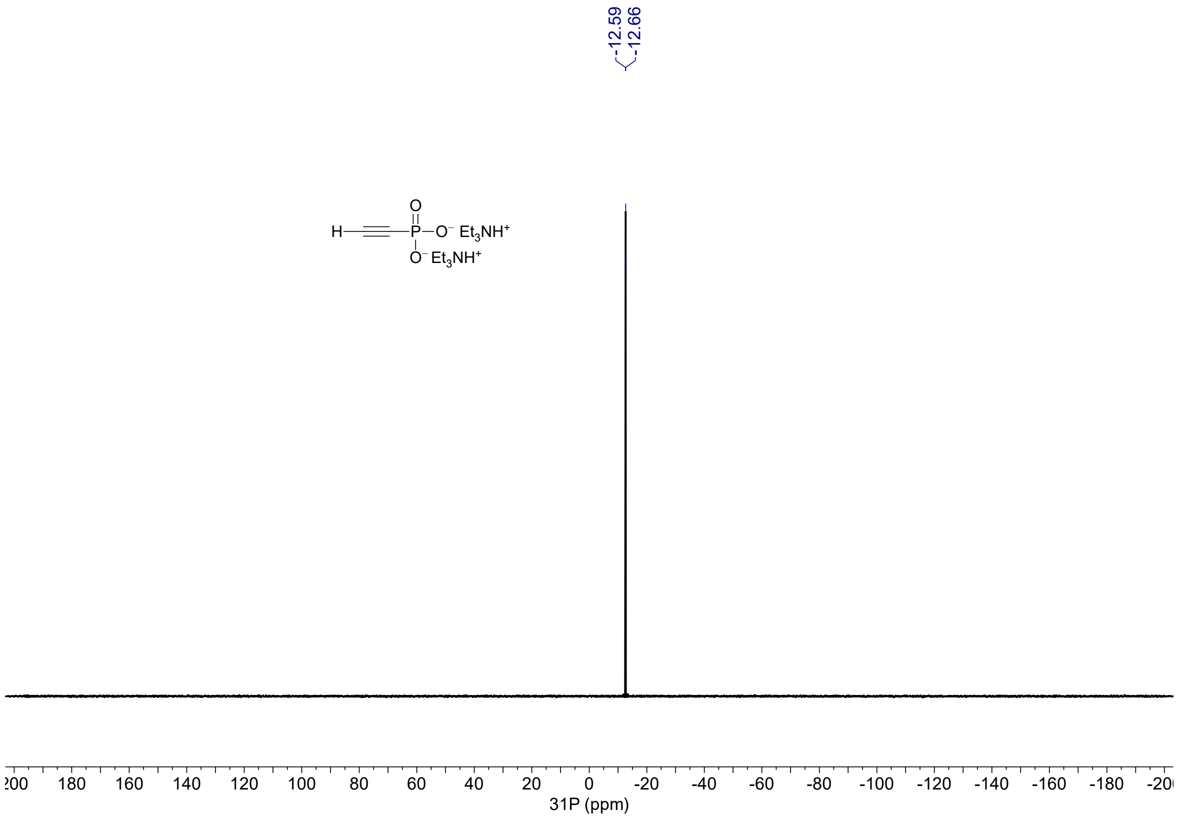

Figure S51: ^{31}P NMR spectrum of **2′′** in CD_3CN at 25 °C, recorded at 203 MHz.

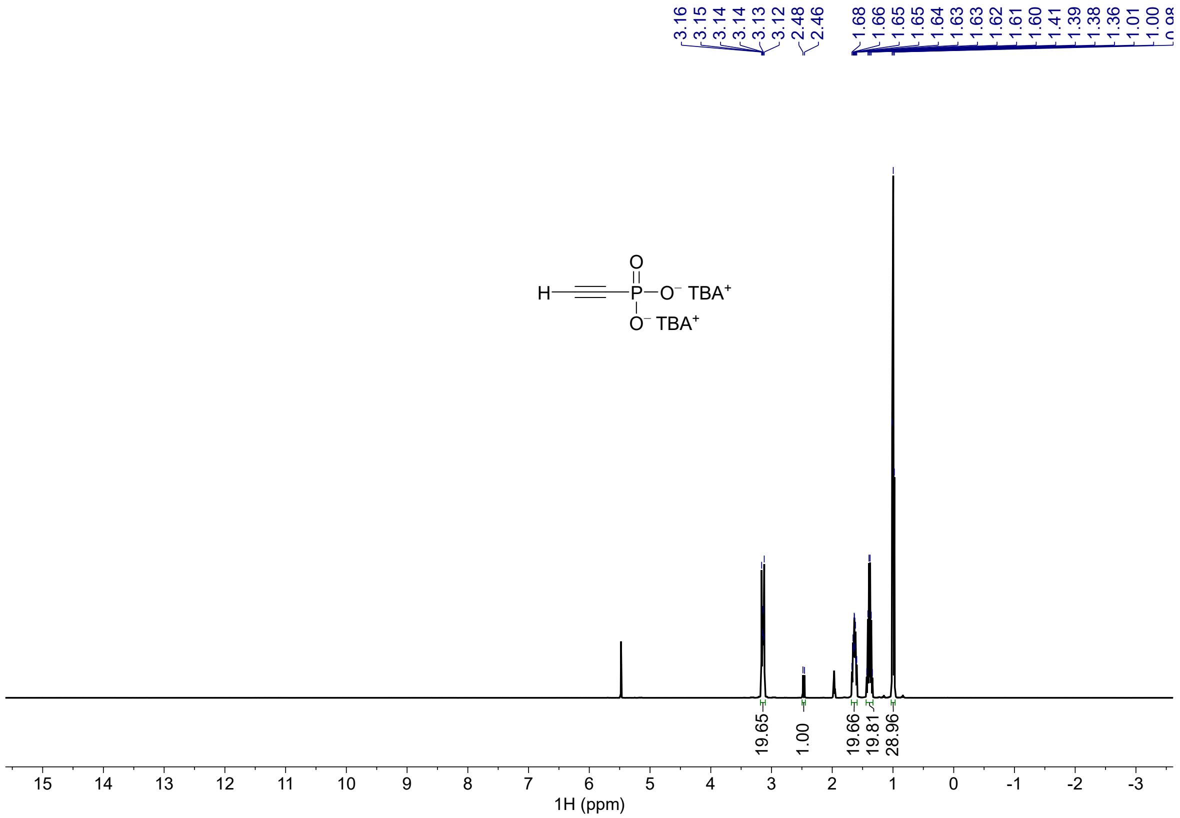

Figure S52: ^{1}H NMR spectrum of **2′** in CD_3CN at 25 °C, recorded at 500 MHz.

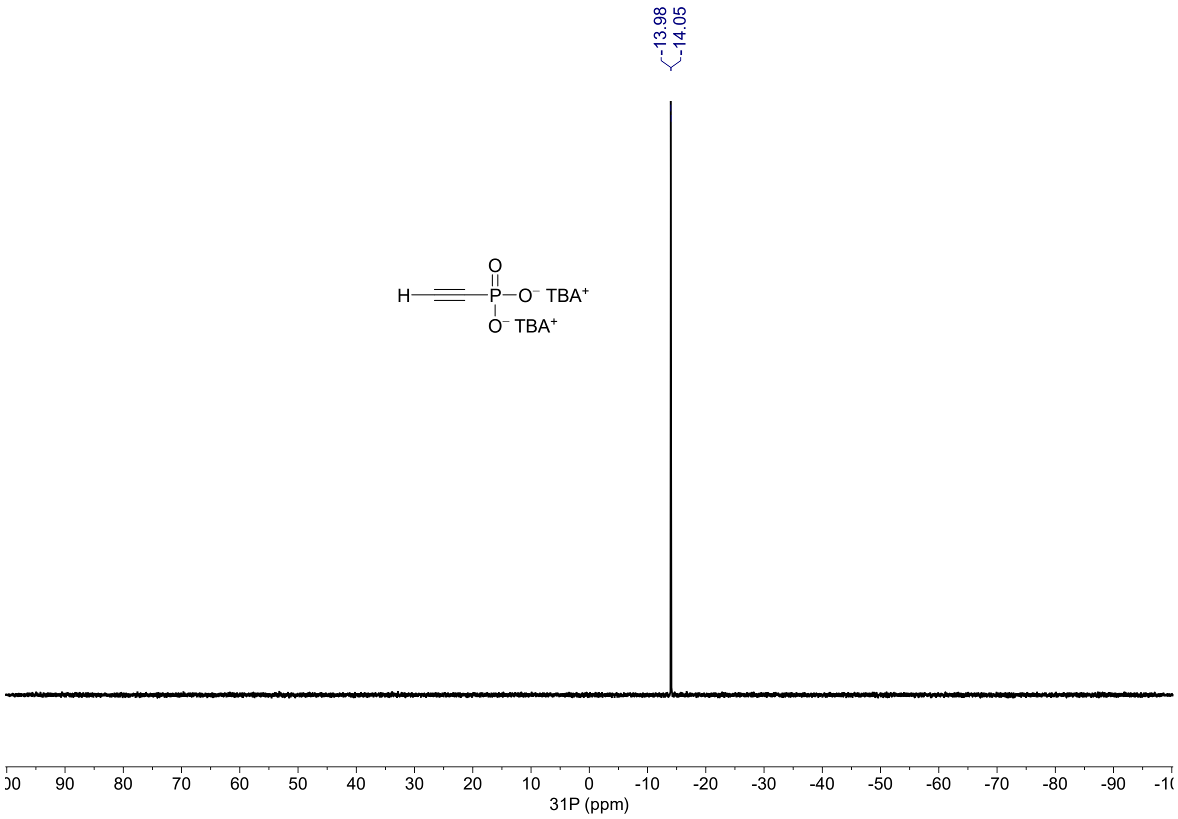

Figure S53: ^{31}P NMR spectrum of **2′** in CD_3CN at 25 °C, recorded at 203 MHz.

S5 Functionalization of ethynyl phosphonate

S5.1 Synthesis of bis(trimethylsilyl) ethynyl phosphonate 2a

In the glovebox, triethylammonium hydrogen ethynyl phosphonate **2** (2.48 g, 12 mmol) was suspended in dry hexanes (100 mL) in a 200 mL flask. Pyridine (1.42 g, 18 mmol, 1.5 equiv) was added to the suspension, followed by chlorotrimethylsilane (2.59 g, 24 mmol, 2.0 equiv). The suspension was stirred vigorously for 16 h at 23 °C. The mixture was filtered through Celite with the help of 30 mL of hexanes. The filtrate was concentrated to ca. 10 mL and placed at –35 °C overnight. The formed colorless needle crystals were collected on a frit, washed with cold (–35 °C) penatne and dried under reduced pressure to yield **2a** as a white crystalline solid (2.15 g, 8.6 mmol, 72%). The compound is prone to hydrolysis and oligomerization, and is recommended to be stored at low temperature under dry atmosphere.

^{1}H NMR (500 MHz, $CDCl_3$, Figure S54) δ 2.77 (d, J = 13.6 Hz, 1H), 0.33 (s, 18H) ppm. ^{13}C NMR (126 MHz, $CDCl_3$, Figure S55) δ 85.10 (d, J = 54.7 Hz), 78.16 (d, J = 301.5 Hz), 0.90 (d, J = 1.8 Hz) ppm. ^{31}P NMR (203 MHz, $CDCl_3$, Figure S56) δ –29.13 (d, J = 13.6 Hz) ppm. HRMS (m/z): $[M + H]^+$ calcd for $C_8H_{19}O_3PSi_2$, 251.06831; found, 251.06796.

S5.2 Synthesis of diethyl ethynyl phosphonate 2b

A suspension of triethylammonium hydrogen ethynyl phosphonate **2** (240 mg, 1.2 mmol) in triethylorthoformate (15 mL) was refluxed under nitrogen at 148 °C for 16 h in a 50 mL flask, resulting in the dissolution of solid. After cooling, excess triethylorthoformate was vacuum distilled off at 40 °C, leaving a brown liquid residue. The residue was purified by

flash chromatography using 1:1 EtOAc/Hexane as eluent to afford **2b** as a colorless liquid (156 mg, 0.96 mmol, 83%). The compound is very prone to oligomerization, and is recommended to be stored at low temperature. This is a known compound and characterization data are consistent with reported values.[S4–S6]

^{1}H NMR (500 MHz, $CDCl_3$, Figure S57) δ 4.19 (dqd, J = 8.5, 7.0, 1.4 Hz, 4H), 2.89 (d, J = 13.3 Hz, 1H), 1.38 (t, J = 7.1 Hz, 6H) ppm. ^{13}C NMR (126 MHz, $CDCl_3$, Figure S58) δ 87.69 (d, J = 50.7 Hz), 74.58 (d, J = 288.6 Hz), 63.65 (d, J = 5.5 Hz), 16.19 (d, J = 6.9 Hz) ppm. ^{31}P NMR (203 MHz, $CDCl_3$, Figure S59) δ –8.37 (dt, J = 13.4, 8.5 Hz) ppm.

S5.3 Synthesis of vinyl phosphonate 3 from 2

A solution of triethylammonium hydrogen ethynyl phosphonate **2** (104 mg, 0.5 mmol), and Lindlar's catalyst (5 wt%, 5 mg, 0.5 mol%) in MeOH (15 mL) was prepared in a 100 mL Schlenk flask. One drop of quinoline was added. The atmosphere was replaced by H_2 via flash-pumping and refilling more than 5 times. The resulting solution was stirred for 15 min under 1 atm of H_2 using a balloon. The balloon was removed and the solution was filtered through Celite to removed Pd catalyst. The filtrate was concentrated to ca. 2 mL, then methyl *t*-butyl ether (MTBE, 6 mL) was layered on top and the mixture was placed at –25 °C overnight. The supernatant was decanted, and the residue was rinsed with MTBE (3 mL) and dried in vacuo to yield trimethylammonium hydrogen vinyl phosphonate **3** as a pale yellow viscous liquid (80 mg, 0.38 mmol, 76%).

^{1}H NMR (500 MHz, D_2O, Figure S60) δ 6.17 – 5.82 (m, 3H), 3.19 (q, J = 7.4 Hz, 6H), 1.27 (t, J = 7.3 Hz, 9H) ppm. ^{13}C NMR (126 MHz, D_2O, Figure S61) δ 130.66 (d, J = 1.7 Hz), 130.13 (d, J = 174.2 Hz), 46.62, 8.19 ppm. ^{31}P NMR (203 MHz, D_2O, Figure S62) δ 13.65 (ddd, J = 48.2, 24.4, 21.9 Hz) ppm.

S5.4 Synthesis of triphenylphosphine from 2b

S5.4.1 Synthesis of diethyl phenyl phosphonate

This compound is prepared following literature procedure:[S7] in the glovebox, a solution of diethyl ethynyl phosphonate **2b** (133 mg, 0.82 mmol) in neat 1,3-cyclohexadiene (1 mL, ca. 10 mmol) was prepared in a 25 mL thick wall Schlenk tube. The tube was brought out and heated at 120 °C for 16 h. After cooling to ambient temperature, volatile material was removed under reduced pressure. The residue was purified by column chromatography using 1:1 hexanes/ethyl acetate to afford diethyl phenyl phosphonate as a colorless liquid (132 mg, 0.62 mmol, 75%). This is a known compound and characterization data are consistent with reported values.[S8,S9]

S5.4.2 Synthesis of triphenylphosphine oxide

This compound is prepared following literature procedure:[S10] a solution of diethyl phenyl phosphonate (130 mg, 0.61 mmol, 1.0 equiv) and NaOTf (230 mg, 1.3 mmol, 2.2 equiv) in dry THF (8 mL) was prepared under N_2 in a 50 mL Schlenk tube. The solution was cool to 0 °C and PhMgBr (3 M in ether, 0.43 mL, 1.3 mmol, 2.2 equiv) was added dropwise. The tube was sealed and heated at 70 °C for 4 h. The reaction mixture was cooled to 0 °C and quenched with 0.1 M H_2SO_4 (10 mL). The aqueous phase was extracted with 5×8 mL DCM. The combined organic phase was concentrated and the residue was purified by column chromatography using ethyl acetate to afford triphenylphosphine oxide as a white solid (121 mg, 0.43 mmol, 72%). This is a known compound and characterization data are consistent with those of a commercial sample.

S5.4.3 Synthesis of triphenylphosphine

This compound is prepared following literature procedure:[S10] in the glovebox, to a solution of triphenylphosphine oxide (115 mg, 0.41 mmol) in dry toluene (3 mL) in a 25 mL thick wall reaction vessel was added DIBAL-H (25 wt% in toluene, 1.17 g, 2.0 mmol, 5 equiv). The vessel was capped with a septum pierced by a needle, and the solution was allowed to stir and vent overnight. The septum was removed and the vessel was then sealed, brought out and heated at 150 °C for 12 h. After cooling to 0 °C, the reaction was quenched with 0.1 M HCl (5 mL) and diluted with diethyl ether (5 mL). The aqueous layer was extracted with ether (3×5 mL). The combined organic phase was dried over Na_2SO_4 and evaporated to afford triphenylphosphine as a white solid (94 mg, 0.36 mmol, 89%). This is a known compound and characterization data are consistent with those of a commercial sample.

S5.5 Synthesis of 4 from 2b

This compound is prepared following reported procedure:[S11] in the glovebox, bis(tetra-*n*-butylammonium) ethynyl phosphonate **2′** (59 mg, 0.10 mmol) and P_2O_5(pyridine)$_2$ (30 mg, 0.10 mmol, 1.0 equiv) were weighed in a 20 mL scintillation vial. DCM (3 mL) was added to the mixture with rapid stirring, resulting in dissolution of most solid material. The solution was filtered and solvent was removed under reduced pressure. The residue was redissolved in DMF (4 mL). Tetra-*n*-butylammonium adenosine monophosphate (75 mg, 0.1 mmol, 1.0 equiv) was added in one portion, followed by anhydrous $MgCl_2$ (19 mg, 0.2 mmol, 2 equiv). The mixture was stirred at 23 °C for 16 h. The solution was brought out of the glovebox and dimethylformamide was removed under reduced pressure at 45 °C. The residue was redissolved in deionized water (5 mL) and

passed through an Acrodisc 0.2 µm wwPTFE syringe filter before being purified by anion exchange HPLC (AX-HPLC) using an Agilent 1260 Infinity II HPLC system comprised of a 1260 Infinity II Preparative Binary Pump coupled to a 1260 Infinity II Diode Array Detector, utilizing a PRP-X100 anion exchange column with a gradient method from 100% A, 0% B to 0% A, 100% B (A = 100% water; B = aqueous 1 M ammonium bicarbonate) with a 6 mL/min flow rate. Compound **4** was obtained as the ammonium salt as a colorless solid after lyophilization (32 mg, 0.048 mmol, 48%). This is a known compound and characterization data are consistent with reported values.[S11]

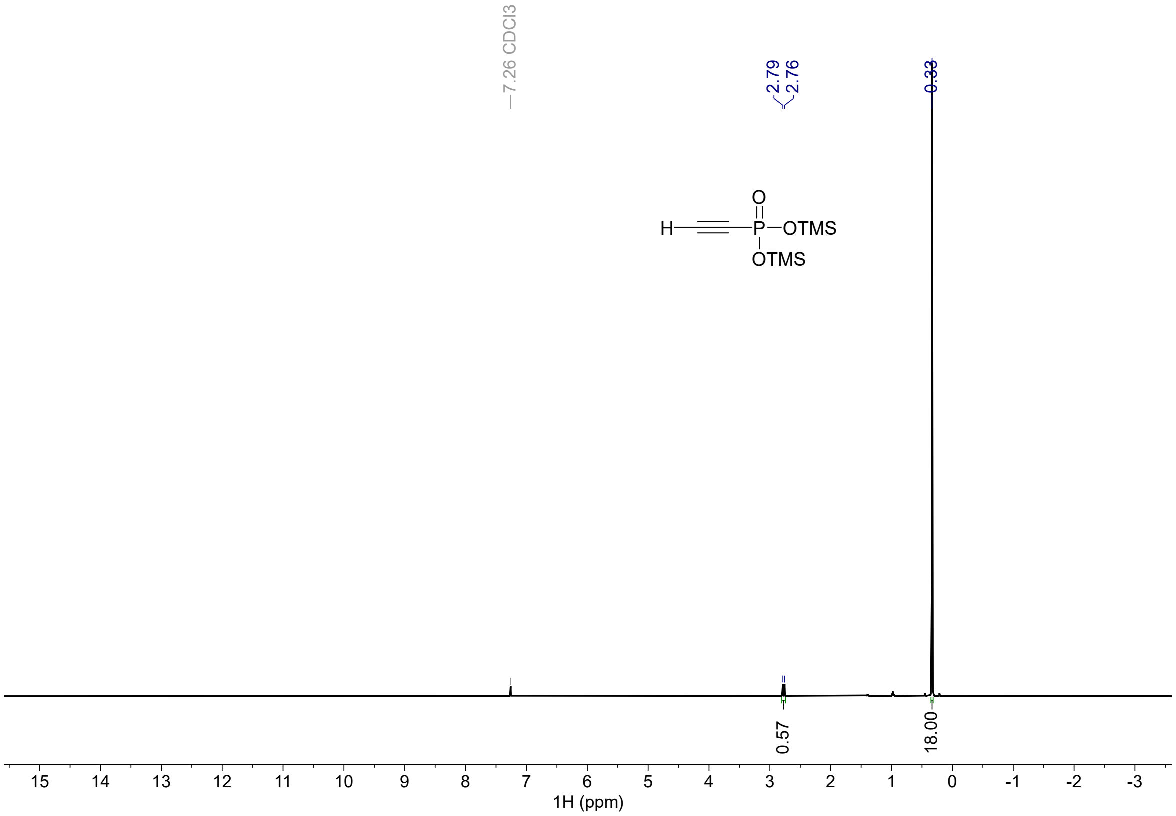

Figure S54: ^{1}H NMR spectrum of **2a** in $CDCl_3$ at 25 °C, recorded at 500 MHz.

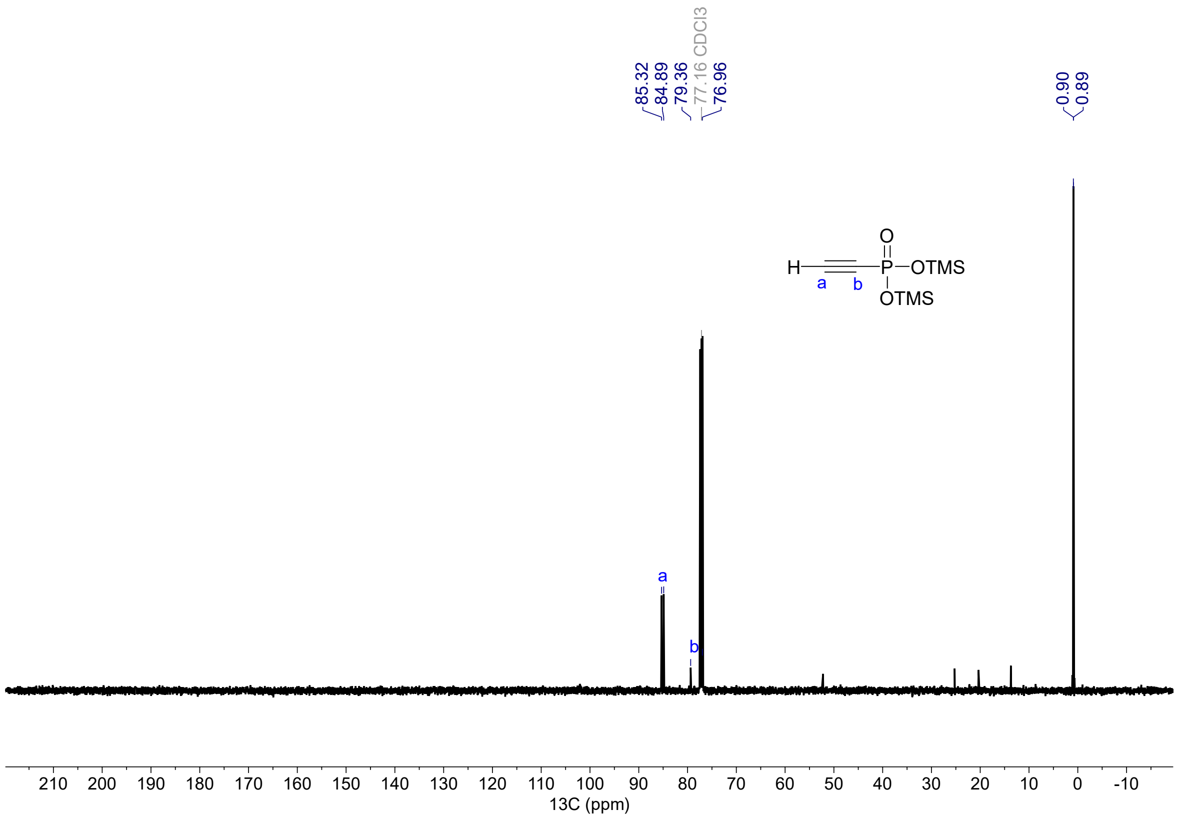

Figure S55: ^{13}C NMR spectrum of **2a** in $CDCl_3$ at 25 °C, recorded at 126 MHz.

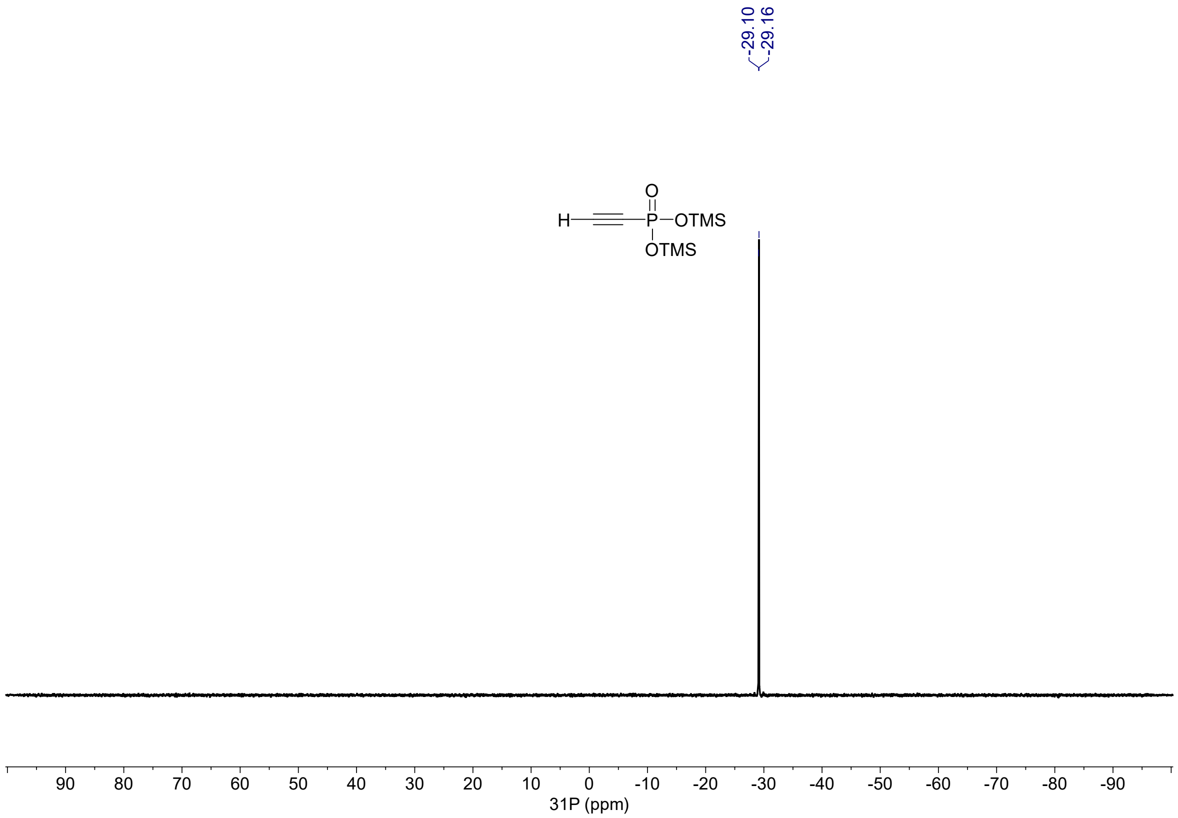

Figure S56: ^{31}P NMR spectrum of **2a** in $CDCl_3$ at 25 °C, recorded at 203 MHz.

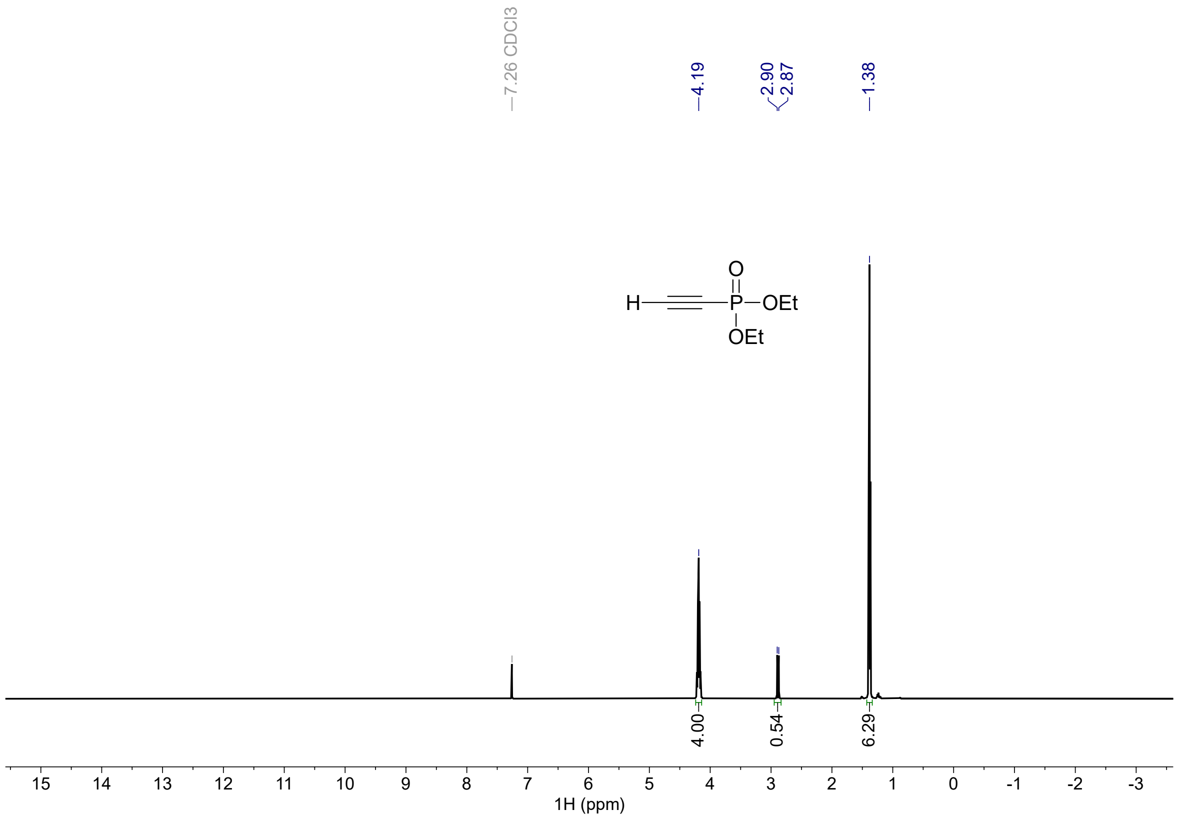

Figure S57: ^{1}H NMR spectrum of **2b** in $CDCl_3$ at 25 °C, recorded at 500 MHz.

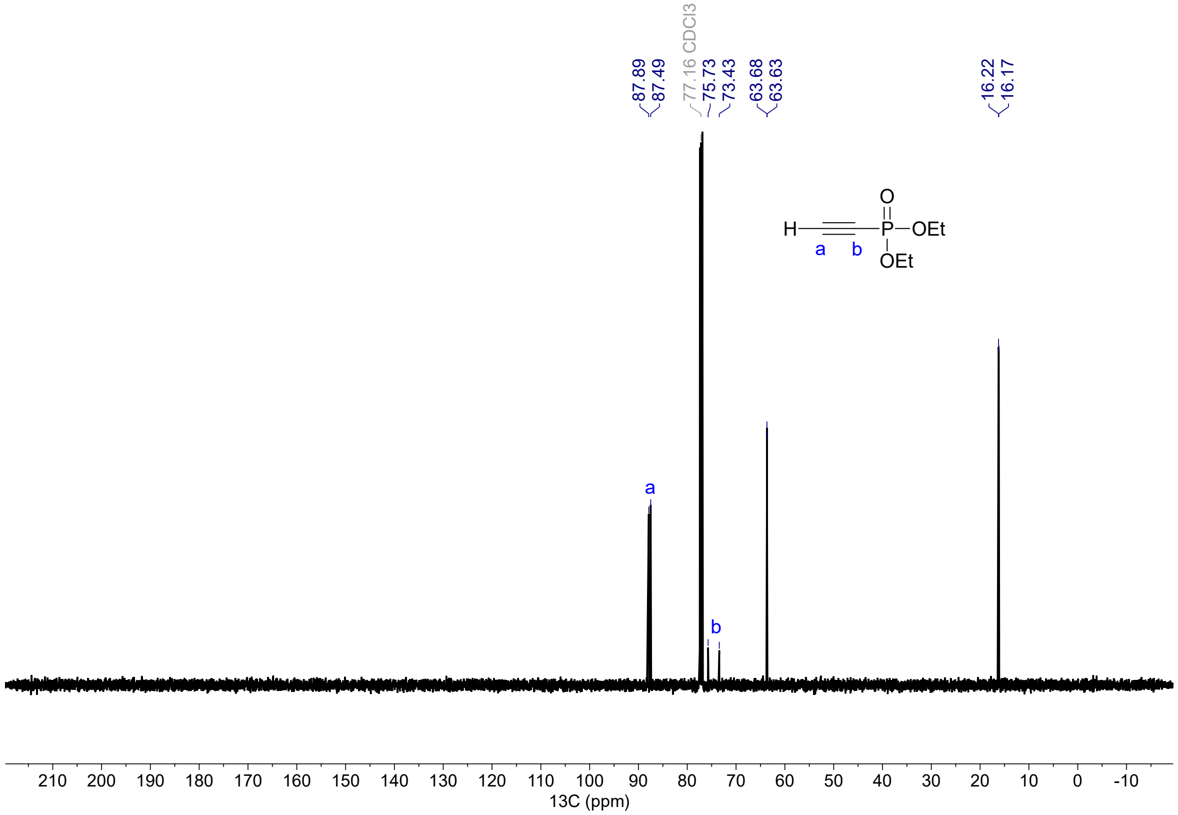

Figure S58: ^{13}C NMR spectrum of **2b** in $CDCl_3$ at 25 °C, recorded at 126 MHz.

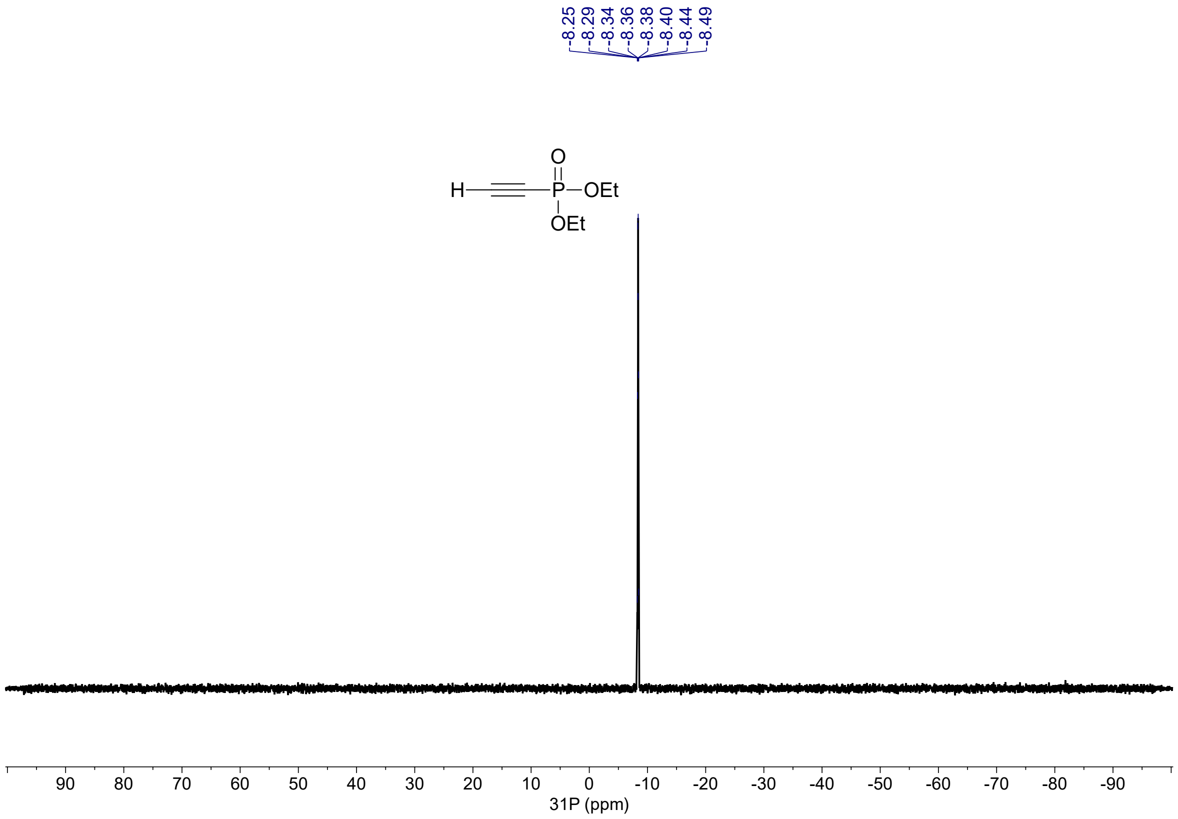

Figure S59: ^{31}P NMR spectrum of **2b** in $CDCl_3$ at 25 °C, recorded at 203 MHz.

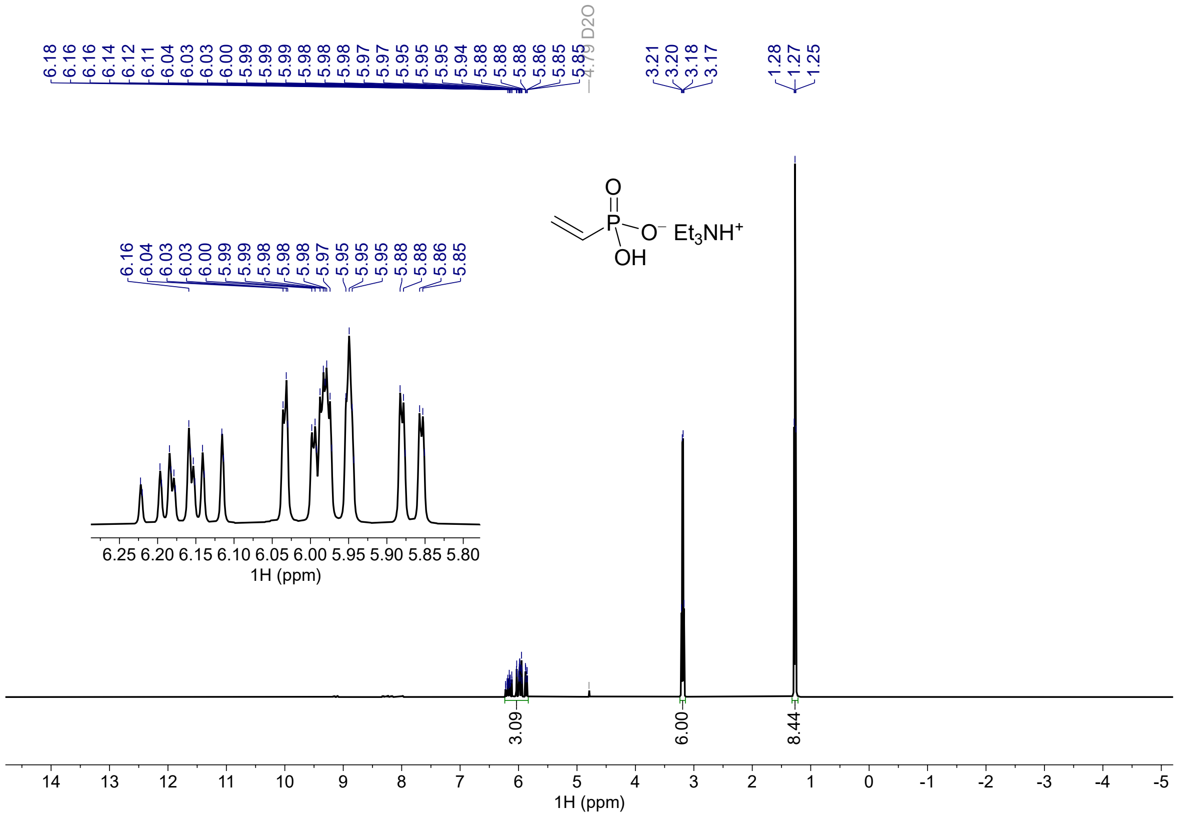

Figure S60: ^{1}H NMR spectrum of **3** in D_2O at 25 °C, recorded at 500 MHz.

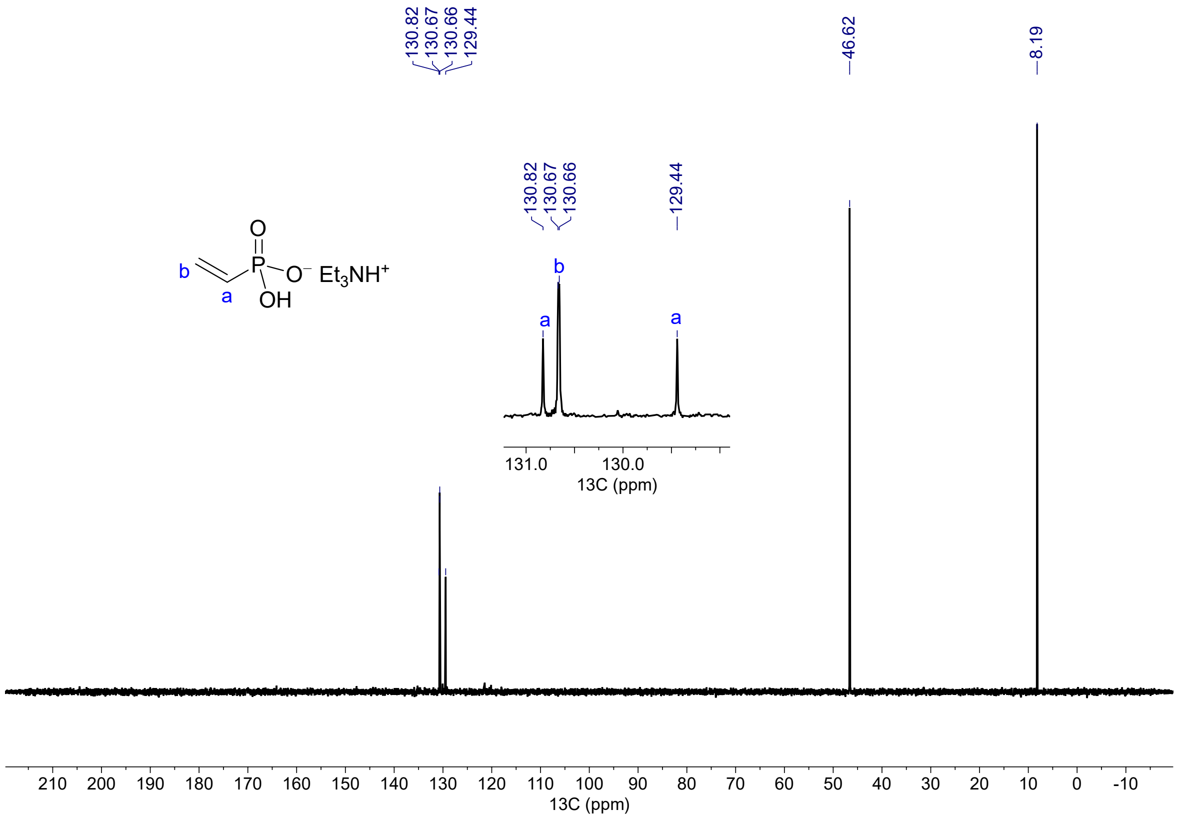

Figure S61: ^{13}C NMR spectrum of **3** in D_2O at 25 °C, recorded at 126 MHz.

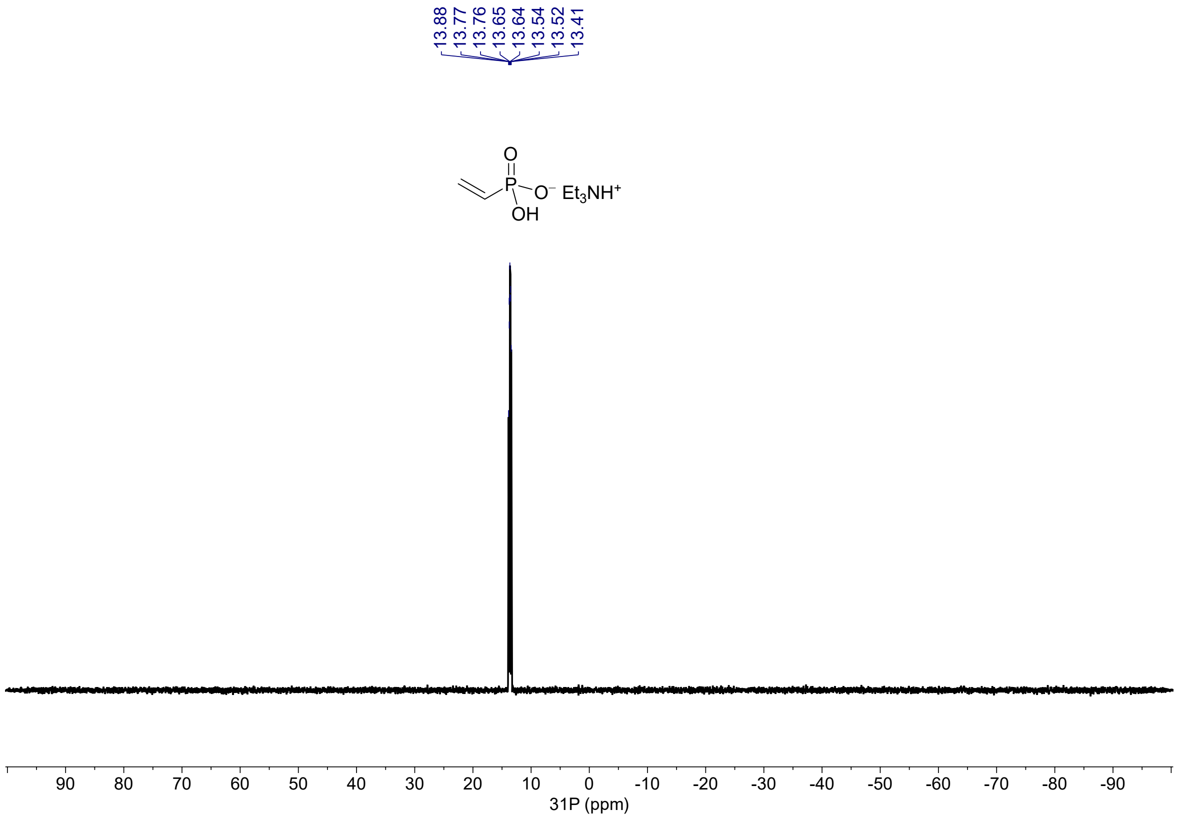

Figure S62: ^{31}P NMR spectrum of **3** in D_2O at 25 °C, recorded at 203 MHz.

S6 Synthesis of alkynylphosphonic acids from 2a

S6.1 General procedure

In the glovebox, a solution of aryl iodide (0.3 mmol, 1.0 equiv) and diisopropylethylamine (43 mg, 0.33 mmol, 1.1 equiv) in THF (5 mL) was added to a 20 mL scintillation vial charged with bis(trimethylsilyl) ethynyl phosphonate **2a** (75 mg, 0.3 mmol, 1.0 equiv), $Pd(PPh_3)_2Cl_2$ (7 mg, 3 mol%), CuI (6 mg, 10 mol%) and a stir bar. The resulting solution was stirred at 23 °C for 16 h. Volatile material was removed under reduced pressure and the brown residue was extracted with hexanes (3×4 mL). Volatile material was removed from the combined extract to afford the bis(trimethylsilyl)phosphonate **1′**. The obtained **1′** was brought out of the glovebox and dissolved in methanol (5 mL). Volatile material was removed under reduced pressure to yield the corresponding phosphonic acid **1**.

S6.2 Synthesis of bis(trimethylsilyl) alkynyl phosphonates 1m–s′

S6.2.1 Bis(trimethylsilyl) ((4-(methoxycarbonyl)phenyl)ethynyl)phosphonate 1m′

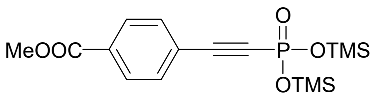

Following the general procedure, the reaction of methyl 4-iodobenzoate (79 mg, 0.3 mmol) and **2a** (75 mg, 0.3 mmol) afforded **1m′** as a white solid (83 mg, 0.22 mol, 72%).

^{1}H NMR (400 MHz, $CDCl_3$, Figure S63) δ 8.03 (d, J = 8.4 Hz, 2H), 7.58 (d, J = 8.3 Hz, 2H), 3.93 (s, 3H), 0.37 (s, 18H) ppm. ^{13}C NMR (126 MHz, $CDCl_3$, Figure S64)

δ 165.99, 137.62, 132.11 (d, J = 2.5 Hz), 129.50, 124.57 (d, J = 5.9 Hz), 94.72 (d, J = 55.7 Hz), 84.76 (d, J = 307.4 Hz), 52.32, 0.74 (d, J = 1.8 Hz) ppm. ^{31}P NMR (162 MHz, $CDCl_3$, Figure S65) δ –27.60 (s) ppm. HRMS (m/z): $[M + H]^+$ calcd for $C_{16}H_{25}O_5PSi_2$, 385.10619; found, 385.10655.

S6.2.2 Bis(trimethylsilyl) ((4-nitrophenyl)ethynyl)phosphonate 1n′

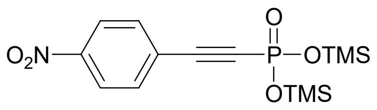

Following the general procedure, the reaction of 4-nitro-iodobenzene (75 mg, 0.3 mmol) and **2a** (75 mg, 0.3 mmol) afforded **1n′** as a pale yellow solid (86 mg, 0.23 mol, 77%).

^{1}H NMR (500 MHz, $CDCl_3$, Figure S69) δ 8.24 (d, J = 8.9 Hz, 2H), 7.69 (d, J = 8.7 Hz, 2H), 0.37 (s, 18H) ppm. ^{13}C NMR (126 MHz, $CDCl_3$, Figure S70) δ 148.47, 133.33 (d, J = 2.5 Hz), 126.99 (d, J = 5.9 Hz), 123.91, 93.18 (d, J = 55.2 Hz), 87.12 (d, J = 304.8 Hz), 1.01 (d, J = 1.8 Hz) ppm. ^{31}P NMR (203 MHz, $CDCl_3$, Figure S71) δ –28.19 (s) ppm. HRMS (m/z): $[M + H]^+$ calcd for $C_{14}H_{22}NO_5PSi_2$, 372.08739; found, 372.08579.

S6.2.3 Bis(trimethylsily) ((4-formylphenyl)ethynyl)phosphonate 1o′

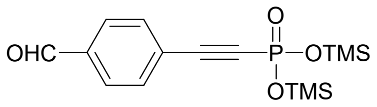

Following the general procedure, the reaction of 4-iodobenzaldehyde (70 mg, 0.3 mmol) and **2a** (75 mg, 0.3 mmol) afforded **1o′** as an off-white crystalline solid (84 mg, 0.24 mol, 79%).

^{1}H NMR (500 MHz, $CDCl_3$, Figure S75) δ 10.04 (s, 1H), 7.88 (d, J = 8.3 Hz, 2H), 7.68 (d, J = 8.1 Hz, 2H), 0.37 (s, 18H) ppm. ^{13}C NMR (126 MHz, $CDCl_3$, Figure S76)

δ 191.13, 136.85, 132.86 (d, J = 2.6 Hz), 129.56, 126.15 (d, J = 5.9 Hz), 94.41 (d, J = 55.6 Hz), 85.74 (d, J = 306.4 Hz), 0.86 (d, J = 1.7 Hz) ppm. ^{31}P NMR (203 MHz, $CDCl_3$, Figure S77) δ –27.81 (s) ppm. HRMS (m/z): $[M + H]^+$ calcd for $C_{15}H_{23}O_4PSi_2$, 355.09562; found, 355.09708.

S6.2.4 Tetrakis(trimethylsilyl) (1,4-phenylenebis(ethyne-2,1-diyl))bis(phosphonate) 1p′

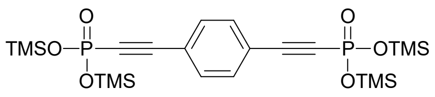

Following the general procedure, the reaction of 1,4-diiodobenzene (50 mg, 0.15 mmol) and **2a** (75 mg, 0.3 mmol) afforded **1p′** as a white solid (65 mg, 0.11 mol, 75%).

^{1}H NMR (500 MHz, $CDCl_3$, Figure S81) δ 7.51 (s, 4H), 0.36 (s, 36H) ppm. ^{13}C NMR (126 MHz, $CDCl_3$, Figure S82) δ 132.18 (d, J = 2.6 Hz), 121.92 (d, J = 5.9 Hz), 94.63 (d, J = 55.9 Hz), 84.83 (d, J = 307.5 Hz), 0.73 (d, J = 1.8 Hz) ppm. ^{31}P NMR (203 MHz, $CDCl_3$, Figure S83) δ –27.61 (s) ppm. HRMS (m/z): $[M + H]^+$ calcd for $C_{22}H_{40}O_6P_2Si_4$, 575.14500; found, 575.15428.

S6.2.5 Tetrakis(trimethylsilyl) (1,3-phenylenebis(ethyne-2,1-diyl))bis(phosphonate) 1q′

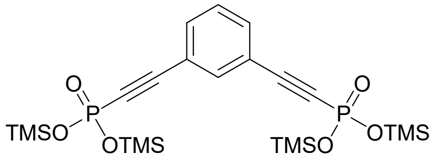

Following the general procedure, the reaction of 1,3-diiodobenzene (50 mg, 0.15 mmol) and **2a** (75 mg, 0.3 mmol) afforded **1q′** as a pale yellow liquid (68 mg, 0.12 mol, 79%).

^{1}H NMR (500 MHz, $CDCl_3$, Figure S87) δ 7.65 (s, 1H), 7.56 (dd, J = 7.9, 1.8 Hz, 2H), 7.38 (t, J = 7.8 Hz, 1H), 0.37 (s, 36H) ppm. ^{13}C NMR (126 MHz, $CDCl_3$, Figure S88) δ 135.81 (t, J = 2.6 Hz), 133.85 (d, J = 2.5 Hz), 129.12, 121.20 (d, J = 6.0 Hz), 94.38 (d, J = 55.9 Hz), 83.73 (d, J = 307.5 Hz), 0.99 (d, J = 1.8 Hz) ppm. ^{31}P NMR (203 MHz, $CDCl_3$, Figure S89) δ –27.64 (s) ppm. HRMS (m/z): $[M + H]^+$ calcd for $C_{22}H_{40}O_6P_2Si_4$, 575.14500; found, 575.15529.

S6.2.6 Tetrakis(trimethylsilyl) ([1,1'-biphenyl]-4,4'-diylbis(ethyne-2,1-diyl))-bis(phosphonate) 1r′

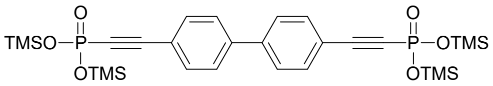

Following the general procedure, the reaction of 4,4'-diiodobiphenyl (61 mg, 0.15 mmol) and **2a** (75 mg, 0.3 mmol) afforded **1r′** as an off-white solid (77 mg, 0.12 mol, 79%).

^{1}H NMR (500 MHz, $CDCl_3$, Figure S93) δ 7.61 (d, J = 8.5 Hz, 4H), 7.58 (d, J = 8.7 Hz, 4H), 0.38 (s, 36H) ppm. ^{13}C NMR (126 MHz, $CDCl_3$, Figure S94) δ 141.52, 132.79 (d, J = 2.6 Hz), 127.09, 119.75 (d, J = 5.9 Hz), 95.70 (d, J = 56.1 Hz), 83.30 (d, J = 309.4 Hz), 0.75 (d, J = 1.6 Hz) ppm. ^{31}P NMR (203 MHz, $CDCl_3$, Figure S95) δ –27.13 (s) ppm. HRMS (m/z): $[M + H]^+$ calcd for $C_{28}H_{44}O_6P_2Si_4$, 651.17630; found, 651.18866.

S6.2.7 Hexakis(trimethylsilyl) [benzene-1,3,5-triyltris(4,1-phenyleneethyne-2,1-diyl)]tris(phosphonate) 1s′

Following the general procedure, the reaction of 1,3,5-tris(4-iodophenyl)benzene (68 mg, 0.10 mmol) and **2a** (75 mg, 0.3 mmol) afforded **1s′** as an off-white solid (47 mg, 0.045 mol, 45%).

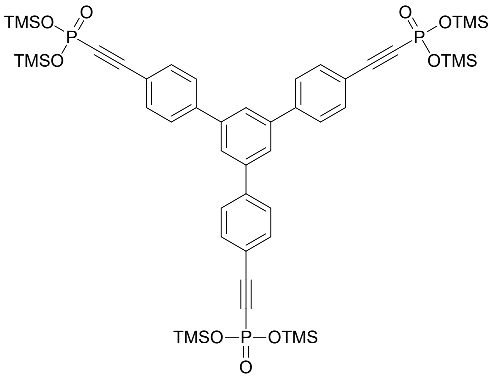

^{1}H NMR (500 MHz, $CDCl_3$, Figure S99) δ 7.77 (s, 3H), 7.68 (d, J = 8.5 Hz, 6H), 7.65 (d, J = 8.2 Hz, 6H), 0.39 (s, 54H) ppm. ^{13}C NMR (126 MHz, $CDCl_3$, Figure S100) δ 142.49, 141.73, 133.07 (d, J = 2.5 Hz), 127.56, 125.82, 119.85 (d, J = 5.9 Hz), 96.04 (d, J = 56.7 Hz), 83.47 (d, J = 309.8 Hz), 1.02 (d, J = 1.7 Hz) ppm. ^{31}P NMR (203 MHz, $CDCl_3$, Figure S101) δ –27.06 (s) ppm.

S6.2.8 Bis(trimethylsilyl) ((4-(tetramethyldioxaborolanyl)phenyl)ethynyl)phosphonate 1t′

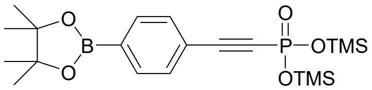

Following the general procedure, the reaction of 4-iodophenylboronic acid pinacol ester (99 mg, 0.3 mmol) and **2a** (75 mg, 0.3 mmol) afforded **1t′** as a white solid (89 mg, 0.20 mol, 66%).

^{1}H NMR (500 MHz, $CDCl_3$, Figure S105) δ 7.78 (d, J = 8.1 Hz, 2H), 7.50 (d, J = 8.1 Hz, 2H), 1.34 (s, 12H), 0.36 (s, 18H) ppm. ^{13}C NMR (126 MHz, $CDCl_3$, Figure S106) δ 134.80, 131.49 (d, J = 2.5 Hz), 122.82 (d, J = 5.6 Hz), 96.40 (d, J = 56.2 Hz), 84.32, 83.48 (d, J = 309.6 Hz), 25.01, 0.98 (d, J = 1.7 Hz) ppm. ^{31}P NMR (203 MHz, $CDCl_3$, Figure S107) δ –27.10 (s) ppm. HRMS (m/z): $[M + H]^+$ calcd for $C_{20}H_{34}BO_5PSi_2$, 453.18482;

found, 453.19506.

S6.3 Syntheis of alkynylphosphonic acids 1m–s

S6.3.1 ((4-(methoxycarbonyl)phenyl)ethynyl)phosphonic acid 1m

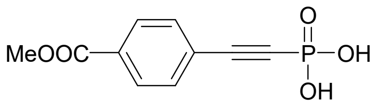

Following the general procedure, methanolysis of **1m′** yielded **1m** as a colorless solid (52 mg, quant.)

^{1}H NMR (500 MHz, D_2O, Figure S66) δ 7.81 (d, J = 8.5 Hz, 2H), 7.59 (d, J = 8.3 Hz, 2H), 3.34 (s, 3H) ppm. ^{13}C NMR (126 MHz, D_2O, Figure S67) δ 174.96, 136.31, 131.70 (d, J = 2.0 Hz), 128.71, 124.76 (d, J = 4.4 Hz), 91.48 (d, J = 231.2 Hz), 90.18 (d, J = 41.3 Hz), 48.85 ppm. ^{31}P NMR (203 MHz, D_2O, Figure S68) δ –9.17 (s) ppm. ESI-MS(–) (m/z): $[M - H]^-$ calcd for $C_{10}H_9O_5P$, 239.0115; found, 239.0113.

S6.3.2 ((4-nitrophenyl)ethynyl)phosphonic acid 1n

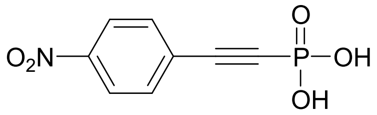

Following the general procedure, methanolysis of **1n′** yielded **1n** as a pale yellow solid (53 mg, quant.)

^{1}H NMR (500 MHz, D_2O, Figure S72) δ 8.23 (d, J = 8.1 Hz, 2H), 7.74 (d, J = 8.2 Hz, 2H) ppm. ^{13}C NMR (126 MHz, D_2O, Figure S73) δ 146.98, 132.78 (d, J = 2.2 Hz), 129.54 (d, J = 4.5 Hz), 123.58, 94.94 (d, J = 227.9 Hz), 88.72 (d, J = 40.5 Hz) ppm. ^{31}P NMR (203 MHz, D_2O, Figure S74) δ –9.57 (s) ppm. ESI-MS(–) (m/z): $[M - H]^-$ calcd for $C_8H_6NO_5P$, 225.9911; found, 225.9911.

S6.3.3 ((4-formylphenyl)ethynyl)phosphonic acid 1o

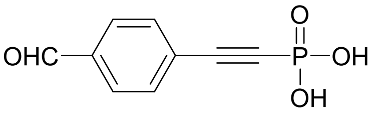

Following the general procedure, methanolysis of **1o′** yielded **1o** as a white solid (50 mg, quant.)

^{1}H NMR (500 MHz, D_2O, Figure S78) δ 9.94 (s, 1H), 7.93 (d, J = 8.3 Hz, 2H), 7.75 (d, J = 8.2 Hz, 2H) ppm. ^{13}C NMR (126 MHz, D_2O, Figure S79) δ 195.59, 135.39, 132.58 (d, J = 2.1 Hz), 129.85, 128.40 (d, J = 4.6 Hz), 91.69 (d, J = 240.5 Hz), 90.73 (d, J = 42.9 Hz) ppm. ^{31}P NMR (203 MHz, D_2O, Figure S80) δ –9.98 (s) ppm. ESI-MS(–) (m/z): $[M - H]^-$ calcd for $C_9H_7O_4P$, 209.0009; found, 209.0046.

S6.3.4 1,4-phenylenebis(ethyne-2,1-diyl))bis(phosphonic acid) 1p

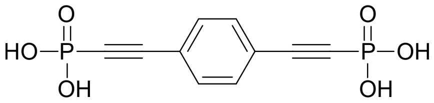

Following the general procedure, methanolysis of **1p′** yielded **1p** as a colorless viscous liquid (32 mg, quant.).

^{1}H NMR (500 MHz, D_2O, Figure S84) δ 7.53 (s, 4H) ppm. ^{13}C NMR (126 MHz, D_2O, Figure S85) δ 131.83 (d, J = 2.0 Hz), 122.44 (d, J = 4.6 Hz), 91.22 (d, J = 233.9 Hz), 90.36 (d, J = 41.7 Hz) ppm. ^{31}P NMR (203 MHz, D_2O, Figure S86) δ –9.26 (s) ppm. ESI-MS(–) (m/z): $[M - H]^-$ calcd for $C_{10}H_8O_6P_2$, 284.9723; found, 284.9881.

S6.3.5 1,3-phenylenebis(ethyne-2,1-diyl))bis(phosphonic acid) 1q

Following the general procedure, methanolysis of **1p′** yielded **1p** as a pale yellow viscous liquid (34 mg, quant.).

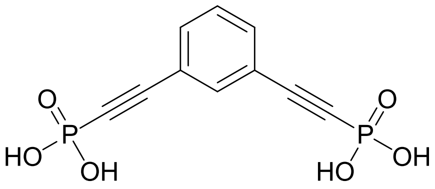

^{1}H NMR (500 MHz, D_2O, Figure S90) δ 7.70 (d, J = 1.7 Hz, 1H), 7.55 (dd, J = 7.8, 1.7 Hz, 2H), 7.37 (t, J = 7.8 Hz, 1H) ppm. ^{13}C NMR (126 MHz, D_2O, Figure S91) δ 134.74, 132.24 (d, J = 2.1 Hz), 128.63, 122.47 (d, J = 4.5 Hz), 90.32 (d, J = 231.7 Hz), 89.65 (d, J = 41.3 Hz) ppm. ^{31}P NMR (203 MHz, D_2O, Figure S92) δ –9.18 (s) ESI-MS(–) (m/z): [M – H]$^-$ calcd for $C_{10}H_8O_6P_2$, 284.9723; found, 284.9903.

S6.3.6 ([1,1'-biphenyl]-4,4'-diylbis(ethyne-2,1-diyl))bis(phosphonic acid) 1r

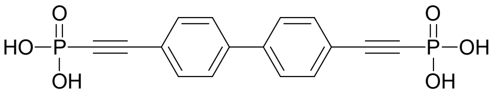

Following the general procedure, methanolysis of **1r**′ yielded **1r** as a pale yellow viscous liquid (45 mg, quant.).

^{1}H NMR (500 MHz, D_2O, Figure S96) δ 7.72 (d, J = 8.5 Hz, 4H), 7.66 (d, J = 8.4 Hz, 4H) ppm. ^{13}C NMR (126 MHz, D_2O, Figure S97) δ 140.00, 132.49 (d, J = 2.1 Hz), 126.85, 121.60 (d, J = 4.5 Hz), 90.72 (d, J = 232.6 Hz), 90.39 (d, J = 41.5 Hz) ppm. ^{31}P NMR (203 MHz, D_2O, Figure S98) δ –9.05 (s) ppm. ESI-MS(–) (m/z): [M – H]$^-$ calcd for $C_{16}H_{12}O_6P_2$, 361.0036; found, 361.0072.

S6.3.7 ([benzene-1,3,5-triyltris(4,1-phenyleneethyne-2,1-diyl)]tris(phosphonic acid) 1s

Following the general procedure, methanolysis of **1s**′ yielded **1s** as a yellow solid (27 mg, quant.).

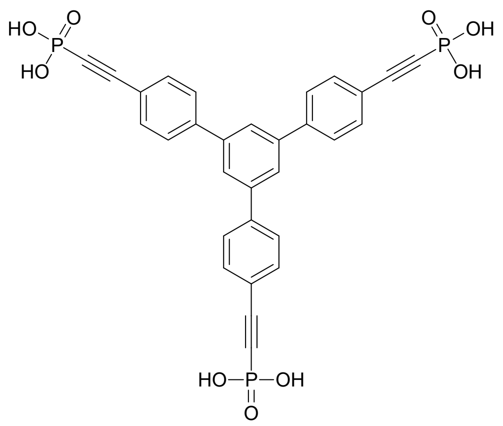

^{1}H NMR (500 MHz, D_2O, Figure S102) δ 7.68 (s, 3H), 7.61 (d, J = 8.3 Hz, 6H), 7.58 (d, J = 8.5 Hz, 6H) ppm. ^{13}C NMR (126 MHz, D_2O, Figure S103) δ 140.72, 140.59, 132.55, 126.90, 124.63, 120.47 (d, J = 3.2 Hz), 92.66 (d, J = 45.0 Hz), 87.44 (d, J = 251.5 Hz) ppm. ^{31}P NMR (203 MHz, D_2O, Figure S104) δ –9.81 (s) ppm. ESI-MS(–) (m/z): $[M - H]^-$ calcd for $C_{30}H_{21}O_9P_3$, 617.0326; found, 617.0353.

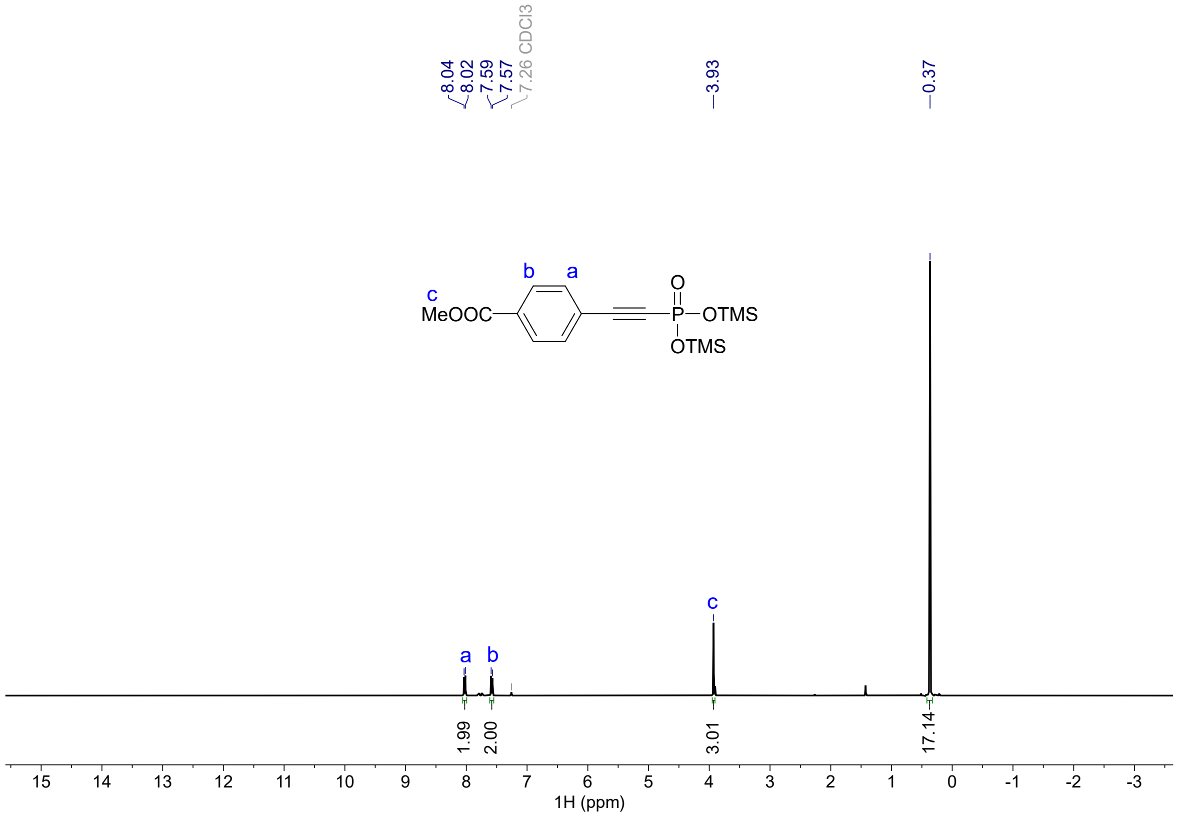

Figure S63: ^{1}H NMR spectrum of **1m′** in $CDCl_3$ at 25 °C, recorded at 400 MHz.

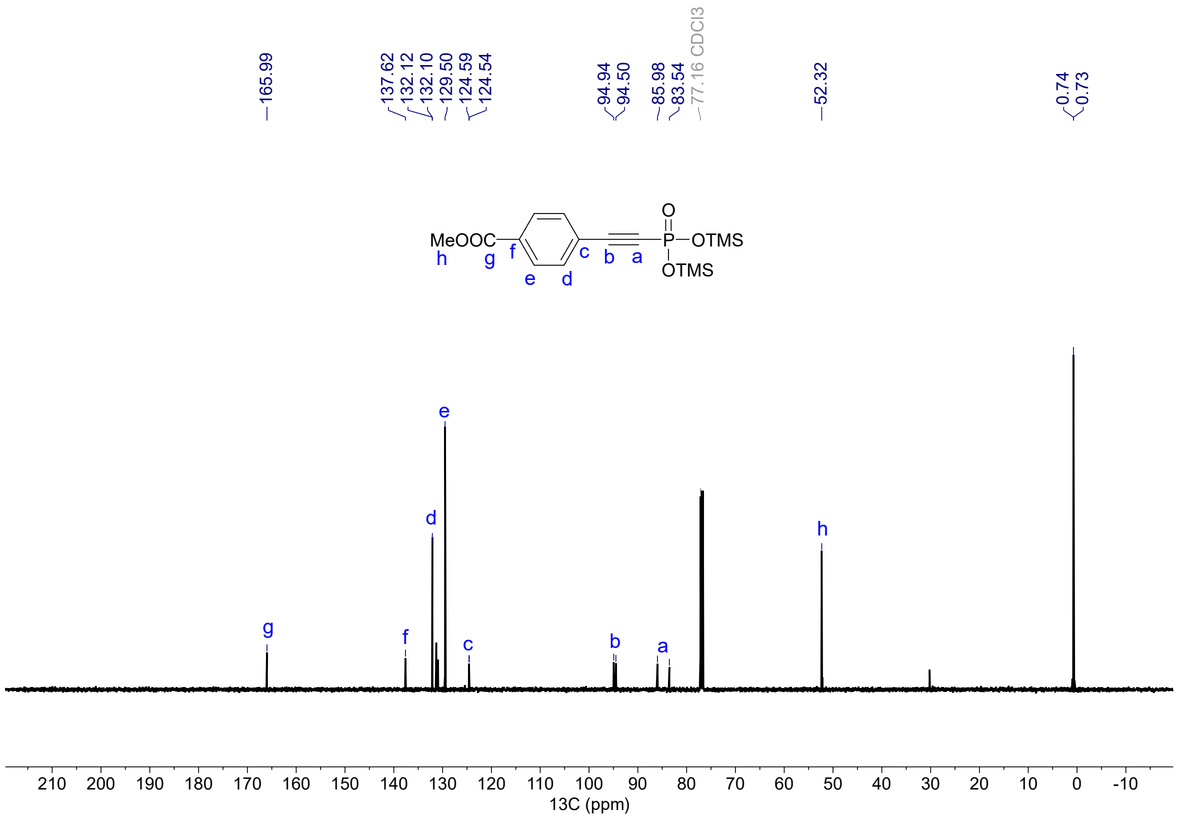

Figure S64: ^{13}C NMR spectrum of **1m′** in $CDCl_3$ at 25 °C, recorded at 126 MHz.

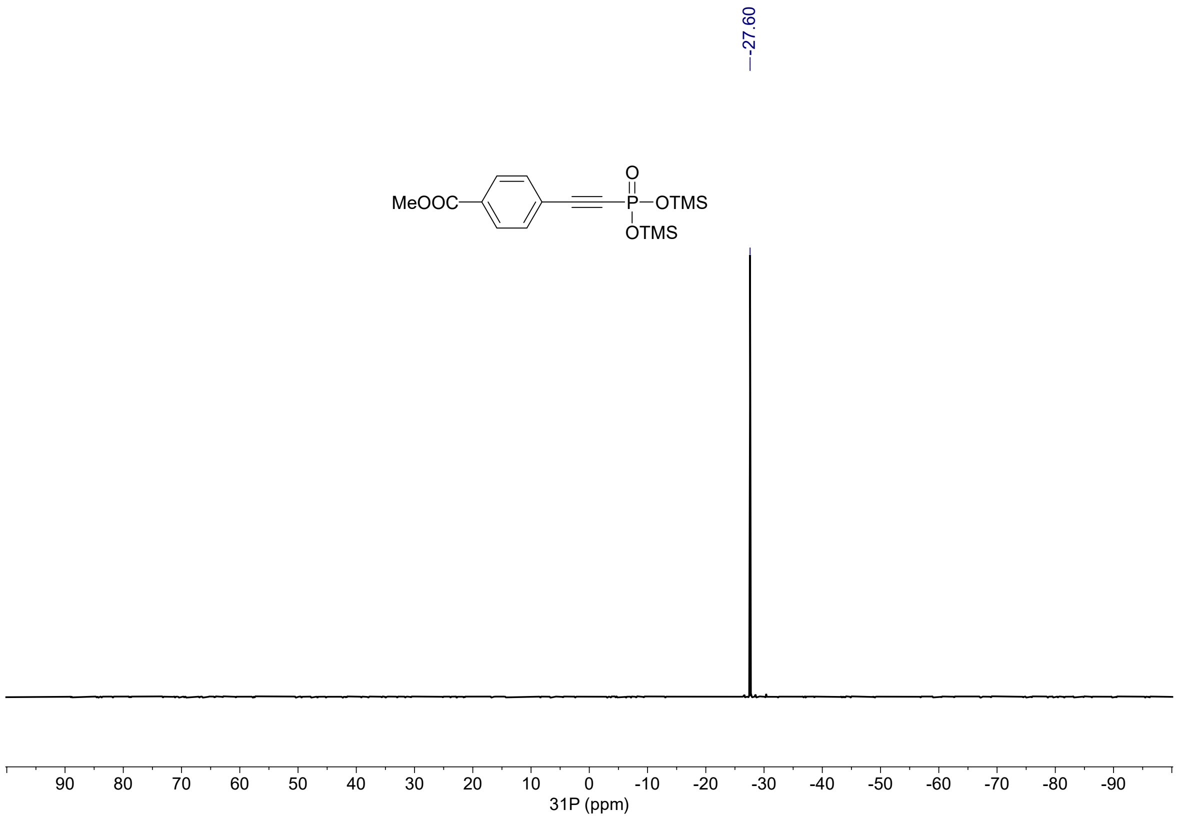

Figure S65: ^{31}P NMR spectrum of **1m′** in $CDCl_3$ at 25 °C, recorded at 162 MHz.

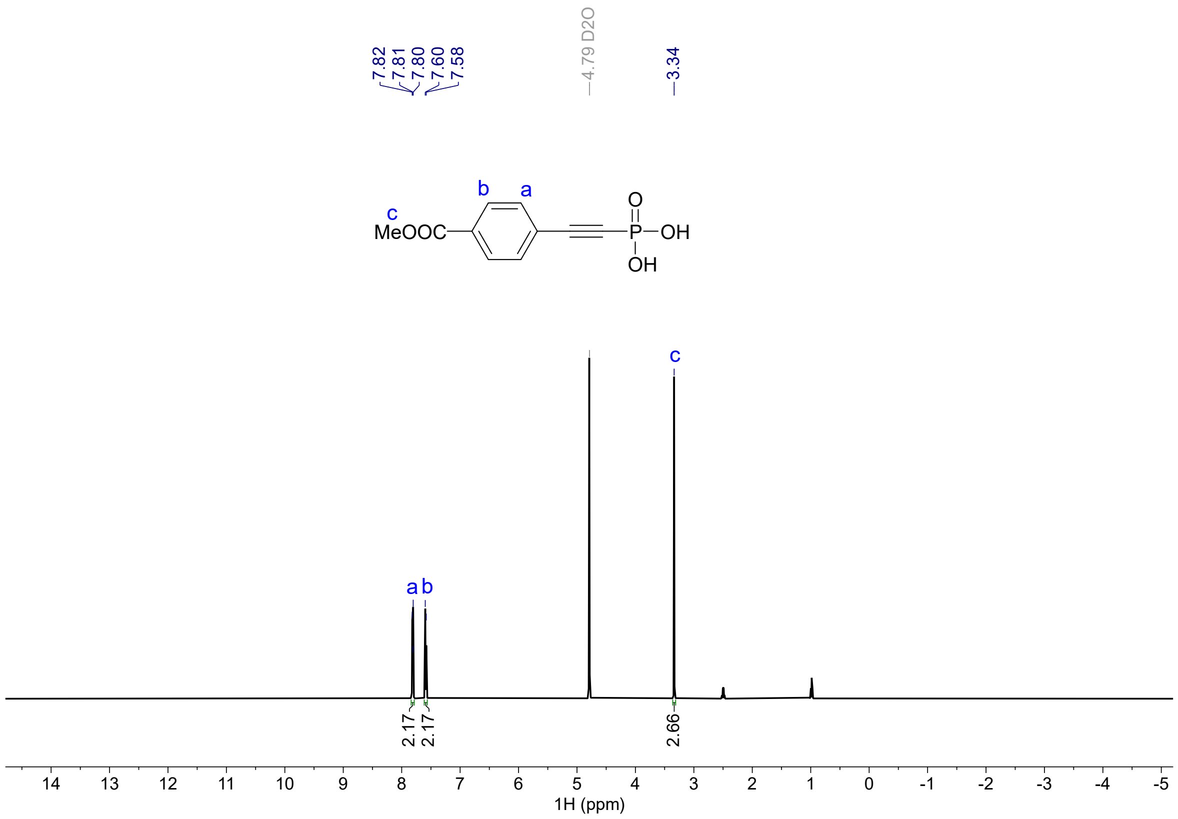

Figure S66: ^{1}H NMR spectrum of **1m** in D_2O at 25 °C, recorded at 500 MHz.

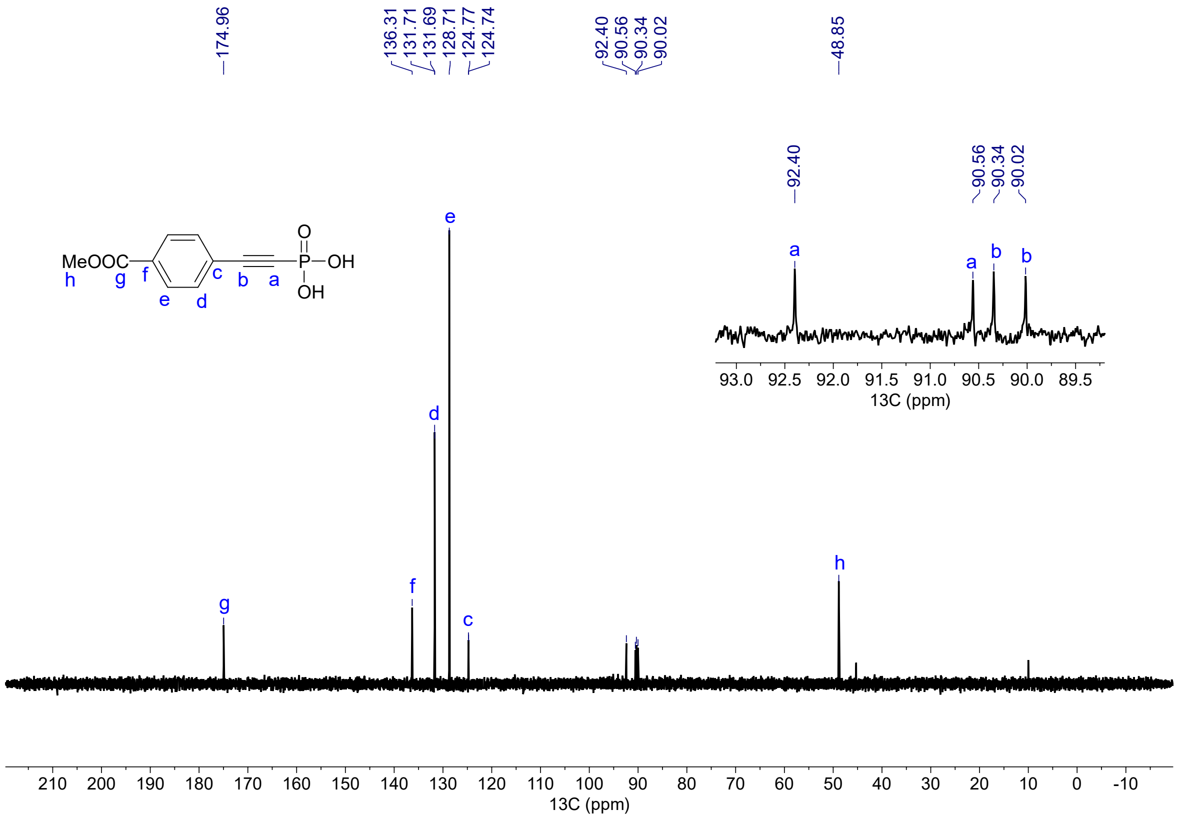

Figure S67: ^{13}C NMR spectrum of **1m** in D_2O at 25 °C, recorded at 126 MHz.

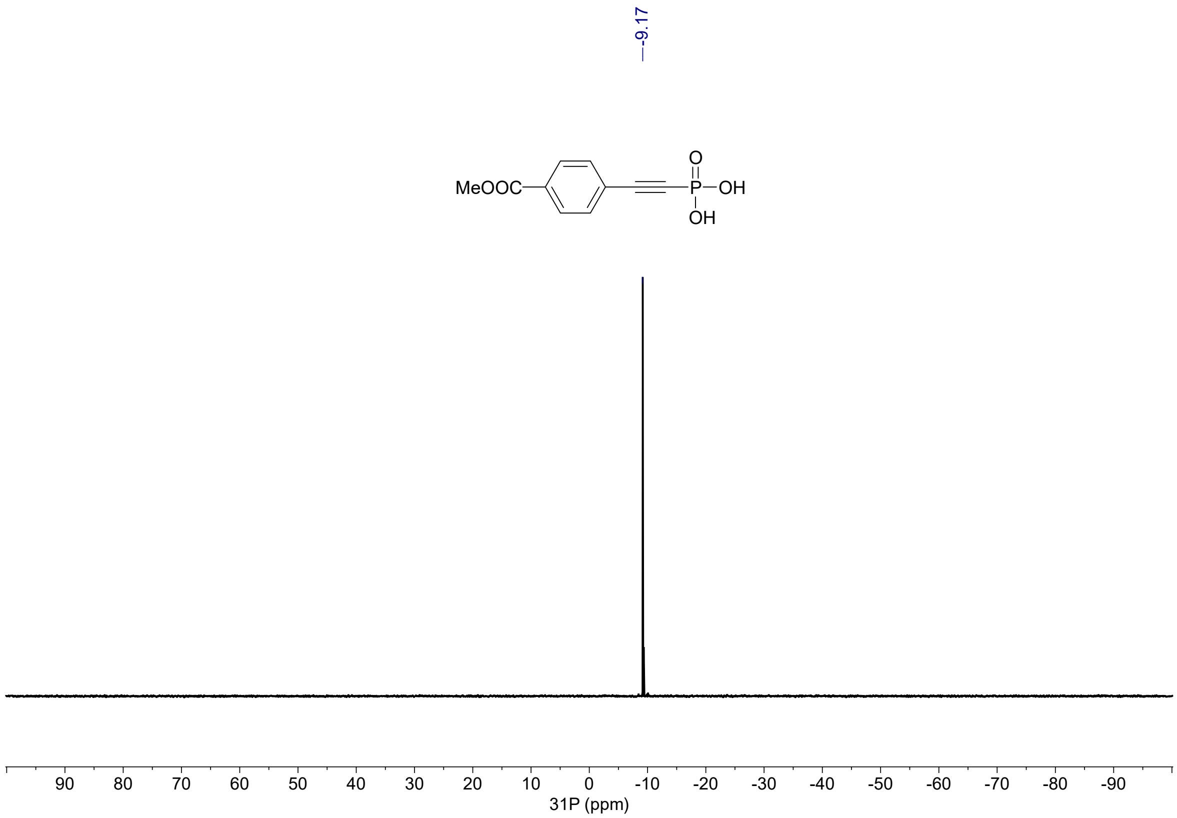

Figure S68: ^{31}P NMR spectrum of **1m** in D_2O at 25 °C, recorded at 203 MHz.

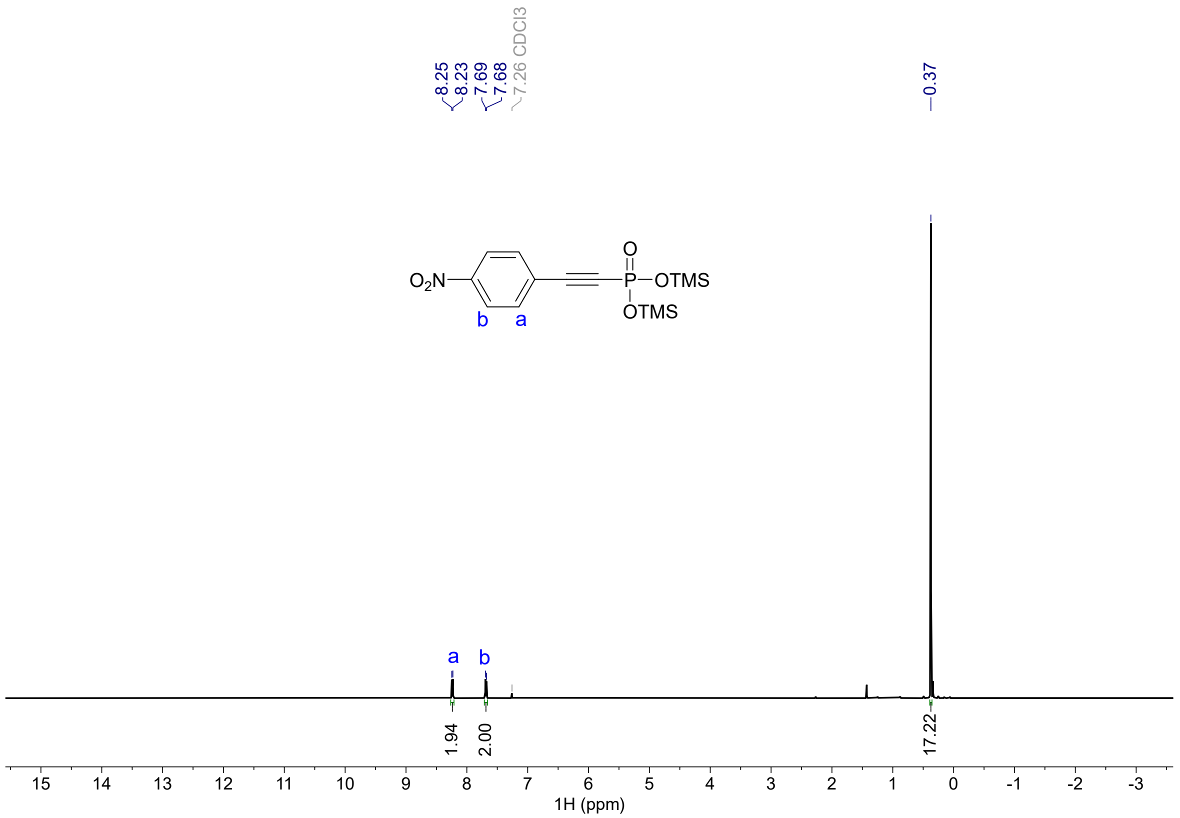

Figure S69: ^{1}H NMR spectrum of **1n′** in $CDCl_3$ at 25 °C, recorded at 500 MHz.

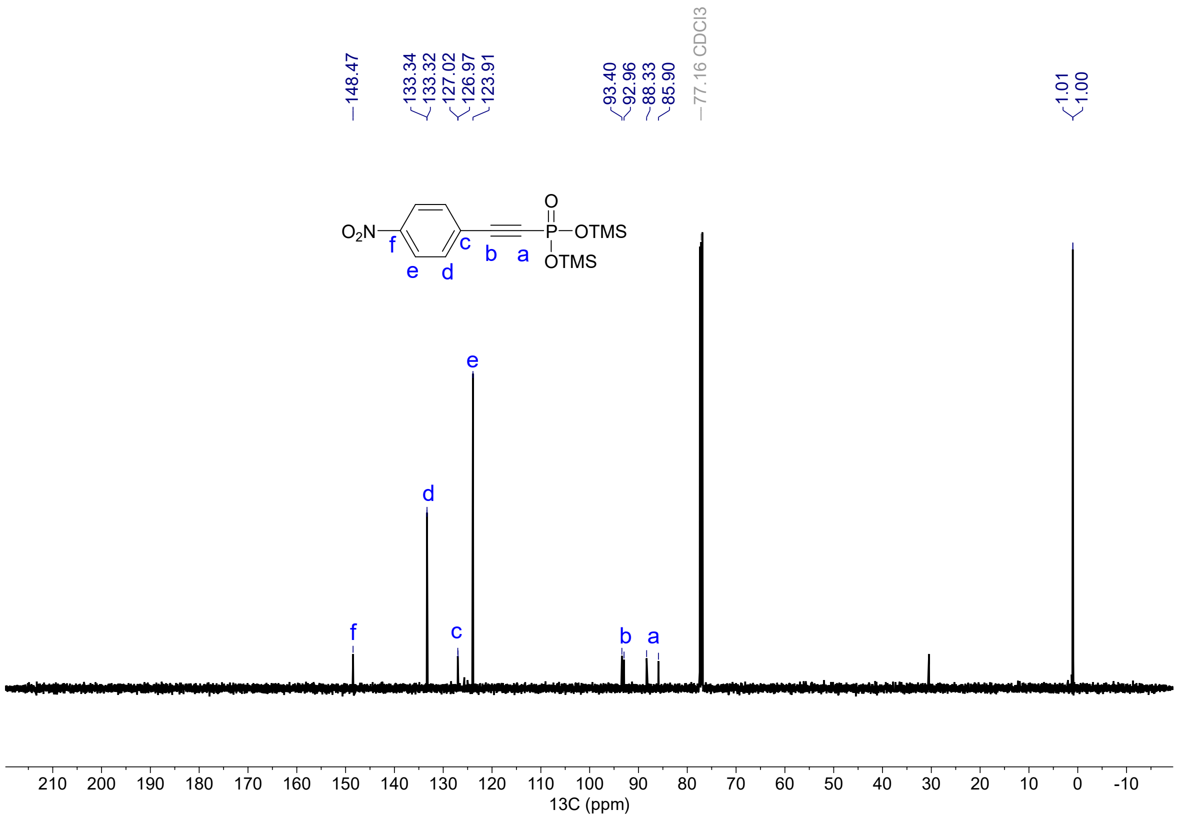

Figure S70: ^{13}C NMR spectrum of **1n′** in $CDCl_3$ at 25 °C, recorded at 126 MHz.

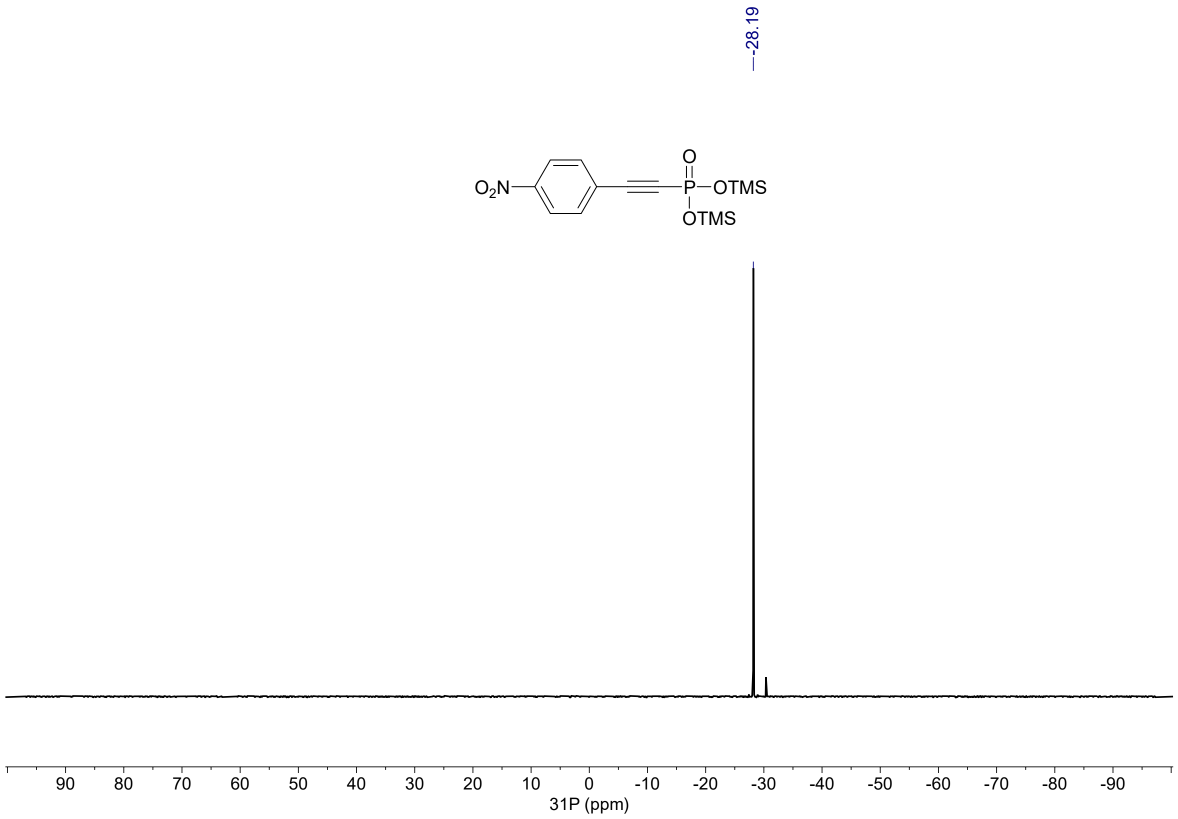

Figure S71: ^{31}P NMR spectrum of **1n′** in $CDCl_3$ at 25 °C, recorded at 203 MHz.

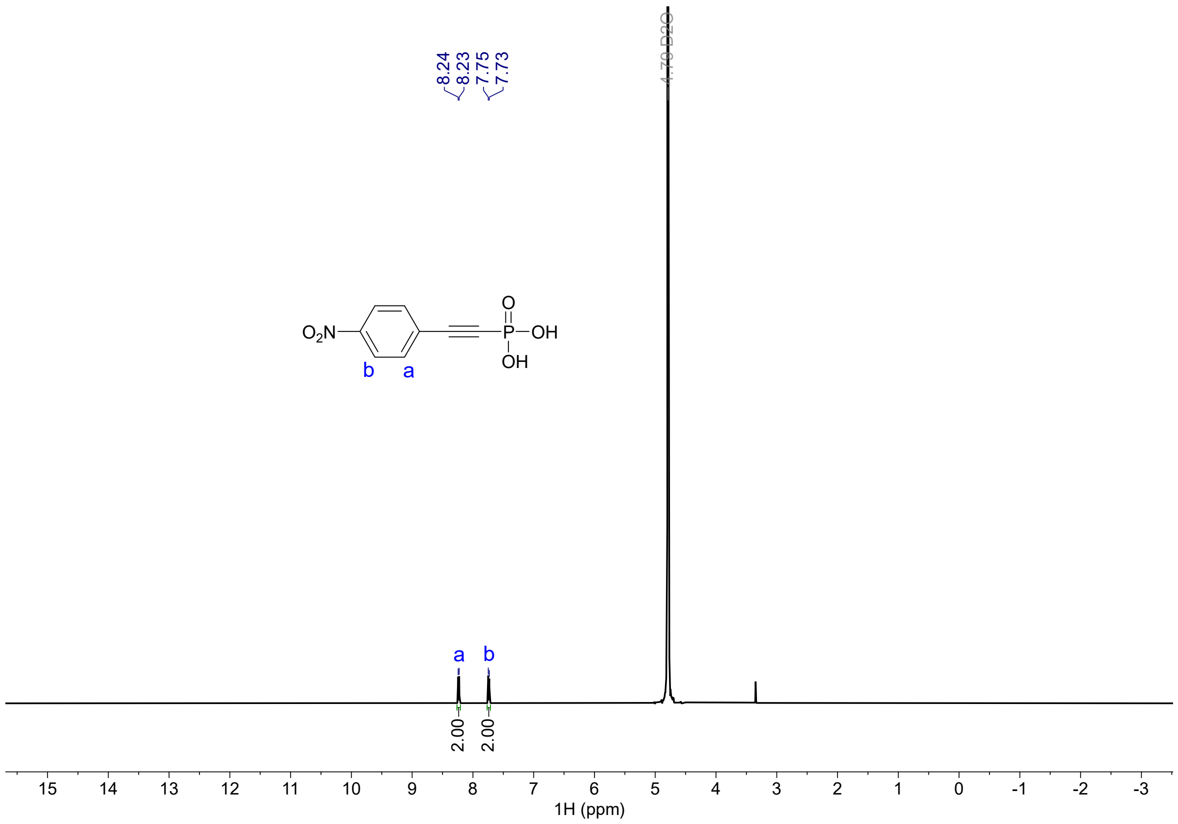

Figure S72: ^{1}H NMR spectrum of **1n** in D_2O at 25 °C, recorded at 500 MHz.

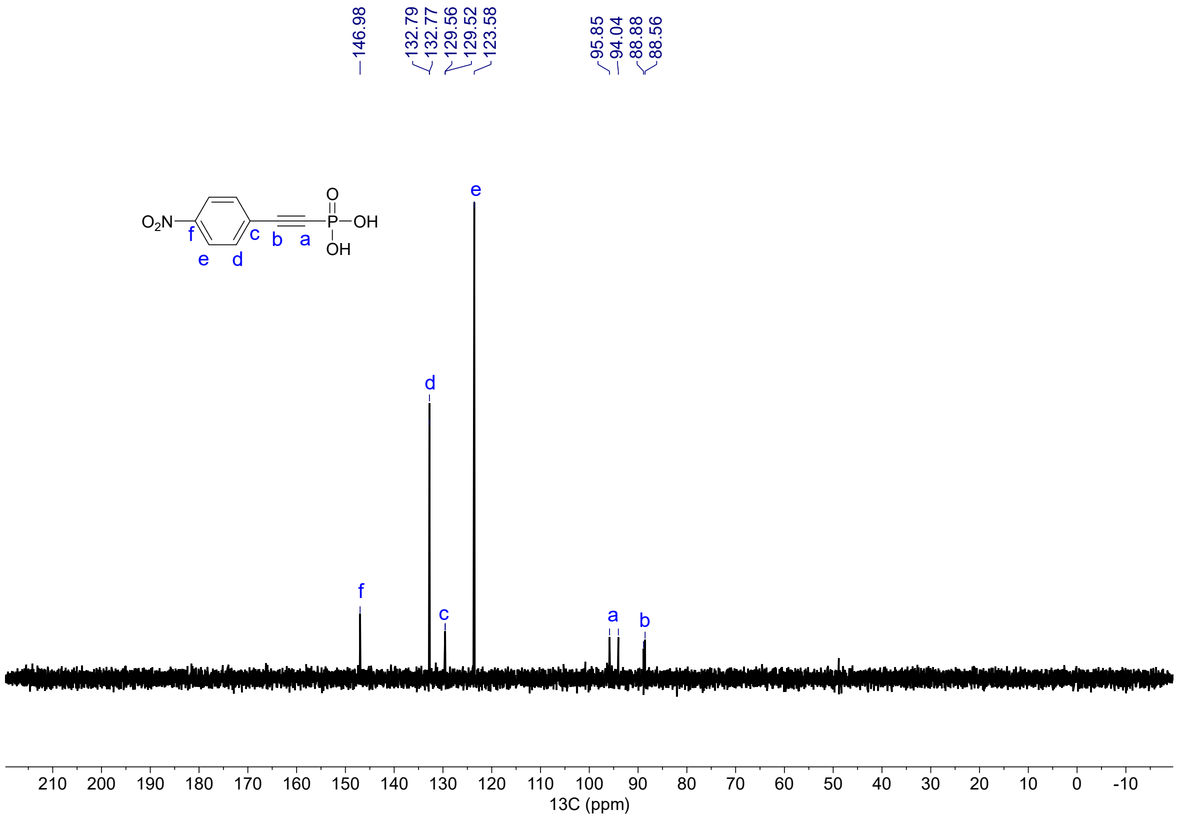

Figure S73: ^{13}C NMR spectrum of **1n** in D_2O at 25 °C, recorded at 126 MHz.

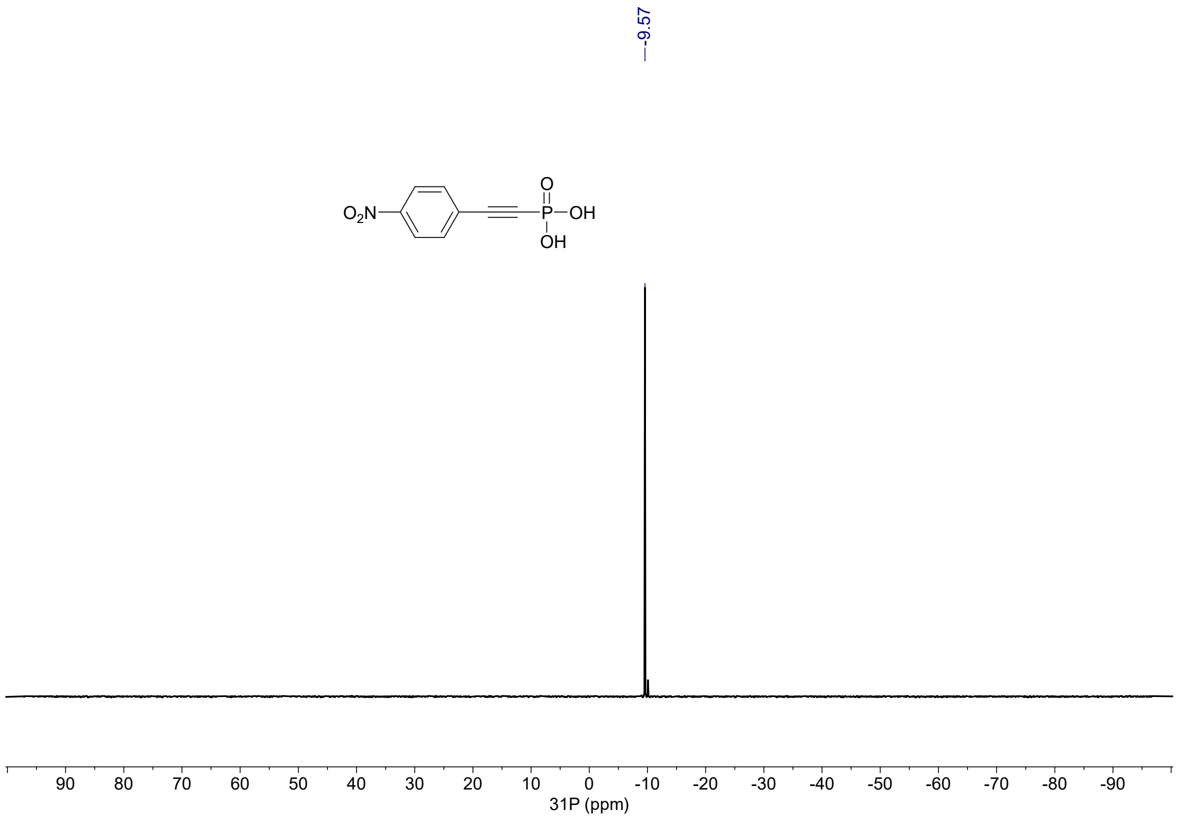

Figure S74: ^{31}P NMR spectrum of **1n** in D_2O at 25 °C, recorded at 203 MHz.

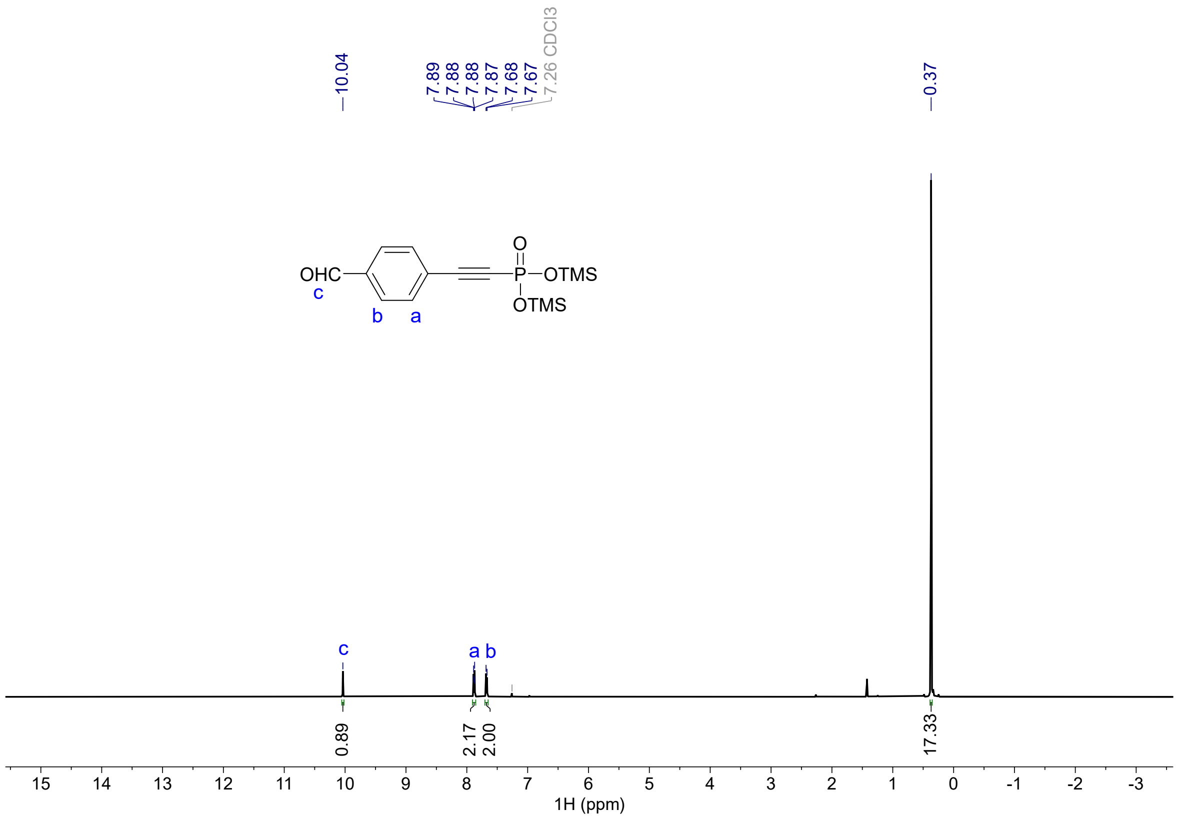

Figure S75: ^{1}H NMR spectrum of **1o′** in $CDCl_3$ at 25 °C, recorded at 500 MHz.

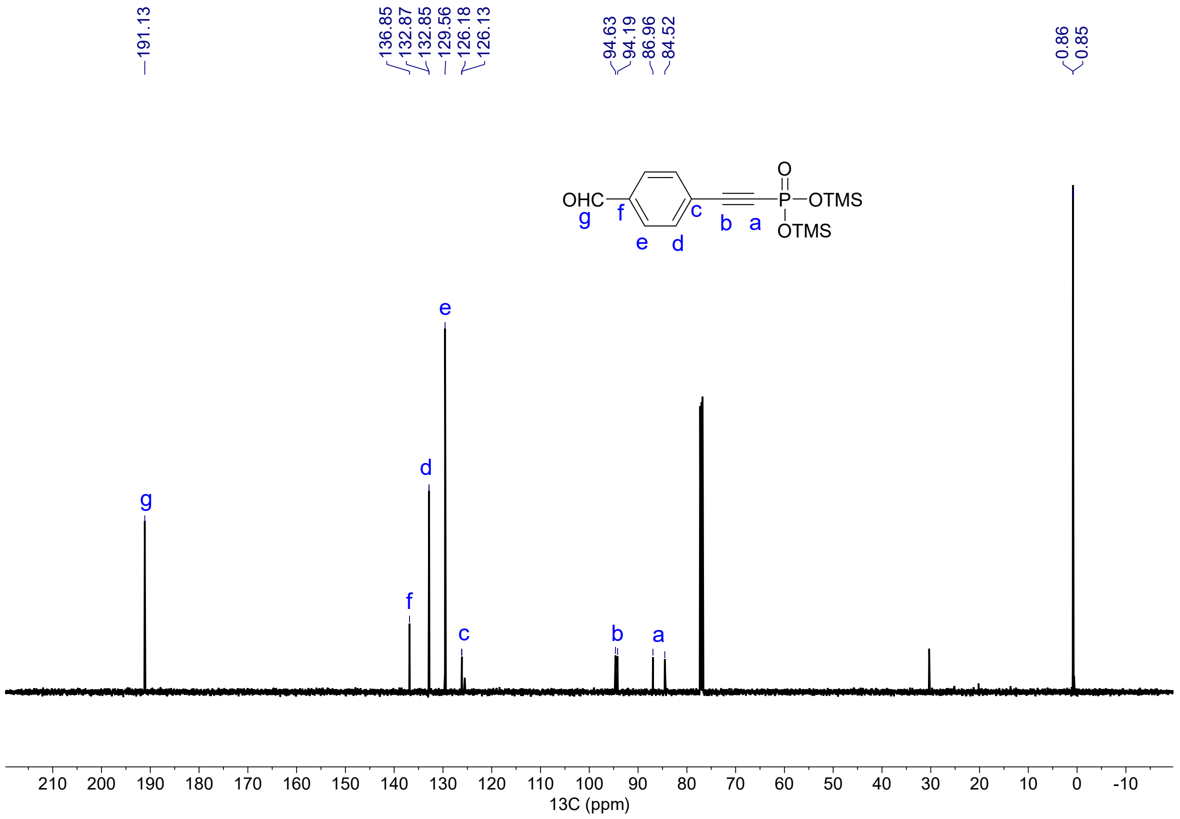

Figure S76: ^{13}C NMR spectrum of **1o′** in $CDCl_3$ at 25 °C, recorded at 126 MHz.

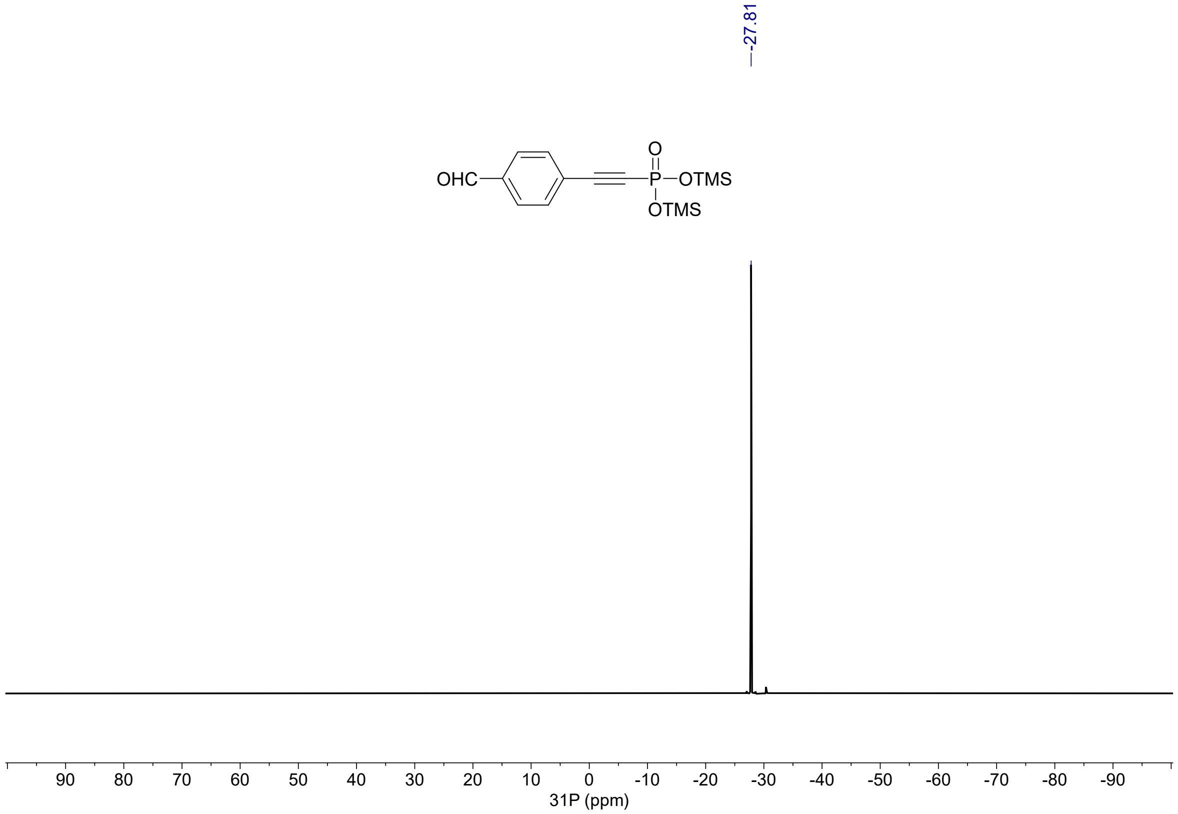

Figure S77: ^{31}P NMR spectrum of **1o′** in $CDCl_3$ at 25 °C, recorded at 203 MHz.

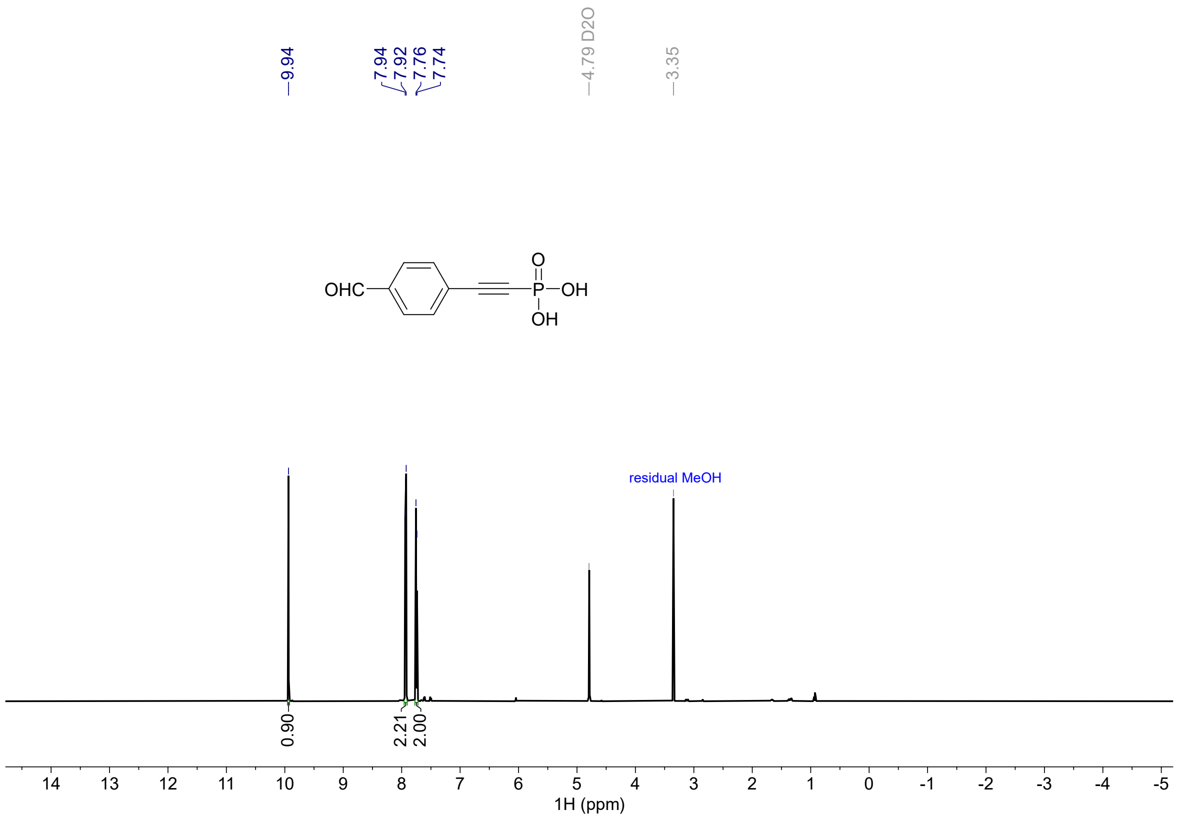

Figure S78: ^{1}H NMR spectrum of **1o** in D_2O at 25 °C, recorded at 500 MHz.

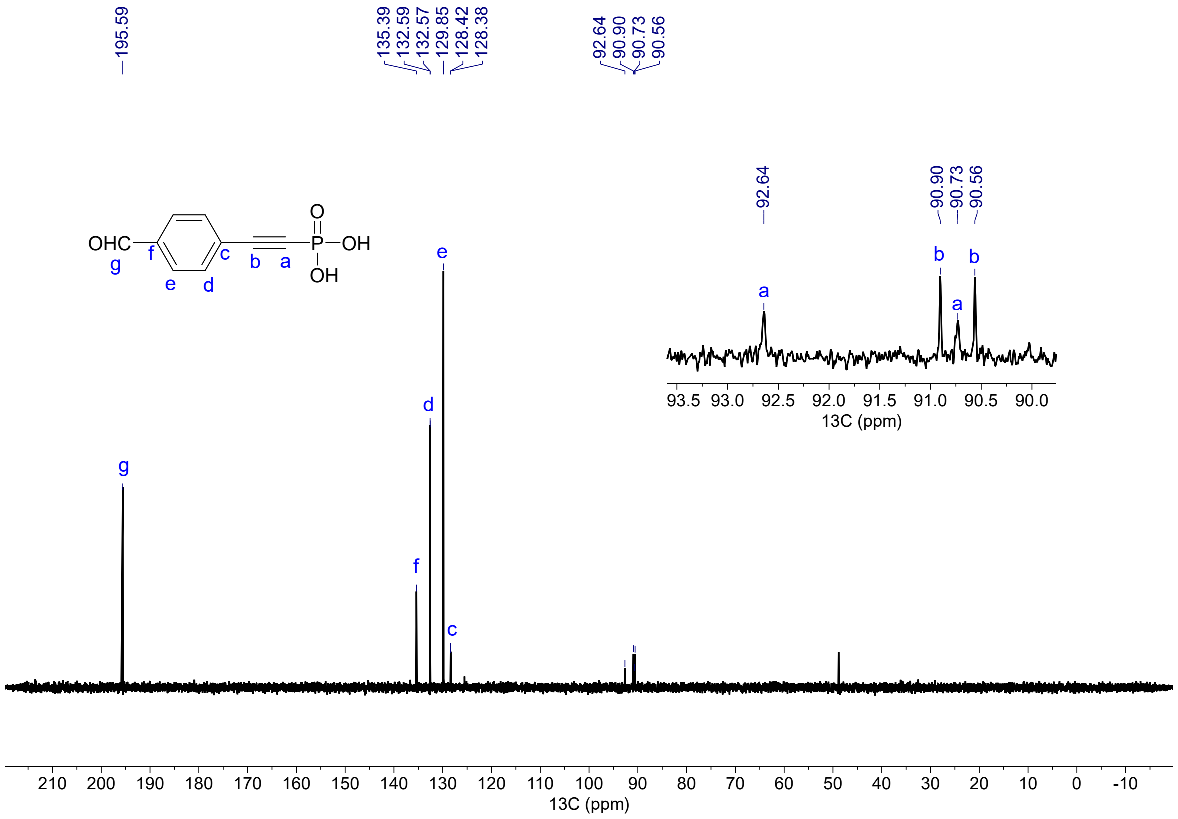

Figure S79: ^{13}C NMR spectrum of **1o** in D_2O at 25 °C, recorded at 126 MHz.

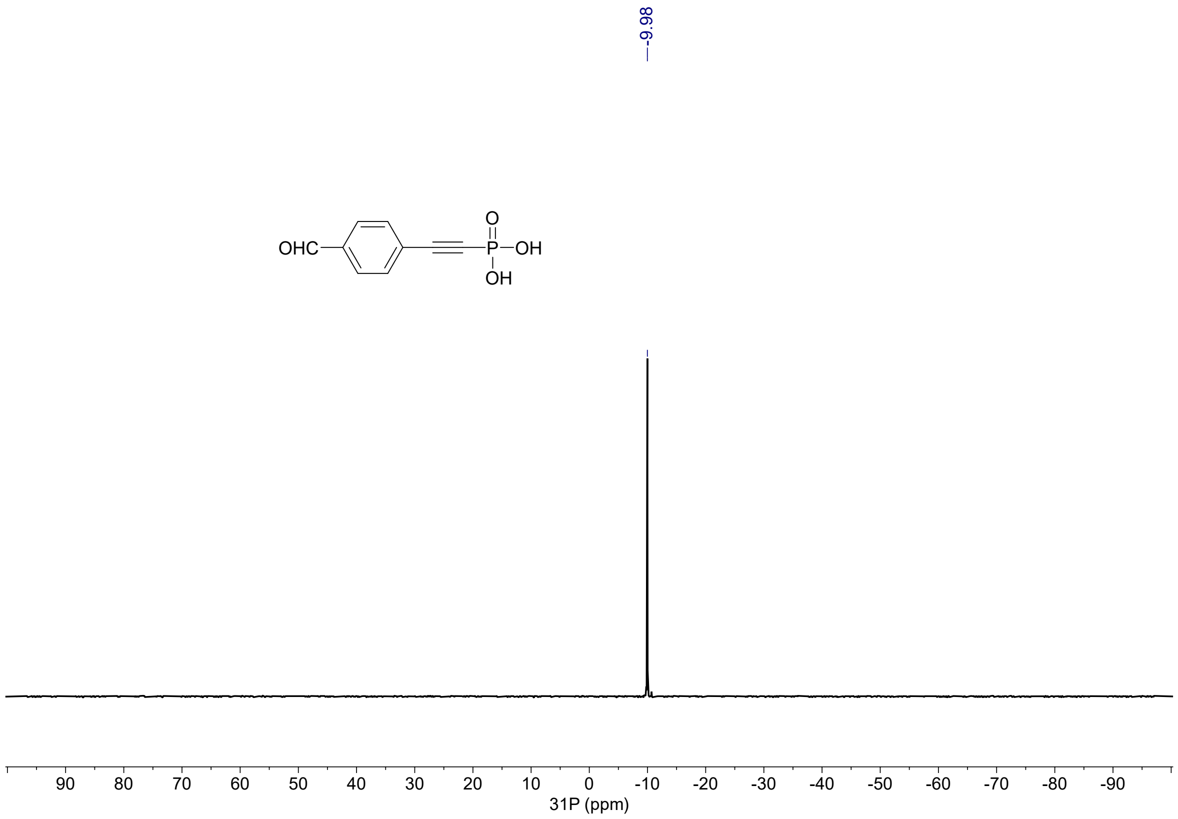

Figure S80: ^{31}P NMR spectrum of **1o** in D_2O at 25 °C, recorded at 203 MHz.

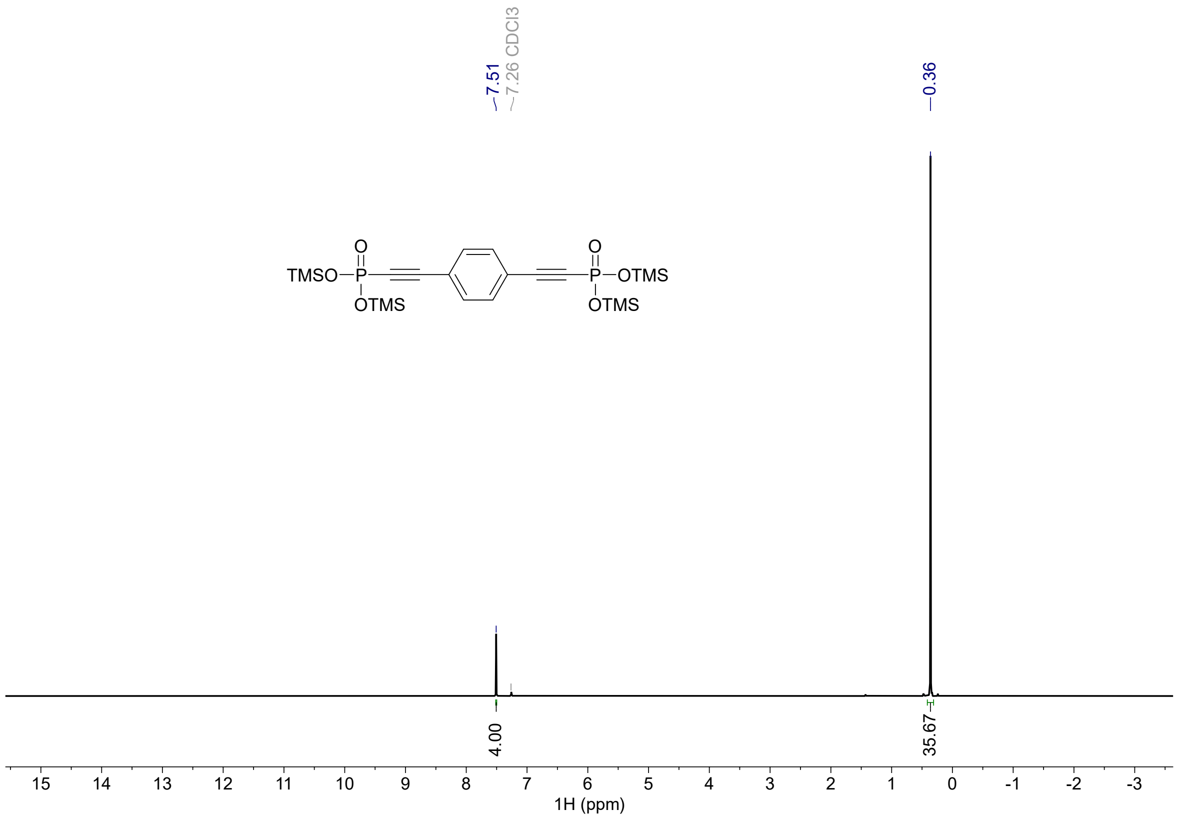

Figure S81: ^{1}H NMR spectrum of **1p′** in $CDCl_3$ at 25 °C, recorded at 500 MHz.

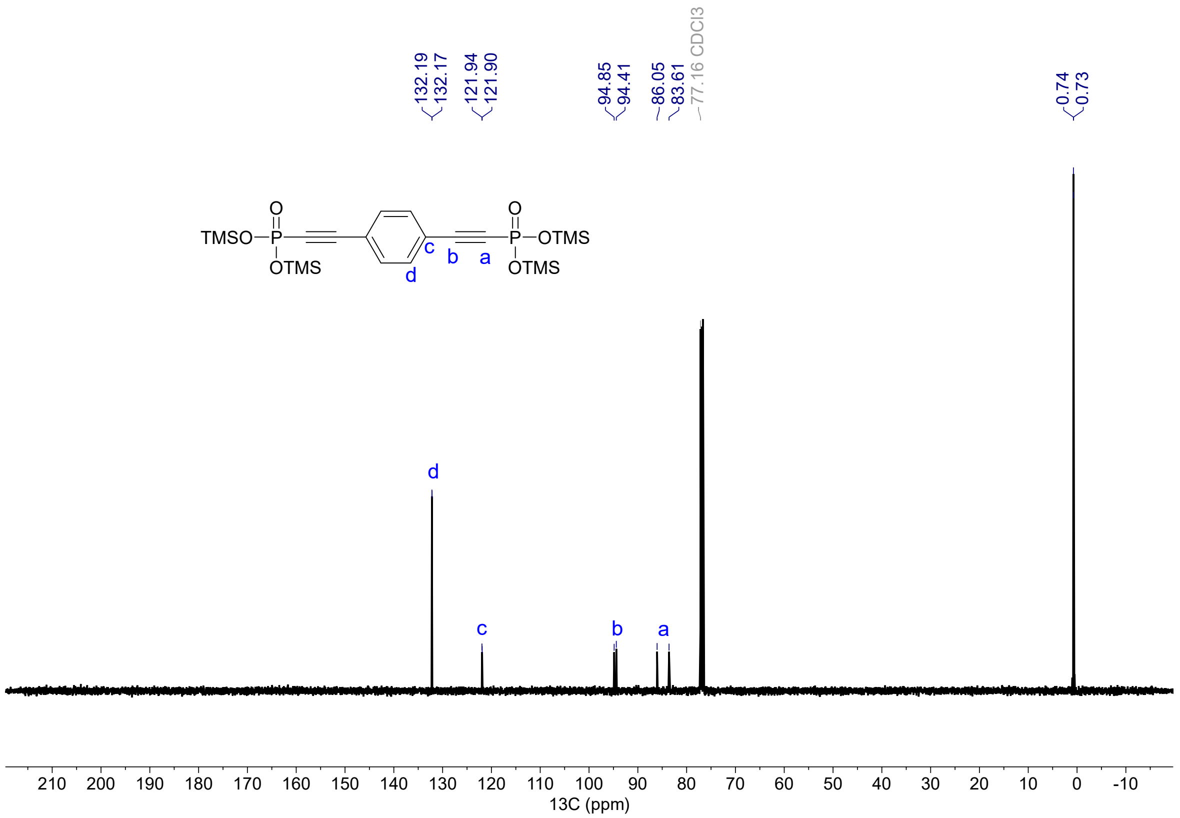

Figure S82: ^{13}C NMR spectrum of **1p′** in $CDCl_3$ at 25 °C, recorded at 126 MHz.

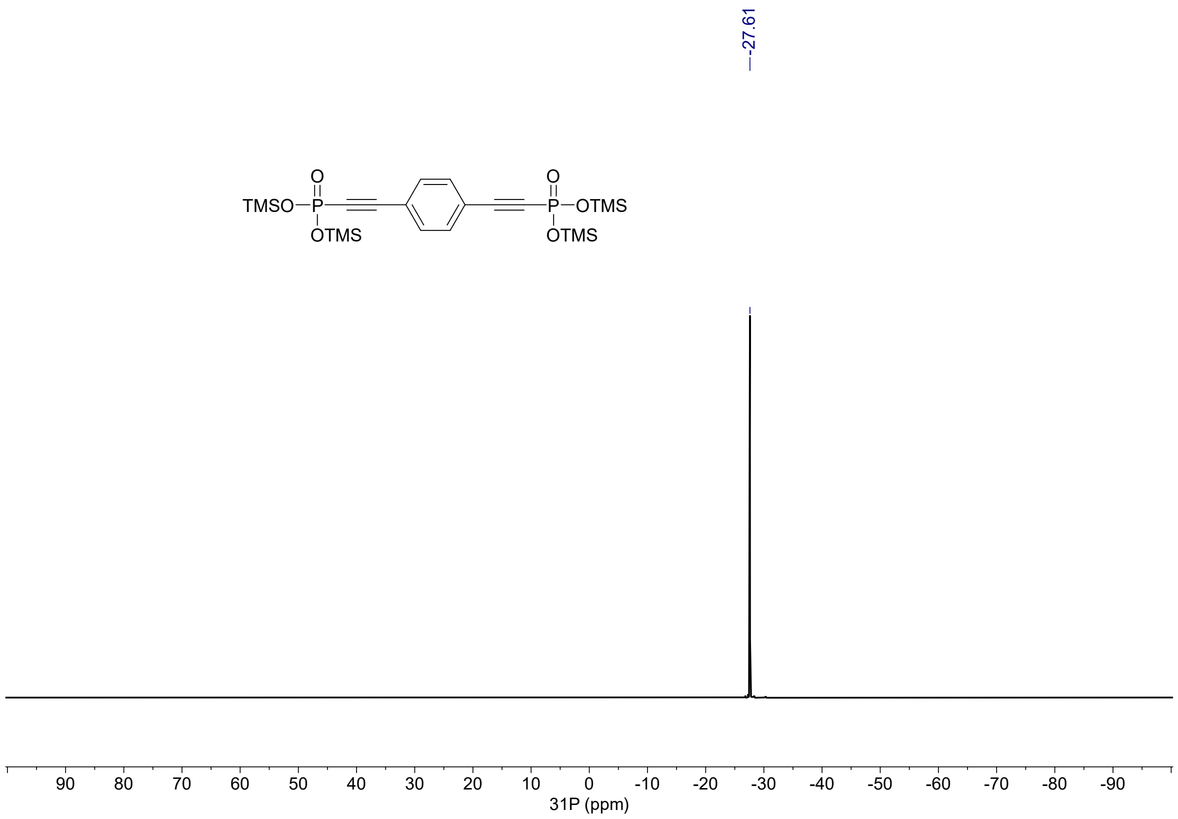

Figure S83: ^{31}P NMR spectrum of **1p′** in $CDCl_3$ at 25 °C, recorded at 203 MHz.

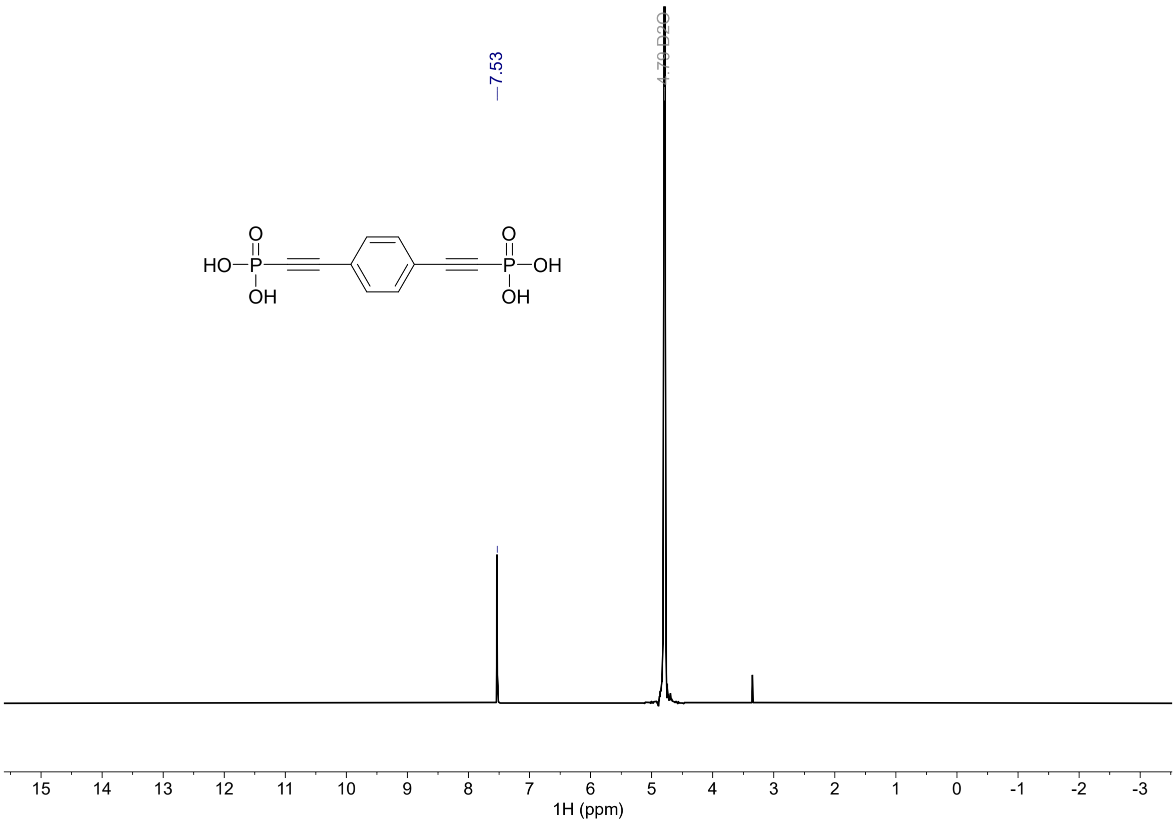

Figure S84: ^{1}H NMR spectrum of **1p** in D_2O at 25 °C, recorded at 500 MHz.

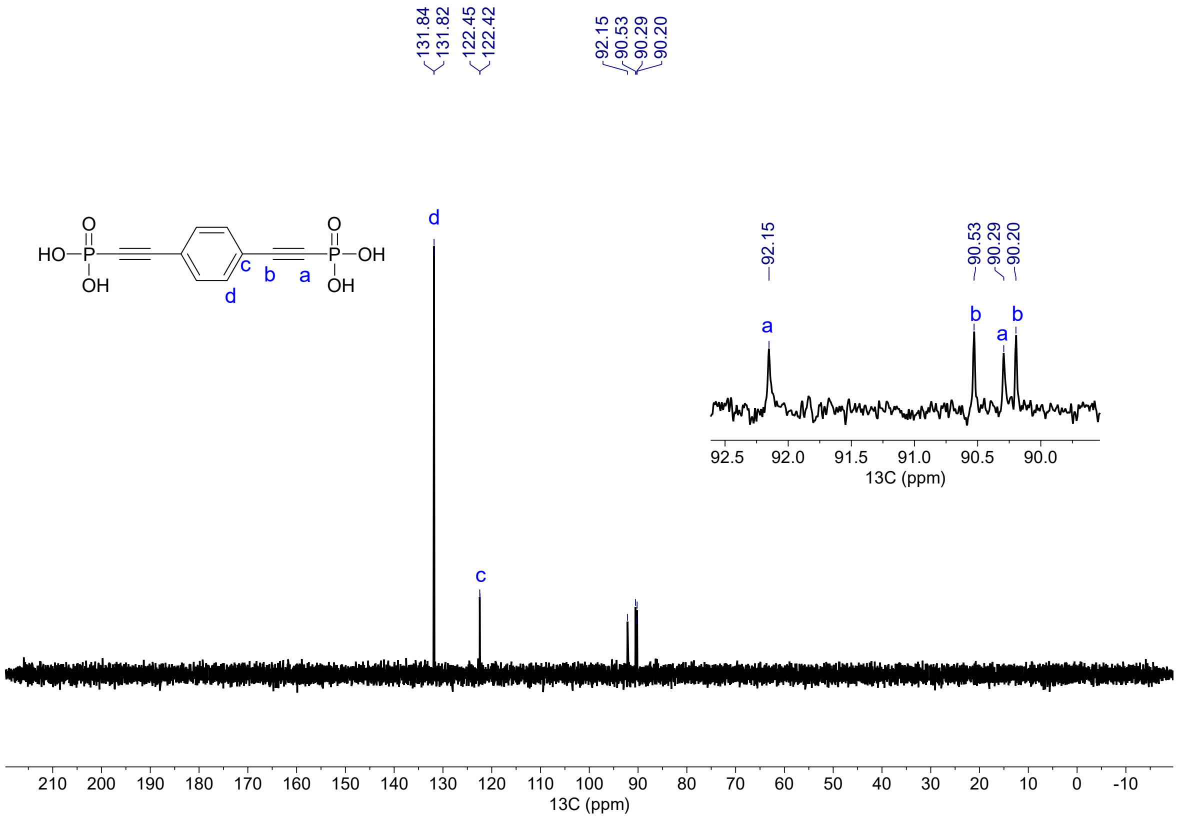

Figure S85: ^{13}C NMR spectrum of **1p** in D_2O at 25 °C, recorded at 126 MHz.

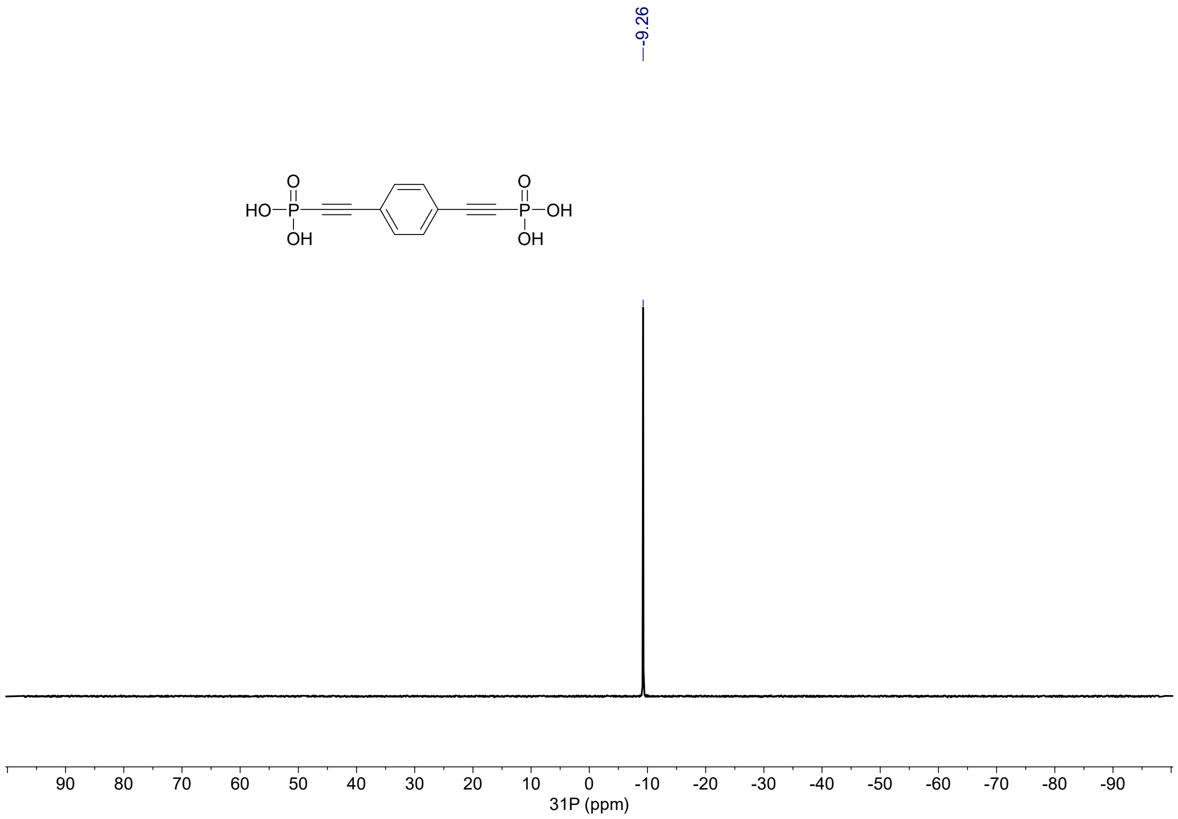

Figure S86: ^{31}P NMR spectrum of **1p** in D_2O at 25 °C, recorded at 203 MHz.

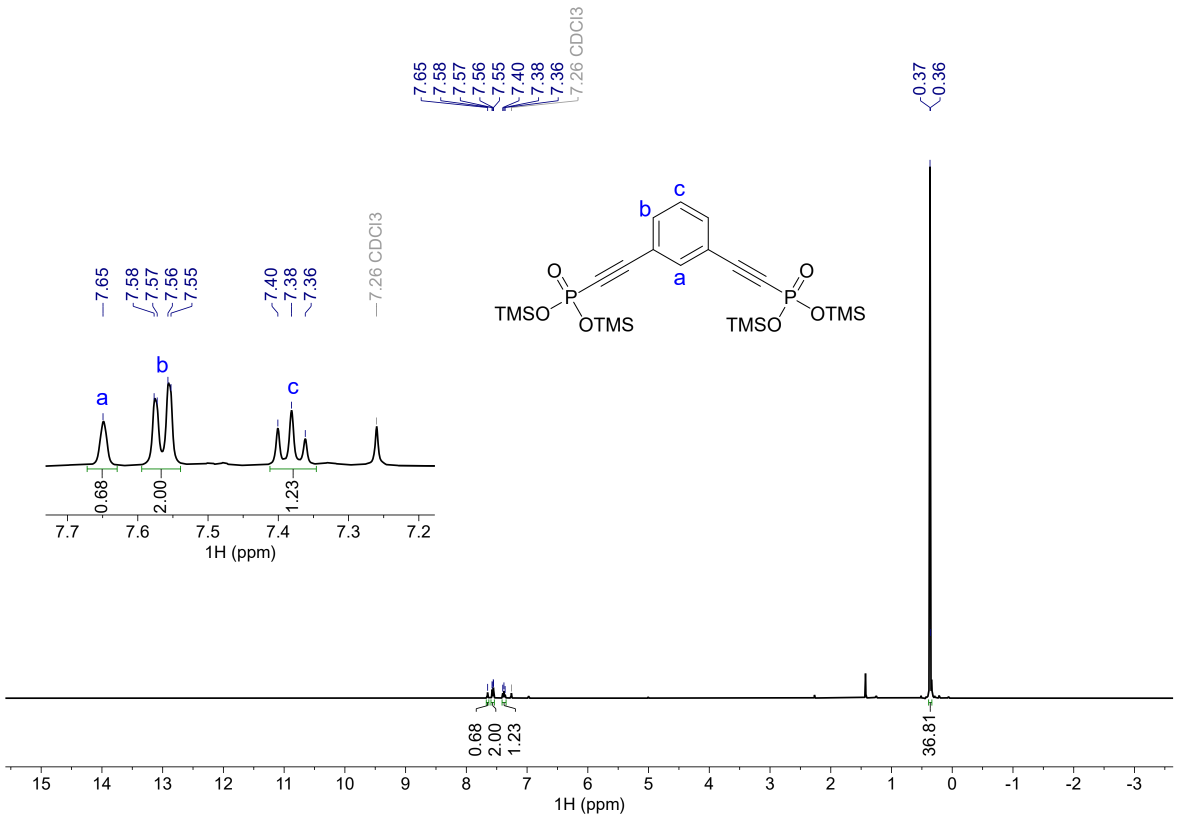

Figure S87: ^{1}H NMR spectrum of **1q′** in $CDCl_3$ at 25 °C, recorded at 500 MHz.

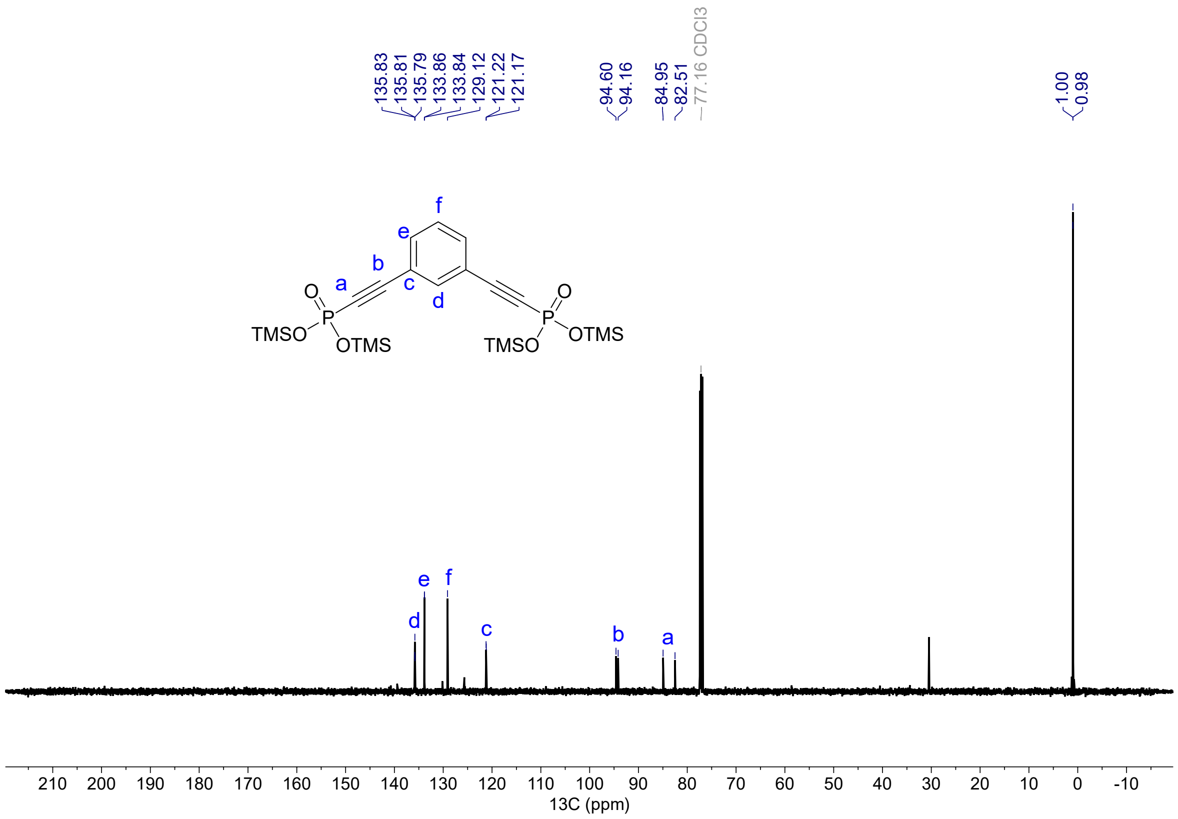

Figure S88: ^{13}C NMR spectrum of **1q′** in $CDCl_3$ at 25 °C, recorded at 126 MHz.

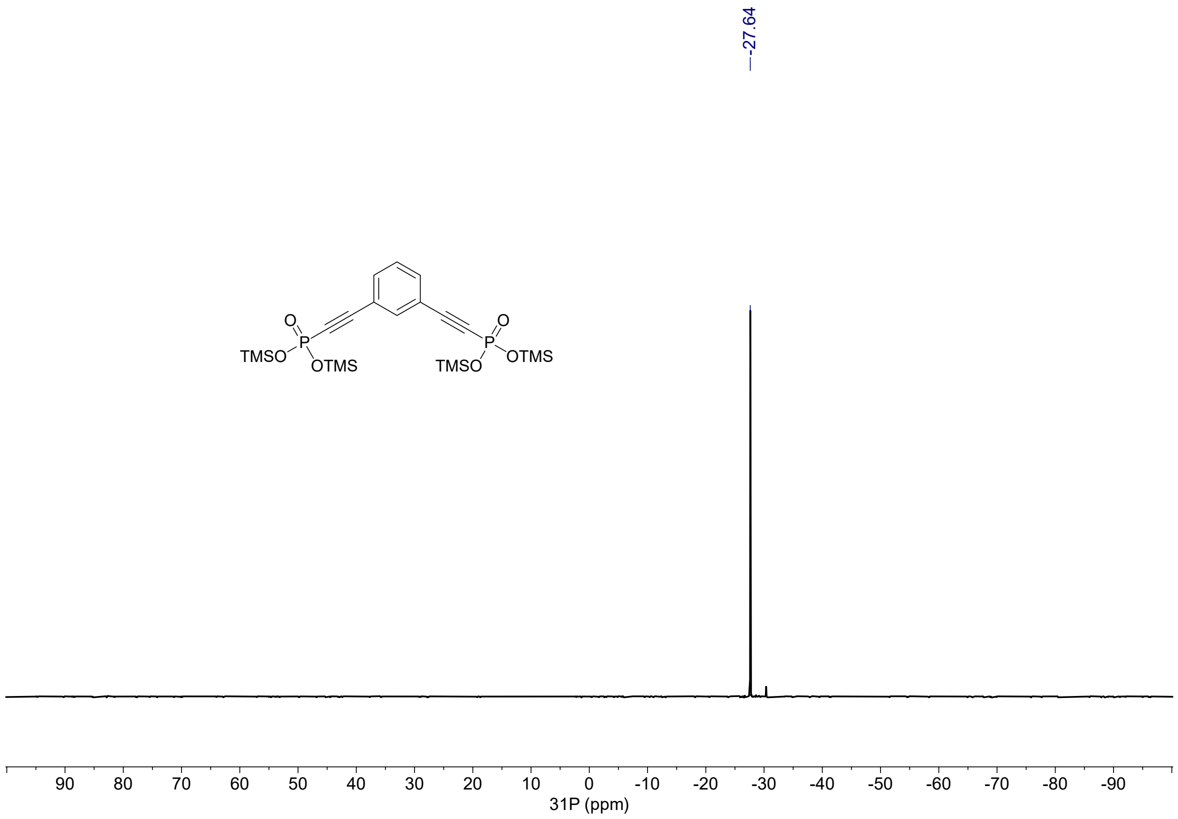

Figure S89: ^{31}P NMR spectrum of **1q′** in $CDCl_3$ at 25 °C, recorded at 203 MHz.

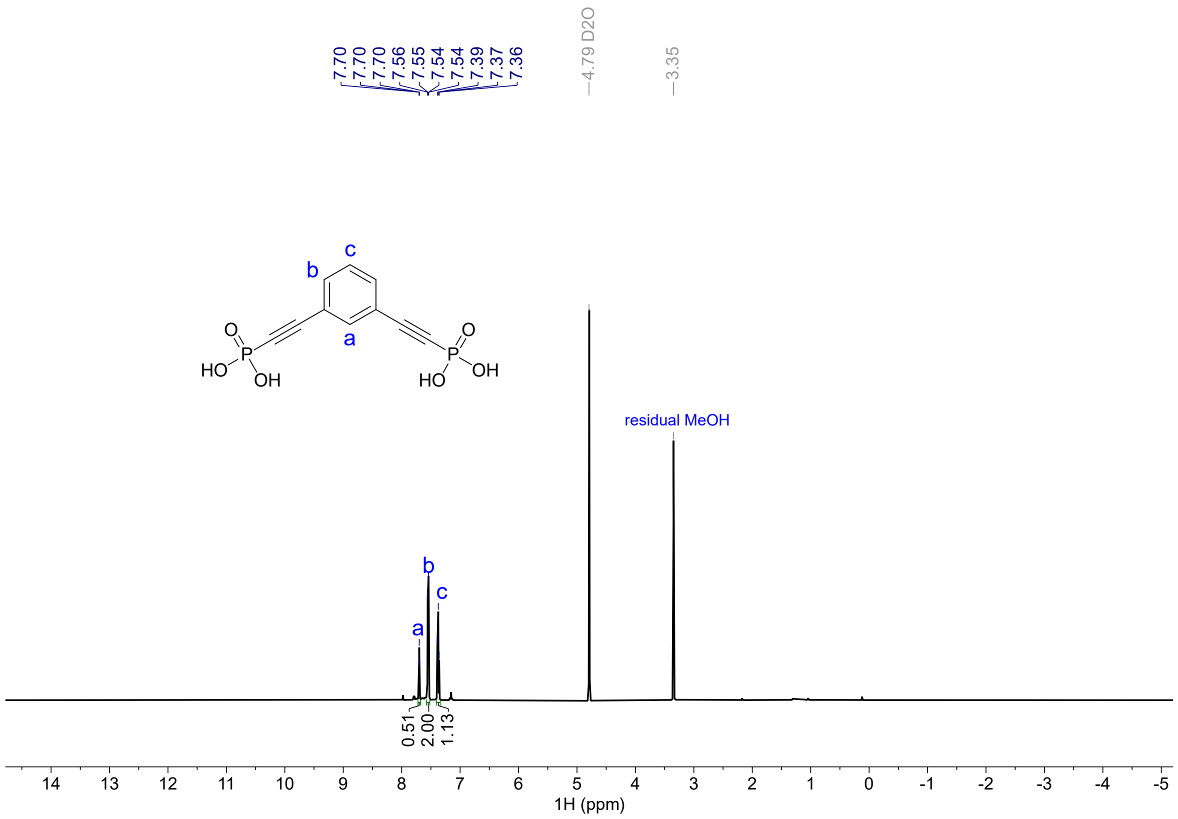

Figure S90: ^{1}H NMR spectrum of **1q** in D_2O at 25 °C, recorded at 500 MHz.

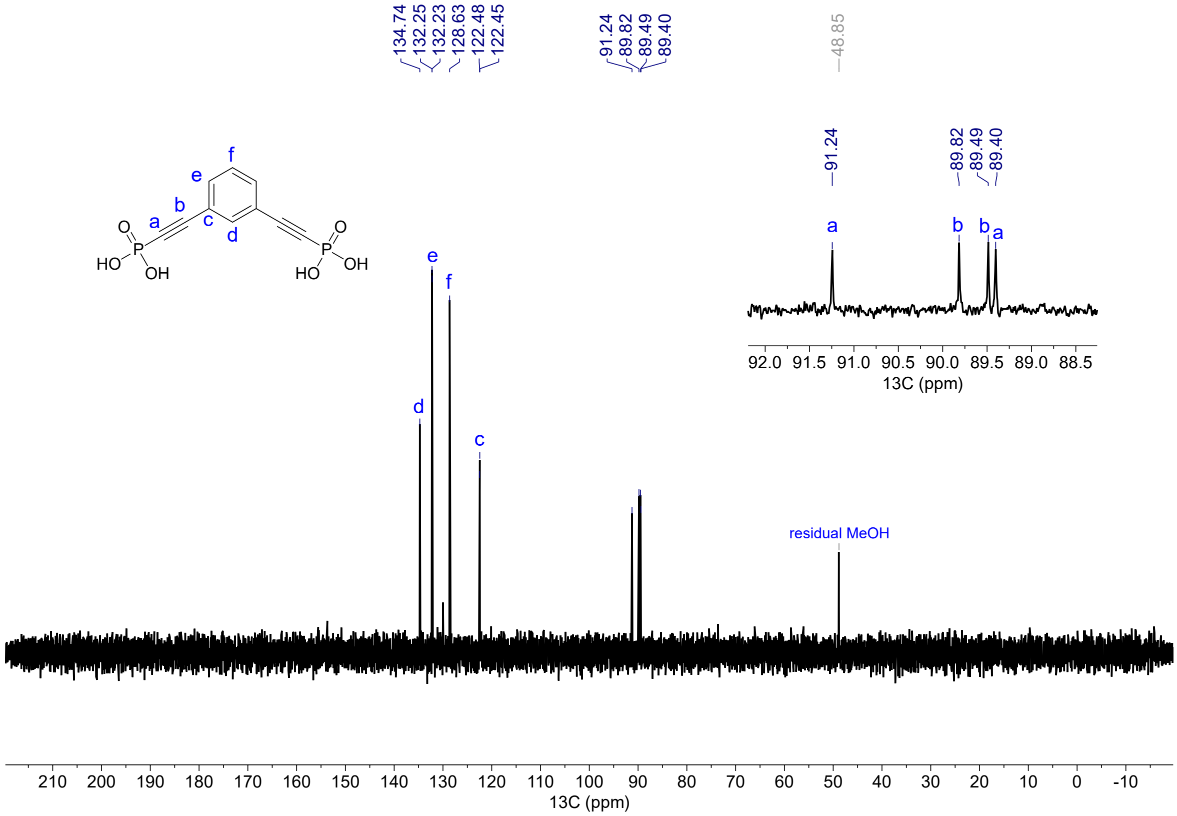

Figure S91: ^{13}C NMR spectrum of **1q** in D_2O at 25 °C, recorded at 126 MHz.

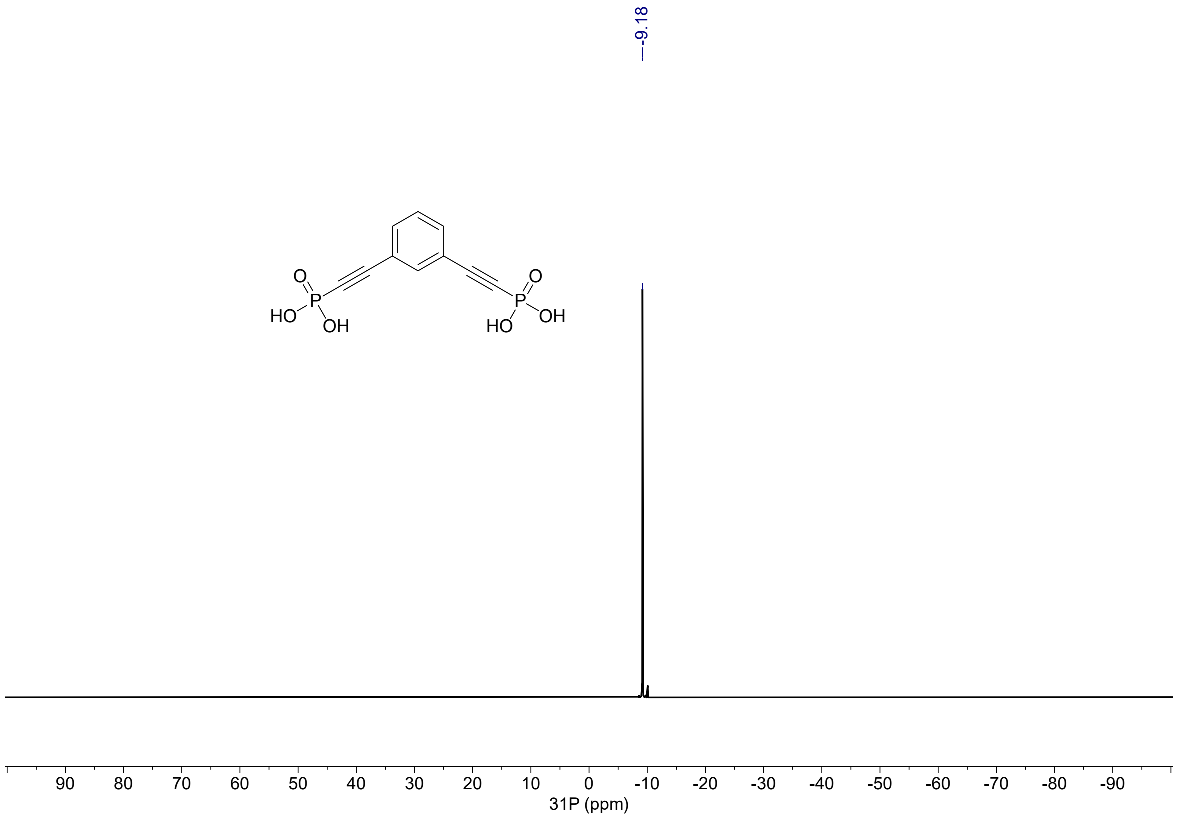

Figure S92: ^{31}P NMR spectrum of **1q** in D_2O at 25 °C, recorded at 203 MHz.

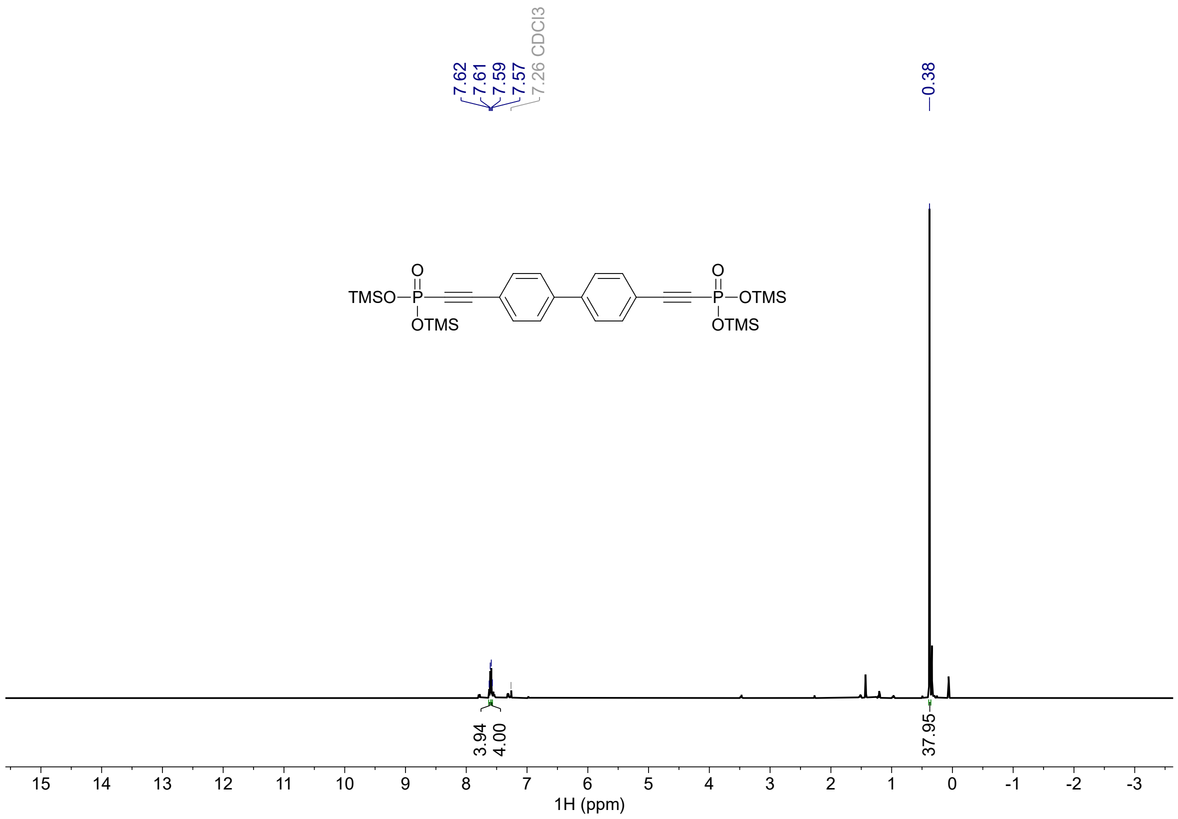

Figure S93: ^{1}H NMR spectrum of **1r′** in $CDCl_3$ at 25 °C, recorded at 500 MHz.

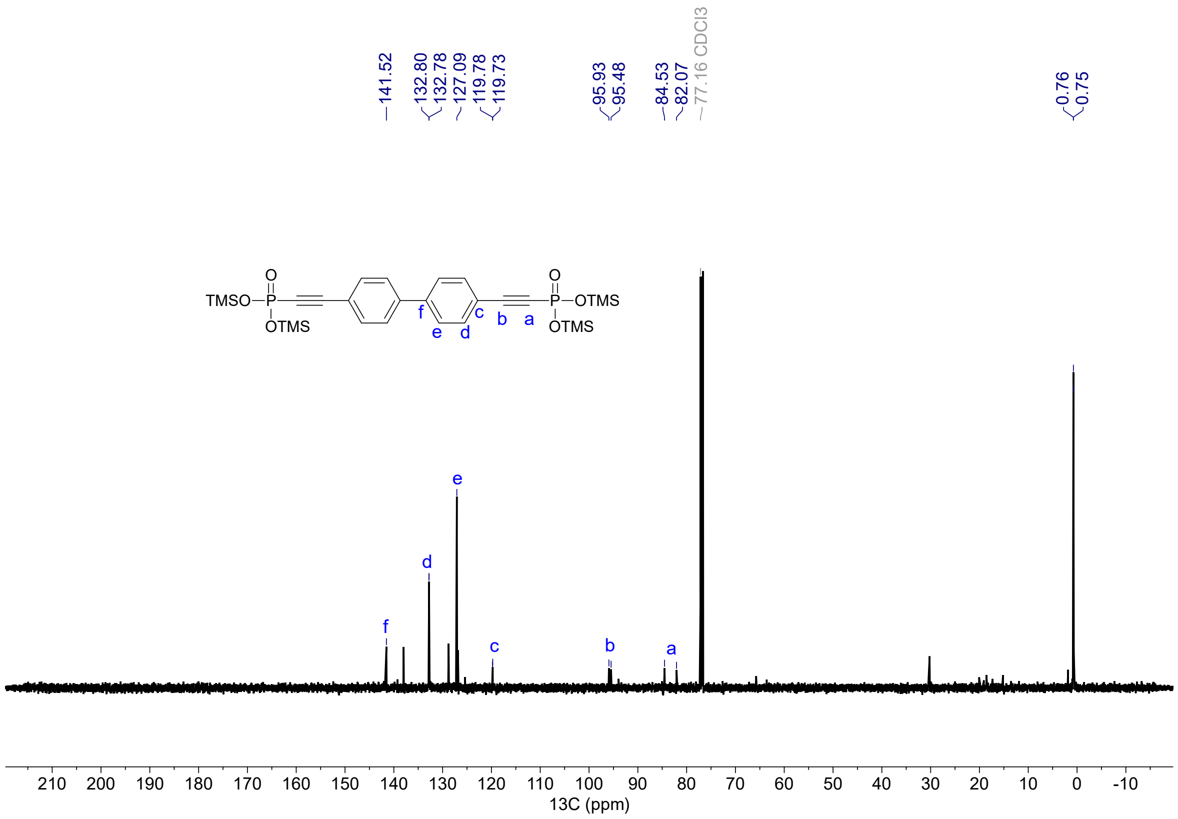

Figure S94: ^{13}C NMR spectrum of **1r′** in $CDCl_3$ at 25 °C, recorded at 126 MHz.

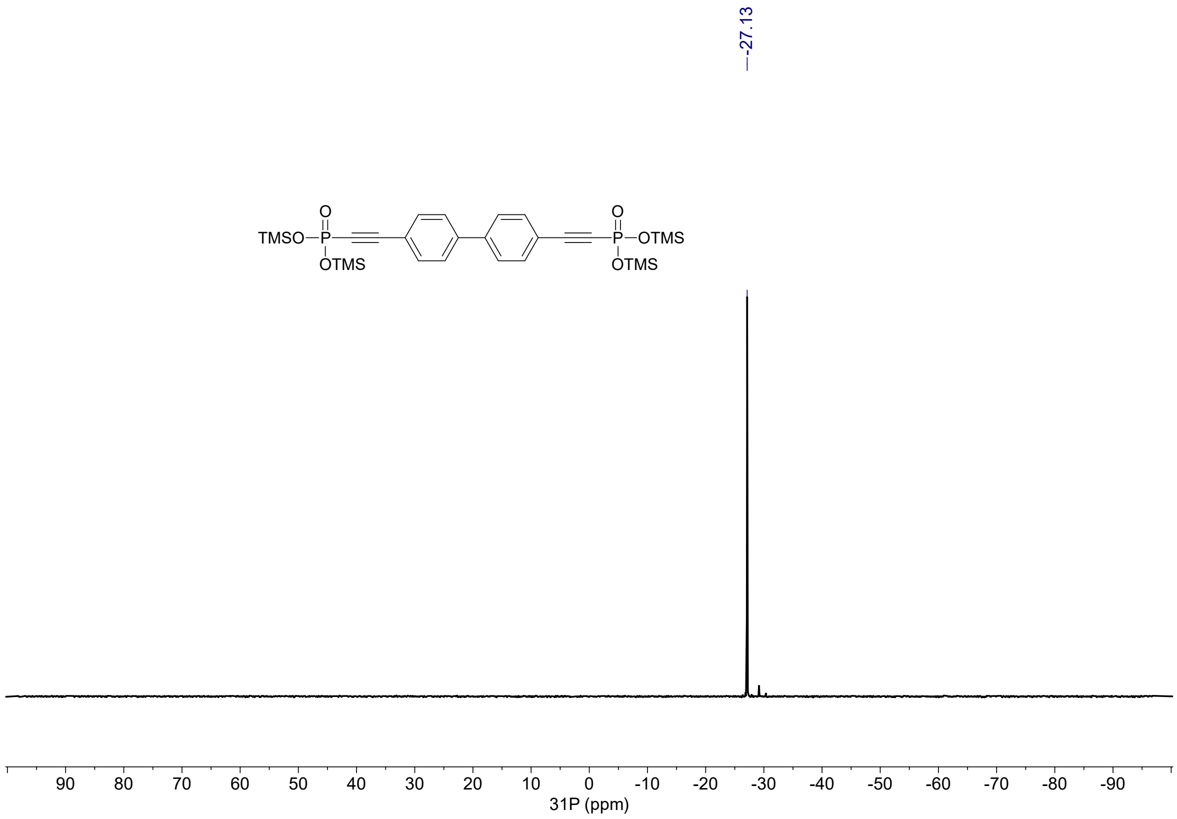

Figure S95: ^{31}P NMR spectrum of **1r′** in $CDCl_3$ at 25 °C, recorded at 203 MHz.

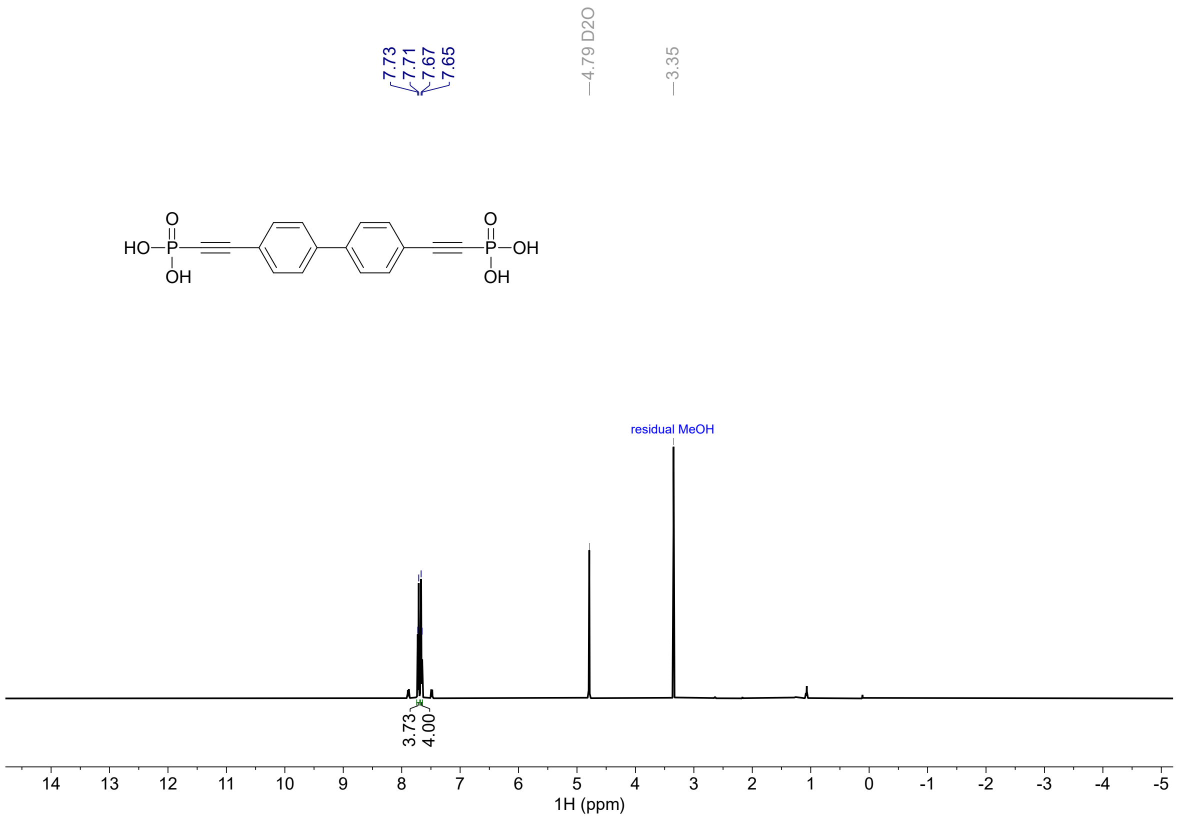

Figure S96: ^{1}H NMR spectrum of **1r** in D_2O at 25 °C, recorded at 500 MHz.

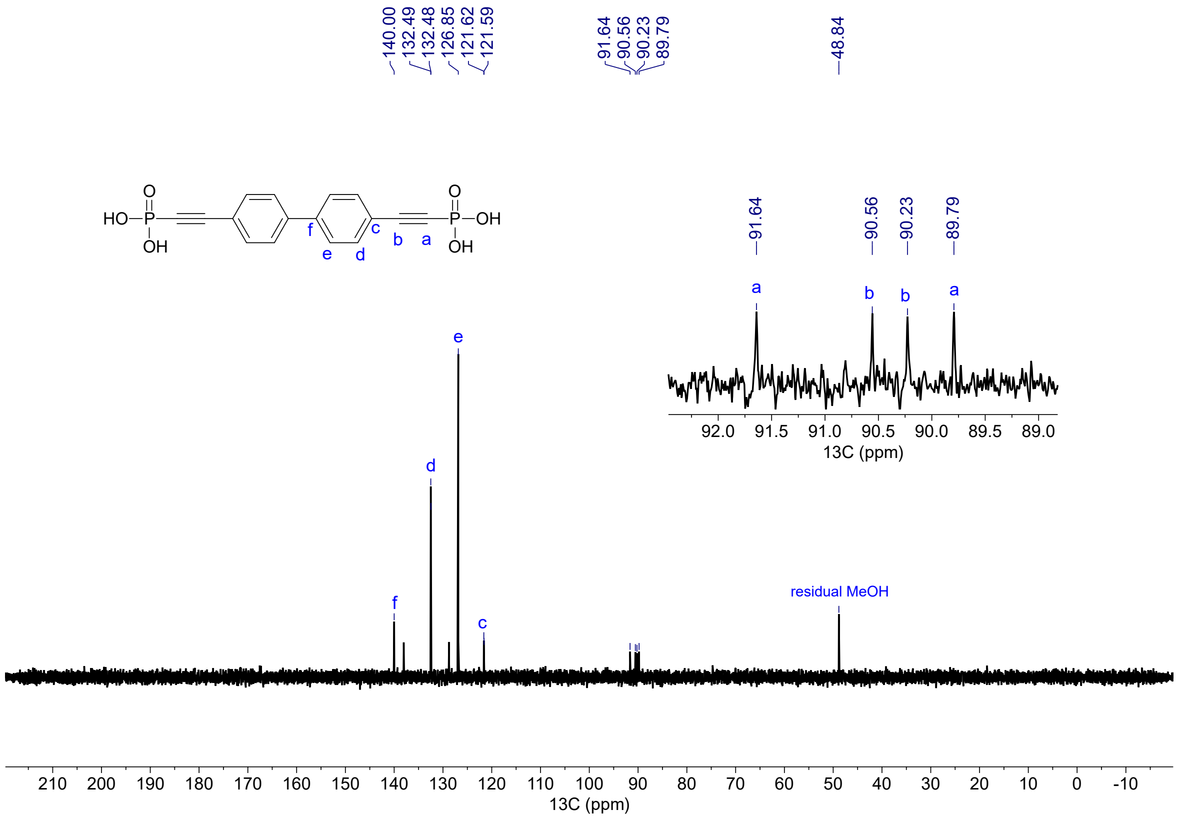

Figure S97: ^{13}C NMR spectrum of **1r** in D_2O at 25 °C, recorded at 126 MHz.

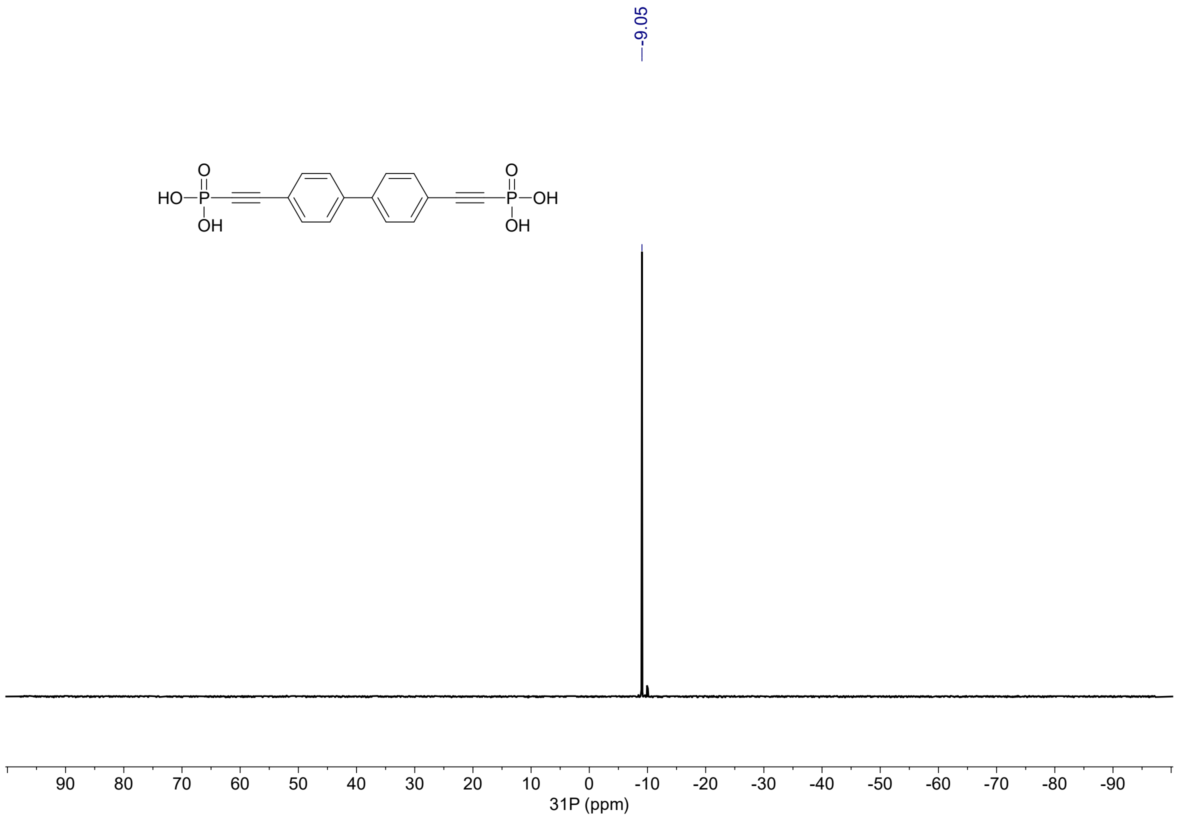

Figure S98: ^{31}P NMR spectrum of **1r** in D_2O at 25 °C, recorded at 203 MHz.

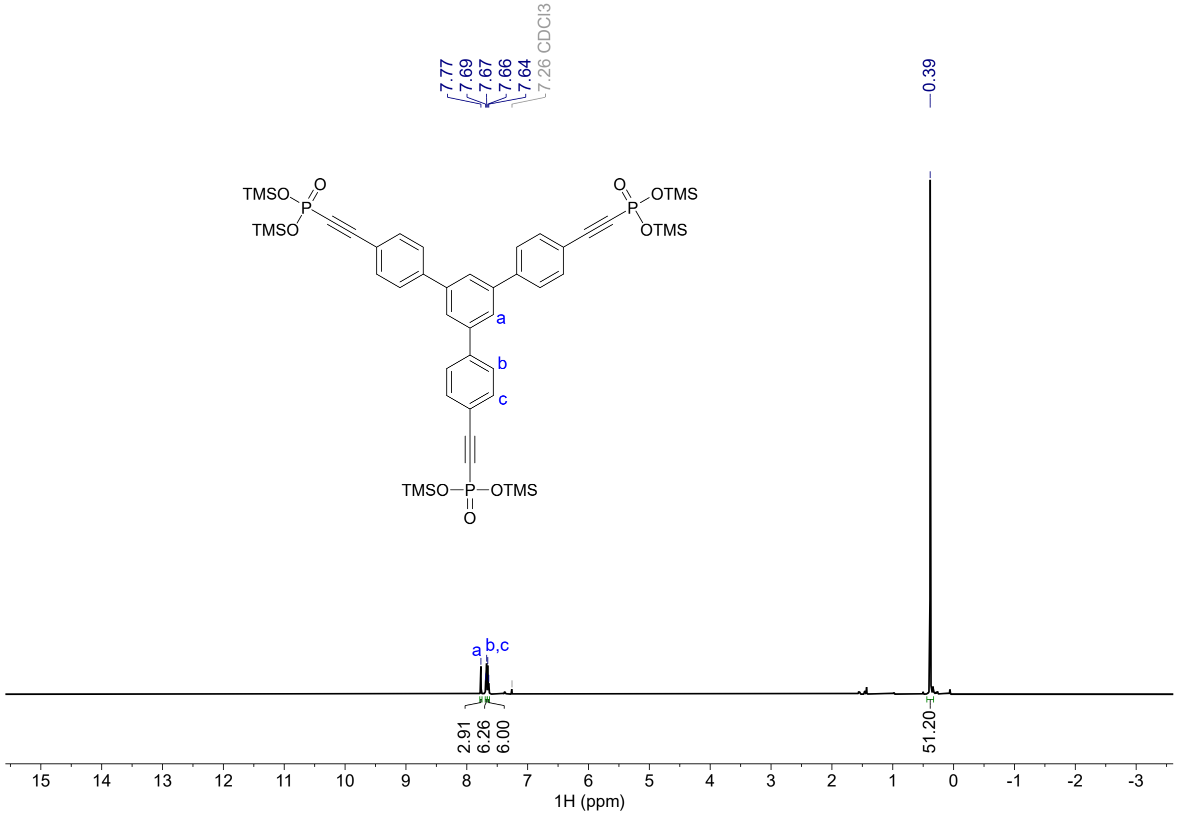

Figure S99: ^{1}H NMR spectrum of **1s′** in $CDCl_3$ at 25 °C, recorded at 500 MHz.

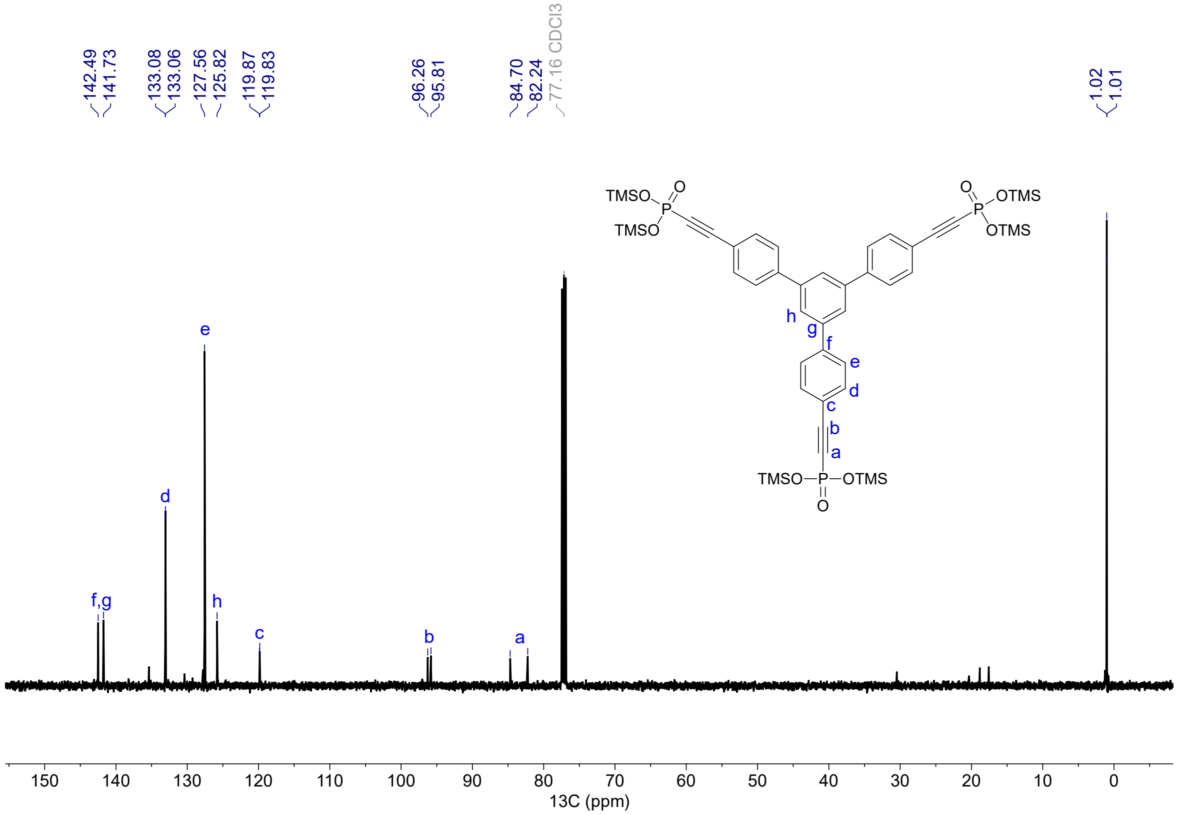

Figure S100: ^{13}C NMR spectrum of **1s′** in $CDCl_3$ at 25 °C, recorded at 126 MHz.

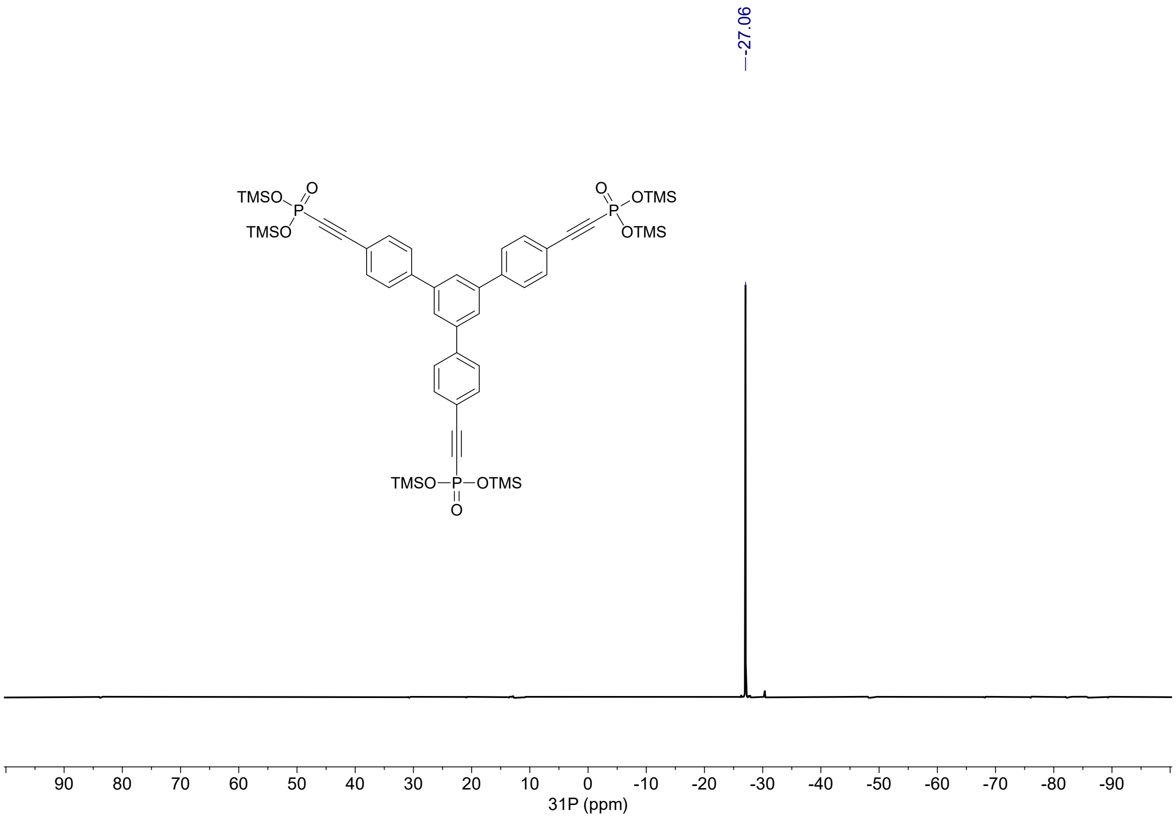

Figure S101: ^{31}P NMR spectrum of **1s′** in $CDCl_3$ at 25 °C, recorded at 203 MHz.

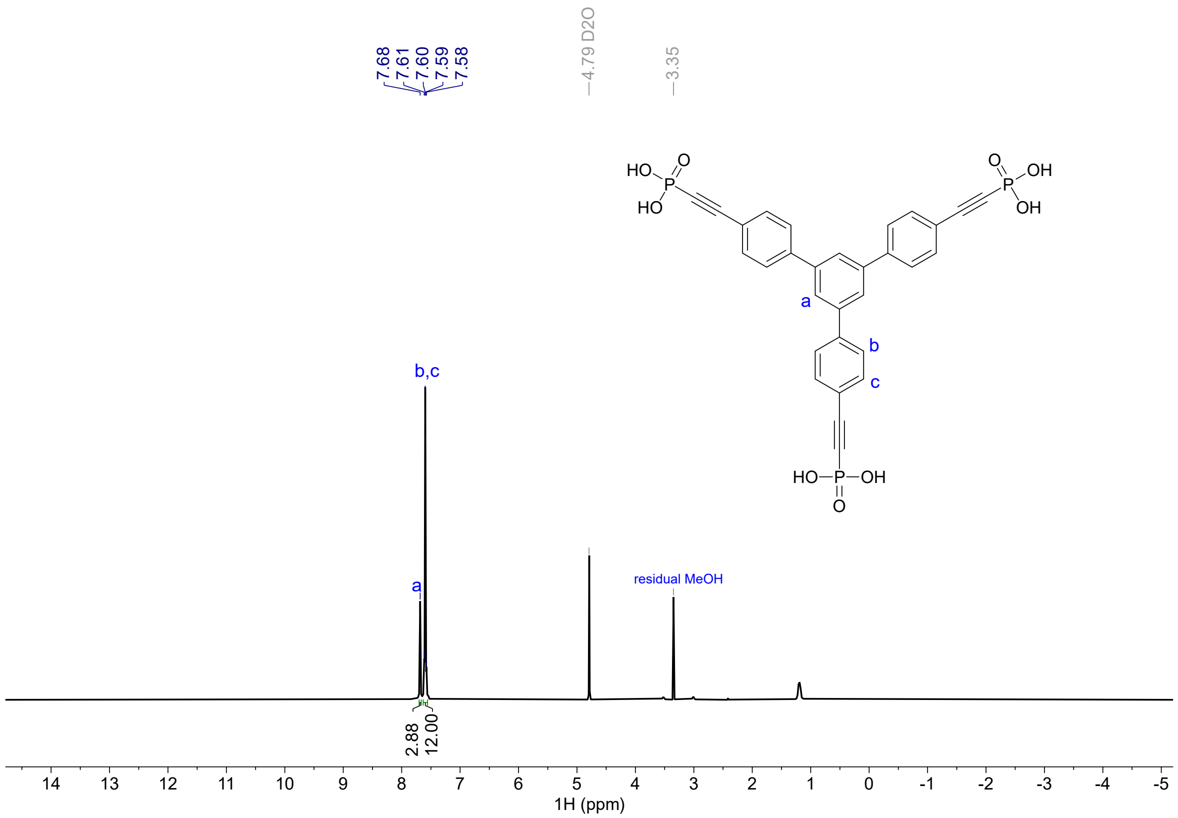

Figure S102: ^{1}H NMR spectrum of **1s** in D_2O at 25 °C, recorded at 500 MHz.

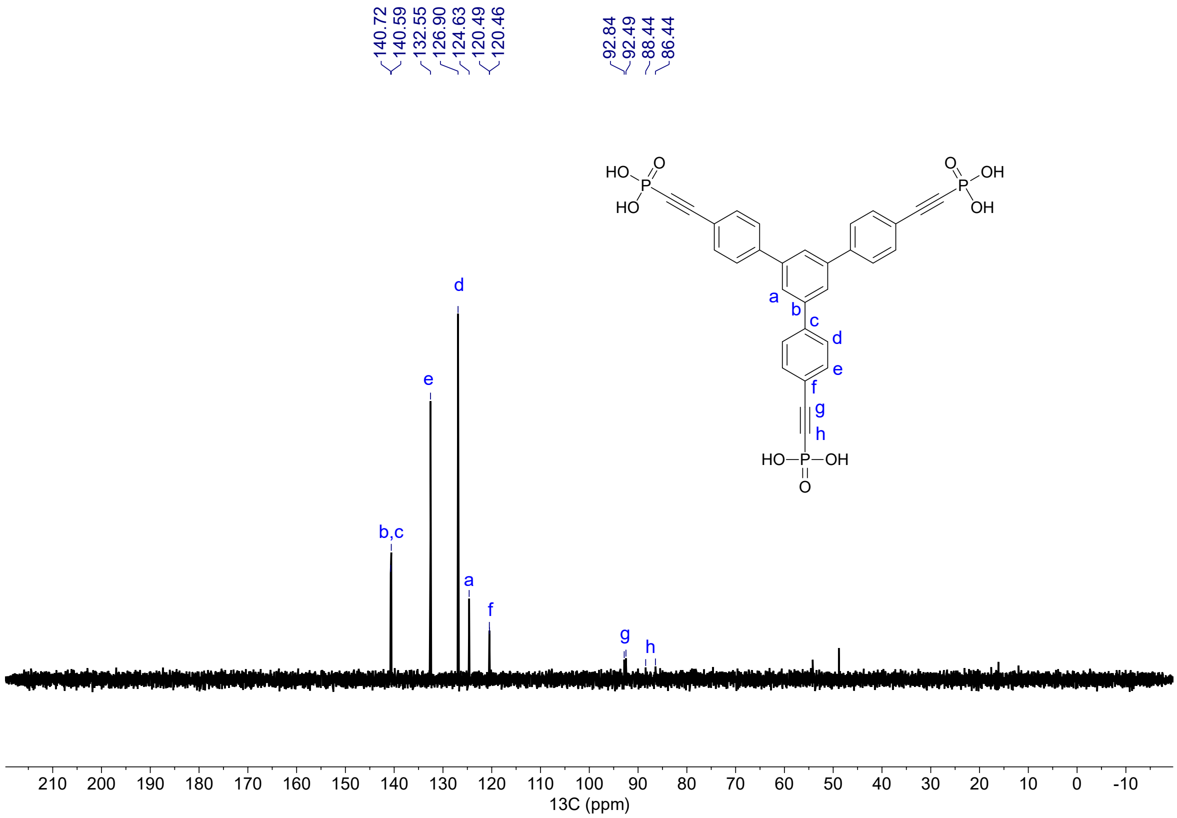

Figure S103: ^{13}C NMR spectrum of **1s** in D_2O at 25 °C, recorded at 126 MHz.

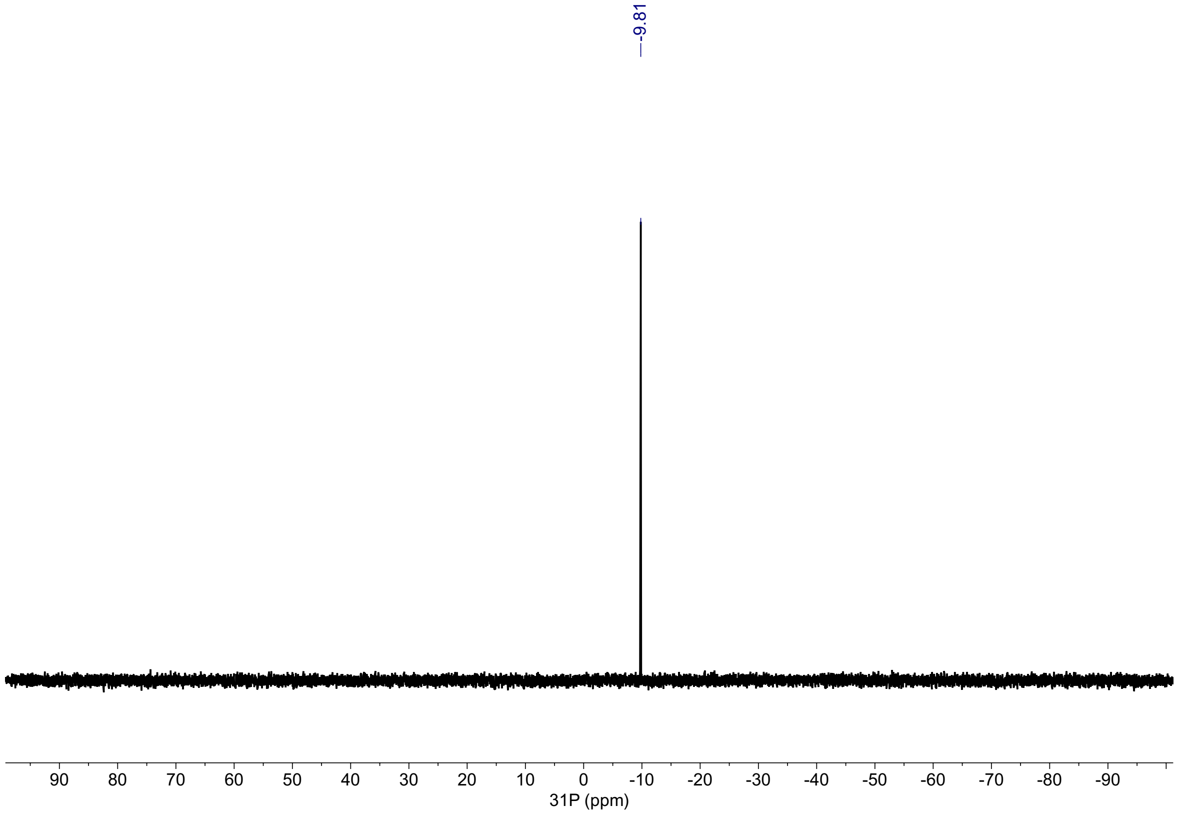

Figure S104: ^{31}P NMR spectrum of **1s** in D_2O at 25 °C, recorded at 203 MHz.

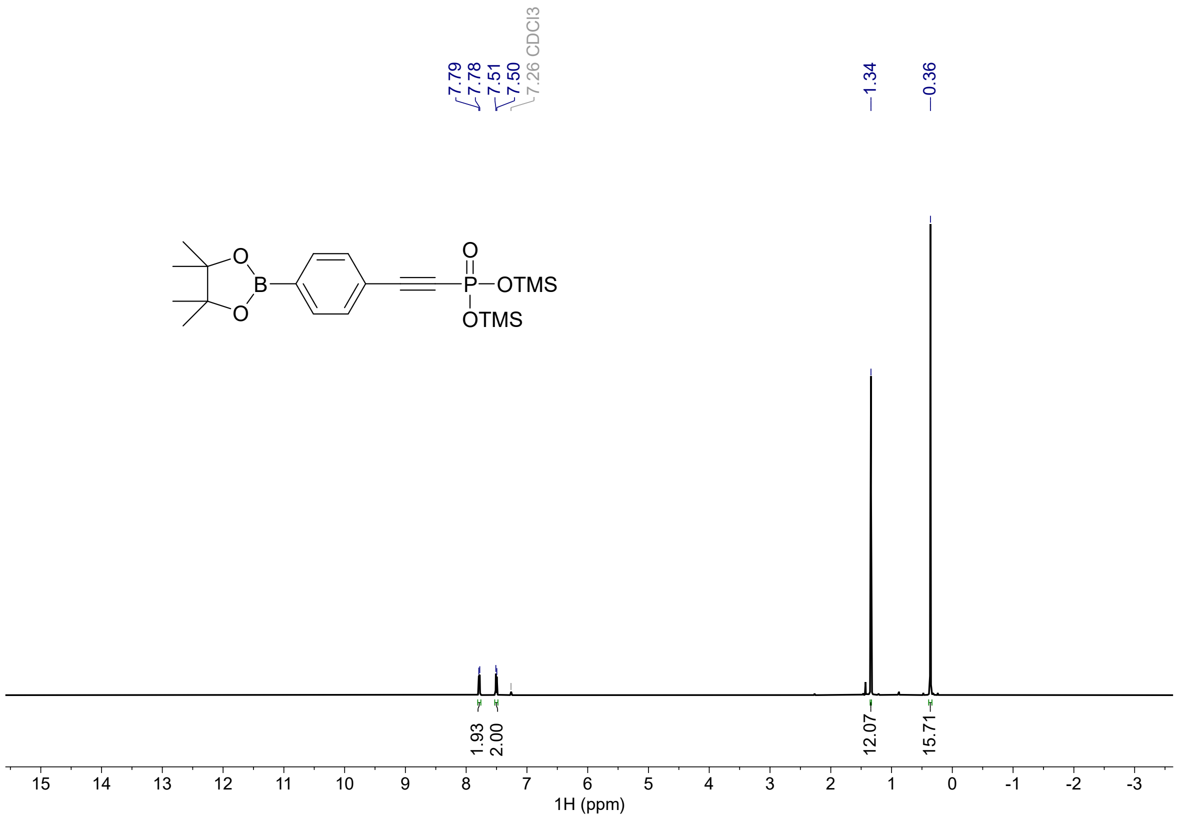

Figure S105: ^{1}H NMR spectrum of **1t′** in $CDCl_3$ at 25 °C, recorded at 500 MHz.

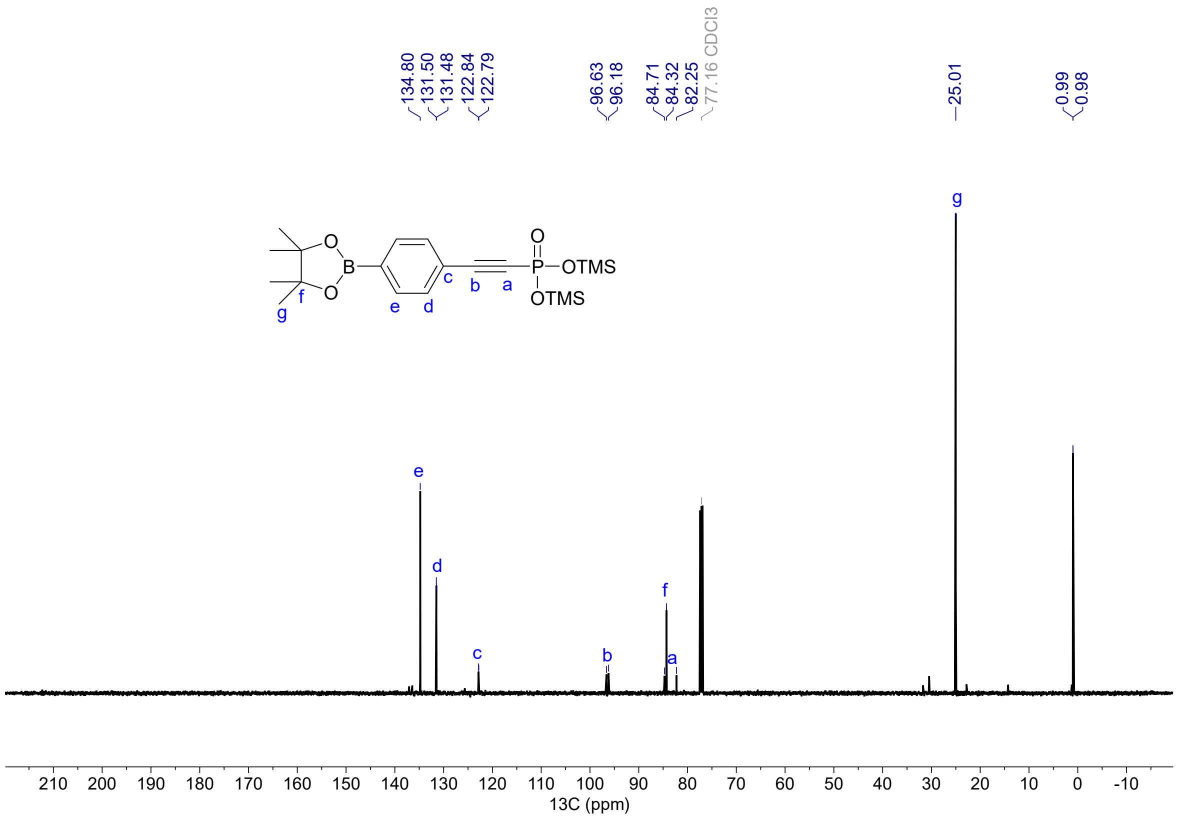

Figure S106: ^{13}C NMR spectrum of **1t′** in $CDCl_3$ at 25 °C, recorded at 126 MHz.

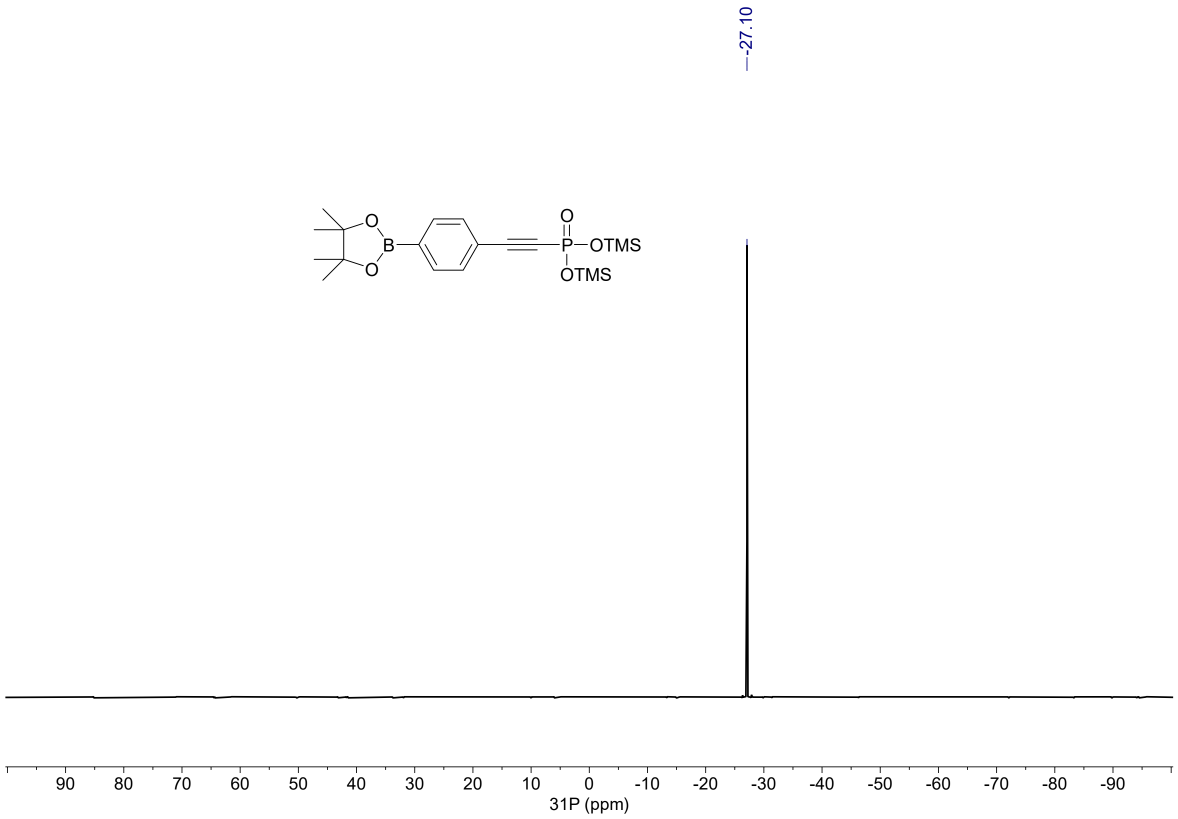

Figure S107: ^{31}P NMR spectrum of **1t′** in $CDCl_3$ at 25 °C, recorded at 203 MHz.

S7 Synthesis of 2 from Graham's salt and bio-polyP

S7.1 Breaking down Graham's salt

In the glovebox, a 125 mL stainless steel milling jar was charged with Na_3PO_4 (1.64 g, 10 mmol), Graham's salt (1.02 g, 10 mmol based on $NaPO_3$) and 30 grinding balls. The milling jar was sealed with the safety closure device, removed from the glovebox, and mounted onto the planetary ball mill. Milling was first conducted at a rotational speed of 200 rpm for 10 min to mix up the starting materials, and then conducted at 450 rpm for 4 h (with 30 min of break time and direction reversal after every 30 min of grinding). After cooling to ambient temperature, the jar was brought back into the glovebox and opened. An aliquot (ca. 30 mg) was removed from the jar and transferred into a 20 mL scintillation vial. The vial was brought out of the glovebox and the solid was dissolved in D_2O (2 mL). Analysis of this solution by ^{31}P NMR spectroscopy showed that it consisted of 89% of $P_2O_7^{4-}$ and 11% PO_4^{3-} (Figure S108) This solid material was used directly in the next reaction.

S7.2 Synthesis of 2 from Graham's salt

In the glovebox, to the jar containing the solid material from last step was added Na_2C_2 (1.05 g, 15 mmol). The milling jar was sealed again with the safety closure device, removed from the glovebox, and mounted onto the planetary ball mill. Milling was first conducted at a rotational speed of 200 rpm for 10 min to mix up the starting materials, and then conducted at 450 rpm for 18 h (with 30 min of break time and direction reversal after every 30 min of grinding). After working up the reaction according to S4.1, compound **2** was isolated as an off-white powder (0.87 g, 4.2 mmol, 42%).

S7.3 Breaking down bio-polyP

Bio-produced polyphosphate (bio-polyP) was obtained following the procedure we recently reported.[S12]

In the glovebox, a 125 mL stainless steel milling jar was charged with bio-polyP (1.22 g, ca. 9.2 mmol of P, average chain length 8.1, Figure S109) and 30 grinding balls. The milling jar was sealed with the safety closure device, removed from the glovebox, and mounted onto the planetary ball mill. Milling was first conducted at 450 rpm for 1 h. After cooling to ambient temperature, the jar was brought back into the glovebox and opened. An aliquot (ca. 20 mg) was removed from the jar, brought out and analyzed by ^{31}P NMR spectroscopy in D_2O, showing a mixture of phosphates with an average chain length of 3.0 (Figure S110). To the jar was added Na_3PO_4 (0.60 g, 3.6 mmol). The mixture was ball milled again at 450 rpm for 1 h. Analysis of an aliquot (ca. 20 mg) showed an average chain length of 1.7 (Figure S111). This solid material was used directly in the next reaction.

S7.4 Synthesis of 2 from bio-polyP

In the glovebox, to the jar containing the solid material from last step was added Na_2C_2 (0.70 g, 10 mmol). The milling jar was sealed again with the safety closure device, removed from the glovebox, and mounted onto the planetary ball mill. Milling was first conducted at a rotational speed of 200 rpm for 10 min to mix up the starting materials, and then conducted at 450 rpm for 8 h (with 30 min of break time and direction reversal after every 30 min of grinding). After working up the reaction according to S4.1, compound **2** was isolated as an off-white powder (0.41 g, 2.0 mmol, 31%).

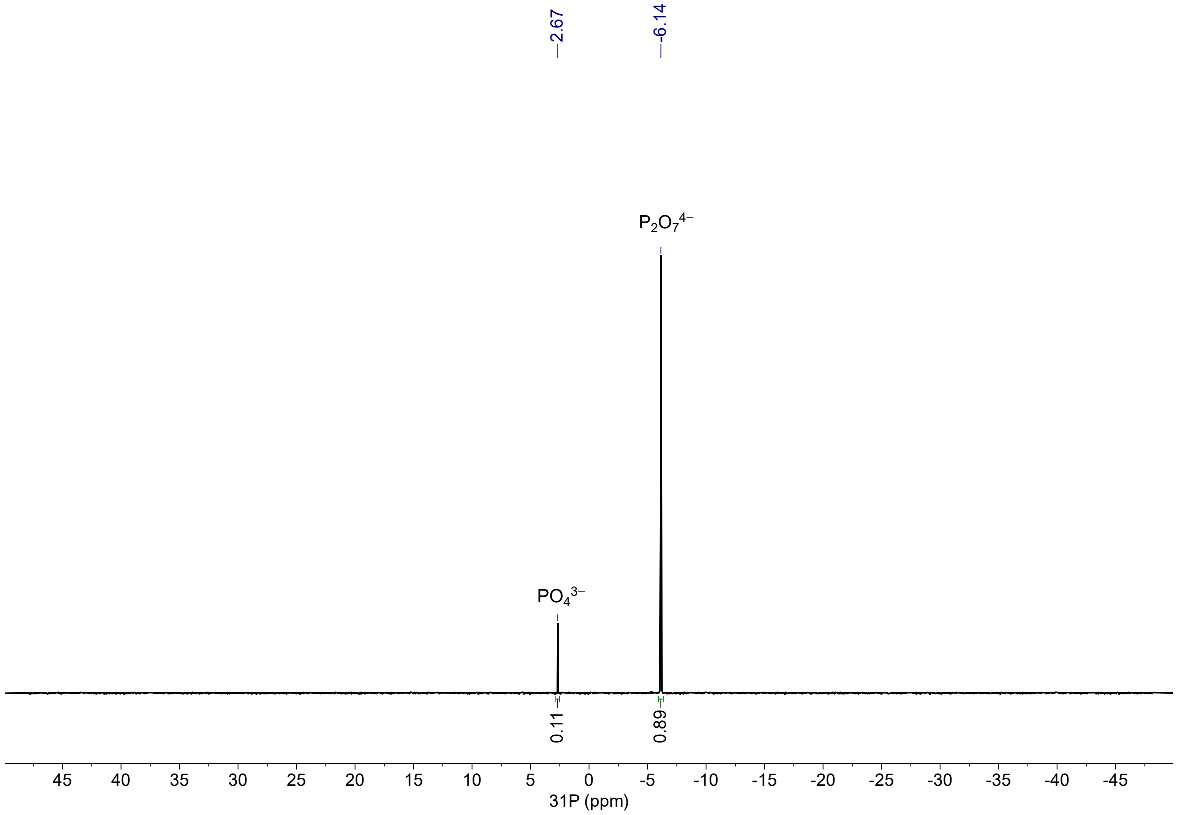

Figure S108: ^{31}P NMR spectrum of the mixture resulting from reaction of Graham's salt and Na_3PO_4 after 4 h.

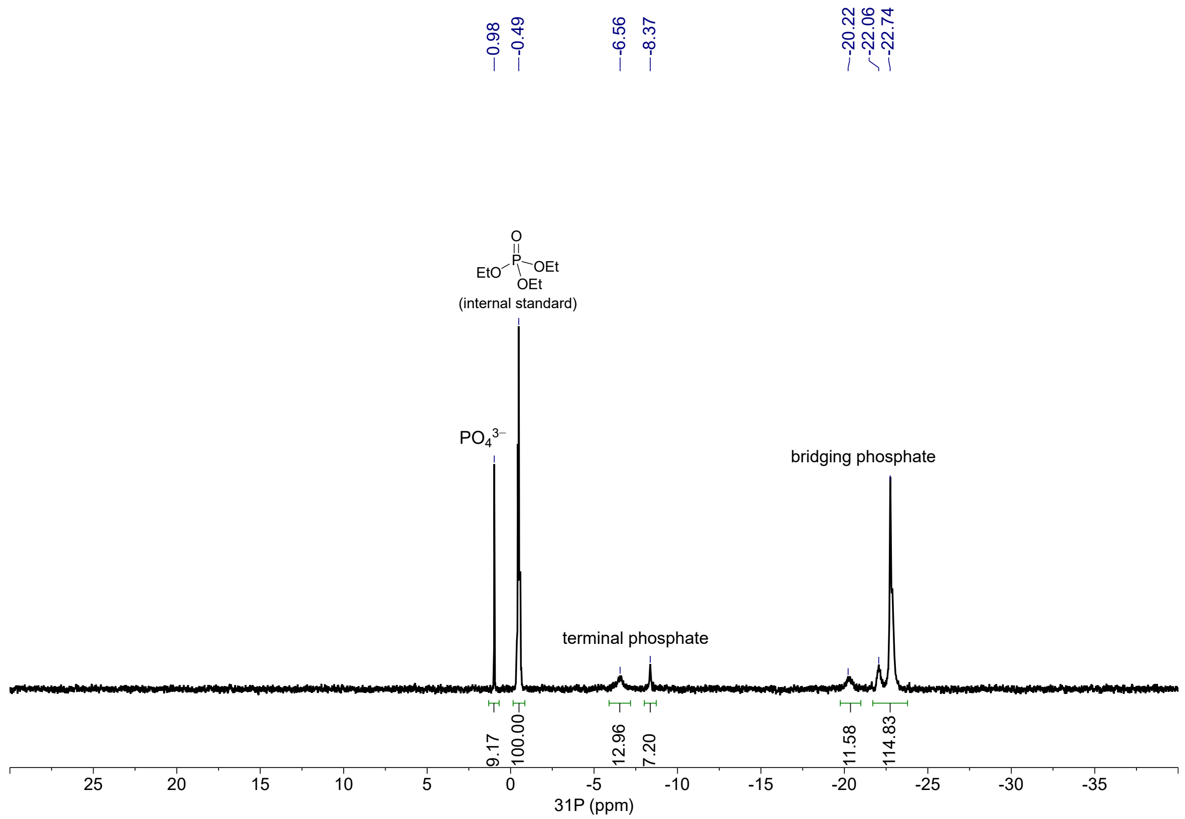

Figure S109: ^{31}P NMR spectrum of bio-polyP (18.2 mg of bio-polyP with 16.1 mg of $PO(OEt)_3$ added).

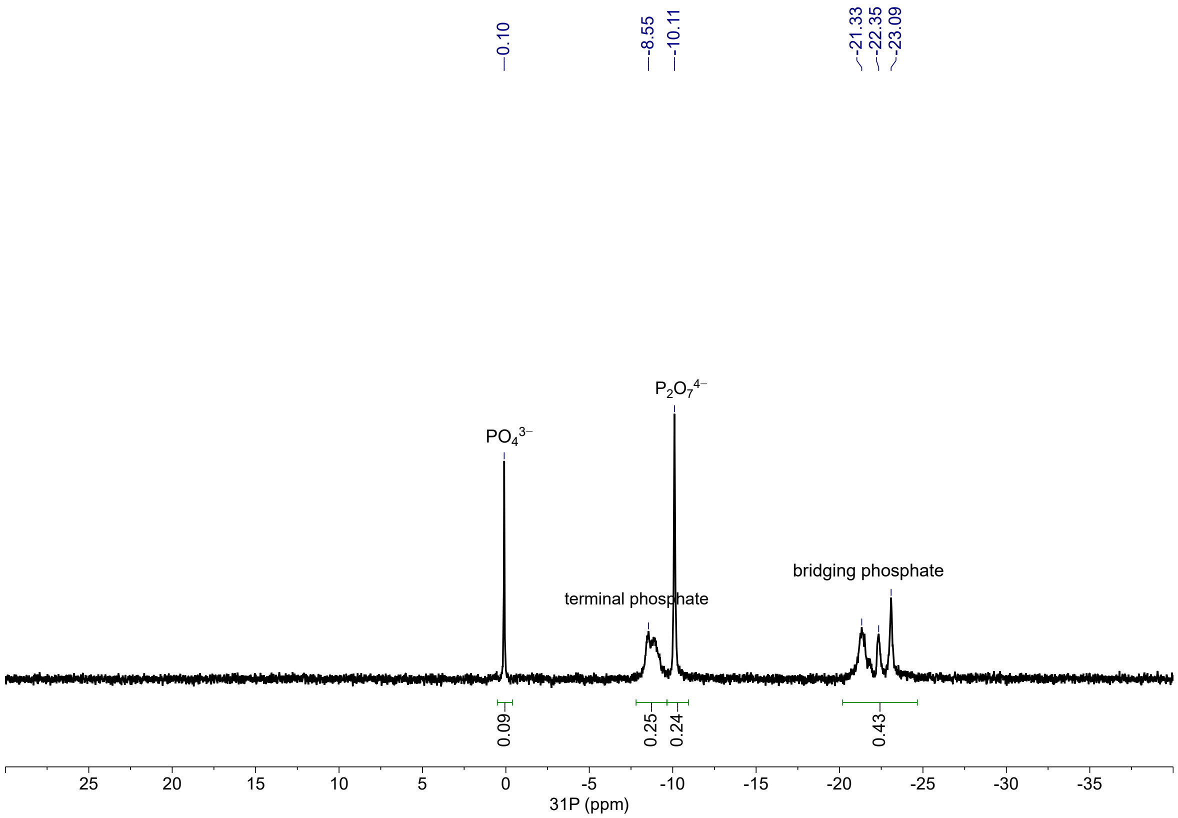

Figure S110: ^{31}P NMR spectrum of bio-polyP after grinding for 1 h.

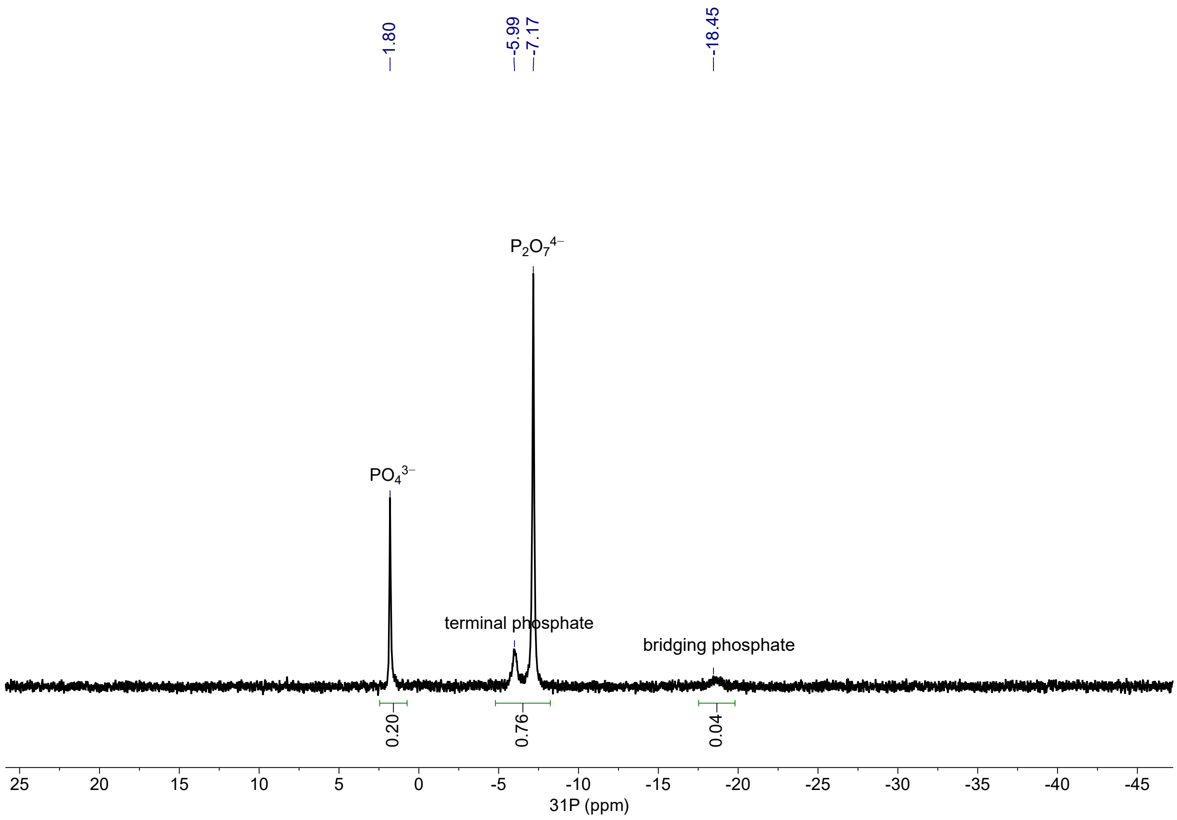

Figure S111: ^{31}P NMR spectrum of the mixture resulting from grinding bio-polyP and Na_3PO_4 after 1 h.

S8 References

References

(S1) Pangborn, A. B.; Giardello, M. A.; Grubbs, R. H.; Rosen, R. K.; Timmers, F. J. Safe and Convenient Procedure for Solvent Purification. *Organometallics* **1996**, *15*, 1518–1520.

(S2) Williams, D. B. G.; Lawton, M. Drying of Organic Solvents: Quantitative Evaluation of the Efficiency of Several Desiccants. *J. Org. Chem.* **2010**, *75*, 8351–8354.

(S3) Klöss, K.-H.; Hinz-Hübner, D.; Ruschewitz, U. Über eine neue Modifikation des Na_2C_2. *Z. Anorg. Allg. Chem.* **2002**, *628*, 2701–2704.

(S4) McAllister, T. E.; Nix, M. G.; Webb, M. E. Fmoc-chemistry of a stable phosphohistidine analogue. *Chem. Commun.* **2011**, *47*, 1297–1299.

(S5) Wanat, P.; Walczak, S.; Wojtczak, B. A.; Nowakowska, M.; Jemielity, J.; Kowalska, J. Ethynyl, 2-propynyl, and 3-butynyl C-phosphonate analogues of nucleoside di-and triphosphates: Synthesis and reactivity in CuAAC. *Org. Lett.* **2015**, *17*, 3062–3065.

(S6) Walczak, S.; Sikorski, P. J.; Kasprzyk, R.; Kowalska, J.; Jemielity, J. Exploring the potential of phosphotriazole 5' mRNA cap analogues as efficient translation initiators. *Org. Biomol. Chem.* **2018**, *16*, 6741–6748.

(S7) Xu, X.; Chen, H.; Wang, Y.; Gao, Y.; Tang, G.; Zhao, Y. Catalyst-free synthesis of cycloalkenyl phosphonates. *RSC Adv.* **2014**, *4*, 14740–14743.

(S8) Hu, G.; Chen, W.; Fu, T.; Peng, Z.; Qiao, H.; Gao, Y.; Zhao, Y. Nickel-catalyzed C–P cross-coupling of arylboronic acids with P(O)H compounds. *Org. Lett.* **2013**, *15*, 5362–5365.

(S9) Chen, Q.; Yan, X.; Du, Z.; Zhang, K.; Wen, C. P-arylation of dialkyl phosphites and secondary phosphine oxides with arynes. *J. Org. Chem.* **2016**, *81*, 276–281.

(S10) Kendall, A. J.; Salazar, C. A.; Martino, P. F.; Tyler, D. R. Direct conversion of phosphonates to phosphine oxides: An improved synthetic route to phosphines including the first synthesis of methyl JohnPhos. *Organometallics* **2014**, *33*, 6171–6178.

(S11) Qian, K.; Shepard, S. M.; Xin, T.; Park, G.; Cummins, C. C. Stabilized Molecular Diphosphorus Pentoxide, $P_2O_5L_2$ (L= N-Donor Base), in the Synthesis of Condensed Phosphate–Organic Molecule Conjugates. *J. Am. Chem. Soc.* **2023**, *145*, 6045–6050.

(S12) Zhai, F.; Xin, T.; Geeson, M. B.; Cummins, C. C. Sustainable production of reduced phosphorus compounds: Mechanochemical hydride phosphorylation using condensed phosphates as a route to phosphite. *ACS Cent. Sci.* **2022**, *8*, 332–339.
